# Supplementary material for: Diastereoselective dearomatization of indoles via photocatalytic hydroboration on hydramine-functionalized carbon nitride
Source: Nat Commun. 2024 May 22;15:4371. doi: 10.1038/s41467-024-48769-1 (PMC11111752; doi:10.1038/s41467-024-48769-1)
Supplement: Supplementary file 1 — Supplementary Information [file 41467_2024_48769_MOESM1_ESM.pdf]

Supplementary Information

Diastereoselective dearomatization of indoles via photocatalytic hydroboration on hydramine-functionalized carbon nitride

Contents

1. General Information....3

2. Complementary Reaction Optimization Data....15

3. General Procedures for Hydroboration of Indole Ester....16

4. Starting Material Preparation....16

5. Analytical Data of the Products....25

6. Gram-Scale Preparation of **3a** and transformations of the Products....40

7. Additional Experiments to Elucidate the Mechanism....44

8. Quantum Yield Measurement....49

9. X-Ray Crystal Data....51

10. Computational Calculation Details on Reaction Pathways....54

^{1}H, ^{19}F, ^{11}B, ^{13}C-NMR Spectra....55

References....126

1. General Information

Chemicals and solvents were purchased from commercial suppliers and used as received. ^{1}H NMR, ^{13}C NMR, ^{19}F NMR, ^{11}B NMR spectra were recorded on a Bruker AV-III400 (400 MHZ) or AMX500 (500 MHz) spectrometer. Chemical shifts were calibrated using residual undeuterated solvent as an internal reference ($CDCl_3$: 7.26 ppm ^{1}H NMR, 77.0 ppm ^{13}C NMR; CD_3OD: 4.87 ppm ^{1}H NMR, 49.0 ppm ^{13}C NMR). Multiplicity was indicated as follows: s (singlet), d (doublet), t (triplet), q (quartet), m (multiplet), dd (doublet of doublet). High-resolution mass spectra (HRMS) were obtained on Agilent 7200 GC-QTOF spectrometer (EI). Cyclic voltammetry was performed using Anhui Chem-n Instrument Co., Ltd. Vertex. C. EIS Chenhua (China). Stern-Volmer fluorescence quenching was performed using a Spectrofluorophotometer Anhui Chem-n Instrument Co., Ltd. RF-6000 Shimadzu Corporation.

1.1 Synthetic protocol of photocatalysts

Polymeric carbon nitride (**CN**) was synthesized by a typical high-temperature pyrolysis process with dicyandiamide as the precursor. 10 g of dicyandiamide powders were loaded in a 50 mL ceramic crucible with a lid and heated to 550 °C in a tube oven with a heating rate of 7 °C·min^{-1} and kept in air for 4 h. 1 g of the yellow solid was ground and transferred to a porcelain boat and heated a temperature of 500 °C for 2 h at a ramping rate of 10 °C min^{-1}.

CN-V was synthesized as follows: 10 g of dicyandiamide powders dispersed in 100 mL water was heated at 80 ºC until it completely dissolved and then 1, 2.5, 5 and 10 ml of 10 vol.% ethanolamine (ETA) aqueous solution was added. The mixture was kept heating at 80 ºC for 2 h, then vapored the water to obtain the faint yellow solid. It was washed with deionized (DI) water, and ethanol and dried at 80 ºC under vacuum for 6 h. The solids were then heated to 550 °C/4 h in a tube oven with a heating rate of 7 °C·min^{-1}, after naturally cooled to room temperature, further grounded into the fine powder and again calcined at 500 °C for 2 h at a ramping rate of 10 °C min^{-1} with air atmosphere. The synthesized catalyst by adding 1, 2.5, 5 and 10 ml of 10 vol.% ethanolamine (ETA) is denoted as CN-V_1, CN-$V_{2.5}$, CN-V_5, and CN-V_{10}. In this manuscript, unless otherwise specified, **CN-V** refers to **CN-V_5**.

1.2 Characterization of carbon nitride photocatalysts

The morphology and microstructure images were observed by using a Field Emission Hitachi SU8220 scanning electron microscope (SEM) and a JEOL JEM-2100 transmission electron microscope (TEM) with an accelerating voltage of 200 kV. The thickness of the lamellar sample was measured by using Bruker Multimode 8 Atomic force microscopy (AFM). X-ray diffraction (XRD) patterns were acquired using a Bruker D8 advance X-ray powder diffractometer (Cu Kα1 radiation) with a scan 2θ range of 5-60 degrees. Fourier transform infrared (FTIR) spectra were collected on a Thermo Fisher Nicolet IS50 II infrared spectrophotometer with a range of 4000-400 cm^{-1}. X-ray photoelectron spectroscopy (XPS) spectra were monitored on a Thermo Fisher K-Alpha$^+$ X-ray photoelectron spectrometer with a monochromatic Al Kα source. The binding energy referenced to the adventitious C 1s peak at 284.80 eV. The molar ratios of carbon, nitrogen and hydrogen were determined on an organic elemental analyzer Elementar vario MACRO cube. The nitrogen absorption-desorption isotherms were operated on a Micromeritics ASAP 2020 instrument at 77 K to determine the specific surface area and pore volume. The vacuum degassing pretreatment was required for 8 h at 150 °C before the sorption measurements. Diffuse reflectance UV-Vis spectra (DRS) were recorded with a a Hitachi U-4100 spectrophotometer referenced to barium sulfate ($BaSO_4$), the corresponding optical band gaps were determined by converted the diffuse reflectance spectrum using the Kubelka-Munk function. Time-resolved photoluminescence spectroscopy were recorded on an Edinburgh PLS980 fluorescence spectrometer with a 450 W ozone free xenon arc lamp by utilizing time-correlated single-photon count. Solid electron spin resonance (ESR) signals were quantitative collected from Bruker model A300 electron paramagnetic resonance at room temperature, equipped with a 300 W xenon arc lamp.

ArSH temperature-programmed desorption (**ArSH**-TPD) was carried out on a Micromeritics Autochem II 2920 Chemisorption Analyzer. Before analysis, 10 mg samples having the size of 0.2-0.3 mm was sealed in the cylindrical glass microreactor with a 2 mm internal diameter in the TPD device to obtain a 0.2 cm height fixed clay-bed, which was then endured the degass pretreatment at 400 °C for 4 h by using a He

gas flow (30 mL/ min) at normal pressure. The operations conditions were essential requirements for achieving adsorption–desorption equilibrium and to prevent diffusion retard. The clay sample was then cooled down to 35 °C. Subsequently, the target **ArSH** liquid molecule was injected through the clay fixed bed at the temperature of 120 °C. To acqiure the saturation **ArSH** adsorption, it was operating with a pulse counting mode, 20 pluse numbers (120 s/time) were monitored in the measurement, then using the He gas to purged the excess probe molecule and heated it to 400 °C with a rising rate of 10 °C /min to obtain the **ArSH**-TPD spetra.

The X-ray absorption near-edge structure (XANES) measurements of carbon and nitrogen K-edge have been performed at the BL20A beam line of synchrotron radiation at the Singapore Synchrotron Light Source. High-resolution X-ray diffraction (XRD) patterns were collected from a Rigaku SmartLab diffractometer in the 2θ range from 3 to 30° with 0.001° data binning equipped with Mo X-ray anode and using a 0.5 mm borosilicate glass holder. Each sample was collected by employing a very slow speed of 0.4° min^{-1} for 60 min. The X-ray total scattering data was then treated with PDFgui refinement of finite-size NCs and we applied the Rigaku in-built software for the subtraction of air and capillary background.

The photocurrent measurements applied cyclic chopped light were carried out in a standard three electrode system by using a CHI760e electrochemical workstation. Electrochemical impedance spectroscopy (EIS) was investigated under an open potential from 0.01 to 10 KHz. Mott-Schottky plots were conducted by employing three different frequencies of AC potential at 2000, 2500, and 3000 Hz to determine the flat-band potential. The work electrode was prepared by using the drop-casting methods. The catalyst was first sonicated to obtain a uniform slurry and then was dipped onto the F-doped tin oxide (FTO) glass. A carbon rod was applied as the counter electrode, saturated Ag/AgCl (3.5 M KCl) as the reference electrode, and 0.2 M $NaSO_4$ solution was used as the electrolyte for during the electrochemical measurements.

The calculations on materials were performed by using the Vienna ab initio simulation package (VASP) based on density functional theory (DFT). The exchange-correlation functional was approximated as a spin-polarized generalized gradient approximation

(GGA) with the Perdew-Burke-Ernzerhof (PBE), all-electron plane-wave basis sets with an energy cutoff of 400 eV, and the electronic structure calculation was presented by a projector augmented wave (PAW) method. Polymeric carbon nitride was simulated involved using a surface model of p (2 × 2) unit cell periodicity. The Brillouin-zone integrations was sampled using a (3 × 3 × 1) Monkhorst-Pack mesh. To calculate the electronic density of states (DOS), a higher 9 × 9 × 1 k-point mesh was exploited for the (2 × 2) supercells. The conjugate gradient algorithm was further applied for optimization. The convergence threshold criterion was set as 1×10^{-4} eV in total energy and 0.05 eV/Å in force criterion on each atom. The adsorption energy change (ΔE_{abs}) was measured as follows: $\Delta E_{abs} = E_{total} - E_{sur} - E_{mol}$ where E_{total} is a measure of the total energy for the adsorption state, E_{sur} is the energy of the pure surface, and E_{mol} is the energy of the molecule.

1.3 Figures of carbon nitride photocatalysts

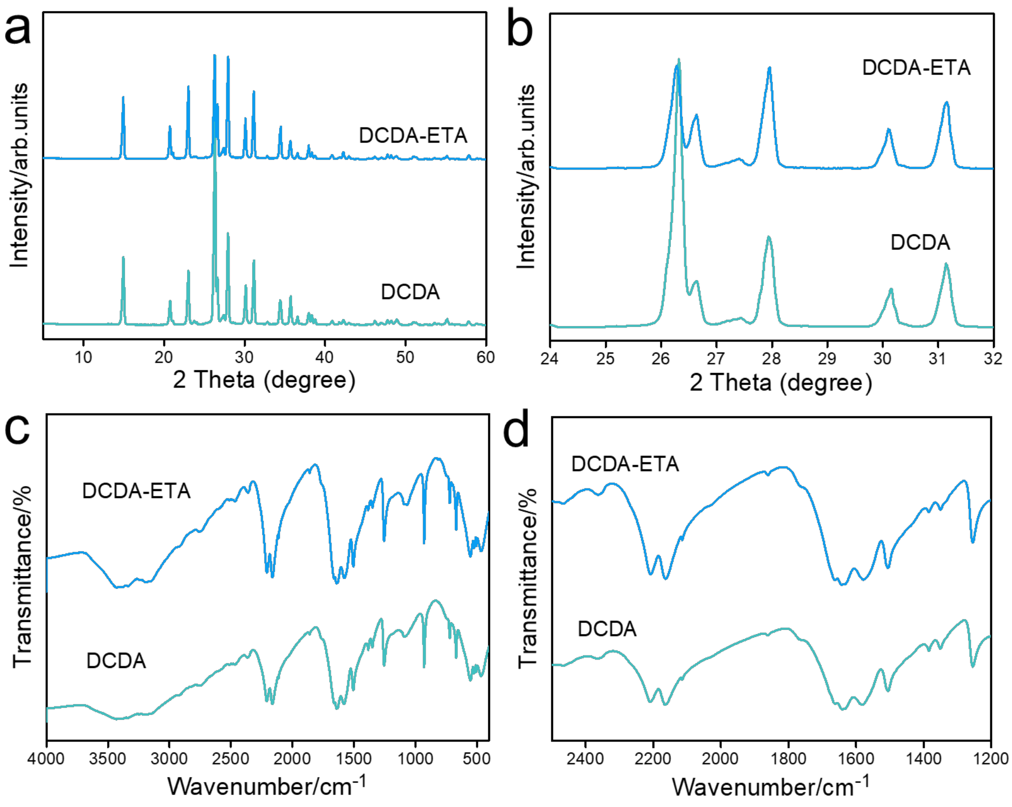

Supplementary Figure 1. Characterization of DCDA and DCDA-ETA. (a-b) XRD patterns and (c-d) FTIR spectra of DCDA and DCDA-ETA.

Note: The isolate precursor by heating dicyandiamide (DCDA) with ethanolamine

(ETA) is denoted as DCDA-ETA. From the XRD patterns, the observed diffraction peaks of DCDA-ETA are largely consistent with those of DCDA, indicating that the crystalline structure of DCDA-ETA is the same as that of DCDA. The FTIR spectra of DCDA-ETA and DCDA are nearly identical, suggesting that the heating of DCDA with ETA does not alter the characteristic structure of DCDA.

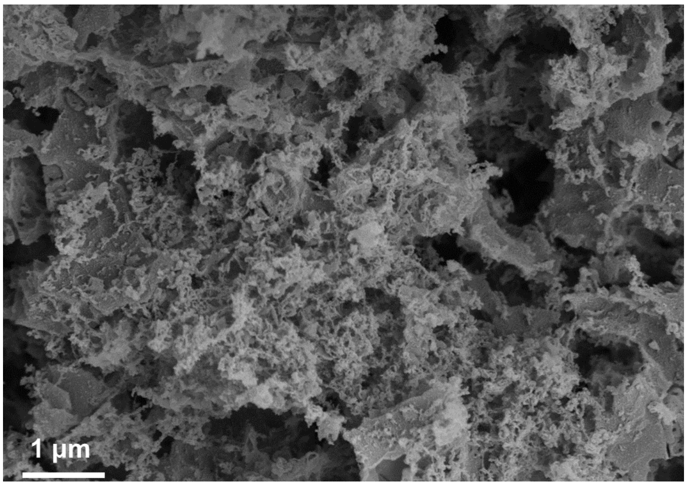

Supplementary Figure 2. SEM images of **CN-V**.

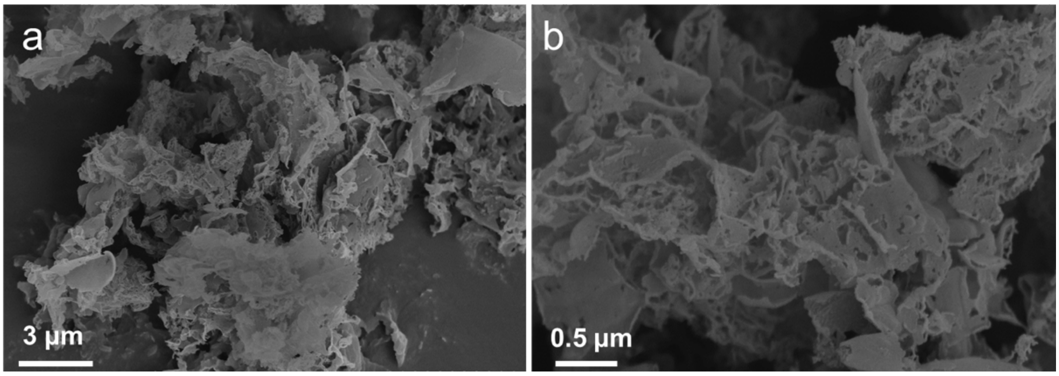

Supplementary Figure 3. SEM images of (a-b) **CN**.

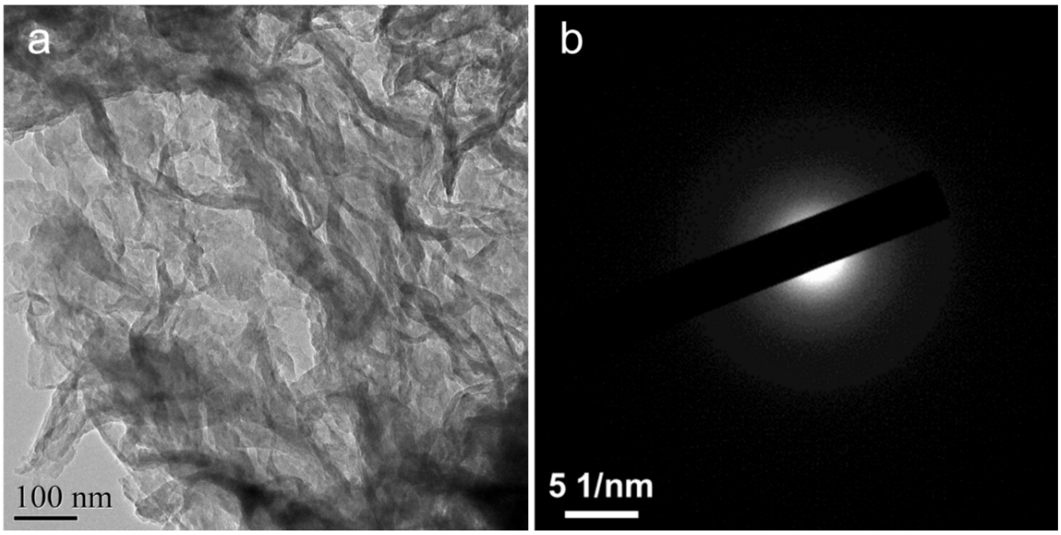

Supplementary Figure 4. Structure Characterization of **CN**. (a) TEM images of **CN** and (b) the selected area electron diffraction (SAED) pattern.

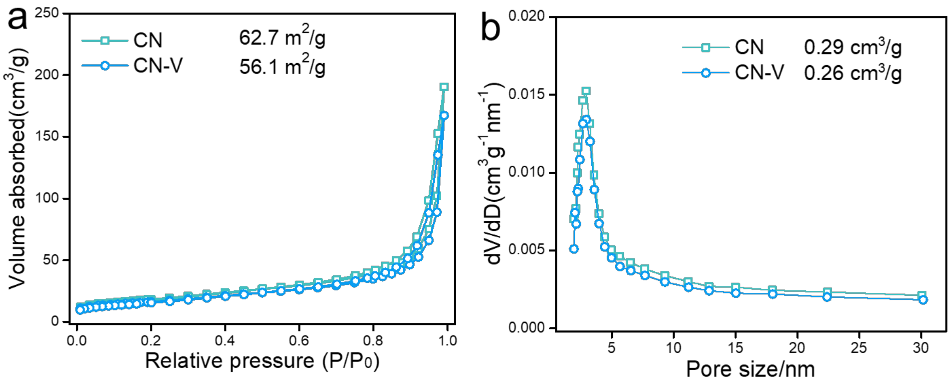

Supplementary Figure 5. N_2 absorption-desorption isotherms of the obtained samples. (a) The specific surface area and (b) the pore distribution plots of the resultant **CN** and **CN-V**.

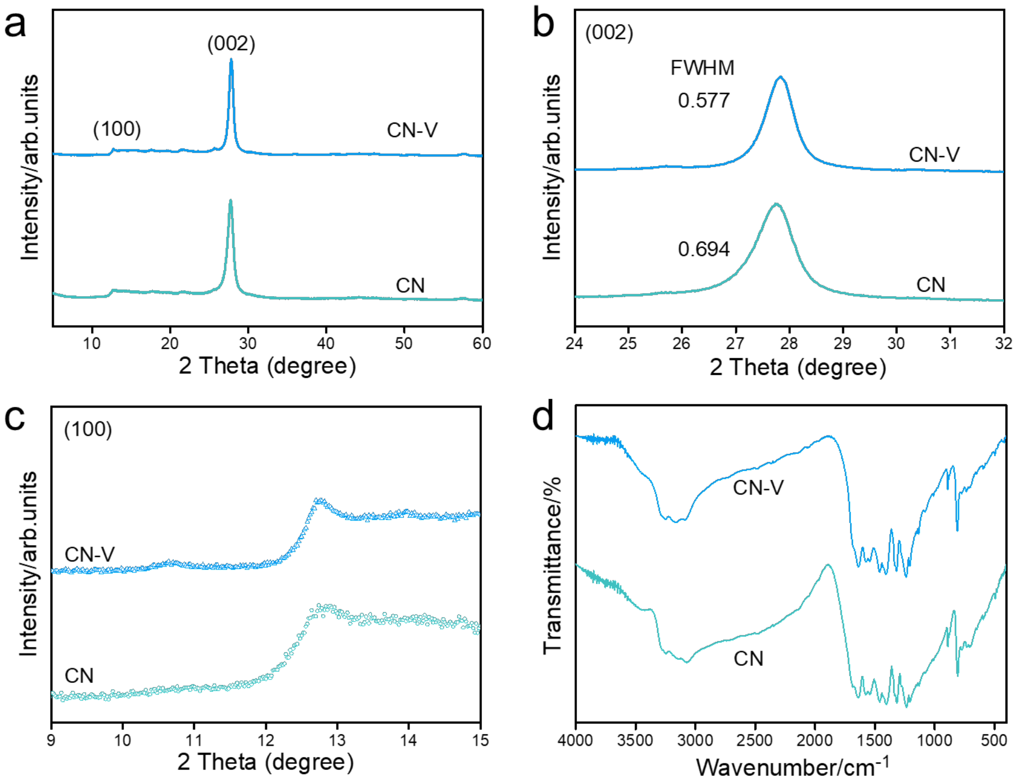

Supplementary Figure 6. Structure characterization of **CN** and **CN-V**. (a) XRD patterns, (b) the enlarged (002) and (c) the enlarged (100) plane in XRD patterns of **CN** and **CN-V**. (d) FTIR spectra of **CN** and **CN-V**.

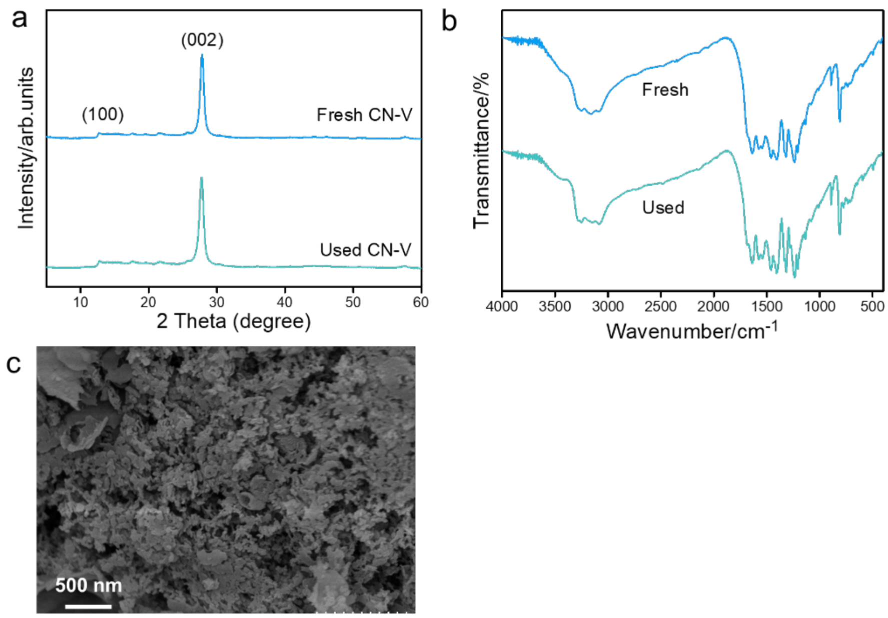

Supplementary Figure 7. Characterization of the used **CN-V**. (a) XRD patterns and

(b) FTIR spectra of the fresh and used **CN-V** sample, (c) The **SEM** image of the used **CN-V** sample.

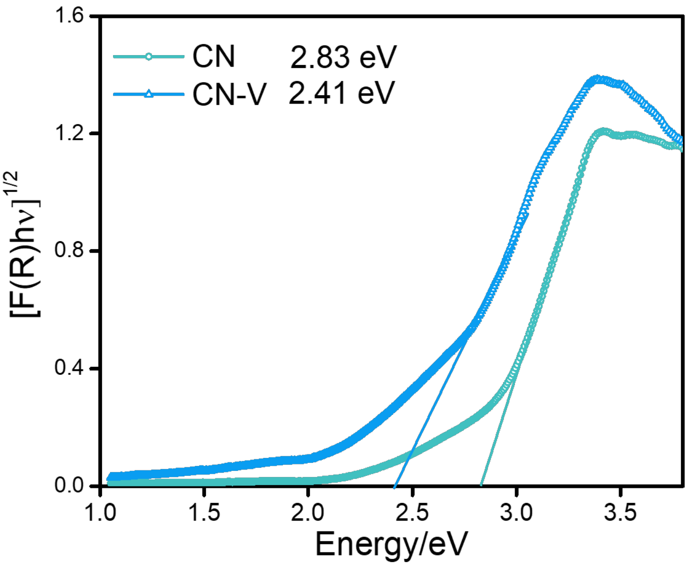

Supplementary Figure 8. The calculated band gap of catalyst samples converted using the Kubelka-Munk function from DRS spectra of **CN** and **CN-V**.

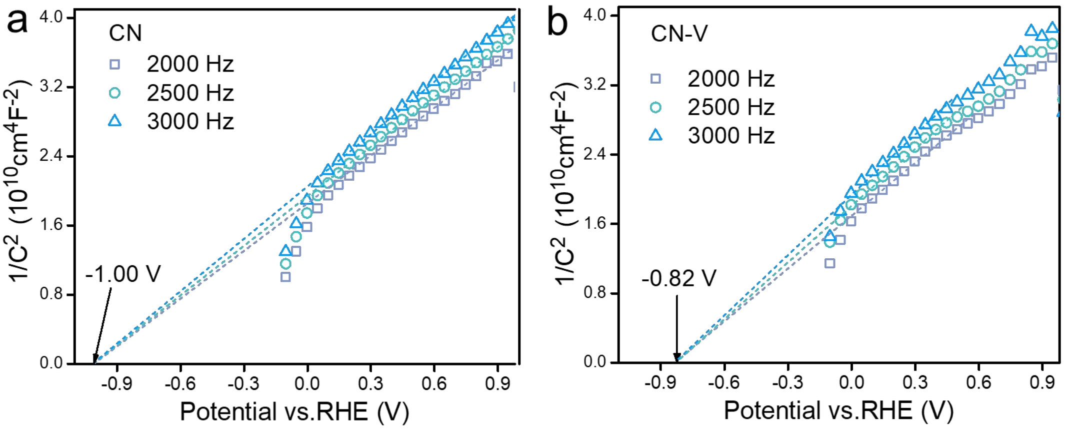

Supplementary Figure 9. Mott–Schottky (M–S) plots over the frequency of 2000, 2500, and 3000 Hz of (a) **CN** and (b) **CN-V**. Note that for many n-type semiconductors, the flat band potential is routinely considered to be about 0.2V below the conduction band (E_{cb}). Based on this, the estimated E_{cb} value of **CN-V** and **CN** is -1.02 V and -1.20

V vs RHE, respectively.

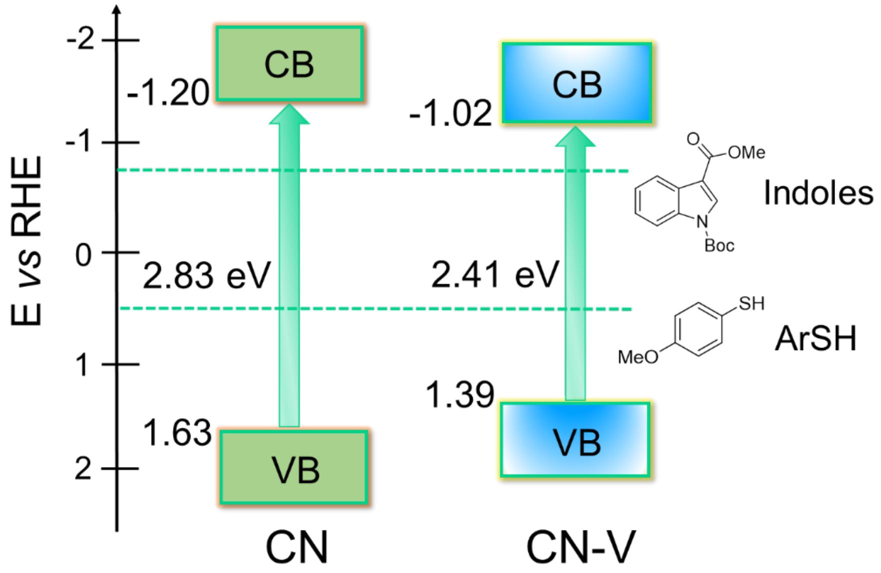

Supplementary Figure 10. Band structure alignments for **CN** and **CN-V** catalyst samples.

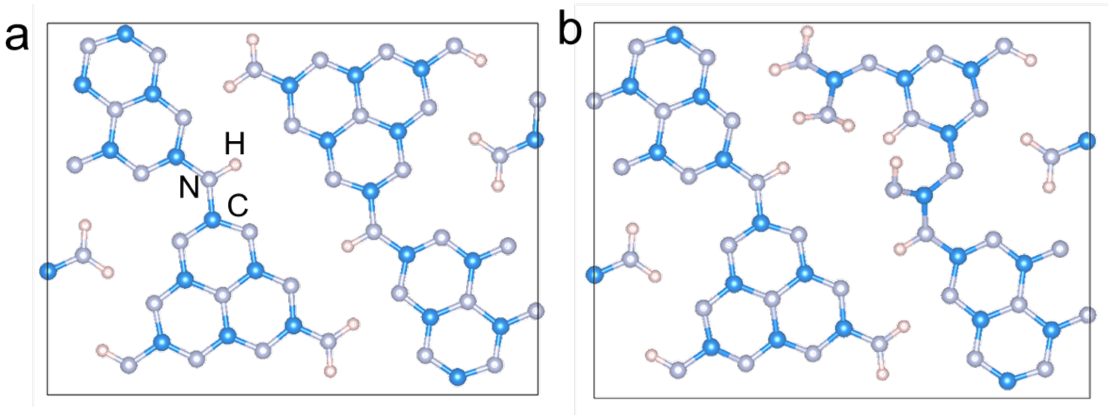

Supplementary Figure 11. The calculated DFT model of the structure cell, (a) the pristine model of **CN** and (b) the **CN-V** with carbon vacancies.

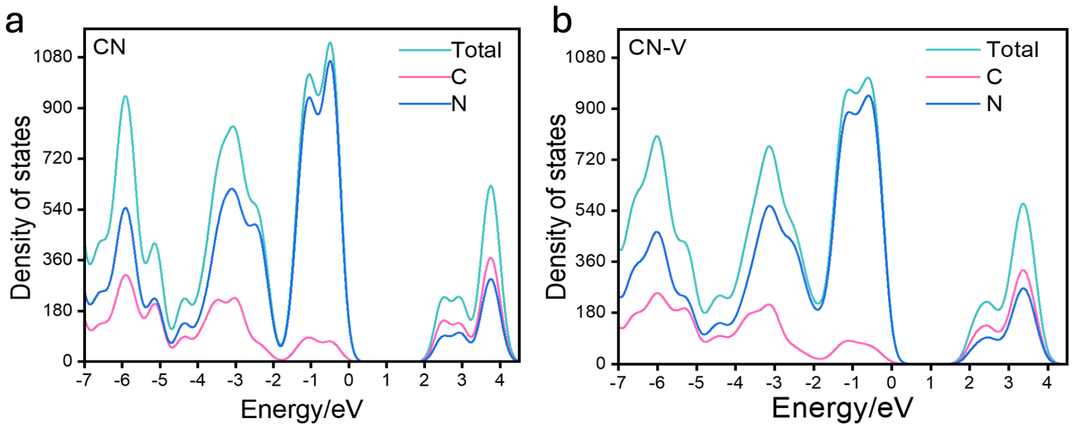

Supplementary Figure 12. Density of states (DOS) of (a) the pristine model of **CN** and (b) the **CN-V** with carbon vacancies.

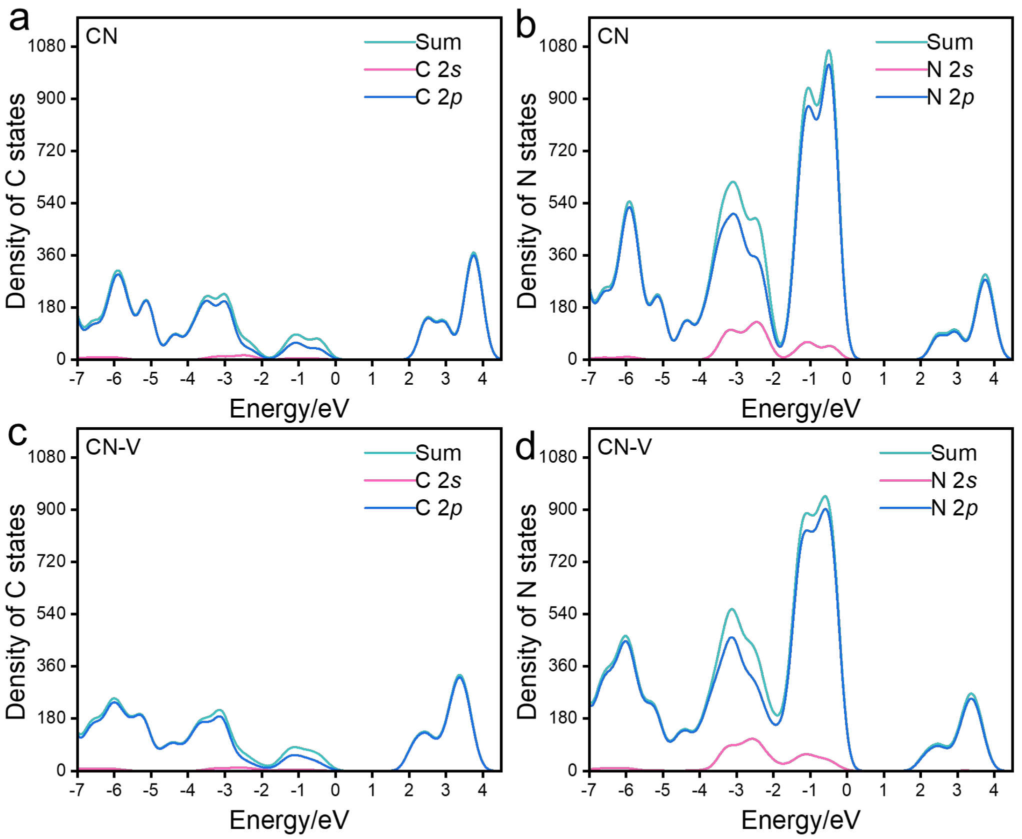

Supplementary Figure 13. The density of states of (a) C 2*s* and C 2*p* and (b) N 2*s* and N 2*p* in CN and (c) C 2*s* and C 2*p* and (d) N 2*s* and N 2*p* in CN-V.

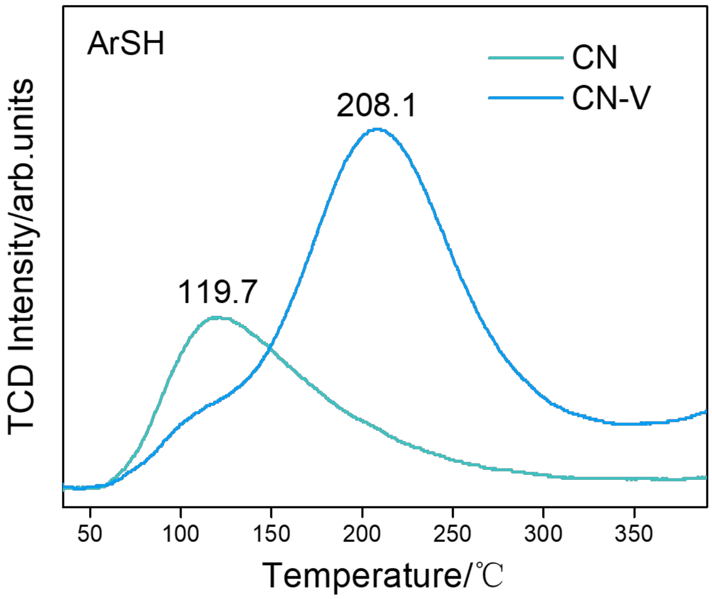

Supplementary Figure 14. ArSH temperature-programmed desorption (**ArSH**-TPD) spectra of **CN** and **CN-V**.

Supplementary Table 1. Organic elemental analysis of **CN** and **CN-V**.

**samples**	**N [%]**	**C [%]**	**H [%]**	**C/N[a]**
**CN**	58.4	32.5	1.7	0.65
**CN-V**	57.5	30.0	2.0	0.61

C/N[a]: mole ratio of C/N;

Supplementary Table 2. Surface C/N molar ratios of **CN** and **CN-V** as determined by XPS.

**Sample**	**C**		**N**		**C/N**
	**area**	**[%]**	**area**	**[%]**	
**CN**	301133	43.87	625636	54.53	0.80
**CN-V**	291261	42.02	646313	55.75	0.75

Supplementary Table 3. Relative ratios of C-N=C, N-$(C)_3$, and C-N-H of **CN**, and **CN-V** as determined from XPS N 1s spectra.

**samples**	**C=N-C**		**N-$(C)_3$**		**C-N-H**		**N-$(C)_3$/ C=N-C**
	**BE[a]**	**ratio[b]**	**BE[a]**	**ratio[b]**	**BE[a]**	**ratio[b]**	
**CN**	398.0	44.7	398.5	36.5	400.1	18.7	0.82
**CN-V**	397.9	47.1	398.4	31.5	399.9	21.3	0.67

BE[a]: Binding Energy; **ratio[b]:** calculated area ratio versus the total area;

2. Complementary Reaction Optimization Data

Supplementary Table 4. Screening of CN-V photocatalysts

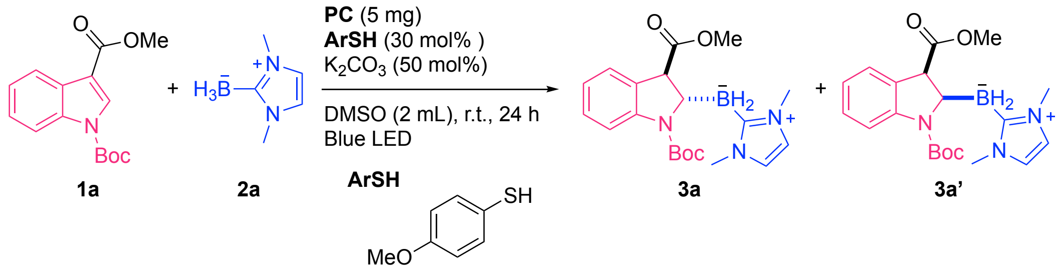

entry	deviation	Yield% of **3a**[a]	Yield% of **3a'**[a]
1	**CN-V$_1$**	79	0
2	**CN-V$_{2.5}$**	86	0
3	**CN-V$_5$**	96	0
4	**CN-V$_{10}$**	81	0
5	**CN**	63	0

Reaction conditions: 1-(*tert*-butyl) 3-methyl 1*H*-indole-1,3-dicarboxylate (**1a**, 0.10 mmol), NHC-borane (**2a**, 0.15 mmol), **PC** (5 mg), **ArSH** (30 mol%), K_2CO_3 (0.05 mmol), THF (2 mL), 18W blue LED irradiation, room temperature, 24 h. [a]Yields were determined by analysis of the crude ^{1}H NMR spectra using 1,3,5-trimethoxybenzene as an internal standard.

Note: The catalytic activity of the serial of **CN-V** serving as the photocatalyst (**PC**) was then examined thereafter for the dearomatization of indole derivatives via hydroboration, using 3-substituted indole (**1a**) and NHC-borane (**2a**) as the reaction partners with the cooperation HAT catalyst and bases. Compared to the production of diastereoselective trans-hydroboration products with a yield of 63% using **CN**, the introduction of ETA modification resulted in a improvement in

yield for **CN-V.** When the concentration of ETA increases gradually from 1 to 2.5 and then 5 ml during the CN-V prepared process, the yield of trans-hydroboration products is enhanced, reaching 79% for **CN-V$_1$**, 86% for **CN-V$_{2.5}$**, and 96% for **CN-V$_5$**, respectively. The **CN-V$_5$** exhibited the highest yield among the tested samples. However, excessive ETA addition in **CN-V$_{10}$** led to a decreased yield of 81%. Therefore, when the addition of a 10% volume fraction ETA aqueous solution is 5 ml, the prepared **CN-V$_5$** exhibits the best performance in photocatalysis process. In this manuscript, unless otherwise specified, **CN-V** refers to **CN-V$_5$**.

3. General Procedures for Hydroboration of Indole Ester

In a 20 mL Schlenk tube with a magnetic stir bar were placed **CN-V** (5 mg), K_2CO_3 (6.9 mg, 0.05 mmol, 50 mol%), and NHC-BH_3 (**2**, 0.15 mmol, 1.5 equiv). Under nitrogen atmosphere, indole ester (**1**, 0.1 mmol, 1 equiv), 4-methoxybenzenethiol (**ArSH**, 4 μL, 0.03 mmol, 30 mol%), DMSO (2 mL) were added, subsequently. The resulting mixture was sealed and degassed via freeze-pump-thaw three times. Then, the reaction was placed under a blue LED (2-meter strips, 20 W) and irradiated for 24 hrs at room temperature. To the resulting mixture was added water (3 mL), followed by eaxtraction with diethyl ether (5 mL × 3). The combined organic layer was washed with brine (10 mL × 3). The solvent was removed under vacuum. Silica gel chromatography (eluent: petroleum ether (PE)/EtOAc = 1/1) of the crude product afforded the desired compound.

4. Starting Material Preparation

4.1 Preparation of 1a-1d and 1i-1n

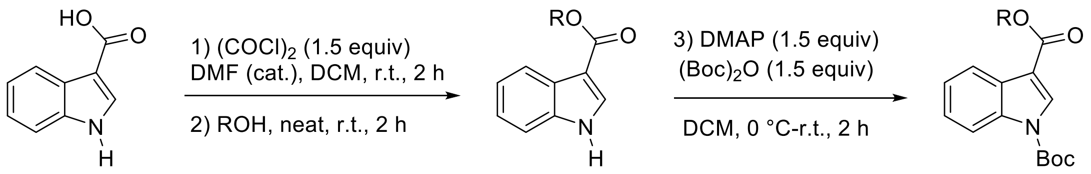

The synthesis of compounds **1a-1d** and **1i-1n** were adapted from literature procedures[1]. To a 100-mL flask charged with 1H-indole-3-carboxylic acid (5 mmol), DMF (0.1 mL), and DCM (50 mL) was added $(COCl)_2$ (0.96 g, 7.5 mmol) slowly at 0 °C. The resulting mixture was then allowed to warm to room temperature and stirred at the same temperature for 2 hrs. After concentration, alcohol (30 mL) was introduced, and the mixture was continued to stir at room temperature for an additional 2 hrs. The alcohol was then removed, and the residue was dissolved in THF (5 mL) followed by the addition of DMAP (0.92 g, 7.5 mmol) and Boc_2O (1.64 g, 7.5 mmol) at 0 °C. The reaction mixture was then allowed to warm to room temperature and stirred for 2 hrs. After concentration, the residue was purified by column chromatography on silica gel using PE/EtOAc (10:1) as the eluent to afford corresponding product.

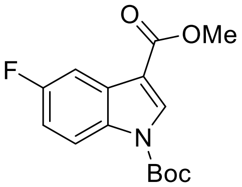

1-(tert-Butyl)-3-methyl-5-fluoro-1H-indole-1,3-dicarboxylate (1k): red solid (0.74 mg, 50%); Mp. 178 – 179 °C. ^{1}H NMR (400 MHz, $CDCl_3$) δ 8.28 (s, 1H), 8.13 (dd, *J* = 9.1, 4.6 Hz, 1H), 7.81 (dd, *J* = 9.2, 2.7 Hz, 1H), 7.09 (td, *J* = 9.1, 2.7 Hz, 1H), 3.94 (s, 3H), 1.68 (s, 9H); ^{13}C NMR (100 MHz, $CDCl_3$) δ 164.28, 161.12, 148.64, 133.19, 131.85, 128.49 (d, *J* = 10.9 Hz), 116.22 (d, *J* = 9.2 Hz), 113.05 (d, *J* = 25.3 Hz), 107.36 (d, *J* = 25.3 Hz), 85.35, 63.62, 51.51, 28.02; ^{19}F NMR (376 MHz, $CDCl_3$) δ -118.81 (m, 1F); HRMS (EI): Calcd for $C_{15}H_{17}FNO_4$ $[M+H]^+$ 294.2944, found 294.2943.

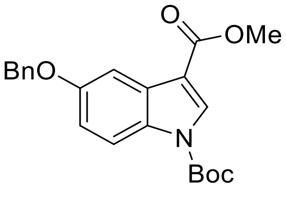

1-(tert-Butyl)-3-methyl-5-(benzyloxy)-1H-indole-1,3-dicarboxylate (1j): white solid (1.26 g, 66%); Mp. 165 – 166 °C. ^{1}H NMR (400 MHz, $CDCl_3$) δ 8.23 (s, 1H),

8.05 (d, J = 9.1 Hz, 1H), 7.75 (d, J = 2.6 Hz, 1H), 7.49 (d, J = 7.3 Hz, 2H), 7.40 (t, J = 7.4 Hz, 2H), 7.34 (d, J = 7.2 Hz, 1H), 7.06 (dd, J = 9.1, 2.6 Hz, 1H), 5.15 (s, 2H), 3.93 (s, 3H), 1.68 (s, 9H); ^{13}C NMR (100 MHz, $CDCl_3$) δ 164.73, 155.94, 148.89, 137.10, 132.37, 130.29, 128.54, 128.50, 127.92, 127.63, 115.97, 115.00, 111.78, 105.03, 84.95, 70.48, 51.42, 28.08; HRMS (EI): Calcd for $C_{22}H_{24}NO_5$ $[M+H]^+$ 382.4280, found 382.4283.

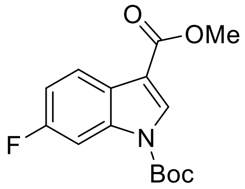

1-(tert-Butyl)-3-methyl-6-fluoro-1H-indole-1,3-dicarboxylate (1n): white solid (1.03 g, 70%); Mp. 187 – 188 °C. ^{1}H NMR (400 MHz, $CDCl_3$) δ 8.23 (s, 1H), 8.09 (dd, J = 8.8, 5.5 Hz, 1H), 7.90 (dd, J = 9.8, 2.4 Hz, 1H), 7.10 (td, J = 9.0, 2.4 Hz, 1H), 3.93 (s, 3H), 1.69 (s, 9H); ^{13}C NMR (100 MHz, $CDCl_3$) δ 164.38, 162.34, 148.64, 135.68 (d, J = 13.0 Hz), 132.10 (d, J = 3.5 Hz), 123.71 (d, J = 1.6 Hz), 122.51 (d, J = 9.8 Hz), 112.37, 102.50 (d, J = 28.8 Hz), 85.47, 63.61, 51.49, 28.02; ^{19}F NMR (376 MHz, $CDCl_3$) δ -116.04 (d, J = 5.6 Hz); HRMS (EI): Calcd for $C_{15}H_{17}FNO_4$ $[M+H]^+$ 294.2944, found 294.2945.

4.3 Preparation of 1o-1p

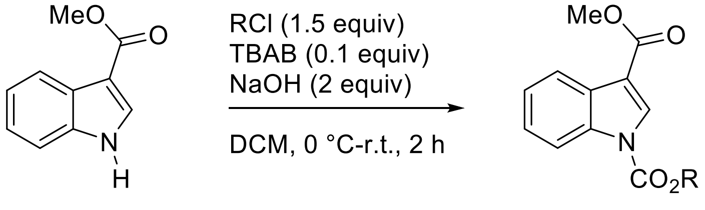

The synthesis of compounds **1o** and **1p** was adapted from literature procedures[2]. To a 100-mL flask charged with 3-subsitiuted indole (10 mmol), DCM (10 mL), TBAB (0.32 g, 1.0 mmol), and NaOH (0.80 g, 20 mmol) was added corresponding benzyl chloroformate or benzoyl chloride (15 mmol, 1.5 equiv) slowly at 0 °C. The resulting mixture was allowed to warm to room temperature and stirred for 2 hrs. The reaction

mixture was then diluted by 1 M HCl (20 mL) and extracted with DCM (3 × 20 mL). The combined organic phase was dried over Na_2SO_4. After removal of the solvent, the residue was purified by column chromatography on silica gel using PE/EtOAc (10:1) as the eluent to afford corresponding 1,3-disubstituted indole. The characterization data were in good accord with literature reports.[1]

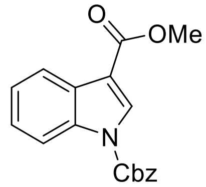

1-Benzyl 3-methyl 1H-indole-1,3-dicarboxylate (1o): White solid (2.93 g, 95%). ^{1}H NMR (400 MHz, $CDCl_3$) δ 8.28 (s, 1H), 8.20-8.14 (m, 2H), 7.49-7.32 (m, 7H), 5.46 (s, 2H), 3.91 (s, 3H); ^{13}C NMR (100 MHz, $CDCl_3$) δ 164.4, 150.2, 135.5, 134.4, 131.5, 129.0, 128.8, 128.7, 127.4, 125.4, 124.2, 121.7, 115.1, 113.0, 69.4, 51.5; HRMS (ESI) calcd for $C_{18}H_{16}NO_4$ $[M+H]^+$ 310.1079, found 310.1078.

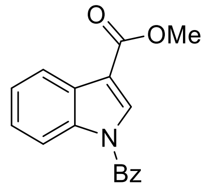

Methyl 1-benzoyl-1H-indole-3-carboxylate (1p): White solid (2.18 g, 78%). ^{1}H NMR (400 MHz, $CDCl_3$) δ 8.39 (d, J = 8.0 Hz, 1H), 8.20 (d, J = 7.2 Hz, 1H), 7.99 (s, 1H), 7.76 (d, J = 7.6 Hz, 2H), 7.68-7.65 (m, 1H), 7.59-7.55 (m, 2H), 7.46-7.41(m, 2H), 3.91(s, 3H); ^{13}C NMR (100 MHz, $CDCl_3$) δ 168.6, 164.4, 136.3, 133.4, 133.3, 132.7, 129.4, 128.9, 127.6, 125.7, 125.0, 121.6, 116.2, 113.2, 51.6; HRMS (ESI) calcd for $C_{17}H_{13}NNaO_3$ $[M+Na]^+$ 302.0793, found 302.0787.

4.4 Preparation of 1e-1h and 1w-1aa

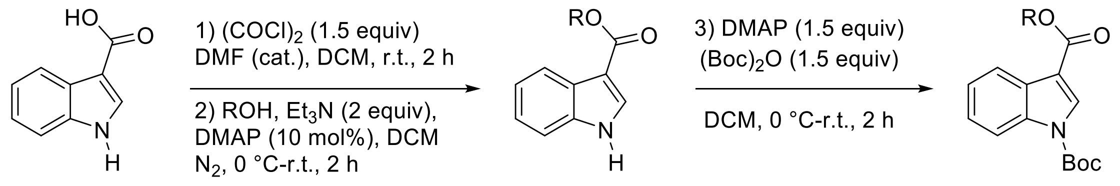

The synthesis of compounds **1e-1h** and **1w-1aa** were adapted from literature

procedures[1, 3]. To a 100-mL flask charged with a mixture of 1*H*-indole-3-carboxylic acid (0.81 g, 5 mmol), DCM (50 mL) and DMF (0.1 mL) was added $(COCl)_2$ (0.96 g, 7.5 mmol) slowly at 0 °C. The resulting mixture was then allowed to warm to room temperature and stirred at the same temperature for 2 hrs. After concentration, the mixture was cooled to 0 °C, stirred under an inert atmosphere of dry N_2. The corresponding alcohol (1.2 equiv) was dropwise added via syringe followed by dropwise addition of Et_3N (1.4 mL, 10 mmol) and then DMAP (61 mg, 0.5 mmol) at once. The mixture was continued to stir at room temperature for additional 2 hrs. The solvent was then removed and the residue was dissolved in DCM (50 mL) followed by addition of DMAP (0.92 g, 7.5 mmol) and Boc_2O (1.64 g, 7.5 mmol) at 0 °C. The reaction was then allowed to warm to room temperature and stir for 2 hrs. After concentration, the residue was purified by column chromatography on silica gel using PE/EtOAc (10:1) as the eluent to afford corresponding product.

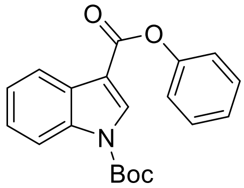

1-(tert-Butyl)-3-phenyl-1H-indole-1,3-dicarboxylate (1e): white solid (1.28 g, 76%); Mp. 182 – 183 °C. ^{1}H NMR (400 MHz, $CDCl_3$) δ 8.46 (s, 1H), 8.23 (dt, *J* = 7.8, 2.0 Hz, 2H), 7.57 – 7.38 (m, 4H), 7.33 – 7.23 (m, 3H), 1.71 (s, 9H); ^{13}C NMR (100 MHz, $CDCl_3$) δ 162.45, 150.57, 148.86, 135.62, 132.99, 129.47, 127.53, 125.82, 125.36, 124.18, 121.86, 121.67, 115.25, 111.52, 85.35, 28.08; HRMS (EI): Calcd for $C_{20}H_{20}NO_4$ $[M+H]^+$ 338.3750, found 338.3751.

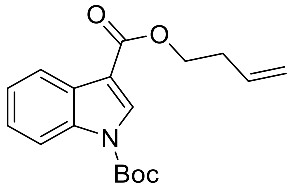

3-(But-3-en-1-yl)-1-(tert-butyl)-1H-indole-1,3-dicarboxylate (1g): colorless oil (1.26 g, 80%). ^{1}H NMR (400 MHz, $CDCl_3$) δ 8.28 (s, 1H), 8.22 – 8.07 (m, 2H), 7.36 (pd, *J* = 7.2, 1.6 Hz, 2H), 5.91 (ddt, *J* = 17.0, 10.3, 6.7 Hz, 1H), 5.28 – 5.04 (m, 2H), 4.42 (t, *J* = 6.7 Hz, 2H), 2.57 (qt, *J* = 6.7, 1.4 Hz, 2H), 1.69 (s, 9H); ^{13}C NMR (100

MHz, $CDCl_3$) δ 164.24, 149.01, 135.58, 134.21, 132.17, 127.53, 125.10, 123.93, 121.76, 117.37, 115.19, 112.46, 85.07, 63.45, 33.33, 28.11; HRMS (EI): Calcd for $C_{18}H_{22}NO_4$ $[M+H]^+$ 316.3690, found 316.3691.

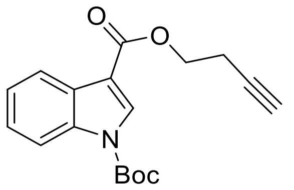

3-(But-3-yn-1-yl)-1-(tert-butyl)-1H-indole-1,3-dicarboxylate (1h): colorless oil (0.94 g, 60%). ^1H NMR (400 MHz, $CDCl_3$) δ 8.30 (s, 1H), 8.18 (d, J = 6.7 Hz, 2H), 7.36 (t, J = 6.5 Hz, 2H), 4.56 – 4.40 (m, 2H), 2.70 (t, J = 6.8 Hz, 2H), 2.06 (s, 1H), 1.69 (s, 9H); ^{13}C NMR (101 MHz, $CDCl_3$) δ 163.76, 148.83, 135.44, 132.25, 127.35, 125.05, 123.88, 121.63, 115.07, 111.92, 85.02, 80.09, 69.99, 61.92, 27.98, 19.12; HRMS (EI): Calcd for $C_{18}H_{20}NO_4$ $[M+H]^+$ 314.3530, found 314.3532.

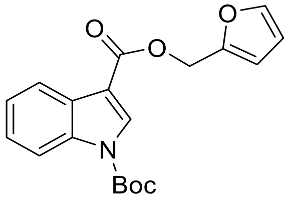

1-(tert-Butyl)-3-(furan-2-ylmethyl)-1H-indole-1,3-dicarboxylate (1w): white solid (1.33g, 78%), Mp. 110 – 111 °C. ^1H NMR (400 MHz, $CDCl_3$) δ 8.29 (s, 1H), 8.16 (ddd, J = 7.3, 5.6, 1.6 Hz, 2H), 7.51 – 7.43 (m, 1H), 7.35 (pd, J = 7.2, 1.5 Hz, 2H), 6.51 (dd, J = 3.3, 0.8 Hz, 1H), 6.40 (dd, J = 3.3, 1.9 Hz, 1H), 5.34 (s, 2H), 1.68 (s, 9H); ^{13}C NMR (100 MHz, $CDCl_3$) δ 163.81, 149.73, 148.96, 143.30, 135.54, 132.41, 127.52, 125.18, 124.03, 121.72, 115.17, 111.87, 110.62, 85.17, 60.43, 57.85, 28.11; HRMS (EI): Calcd for $C_{19}H_{20}NO_5$ $[M+H]^+$ 342.3630, found 342.3633.

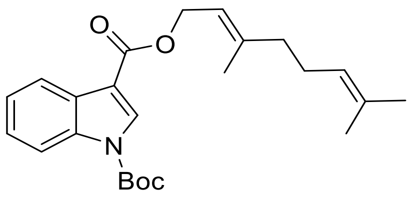

(E)-1-(tert-Butyl)-3-(3,7-dimethylocta-2,6-dien-1-yl)-1H-indole-1,3-dicarboxylate (1x): colorless oil (1.56 g, 80%). ^1H NMR (400 MHz, $CDCl_3$) δ 8.28 (s, 1H), 8.17 (d, J = 7.4 Hz, 2H), 7.41 – 7.30 (m, 2H), 5.51 (ddt, J = 8.5, 7.1, 1.5 Hz, 1H), 5.11 (tq, J = 6.8, 5.6, 1.5 Hz, 1H), 4.88 (d, J = 7.0 Hz, 2H), 2.19 – 2.05 (m, 4H), 1.79 (d, J = 1.4 Hz,

3H), 1.69 (s, 12H), 1.61 (d, J = 1.4 Hz, 3H); ^{13}C NMR (100 MHz, $CDCl_3$) δ 164.32, 149.01, 142.19, 135.56, 132.04, 131.84, 127.58, 125.04, 123.89, 121.74, 118.54, 115.13, 112.53, 85.00, 61.29, 39.55, 28.09, 26.30, 25.67, 17.69, 16.58; HRMS (EI): Calcd for $C_{24}H_{32}NO_4$ $[M+H]^+$ 398.5150, found 398.5154.

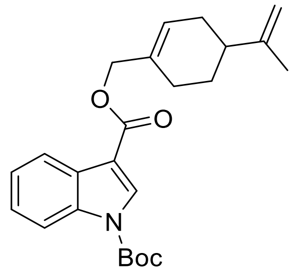

1-(tert-Butyl)-3-((4-(prop-1-en-2-yl)cyclohex-1-en-1-yl)methyl)-1H-indole-1,3-dicarboxylate (1y): colorless oil (1.34 g, 68%). ^{1}H NMR (400 MHz, $CDCl_3$) δ 8.30 (s, 1H), 8.16 (dd, J = 6.6, 3.0 Hz, 2H), 7.36 (tt, J = 7.4, 5.7 Hz, 2H), 5.87 (s, 1H), 4.75 (d, J = 8.9 Hz, 4H), 2.29 – 2.15 (m, 4H), 2.06 – 1.96 (m, 1H), 1.94 – 1.83 (m, 1H), 1.75 (s, 3H), 1.69 (s, 9H), 1.58 (q, J = 3.2, 2.7 Hz, 1H); ^{13}C NMR (100 MHz, $CDCl_3$) δ 164.21, 149.63, 149.06, 135.56, 132.81, 132.17, 127.60, 125.68, 125.11, 123.97, 121.74, 115.20, 112.45, 108.81, 85.13, 68.25, 40.88, 30.51, 28.12, 27.36, 26.56, 20.79; HRMS (EI): Calcd for $C_{24}H_{30}NO_4$ $[M+H]^+$ 396.4990, found 396.4991.

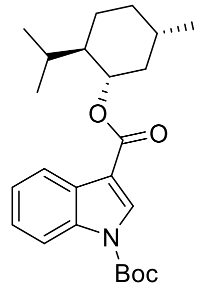

1-(tert-Butyl)-3-((1S,2R,5S)-2-isopropyl-5-methylcyclohexyl)-1H-indole-1,3-dicarboxylate (1z)： colorless oil (1.30 g, 65%). ^{1}H NMR (400 MHz, $CDCl_3$) δ 8.29 (s, 1H), 8.24 – 8.11 (m, 2H), 7.49 – 7.30 (m, 2H), 5.01 (td, J = 10.9, 4.4 Hz, 1H), 2.24 – 2.13 (m, 1H), 2.02 (ddq, J = 13.8, 6.9, 3.7, 2.7 Hz, 1H), 1.80 – 1.72 (m, 2H), 1.70 (s, 9H), 1.59 (tdd, J = 11.7, 5.7, 3.1 Hz, 2H), 1.26 – 1.11 (m, 3H), 0.95 (dd, J = 6.8, 5.1 Hz, 6H), 0.83 (d, J = 6.9 Hz, 3H); ^{13}C NMR (100 MHz, $CDCl_3$) δ 163.87, 149.02, 135.51, 131.90, 127.63, 124.96, 123.82, 121.75, 115.11, 112.87, 84.92, 74.05, 47.28,

41.16, 34.27, 31.42, 28.02, 26.45, 23.59, 22.02, 20.76, 16.47; HRMS (EI): Calcd for $C_{24}H_{34}NO_4$ $[M+H]^+$ 400.5310, found 400.5311.

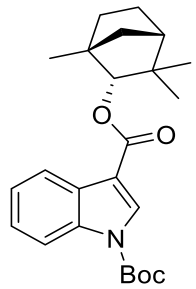

1-(tert-Butyl)-3-((1R,2R,4S)-1,3,3-trimethylbicyclo[2.2.1]heptan-2-yl)-1H-indole-1,3-dicarboxylate (1aa)： colorless oil (1.43 g, 72%). ^{1}H NMR (400 MHz, $CDCl_3$) δ 8.30 (s, 1H), 8.23 – 8.14 (m, 2H), 7.40 – 7.34 (m, 2H), 4.68 (d, J = 1.9 Hz, 1H), 1.98 (dddd, J = 12.3, 8.5, 5.6, 2.2 Hz, 1H), 1.86 – 1.76 (m, 2H), 1.70 (s, 9H), 1.66 (d, J = 17.4 Hz, 2H), 1.29 – 1.24 (m, 2H), 1.21 (s, 3H), 1.15 (s, 3H), 0.87 (s, 3H); ^{13}C NMR (100 MHz, $CDCl_3$) δ 164.81, 149.11, 135.53, 132.09, 127.58, 125.01, 123.91, 121.71, 115.20, 112.84, 86.51, 85.09, 48.49, 48.41, 41.47, 39.74, 28.08, 27.21, 25.94, 20.35, 19.53; HRMS (EI): Calcd for $C_{24}H_{32}NO_4$ $[M+H]^+$ 398.5150, found 398.5151.

4.5 Preparation of 1r–1t

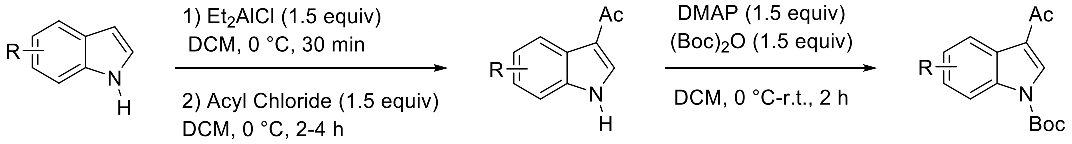

To a solution of indole derivative (5 mmol) in DCM (10 mL) was added Et_2AlCl (2 mol/L in hexane, 4 mL, 7.5 mmol) at 0 °C. The mixture was stirred at 0 °C for 30 min. [4] To this solution was added dropwise a DCM solution (10 mL) of acyl chloride (0.5 mL, 6 mmol) at 0 °C. The resulting solution was stirred at 0 °C until completion of the reaction (monitored by TLC), and buffer solution (pH = 7) was added to quench the reaction. After concentration, the residue was purified by column chromatography on silica gel using PE/EtOAc (8:1) as the eluent to afford the corresponding product.

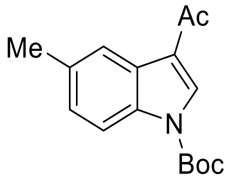

tert-Butyl-3-acetyl-5-methyl-1H-indole-1-carboxylate (1r): white solid (0.75 g, 55%); Mp. 211 – 212 °C. ^{1}H NMR (400 MHz, $CDCl_3$) δ 8.18 (s, 1H), 8.17 (s, 1H), 7.97 (d, J = 8.5 Hz, 1H), 7.18 (dd, J = 8.6, 1.8 Hz, 1H), 2.55 (s, 3H), 2.47 (s, 3H), 1.70 (s, 9H); ^{13}C NMR (100 MHz, $CDCl_3$) δ 194.02, 149.20, 134.09, 133.76, 132.49, 127.53, 126.82, 122.51, 120.42, 114.53, 85.22, 28.13, 27.70, 21.42; HRMS (EI): Calcd for $C_{16}H_{20}NO_3$ $[M+H]^+$ 274.3320, found 274.3321.

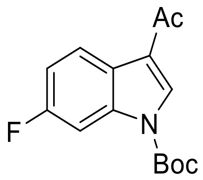

tert-Butyl-3-acetyl-6-fluoro-1H-indole-1-carboxylate (1s): white solid (0.93 g, 67%); Mp. 207 – 208 °C. ^{1}H NMR (400 MHz, $CDCl_3$) δ 8.30 (ddd, J = 8.5, 5.7, 1.8 Hz, 1H), 8.17 (d, J = 1.9 Hz, 1H), 7.81 (dd, J = 10.0, 2.5 Hz, 1H), 7.09 (tt, J = 9.0, 2.0 Hz, 1H), 2.54 (s, 3H), 1.71 (s, 9H); ^{13}C NMR (101 MHz, $CDCl_3$) δ 193.69, 162.48, 160.07, 148.81, 135.74 (d, J = 12.8 Hz), 132.39 (d, J = 3.3 Hz), 123.65 (d, J = 9.7 Hz), 120.39, 112.59 (d, J = 23.7 Hz), 102.30 (d, J = 28.8 Hz), 85.83, 28.04, 27.50; ^{19}F NMR (376 MHz, Chloroform-*d*) δ -115.41 (dd, J = 9.6, 5.6 Hz); HRMS (EI): Calcd for $C_{15}H_{17}FNO_3$ $[M+H]^+$ 278.2954, found 278.2955.

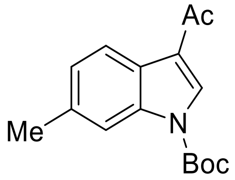

tert-Butyl-3-acetyl-6-methyl-1H-indole-1-carboxylate (1t): white solid (0.63 g, 46%); Mp. 175 – 176 °C. ^{1}H NMR (400 MHz, $CDCl_3$) δ 8.21 (d, J = 8.1 Hz, 1H), 8.13 (d, J = 1.5 Hz, 1H), 7.96 (s, 1H), 7.17 (d, J = 8.1 Hz, 1H), 2.54 (s, 3H), 2.48 (s, 3H), 1.71 (s, 9H); ^{13}C NMR (101 MHz, $CDCl_3$) δ 193.87, 149.15, 135.94, 135.51, 131.75, 125.74, 124.95, 122.13, 120.62, 115.07, 85.13, 28.04, 27.59, 21.93; HRMS (EI): Calcd for $C_{16}H_{20}NO_3$ $[M+H]^+$ 274.3320, found 274.3321.

5. Analytical Data of the Products

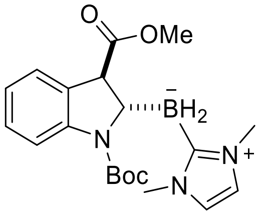

***trans*-(1-(tert-Butoxycarbonyl)-3-(methoxycarbonyl)indolin-2-yl)(1,3-dimethyl-1H-imidazol-3-ium-2-yl)dihydroborate (3a):** white solid (37 mg, 96%); Mp. 163 – 164 °C. ^{1}H NMR (400 MHz, $CDCl_3$) δ 7.70 (brs, 1H), 7.33 (d, *J* = 7.4 Hz, 1H), 7.17 (t, *J* = 7.7 Hz, 1H), 6.93 (t, *J* = 7.4 Hz, 1H), 6.80 (s, 2H), 4.36 (d, *J* = 8.0 Hz, 1H), 3.77 (s, 1H), 3.70 (s, 6H), 3.64 (s, 3H), 1.34 (s, 9H), δ 1.45 – 1.18 (m, 2H); ^{13}C NMR (100 MHz, $CDCl_3$) δ 174.55, 152.03, 142.39, 131.80, 127.90, 125.96, 121.78, 120.11, 115.90, 79.06, 52.60, 51.86, 35.91, 28.35; ^{11}B NMR (128 MHz, $CDCl_3$) δ -26.60 (t, *J* = 86.8 Hz); HRMS (EI): Calcd for $C_{20}H_{28}BN_3O_4Na$ $[M+Na]^+$ 408.2071, found 408.2078.

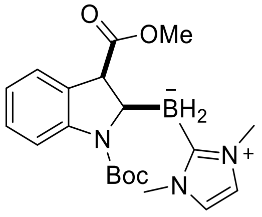

***cis*-(1-(tert-butoxycarbonyl)-3-(methoxycarbonyl)indolin-2-yl)(1,3-dimethyl-1H-imidazol-3-ium-2-yl)dihydroborate (3a'):** white solid (34 mg, 87%); Mp. 163 – 164 °C. ^{1}H NMR (400 MHz, $CDCl_3$) δ 7.45 (brs, 1H), 7.26 (d, *J* = 7.4 Hz, 1H), 7.10 (t, *J* = 7.7 Hz, 1H), 6.86 (t, *J* = 7.4 Hz, 1H), 6.72 (s, 2H), 4.33 – 4.25 (m, 1H), 3.70 (d, *J* = 2.3 Hz, 1H), 3.63 (s, 6H), 3.57 (s, 3H), δ 1.43 – 1.19 (m, 2H), 1.29 (s, 9H); ^{13}C NMR (100 MHz, $CDCl_3$) δ 172.74, 151.91, 142.60, 131.90, 127.19, 126.35, 122.20, 120.14, 115.67, 79.37, 60.33, 51.27, 35.81, 28.28; ^{11}B NMR (128 MHz, $CDCl_3$) δ -29.30 (t, *J* = 86.7 Hz); HRMS (EI): Calcd for $C_{20}H_{28}BN_3O_4Na$ $[M+Na]^+$ 408.2071, found 408.2078.

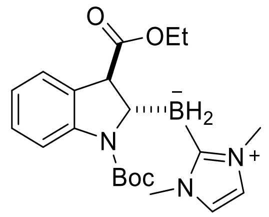

***trans*-(1-(tert-Butoxycarbonyl)-3-(ethoxycarbonyl)indolin-2-yl)(1,3-dimethyl-1H-imidazol-3-ium-2-yl)dihydroborate (3b):** white solid (36 mg, 90%); Mp. 129 – 130 °C. ^{1}H NMR (400 MHz, $CDCl_3$) δ 7.61 (brs, 1H), 7.32 (d, *J* = 7.5 Hz, 1H), 7.15 (t, *J* = 7.8 Hz, 1H), 6.91 (t, *J* = 7.5 Hz, 1H), 6.78 (s, 2H), 4.35 (d, *J* = 6.5 Hz, 1H), 4.16 – 3.98 (m, 2H), 3.72 (s, 1H), 3.68 (s, 6H), 1.90 – 1.23 (m, 2H), 1.31 (s, 9H), 1.19 (t, *J* = 7.2 Hz, 3H); ^{13}C NMR (100 MHz, $CDCl_3$) δ 173.96, 152.16, 142.31, 131.91, 127.74, 125.70, 121.65, 120.09, 115.82, 78.81, 60.27, 52.75, 35.80, 28.25, 14.08; ^{11}B NMR (128 MHz, $CDCl_3$) δ -26.61 (t, *J* = 87.0 Hz); HRMS (EI): Calcd for $C_{21}H_{30}BN_3O_4Na$ $[M+Na]^+$ 422.2227, found 422.2230.

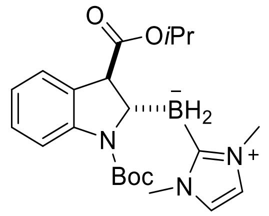

***trans*-(1-(tert-Butoxycarbonyl)-3-(isopropoxycarbonyl)indolin-2-yl)(1,3-dimethyl-1H-imidazol-3-ium-2-yl)dihydroborate (3c):** white solid (37 mg, 90%); Mp. 128 – 129 °C.^{1}H NMR (400 MHz, $CDCl_3$) δ 7.59 (brs, 1H), 7.32 (d, *J* = 7.4 Hz, 1H), 7.14 (t, *J* = 7.8 Hz, 1H), 6.91 (t, *J* = 7.5 Hz, 1H), 6.78 (s, 2H), 4.92 (p, *J* = 6.3 Hz, 1H), 4.35 (d, *J* = 7.9 Hz, 1H), 3.69 (s, 7H), 1.52 – 1.22 (m, 2H), 1.32 (s, 9H), 1.17 (dd, *J* = 14.3, 6.2 Hz, 6H); ^{13}C NMR (100 MHz, $CDCl_3$) δ 173.47, 152.08, 142.36, 132.12, 127.72, 125.65, 121.69, 120.10, 115.86, 78.80, 67.43, 53.02, 35.88, 28.32, 21.74; ^{11}B NMR (128 MHz, $CDCl_3$) δ -26.63 (t, *J* = 86.8 Hz); HRMS (EI): Calcd for $C_{22}H_{32}BN_3O_4Na$ $[M+Na]^+$ 436.2384, found 436.2392.

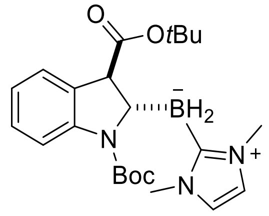

***trans*-(1,3-bis(tert-Butoxycarbonyl)indolin-2-yl)(1,3-dimethyl-1H-imidazol-3-ium-2-yl)dihydroborate (3d):** white solid (33 mg, 80%); Mp. 100 – 101 °C. ^{1}H NMR (400 MHz, CDCl$_3$) δ 7.54 (brs, 1H), 7.25 (d, *J* = 7.4 Hz, 1H), 7.07 (t, *J* = 7.7 Hz, 1H), 6.83 (t, *J* = 7.4 Hz, 1H), 6.72 (s, 2H), 4.25 (d, *J* = 7.9 Hz, 1H), 3.62 (s, 1H), 3.61 (s, *J* = 1.4 Hz, 6H), 1.44 – 1.21 (m, 2H), 1.30 (s, 9H), 1.24 (s, 9H); ^{13}C NMR (100 MHz, CDCl$_3$) δ 173.21, 152.05, 142.42, 132.49, 127.62, 125.65, 121.65, 120.09, 115.81, 79.86, 78.72, 54.03, 35.93, 28.37, 28.01; ^{11}B NMR (128 MHz, CDCl$_3$) δ -26.59 (t, *J* = 86.3 Hz); HRMS (EI): Calcd for $C_{23}H_{34}BN_3O_4Na$ [M+Na]$^+$ 450.2540, found 450.2544.

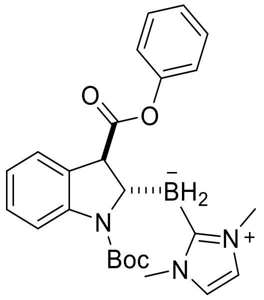

***trans*-(1-(tert-Butoxycarbonyl)-3-(phenoxycarbonyl)indolin-2-yl)(1,3-dimethyl-1H-imidazol-3-ium-2-yl)dihydroborate (3e):** white solid (27 mg, 60%); Mp. 182 – 183 °C. ^{1}H NMR (400 MHz, CDCl$_3$) δ 7.49 (brs, 2H), 7.31 (s, 2H), 7.25 – 7.12 (m, 2H), 7.09 – 6.93 (m, 3H), 6.80 (s, 2H), 4.61 – 4.45 (m, 1H), 4.00 (s, 1H), 3.72 (s, 6H), 2.26 – 1.63 (m, 2H), 1.32 (s, 9H); ^{13}C NMR (100 MHz, CDCl$_3$) δ 172.36, 157.70, 151.14, 138.55, 129.33, 129.19, 128.21, 125.46, 122.46, 121.54, 120.20, 118.89, 115.75, 77.20, 35.92, 28.33, 22.66; ^{11}B NMR (128 MHz, CDCl$_3$) δ -26.59 (t, *J* = 86.7 Hz); HRMS (EI): Calcd for $C_{25}H_{31}N_3O_4B$ [M+H]$^+$ 448.2408, found 448.2409.

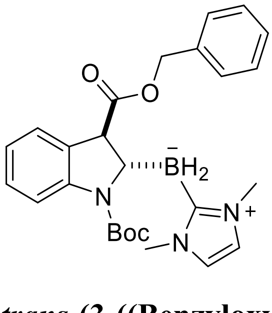

***trans*-(3-((Benzyloxy)carbonyl)-1-(tert-butoxycarbonyl)indolin-2-yl)(1,3-dimethyl-1H-imidazol-3-ium-2-yl)dihydroborate (3f):** white solid (31 mg, 68%); Mp. 103 – 104 °C; ^{1}H NMR (400 MHz, CDCl$_3$) δ 7.69 (s, 1H), 7.35 – 7.20 (m, 6H), 7.15 (t, *J* = 7.7 Hz, 1H), 6.90 (t, *J* = 7.4 Hz, 1H), 6.75 (s, 2H), 5.06 (s, 2H), 4.59 –

4.27 (m, 1H), 3.79 (s, 1H), 3.66 (s, 6H), 1.42 – 1.15 (m, 2H), 1.28 (s, 9H); ^{13}C NMR (100 MHz, CDCl$_3$) δ 173.73, 151.75, 142.09, 136.33, 131.64, 128.33, 127.93, 127.74, 127.54, 125.88, 121.76, 120.11, 115.89, 78.71, 66.02, 52.76, 35.87, 28.27; ^{11}B NMR (128 MHz, CDCl$_3$) δ -26.59 (t, *J* = 86.7 Hz); HRMS (EI): Calcd for $C_{26}H_{32}BN_3O_4Na$ [M+Na]$^+$ 484.2384, found 484.2387.

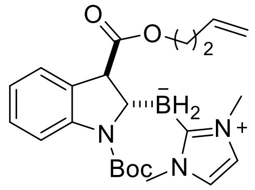

***trans*-(3-((Allyloxy)carbonyl)-1-(tert-butoxycarbonyl)indolin-2-yl)(1,3-dimethyl-1H-imidazol-3-ium-2-yl)dihydroborate (3g):** white solid (36 mg, 85%); Mp. 120 – 121 °C. ^{1}H NMR (400 MHz, CDCl$_3$) δ 7.66 (brs, 1H), 7.31 (d, *J* = 7.4 Hz, 1H), 7.15 (t, *J* = 7.8 Hz, 1H), 6.91 (t, *J* = 7.5 Hz, 1H), 6.78 (s, 2H), 5.73 (ddt, J = 17.1, 10.5, 6.8 Hz, 1H), 5.20 – 4.88 (m, 2H), 4.35 (d, J = 6.6 Hz, 1H), 4.06 (tt, J = 10.8, 5.4 Hz, 2H), 3.73 (s, 1H), 3.68 (s, 6H), 2.32 (q, J = 6.6 Hz, 2H), 1.51– 0.98 (m, 2H), 1.31 (s, 9H); ^{13}C NMR (100 MHz, CDCl$_3$) δ 173.89, 152.16, 142.29, 134.03, 131.78, 127.77, 125.81, 121.63, 120.09, 116.94, 115.81, 78.79, 63.39, 52.74, 35.80, 32.99, 28.25; ^{11}B NMR (128 MHz, CDCl$_3$) δ -26.62 (t, *J* = 86.0 Hz); HRMS (EI): Calcd for $C_{23}H_{32}BN_3O_4Na$ [M+Na]$^+$ 448.2384, found 448.2382.

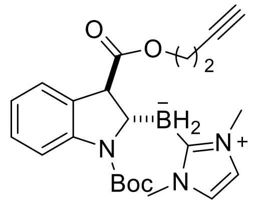

***trans*-(1-(tert-Butoxycarbonyl)-3-((prop-2-yn-1-yloxy)carbonyl)indolin-2-yl)(1,3-dimethyl-1H-imidazol-3-ium-2-yl)dihydroborate (3h):** white solid (38mg, 91%); Mp. 132 – 133 °C. ^{1}H NMR (400 MHz, CDCl$_3$) δ 7.62 (brs, 1H), 7.35 (d, *J* = 7.4 Hz, 1H), 7.16 (t, *J* = 7.8 Hz, 1H), 6.92 (t, *J* = 7.4 Hz, 1H), 6.79 (s, 2H), 4.37 (d, *J* = 5.7 Hz, 1H), 4.29 – 4.03 (m, 2H), 3.77 (s, 1H), 3.69 (s, 6H), 2.47 (td, *J* = 7.0, 2.7 Hz, 2H), 1.96 (s, 1H), 2.22 – 1.44 (m, 2H), 1.32 (s, 9H); ^{13}C NMR (100 MHz, CDCl$_3$) δ 173.66, 151.10, 142.26, 131.55, 127.93, 125.99, 121.78, 120.12, 115.87, 80.11, 79.00, 69.73,

62.05, 52.54, 35.90, 28.31, 18.88; ^{11}B NMR (128 MHz, Chloroform-d) δ -26.61 (t, *J* = 87.1 Hz); HRMS (EI): Calcd for $C_{23}H_{30}N_3O_4BNa$ $[M+Na]^+$ 446.2227, found 446.2230.

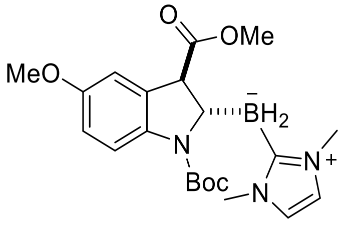

***trans*-(1-(tert-Butoxycarbonyl)-5-methoxy-3-(methoxycarbonyl)indolin-2-yl)(1,3-dimethyl-1H-imidazol-3-ium-2-yl)dihydroborate (3i) :** white solid (29 mg, 70%); Mp. 181 – 182 °C. ^1H NMR (400 MHz, $CDCl_3$) δ 7.64 (brs, 1H), 6.91 (d, *J* = 2.7 Hz, 1H), 6.78 (s, 2H), 6.71 (dd, *J* = 8.8, 2.7 Hz, 1H), 4.33 (d, *J* = 5.2 Hz, 1H), 3.77 (s, 3H), 3.71 (s, 1H), 3.68 (s, 6H), 3.63 (s, 3H), 2.36 – 1.53 (m, 2H), 1.25 (s, 9H); ^{13}C NMR (100 MHz, $CDCl_3$) δ 174.37, 154.96, 135.68, 133.06, 125.46, 120.08, 116.27, 112.97, 111.94, 79.35, 55.58, 52.78, 51.88, 35.88, 28.34; ^{11}B NMR (128 MHz, $CDCl_3$) δ -26.61 (t, *J* = 86.8 Hz); HRMS (EI): Calcd for $C_{21}H_{30}BN_3O_5$ Na $[M+Na]^+$ 438.2176, found 438.2177.

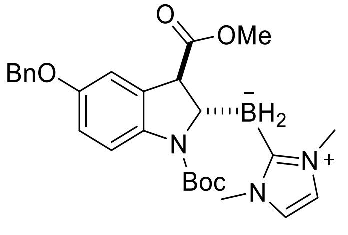

***trans*-(-5-(Benzyloxy)-1-(tert-butoxycarbonyl)-3-(methoxycarbonyl)indolin-2-yl)(1,3-dimethyl-1H-imidazol-3-ium-2-yl)dihydroborate (3j):** white solid (24 mg, 50%); Mp. 124 – 125 °C.^1H NMR (400 MHz, $CDCl_3$) δ 7.50 (brs, 1H), 7.46 (d, *J* = 7.1 Hz, 2H), 7.40 (t, *J* = 7.4 Hz, 2H), 7.34 (d, *J* = 7.2 Hz, 1H), 7.03 (d, *J* = 2.7 Hz, 1H), 6.81 (s, 3H), 5.05 (s, 2H), δ 4.37 (d, *J* = 7.6 Hz, 1H), 3.72 (s, 7H), 3.65 (s, 3H), 1.44 – 1.22 (m, 2H), 1.30 (s, 9H); ^{13}C NMR (100 MHz, $CDCl_3$) δ 174.31, 154.22, 152.34, 137.39, 136.30, 133.02, 128.42, 127.72, 127.48, 120.09, 116.27, 114.09, 113.00, 78.61, 70.45, 52.80, 51.83, 35.86, 28.34; ^{11}B NMR (128 MHz, $CDCl_3$) δ -26.58 (t, *J* = 86.4 Hz); HRMS (EI): Calcd for $C_{27}H_{34}BN_3O_5Na$ $[M+Na]^+$ 514.2489, found 514.2489.

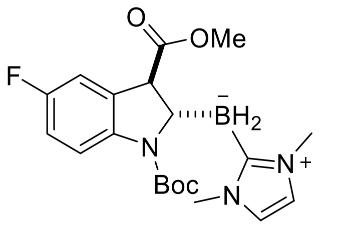

***trans*-(1-(tert-Butoxycarbonyl)-5-fluoro-3-(methoxycarbonyl)indolin-2-yl)(1,3-dimethyl-1H-imidazol-3-ium-2-yl)dihydroborate (3k):** white solid (23 mg, 58%); Mp. 178 – 179 °C. ^{1}H NMR (400 MHz, $CDCl_3$) δ 7.64 (s, 1H), 7.03 (dd, *J* = 8.3, 2.7 Hz, 1H), 6.92 – 6.72 (m, 3H), 4.36 (d, *J* = 6.2 Hz, 1H), 3.71 (s, 1H), 3.68 (s, 6H), 3.65 (s, 3H), 1.56 – 1.10 (m, 2H), 1.31 (s, 9H); ^{13}C NMR (100 MHz, $CDCl_3$) δ 173.95, 158.29 (d, *J* = 239.2 Hz), 151.51, 138.89, 133.42 (d, *J* = 8.3 Hz), 120.17, 116.27, 114.10 (d, *J* = 22.7 Hz), 113.21 (d, *J* = 23.1 Hz), 78.97, 52.66, 51.96, 35.87, 28.28; ^{11}B NMR (128 MHz, $CDCl_3$) δ -26.61 (t, *J* = 86.7 Hz); ^{19}F NMR (376 MHz, $CDCl_3$) δ -122.43 (d, *J* = 284.7 Hz); ^{19}F NMR (376 MHz, $CDCl_3$)) δ -122.43 (d, *J* = 284.7 Hz); HRMS (EI): Calcd for $C_{20}H_{27}BN_3O_4FNa$ $[M+Na]^+$ 426.1976, found 426.1982.

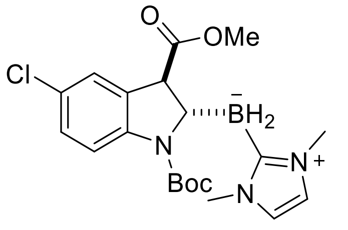

***trans*-(1-(tert-Butoxycarbonyl)-5-chloro-3-(methoxycarbonyl)indolin-2-yl)(1,3-dimethyl-1H-imidazol-3-ium-2-yl)dihydroborate (3l):** white solid (27 mg, 65%); Mp. 158 – 159 °C. ^{1}H NMR (400 MHz, $CDCl_3$) δ 7.60 (brs, 1H), 7.27 (d, *J* = 2.2 Hz, 1H), 7.11 (dd, *J* = 8.4, 2.4 Hz, 1H), 6.80 (s, 2H), 4.35 (d, *J* = 6.1 Hz, 1H), 3.71 (s, 1H), 3.68 (s, 6H), 3.65 (s, 3H), 1.52 – 1.12 (m, 2H), 1.31 (s, 9H); ^{13}C NMR (100 MHz, $CDCl_3$) δ 173.90, 151.86, 141.11, 138.78, 133.53, 127.82, 126.09, 120.18, 116.64, 79.25, 52.39, 52.00, 35.87, 28.25; ^{11}B NMR (128 MHz, $CDCl_3$) δ -26.24 (t, *J* = 88.0 Hz); HRMS (EI): Calcd for $C_{20}H_{27}N_3O_4NaCl$ $[M+Na]^+$ 442.1681, found 442.1684.

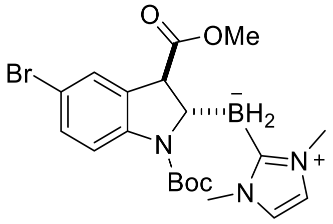

***trans*-(5-Bromo-1-(tert-butoxycarbonyl)-3-(methoxycarbonyl)indolin-2-yl)(1,3-dimethyl-1H-imidazol-3-ium-2-yl)dihydroborate (3m):** white solid (15 mg, 33%); Mp. 174 – 175 °C. ^{1}H NMR (400 MHz, $CDCl_3$) δ 7.62 – 7.15 (m, 2H), 6.90 (s, 2H), 6.71 (td, J = 8.6, 2.5 Hz, 1H), 4.48 (d, J = 6.5 Hz, 1H), 3.80 (s, 7H), 3.75 (s, 3H), 1.40 – 1.21 (m, 2H), 1.43 (s, 9H); ^{13}C NMR (100 MHz, $CDCl_3$) δ 173.85, 152.00, 142.37, 133.94, 130.70, 128.89, 125.87, 120.17, 113.81, 79.13, 52.57, 51.79, 35.82, 28.28; ^{11}B NMR (128 MHz, $CDCl_3$) δ -26.57 (t, J = 86.8 Hz); HRMS (EI): Calcd for $C_{20}H_{27}BN_3O_4NaBr$ $[M+Na]^+$ 486.1176, found 486.1184.

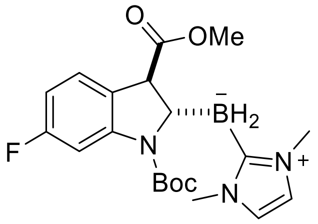

***trans*-(1-(tert-Butoxycarbonyl)-6-fluoro-3-(methoxycarbonyl)indolin-2-yl)(1,3-dimethyl-1H-imidazol-3-ium-2-yl)dihydroborate (3n):** white solid (25 mg, 63%); Mp. 127 – 128 °C. ^{1}H NMR (400 MHz, $CDCl_3$) δ 7.52 (brs, 1H), 7.22 (dd, J = 8.2, 5.9 Hz, 1H), 6.80 (s, 2H), 6.61 (td, J = 8.6, 2.4 Hz, 1H), 4.37 (d, J = 5.8 Hz, 1H), 3.70 (s, 7H), 3.64 (s, 3H), 1.47-1.17 (m, 2H),1.34 (s, 9H); ^{13}C NMR (100 MHz, $CDCl_3$) δ 174.46, 163.05 (d, J = 241.0 Hz), 152.28, 142.83, 126.80 (d, J = 76.4 Hz), 120.18, 112.66, 108.09 (d, J = 20.9 Hz), 103.85 (d, J = 29.3 Hz), 79.70, 52.66, 51.94, 35.92, 28.28; ^{11}B NMR (128 MHz, $CDCl_3$) δ -26.57 (t, J = 86.8 Hz); ^{19}F NMR (376 MHz, $CDCl_3$) δ -114.46; HRMS (EI): Calcd for $C_{20}H_{28}BFN_3O_4$ $[M+H]^+$ 404.2614, found 404.2616.

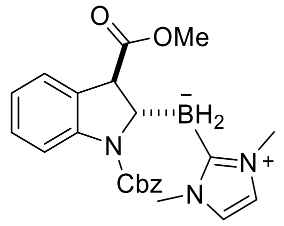

***trans*-(1-((Benzyloxy)carbonyl)-3-(methoxycarbonyl)indolin-2-yl)(1,3-dimethyl-1H-imidazol-3-ium-2-yl)dihydroborate (3o):** white solid (13 mg, 33%); Mp. 119 – 120 °C; ^{1}H NMR (400 MHz, $CDCl_3$) δ 7.83 (brs, 1H), 7.45 – 7.28 (m, 6H), 7.21 (s, 1H), 6.97 (t, J = 7.5 Hz, 1H), 6.54 (s, 2H), 5.24 – 4.93 (m, 1H), 4.46 (s, 2H), 3.82 (s, 1H), 3.66 (s, 3H), 3.46 (s, 6H), 1.43 – 1.24 (m, 2H); ^{13}C NMR (101 MHz, $CDCl_3$) δ 174.45, 152.75, 142.03, 136.53, 131.58, 128.32, 128.09, 127.99, 125.96, 122.68, 122.34, 119.96, 115.81, 66.77, 58.34, 51.95, 35.65; ^{11}B NMR (128 MHz, $CDCl_3$) δ -26.70 (t, J = 87.3 Hz); HRMS (EI): Calcd for $C_{23}H_{26}BN_3O_4Na$ $[M+Na]^+$ 442.1914, found 442.1914.

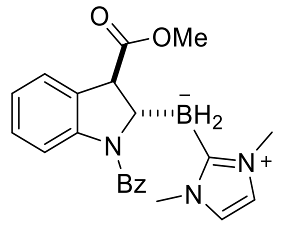

***trans*-(1-Benzoyl-3-(methoxycarbonyl)indolin-2-yl)(1,3-dimethyl-1H-imidazol-3-ium-2-yl)dihydroborate (3p):** white solid (18 mg, 47%); Mp. 135 – 136 °C. ^{1}H NMR (400 MHz, $CDCl_3$) δ 8.37 (brs, 1H), 7.43 (d, J = 7.2 Hz, 1H), 7.39 – 7.26 (m, 5H), 7.19 – 6.93 (m, 2H), 6.70 (s, 2H), 4.89 (s, 1H), 3.81 (s, 1H), 3.73 (s, 3H), 3.27 (s, 6H), 1.48 – 1.25 (m, 2H); ^{13}C NMR (100 MHz, $CDCl_3$) δ 174.48, 167.59, 143.04, 137.49, 129.44, 128.99, 128.18, 127.44, 125.95, 123.59, 122.44, 120.56, 117.79, 52.69, 52.01, 35.47; ^{11}B NMR (128 MHz, $CDCl_3$) δ -26.77 (t, J = 87.4 Hz); HRMS (EI): Calcd for $C_{22}H_{24}BN_3O_3Na$ $[M+Na]^+$ 412.1808, found 412.1814.

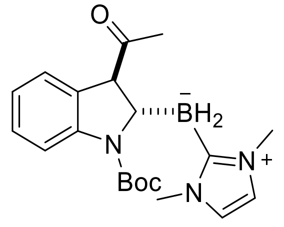

***trans*-(3-Acetyl-1-(tert-butoxycarbonyl)indolin-2-yl)(1,3-dimethyl-1H-imidazol-3-ium-2-yl)dihydroborate (3q):** white solid (14 mg, 40%); Mp. 100 – 101 °C; ^{1}H NMR (400 MHz, $CDCl_3$) δ 7.76 (s, 1H), 7.18 (t, *J* = 6.2 Hz, 2H), 6.93 (t, *J* = 7.4 Hz, 1H), 6.79 (s, 2H), 4.19 (s, 1H), 3.68 (s, 7H), 2.03 (s, 3H), 1.51 – 1.17 (m, 2H),1.33 (s, 9H); ^{13}C NMR (100 MHz, $CDCl_3$) δ 208.73, 151.98, 142.68, 132.15, 127.96, 125.61, 122.09, 120.17, 116.14, 79.66, 61.78, 58.50, 35.87, 28.30; ^{11}B NMR (128 MHz, $CDCl_3$) δ -26.57 (t, *J* = 87.5 Hz); HRMS (EI): Calcd for $C_{20}H_{28}BN_3O_3Na$ $[M+Na]^+$ 392.2121, found 392.2127.

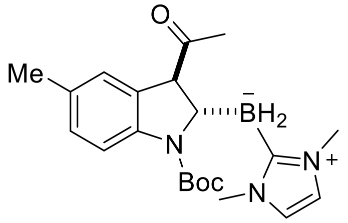

***trans*-(3-Acetyl-1-(tert-butoxycarbonyl)-5-methylindolin-2-yl)(1,3-dimethyl-1H-imidazol-3-ium-2-yl)dihydroborate (3r):** white solid (30 mg, 80%); Mp. 162 – 163 °C; ^{1}H NMR (400 MHz, $CDCl_3$) δ 7.63 (brs, 1H), 6.98 (d, *J* = 15.6 Hz, 2H), 6.79 (s, 2H), 4.16 (s, 1H), 3.69 (s, 6H), 3.65 (s,1H), 2.28 (s, 3H), 2.03 (s, 3H), 1.44 – 1.19 (m, 2H), 1.32 (s, 9H); ^{13}C NMR (100 MHz, $CDCl_3$) δ 208.97, 151.82, 139.57, 132.24, 131.56, 128.48, 126.31, 120.14, 115.88, 79.43, 62.07, 35.88, 28.33, 20.88, 14.14; ^{11}B NMR (128 MHz, $CDCl_3$) δ -26.59 (t, *J* = 87.1 Hz); HRMS (EI): Calcd for $C_{21}H_{30}BN_3O_3Na$ $[M+Na]^+$ 406.2278, found 406.2279.

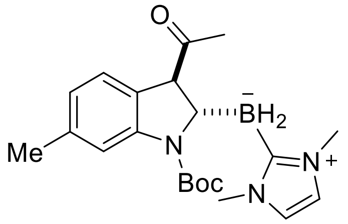

***trans*-(3-Acetyl-1-(tert-butoxycarbonyl)-6-methylindolin-2-yl)(1,3-dimethyl-1H-imidazol-3-ium-2-yl)dihydroborate (3t):** white solid (31 mg, 83%); Mp. 194 – 195 °C. ^{1}H NMR (400 MHz, $CDCl_3$) δ 7.63 (brs, 1H), 7.08 (d, *J* = 7.4 Hz, 1H), 6.81 (s, 2H), 6.76 (d, *J* = 7.4 Hz, 1H), 4.17 (d, *J* = 7.0 Hz, 1H), 3.70 (s, 6H), 3.65 (s, 1H), 2.32 (s, 3H), 2.02 (s, 3H), 1.45-1.17 (m, 2H), 1.29 (s, 9H); ^{13}C NMR (100 MHz, $CDCl_3$) δ 209.04, 152.86, 142.66, 137.73, 129.30, 125.12, 123.02, 120.19, 117.08, 78.54, 61.65,

35.94, 28.27, 21.80, 14.09; ^{11}B NMR (128 MHz, $CDCl_3$) δ -26.56 (t, *J* = 87.5 Hz); HRMS (EI): Calcd for $C_{21}H_{30}BN_3O_3Na$ $[M+Na]^+$ 406.2278, found 406.2280.

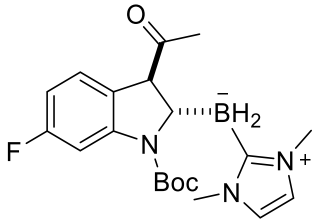

***trans*-(3-Acetyl-1-(tert-butoxycarbonyl)-6-fluoroindolin-2-yl)(1,3-dimethyl-1H-imidazol-3-ium-2-yl)dihydroborate (3s):** white solid (20 mg, 52%); Mp. 130 – 131 °C. ^{1}H NMR (400 MHz, $CDCl_3$) δ 7.53 (s, 1H), 7.10 (dd, J = 8.2, 5.8 Hz, 1H), 6.81 (s, 2H), 6.62 (td, *J* = 8.6, 2.4 Hz, 1H), 4.21 (d, *J* = 6.9 Hz, 1H), 3.70 (s, 6H), 3.67 (s, 1H), 2.06 (s, 3H), 1.48 - 1.21 (m, 2H), 1.34 (s, 9H); ^{13}C NMR (100 MHz, $CDCl_3$) δ 208.55, 163.04 (d, *J* = 241.4 Hz), 151.77, 143.69, 133.01 (d, *J* = 205.5 Hz), 127.54, 120.25, 113.03 (d, *J* = 369.3 Hz), 106.32 (d, *J* = 437.9 Hz), 79.27, 60.79, 35.91, 28.27, 26.20; ^{11}B NMR (128 MHz, $CDCl_3$) δ -26.51 (t, *J* = 87.6 Hz); ^{19}F NMR (376 MHz, $CDCl_3$) δ -114.15 (d, *J* = 117.7 Hz); HRMS (EI): Calcd for $C_{20}H_{27}BN_3O_3FNa$ $[M+Na]^+$ 410.2027, found 410.2028.

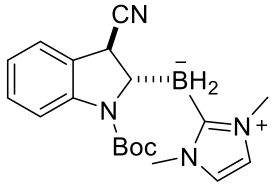

***trans*-(1-(tert-Butoxycarbonyl)-3-cyanoindolin-2-yl)(1,3-dimethyl-1H-imidazol-3-ium-2-yl)dihydroborate (3u):** white solid (14 mg, 42%); Mp. 101 – 102 °C. ^{1}H NMR (400 MHz, $CDCl_3$) δ 7.57 (s, 1H), 7.32 (d, *J* = 7.5 Hz, 1H), 7.22 (t, *J* = 7.8 Hz, 1H), 6.98 (t, *J* = 7.5 Hz, 1H), 6.81 (s, 2H), 4.28 (d, *J* = 4.5 Hz, 1H), 3.85 (s, 1H), 3.68 (s, 6H), 1.47 – 1.13 (m, 2H), 1.33 (s, 9H); ^{13}C NMR (100 MHz, $CDCl_3$) δ 151.43, 141.73, 128.98, 128.23, 125.64, 122.46, 121.99, 120.32, 116.16, 79.72, 60.36, 35.91, 28.26; ^{11}B NMR (128 MHz, $CDCl_3$) δ -26.81 (t, *J* = 87.4 Hz); HRMS (EI): Calcd for $C_{19}H_{25}BN_4O_2Na$ $[M+Na]^+$ 375.1968, found 375.1962.

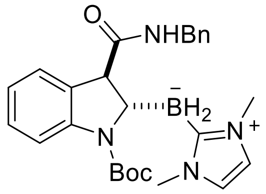

***trans*-(3-(Benzylcarbamoyl)-1-(tert-butoxycarbonyl)indolin-2-yl)(1,3-dimethyl-1H-imidazol-3-ium-2-yl)dihydroborate (3v):** colorless oil (0.55 g, 60%). ^{1}H NMR (400 MHz, $CDCl_3$) δ 7.56 (brs, 1H), 7.38 (d, J = 7.2 Hz, 2H), 7.32 (t, J = 7.3 Hz, 2H), 7.28 – 7.21 (m, 2H), 7.16 (t, J = 7.8 Hz, 1H), 6.94 (t, J = 7.5 Hz, 1H), 6.82 (s, 2H), 6.40 (dd, J = 7.4, 4.0 Hz, 1H), 4.84 (dd, J = 14.8, 7.1 Hz, 1H), 4.55 (d, J = 10.1 Hz, 1H), 4.49 (q, J = 4.8 Hz, 1H), 4.28 (dd, J = 14.8, 4.0 Hz, 1H), 3.67 (s, 6H), 1.27 (m, 11H); ^{13}C NMR (100 MHz, $CDCl_3$) δ 173.08, 151.75, 143.06, 138.47, 133.10, 128.45, 127.98, 127.60, 127.10, 125.39, 122.26, 120.27, 116.22, 79.14, 54.45, 43.85, 35.84, 28.22; ^{11}B NMR (128 MHz, $CDCl_3$) δ -28.82 (t, J = 86.2 Hz); HRMS (EI): Calcd for $C_{26}H_{34}BN_4O_3$ $[M+H]^+$ 461.3930, found 461.3931.

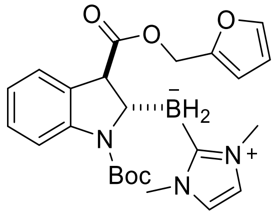

***trans*-(1-(tert-Butoxycarbonyl)-3-((furan-2-ylmethoxy)carbonyl)indolin-2-yl)(1,3-dimethyl-1H-imidazol-3-ium-2-yl)dihydroborate (3w):** white solid (33 mg, 82%); Mp. 120 – 121 °C. ^{1}H NMR (400 MHz, $CDCl_3$) δ 7.63 (s, 1H), 7.38 (t, J = 1.4 Hz, 1H), 7.30 (d, J = 7.5 Hz, 1H), 7.15 (t, J = 7.8 Hz, 1H), 6.90 (t, J = 7.5 Hz, 1H), 6.78 (s, 2H), 6.32 (d, J = 1.5 Hz, 2H), 5.02 (d, J = 1.7 Hz, 2H), 4.35 (d, J = 5.7 Hz, 1H), 3.77 (s, 1H), 3.67 (s, 6H), 1.44– 1.15 (m, 2H), 1.31 (s, 9H); ^{13}C NMR (100 MHz, $CDCl_3$) δ 173.53, 149.81, 142.84, 138.69, 131.47, 127.92, 125.87, 122.94, 121.76, 120.11, 115.87, 110.41, 110.00, 78.92, 58.18, 52.55, 35.87, 28.28; ^{11}B NMR (128 MHz, $CDCl_3$) δ -26.61 (t, J = 87.1 Hz); HRMS (EI): Calcd for $C_{24}H_{30}BN_3O_5Na$ $[M+Na]^+$ 451.3300, found 451.3301.

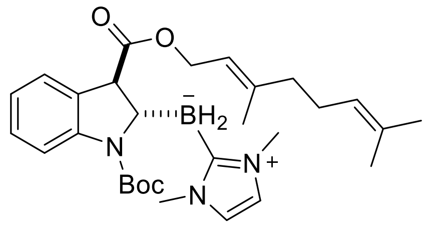

***trans*-(1-(tert-Butoxycarbonyl)-3-((((E)-3,7-dimethylocta-2,6-dien-1-yl)oxy)carbonyl)indolin-2-yl)(1,3-dimethyl-1H-imidazol-3-ium-2-yl)dihydroborate (3x):** white solid (31 mg, 63%); Mp. 101 – 102 °C. ^{1}H NMR (400 MHz, $CDCl_3$) δ 7.60 (brs, 1H), 7.26 (d, *J* = 7.4 Hz, 1H), 7.13 – 7.03 (m, 1H), 6.84 (d, *J* = 7.4, 1H), 6.72 (s, 2H), 5.24 (m, 1H), 5.08 – 4.96 (m, 1H), 4.48 (d, *J* = 6.9 Hz, 2H), 4.30 (d, *J* = 6.7 Hz, 1H), 3.68 (s, 1H), 3.62 (s, 6H), 2.17 – 1.85 (m, 4H), 1.63 – 1.51 (m, 9H), 1.36 – 1.15 (m, 2H), 1.25 (s, 9H); ^{13}C NMR (100 MHz, $CDCl_3$) δ 173.97, 152.26, 141.50, 131.89, 131.63, 127.77, 125.82, 123.77, 121.68, 120.08, 119.84, 118.61, 115.83, 78.95, 61.40, 39.42, 35.85, 28.29, 26.26, 25.62, 17.62, 16.41; ^{11}B NMR (128 MHz, $CDCl_3$) δ -26.60 (t, *J* = 86.3 Hz); HRMS (EI): Calcd for $C_{29}H_{43}BN_3O_4$ $[M+H]^+$ 508.3347, found 508.3342.

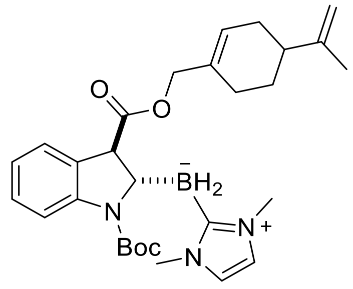

***trans*-(1-(tert-Butoxycarbonyl)-3-(((4-(prop-1-en-2-yl)cyclohex-1-en-1-yl)methoxy)carbonyl)indolin-2-yl)(1,3-dimethyl-1H-imidazol-3-ium-2-yl)dihydroborate (3y):** white solid (29 mg, 58%); Mp. 137 – 138 °C. ^{1}H NMR (400 MHz, $CDCl_3$) δ 7.52 (s, 1H), 7.33 (d, *J* = 7.4 Hz, 1H), 7.16 (t, *J* = 7.7 Hz, 1H), 6.92 (t, *J* = 7.4 Hz, 1H), 6.78 (s, 2H), 5.77 – 5.60 (m, 1H), 4.70 (d, *J* = 8.1 Hz, 2H), 4.54 – 4.32 (m, 3H), 3.77 (s, 1H), 3.70 (s, 6H), 2.22 – 2.06 (m, 2H), 2.04 – 1.75 (m, 5H), 1.72 (s, 3H), 1.40 – 1.19 (m, 2H), 1.33 (s, 9H); ^{13}C NMR (100 MHz, $CDCl_3$) δ 173.85, 151.85, 149.74, 142.32, 132.77, 131.89, 127.86, 125.87, 125.21, 121.72, 120.11, 115.88, 108.62, 78.56, 68.25, 52.91, 40.83, 35.92, 30.39, 28.34, 27.29, 26.18, 20.72,; ^{11}B NMR (128

MHz, $CDCl_3$) δ -26.63 (t, *J* = 86.5 Hz); HRMS (EI): Calcd for $C_{29}H_{41}BN_3O_4$ $[M+H]^+$ 506.3190, found 506.3183.

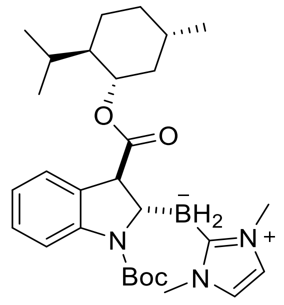

***trans*-(1-(tert-Butoxycarbonyl)-3-((((1S,2R,5S)-2-isopropyl-5-methylcyclohexyl)oxy)carbonyl)indolin-2-yl)(1,3-dimethyl-1H-imidazol-3-ium-2-yl)dihydroborate (3z):** white solid (27 mg, 55%); Mp. 129 – 130 °C; ^{1}H NMR (400 MHz, $CDCl_3$) δ 7.76 (brs, 1H), 7.27 (t, *J* = 7.0 Hz, 1H), 7.13 (t, *J* = 7.5 Hz, 1H), 6.89 (t, *J* = 7.4 Hz, 1H), 6.78 (s, 2H), 4.34 (d, *J* = 5.3 Hz, 1H), 4.26 (dd, *J* = 9.4, 1.9 Hz, 1H), 3.74 (s, 1H), 3.69 (s, 6H), 1.81 (s, 1H), 1.59 (tdd, J = 15.1, 8.0, 3.5 Hz, 3H), 1.53 – 1.44 (m, 2H), 1.41 – 1.21 (m, 11H), 1.12 – 0.92 (m, 7H), 0.86 (dtt, *J* = 8.3, 4.4, 2.2 Hz, 1H), 0.73 (d, *J* = 29.5 Hz, 3H), 0.45 (s, 1H); ^{13}C NMR (100 MHz, $CDCl_3$) δ 174.48, 152.10, 142.56, 132.35, 127.68, 125.70, 121.58, 120.14, 115.72, 85.59, 77.20, 48.29, 41.19, 39.46, 35.88, 29.56, 28.33, 26.35, 25.69, 20.17, 19.43, 18.98; ^{11}B NMR (128 MHz, $CDCl_3$) δ -26.60 (t, *J* = 86.5 Hz); HRMS (EI): Calcd for $C_{29}H_{45}BN_3O_4$ $[M+H]^+$ 510.5060, found 510.5061.

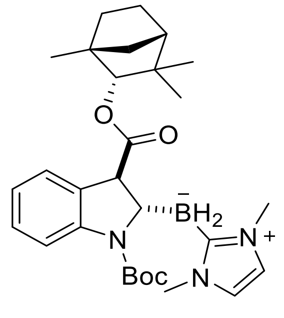

***trans*-(1-(tert-Butoxycarbonyl)-3-((((1R,2R,4S)-1,3,3-trimethylbicyclo[2.2.1]heptan-2-yl)oxy)carbonyl)indolin-2-yl)(1,3-dimethyl-1H-imidazol-3-ium-2-yl)dihydroborate (3aa):** white solid (18 mg, 37%); Mp. 116 – 117 °C. ^{1}H NMR (400 MHz, $CDCl_3$) δ 7.62 (brs, 1H), 7.27 (d, *J* = 7.8 Hz, 1H), 7.12 (t, *J* = 7.7 Hz, 1H), 6.88 (t, *J* = 7.4 Hz, 1H), 6.77 (s, 2H), 4.55 (qd, *J* = 11.0, 4.3 Hz, 1H),

4.37 – 4.25 (m, 1H), 3.69 (s, 1H), 3.67 (s, 6H), 1.98 – 1.86 (m, 1H), 1.60 (ddt, *J* = 13.7, 10.3, 3.4 Hz, 3H), 1.30 (s, 12H), 0.96 – 0.81 (m, 6H), 0.76 (d, *J* = 7.0 Hz, 2H), 0.60 (dd, *J* = 13.5, 6.9 Hz, 3H); ^{13}C NMR (100 MHz, $CDCl_3$) δ 173.58, 151.99, 142.35, 131.97, 127.61, 125.43, 121.59, 120.07, 115.77, 78.61, 73.97, 47.04, 40.58, 35.77, 34.19, 31.21, 28.25, 25.83, 23.33, 21.89, 20.59, 16.15; ^{11}B NMR (128 MHz, $CDCl_3$) δ -26.64 (t, *J* = 87.0 Hz); HRMS (EI): Calcd for $C_{29}H_{42}BN_3O_4Na$ $[M+Na]^+$ 530.4718, found 530.4719.

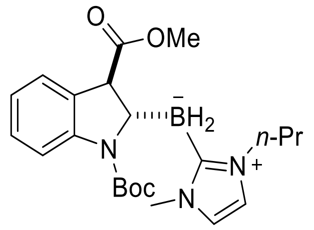

***trans*-(1-(tert-Butoxycarbonyl)-3-(methoxycarbonyl)indolin-2-yl)(1-methyl-3-propyl-1H-imidazol-3-ium-2-yl)dihydroborate (3ab):** white solid (11 mg, 27%); Mp. 176 – 177 °C. ^{1}H NMR (400 MHz, $CDCl_3$) δ 7.52 (brs, 1H), 7.32 (d, *J* = 7.4 Hz, 1H), 7.16 (t, *J* = 7.7 Hz, 1H), 6.92 (t, *J* = 7.4 Hz, 1H), 6.82 (d, *J* = 10.8 Hz, 2H), 4.35 (d, *J* = 6.3 Hz, 1H), 4.12 – 3.88 (m, 2H), 3.74 (s, 1H), 3.72 (s, 3H), 3.63 (s, 3H), 1.75 (q, *J* = 7.5 Hz, 2H), 1.33 (s, 9H), 1.40– 1.24 (m, 2H), 0.89 (t, *J* = 7.4 Hz, 3H); ^{13}C NMR (100 MHz, $CDCl_3$) δ 174.59, 152.46, 142.37, 131.84, 127.85, 125.92, 121.75, 120.22, 118.58, 116.00, 79.26, 52.71, 51.85, 50.05, 35.83, 28.37, 23.52, 11.00; ^{11}B NMR (128 MHz, $CDCl_3$) δ -26.57 (t, *J* = 86.8 Hz); HRMS (EI): Calcd for $C_{22}H_{33}BN_3O_4$ $[M+H]^+$ 414.2564, found 414.2573.

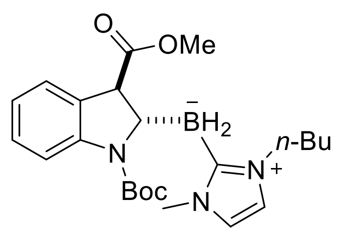

***trans*-(1-(tert-Butoxycarbonyl)-3-(methoxycarbonyl)indolin-2-yl)(3-butyl-1-methyl-1H-imidazol-3-ium-2-yl)dihydroborate (3ac):** white solid (28 mg, 67%); Mp. 99 – 100 °C. ^{1}H NMR (400 MHz, $CDCl_3$) δ 7.52 (brs, 1H), 7.31 (d, *J* = 7.4 Hz, 1H), 7.15 (t, *J* = 7.6 Hz, 1H), 6.91 (t, *J* = 7.4 Hz, 1H), 6.81 (d, *J* = 9.1 Hz, 2H), 4.35 (d, *J* = 5.8 Hz, 1H), 4.14 – 3.90 (m, 2H), 3.73 (s, 1H), 3.72 (s, 3H), 3.63 (s, 3H), 1.73 – 1.64 (m, 2H), 1.33 (s, 9H), 1.41 – 1.28 (m, 2H), 1.40– 1.24 (m, 2H), 0.88 (t, *J* = 7.4 Hz, 3H);

^{13}C NMR (100 MHz, $CDCl_3$) δ 174.57, 152.24, 142.26, 131.81, 127.81, 125.89, 121.71, 120.23, 118.52, 115.97, 78.95, 52.69, 51.83, 48.27, 35.80, 32.28, 28.35, 19.72, 13.53; ^{11}B NMR (128 MHz, $CDCl_3$) δ -26.56 (t, J = 86.4 Hz); HRMS (EI): Calcd for $C_{23}H_{35}BN_3O_4$ $[M+H]^+$ 428.2721, found 428.2722.

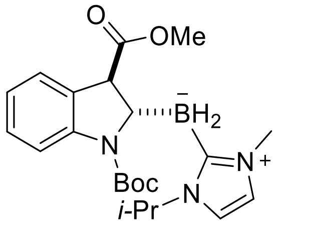

***trans*-(1-(tert-Butoxycarbonyl)-3-(methoxycarbonyl)indolin-2-yl)(3-isopropyl-1-methyl-1H-imidazol-3-ium-2-yl)dihydroborate (3ad):** white solid (36 mg, 89%); Mp. 162 – 163 °C. ^{1}H NMR (400 MHz, $CDCl_3$) δ 7.54 (brs, 1H), 7.31 (d, J = 7.4 Hz, 1H), 7.16 (t, J = 7.6 Hz, 1H), 6.96 – 6.88 (m, 2H), 6.82 (s, 1H), 4.92 (p, J = 6.8 Hz, 1H), 4.36 (s, 1H), 3.75 (s, 3H), 3.72 (s, 1H), 3.62 (s, 3H), 1.37 (d, J = 6.8 Hz, 3H), 1.36 – 1.22 (m, 2H), 1.31 (d, J = 9.0 Hz, 12H); ^{13}C NMR (100 MHz, $CDCl_3$) δ 174.53, 152.17, 142.47, 131.85, 127.80, 125.85, 121.70, 120.57, 116.02, 114.76, 78.97, 52.73, 51.78, 49.61, 35.65, 28.33, 23.27; ^{11}B NMR (128 MHz, $CDCl_3$) δ -26.57 (t, J = 86.4 Hz); HRMS (EI): Calcd for $C_{22}H_{33}BN_3O_4$ $[M+H]^+$ 414.3250, found 414.3252.

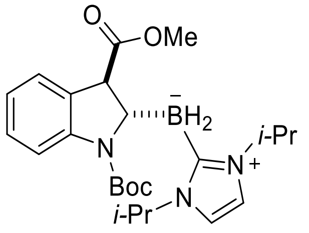

***trans*-(1-(tert-Butoxycarbonyl)-3-(methoxycarbonyl)indolin-2-yl)(1,3-diisopropyl-1H-imidazol-3-ium-2-yl)dihydroborate (3ae):** white solid (40 mg, 91%); Mp. 181 – 182 °C. ^{1}H NMR (400 MHz, $CDCl_3$) δ 7.49 (brs, 1H), 7.28 (d, J = 7.4 Hz, 1H), 7.15 (t, J = 7.5 Hz, 1H), 7.00 – 6.84 (m, 3H), 5.03 (p, J = 6.7 Hz, 2H), 4.36 (s, 1H), 3.68 (s, 1H), 3.61 (s, 3H), 1.85 – 1.05 (m, 2H), 1.37 (d, J = 7.9 Hz, 21H); ^{13}C NMR (100 MHz, $CDCl_3$) δ 174.57, 152.00, 142.65, 131.79, 127.77, 125.81, 121.54, 116.12, 115.20, 79.21, 52.76, 51.77, 49.32, 28.46, 22.90; ^{11}B NMR (128 MHz, $CDCl_3$) δ -26.54 (t, J = 86.6 Hz); HRMS (EI): Calcd for $C_{24}H_{37}BN_3O_4$ $[M+H]^+$ 442.2877, found 442.2881.

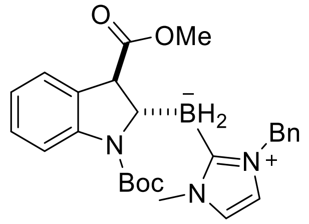

(3-Benzyl-1-methyl-1H-imidazol-3-ium-2-yl)(*trans*-(1-(tert-butoxycarbonyl)-3-(methoxycarbonyl)indolin-2-yl)dihydroborate (3af): white solid (43 mg, 95%); Mp. 105 – 106 °C. ^{1}H NMR (400 MHz, $CDCl_3$) δ 7.59 (brs, 1H), 7.27 (d, J = 7.5 Hz, 4H), 7.20 – 7.15 (m, 2H), 7.11 (td, J = 7.8, 1.4 Hz, 1H), 6.87 (td, J = 7.4, 1.1 Hz, 1H), 6.72 (s, 1H), 6.59 (s, 1H), 5.20 (s, 2H), 4.58 – 4.28 (m, 1H), 3.73 (s, 1H), 3.70 (s, 3H), 3.59 (s, 3H), 1.43-1.14 (m, 2H), 1.31 (s, 9H); ^{13}C NMR (101 MHz, $CDCl_3$) δ 174.52, 152.22, 142.31, 135.45, 131.76, 128.82, 128.44, 128.26, 127.91, 125.94, 120.55, 118.59, 115.93, 78.95, 52.59, 51.91, 51.83, 35.91, 28.38; ^{11}B NMR (128 MHz, $CDCl_3$) δ -26.37 (t, J = 86.9 Hz); HRMS (EI): Calcd for $C_{26}H_{33}BN_3O_4$ $[M+H]^+$ 462.2564, found 462.2563.

6. Gram-Scale Preparation of 3a and transformations of the Products

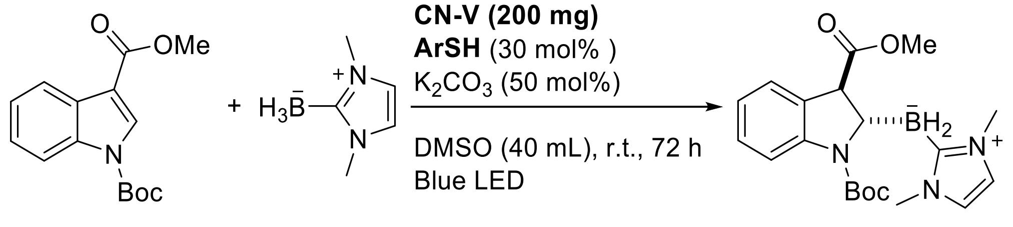

1a, 4 mmol **2a** **3a (1.27 g, 3.3 mmol)**

Gram-scale preparation of 3a: In a 100 mL Schlenk tube with a magnetic stir bar were placed **1a** (1.104 g, 4 mmol), **2a** (0.44 g, 6 mmol), **CN-V** (200 mg) and K_2CO_3 (0.276 g, 2 mmol). Under nitrogen atmosphere, DMSO (40 mL) and **ArSH** (160 μL, 1.5 mmol) were added, subsequently. The resulting mixture was sealed and degassed *via* freeze-pump-thaw three times. Then, the reaction was placed under a blue LED (2-meter strips, 18 W) and irradiated for 72 hrs at rt. The resulting mixture was quenched with H_2O and extracted with ether (20 mL×3). The combined organic layer was washed with brine, dried over $MgSO_4$, and concentrated in vacuo. The residue was purified by column chromatography. Silica gel chromatography (eluent: petroleum ether/EtOAc = 2/1) of the crude product afforded the desired compound as a white solid (1.27 g).

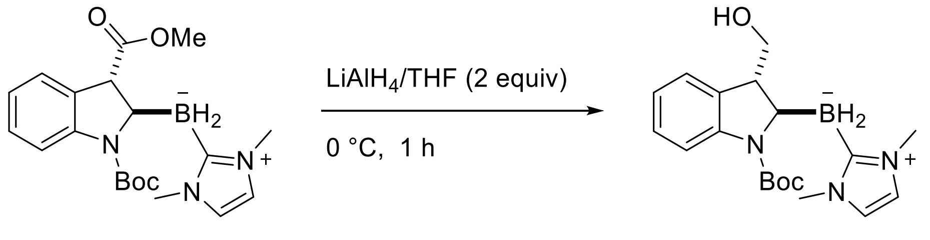

3a, 0.2 mmol **4**, 70%

***trans*-(1-(tert-Butoxycarbonyl)-3-(hydroxymethyl)indolin-2-yl)(1,3-dimethyl-1H-imidazol-3-ium-2-yl)dihydroborate (4):** According to literature report[5], **3a** (77.1 mg, 0.2 mmol) was added portionwise at 0 °C to 0.4 mL (0.4 mmol) of a 1 M $LiAlH_4$ solution (in THF). Then, the reaction mixture was continued to stir at 0 °C for 1 hour. The crude product was purified by column chromatography (PE/EA = 3:1) on silica gel to afford white solid (50 mg, 70% yield). Mp. 103 – 104 °C; ^{1}H NMR (400 MHz, $CDCl_3$) δ 7.39 (brs,1H), 7.20 (d, J = 7.3 Hz, 1H), 7.12 (t, J = 7.6 Hz, 1H), 6.89 (td, J = 7.4, 1.1 Hz, 1H), 6.74 (s, 2H), 3.84 (s, 1H), 3.66 (s, 6H), 3.63 – 3.43 (m, 2H), 3.03 (d, J = 7.2 Hz, 1H), 1.46 – 1.17 (m, 2H), 1.34 (s, 9H); ^{13}C NMR (100 MHz, $CDCl_3$) δ 152.51, 142.47, 135.26, 127.17, 123.04, 121.51, 120.01, 115.59, 78.85, 66.55, 36.29, 35.76, 28.30; ^{11}B NMR (128 MHz, $CDCl_3$) δ -26.68 (t, J = 85.9 Hz). HRMS (EI): Calcd for $C_{19}H_{29}BN_3O_3$ $[M+H]^+$ 358.2690, found 358.2691.

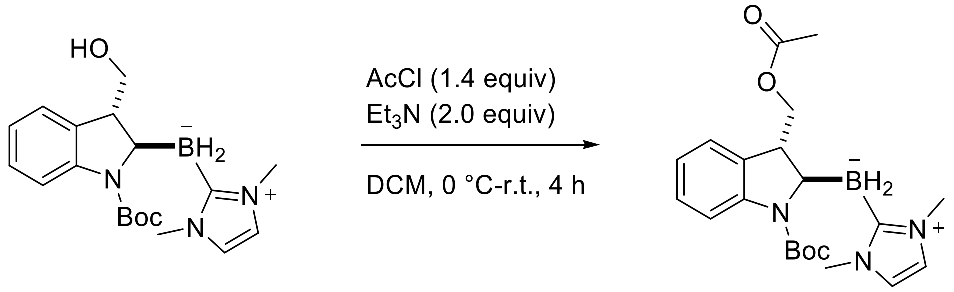

4 **5**

***trans*-(3-(Acetoxymethyl)-1-(tert-butoxycarbonyl)indolin-2-yl)(1,3-dimethyl-1H-imidazol-3-ium-2-yl)dihydroborate (5):** In a dried Schlenk tube equipped with a PTFE-coated stirring bar, **4** (35.7 mg, 0.1 mmol) was dissolved in CH_2Cl_2 (2 mL), and the solution was cooled to 0 °C.[6] Acetyl chloride (10 μL, 11.0 mg, 0.14 mmol) was added, followed by Et_3N (28 μL, 20 mg, 0.2 mmol). The resulting mixture was warmed up to room temperature and stirred for 4 hours and carefully quenched with deionized water (5 mL). Then, the aqueous layer was extracted three times with CH_2Cl_2 (10 mL

each time). The combined organic extracts were dried over $MgSO_4$ and condensed under vacuo. The residue was purified by flash column chromatography on silica (PE/EA = 3:1) to afford colorless oil (27.9 mg, 70% yield); ^1H NMR (400 MHz, $CDCl_3$) δ 7.36 (s, 1H), 7.20 (d, J = 7.3 Hz, 1H), 7.12 (t, J = 7.6 Hz, 1H), 6.88 (t, J = 7.1 Hz, 1H), 6.75 (s, 2H), 4.02 (d, J = 7.2 Hz, 2H), 3.91 – 3.74 (m, 1H), 3.66 (s, 6H), 3.15 (d, J = 7.5 Hz, 1H), 2.01 (s, 3H), 1.48-1.19 (m, 2H), 1.33 (s, 9H); ^{13}C NMR (100 MHz, $CDCl_3$) δ 171.18, 152.41, 142.45, 134.73, 127.35, 125.66, 121.55, 120.04, 115.61, 79.42, 67.83, 46.33, 35.78, 28.33, 20.93; ^{11}B NMR (128 MHz, $CDCl_3$) δ -26.80 (t, J = 86.4 Hz); HRMS (EI): Calcd for $C_{21}H_{31}BN_3O_4$ $[M+H]^+$ 400.3060, found 400.3061.

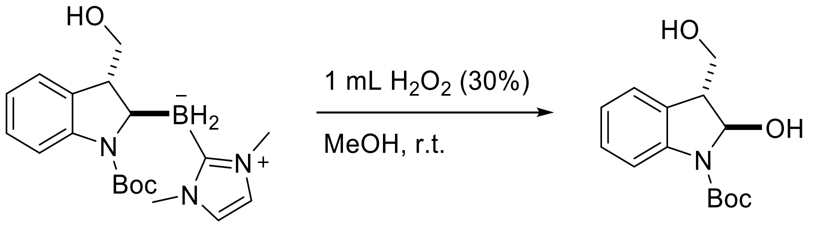

4 **6**, 85%

tert-Butyl-*trans*-2-hydroxy-3-(hydroxymethyl)indoline-1-carboxylate (6): To a solution of **4** (71.4 mg, 0.2 mmol) in MeOH (2 mL), H_2O_2 (30%, 1 mL) was added[7]. The mixture was stirred at room temperature for 12 hrs. The reaction mixture was quenched by saturated $Na_2S_2O_3$ aqueous solution (2 mL). The aqueous layer was extracted with EtOAc (3 × 5 mL). The combined organic solution was washed with brine, and then dried over anhydrous Na_2SO_4. After condensed under vacuum, the residue was purified by flash column chromatography on silica (PE/EA = 3:1) to afford colorless oil (45.1 mg, 85% yield); ^1H NMR (400 MHz, CD_3OD) δ 7.75 (brs, 1H), 7.32 – 7.19 (m, 2H), 7.01 (t, J = 7.8 Hz, 1H), 5.80 (s, 1H), 3.70 (dd, J = 11.1, 5.3 Hz, 1H), 3.23 (q, J = 7.2 Hz, 2H), 1.62 (s, 9H); ^{13}C NMR (100 MHz, CD_3OD) δ 153.92, 142.17, 129.02, 128.63, 126.08, 123.70, 115.89, 86.72, 64.37, 60.05, 53.63, 28.63; HRMS (EI): Calcd for $C_{14}H_{20}NO_4$ $[M+H]^+$ 266.3170, found 266.3173.

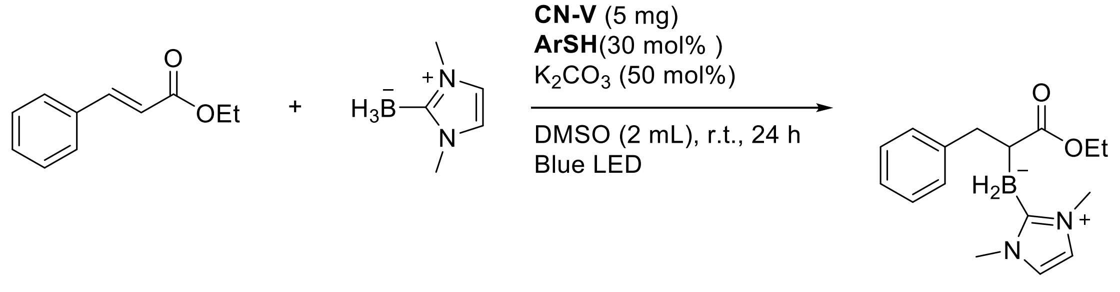

7 **2a** **8**, 44%

(1,3-Dimethyl-1H-imidazol-3-ium-2-yl)(3-ethoxy-3-oxo-1-phenyl-propyl)dihydroborate (8): In a 20 mL Schlenk tube with a magnetic stir bar were placed **CN-V** (5 mg), K_2CO_3 (6.9 mg, 0.05 mmol, 50 mol%), and NHC-BH_3 (**2a**, 0.15 mmol, 1.5 equiv). Under nitrogen atmosphere, ethyl cinnamate (**7**, 0.1 mmol, 1 equiv), 4-methoxybenzenethiol (**ArSH**, 4 µL, 0.03 mmol, 30 mol%), DMSO (2 mL) were added, subsequently. The resulting mixture was sealed and degassed via freeze-pump-thaw three times. Then, the reaction was placed under a blue LED (2-meter strips, 20 W) and irradiated for 24 hrs at room temperature. To the resulting mixture was added water (3 mL), followed by extraction with diethyl ether (5 mL × 3). The combined organic layer was washed with brine (10 mL × 3). The solvent was removed under vacuum. Silica gel chromatography (eluent: PE/EtOAc = 2/1) of the crude product to afford colorless oil (12.5 mg, 44% yield); ^{1}H NMR (400 MHz, $CDCl_3$) δ 7.15-7.23 (m, 4H), 7.05-7.10 (m, 1H), 6.80 (s, 2H), 3.76-3.87 (m, 2H), 3.74 (s, 6H), 3.12 (dd, J = 14.3, 10.2 Hz, 1H), 2.72 (dd, J = 14.3, 4.3 Hz, 1H), 2.19 (m, BH_2CH, 1H), 0.98 (t, J = 7.1 Hz, 3H)[8].

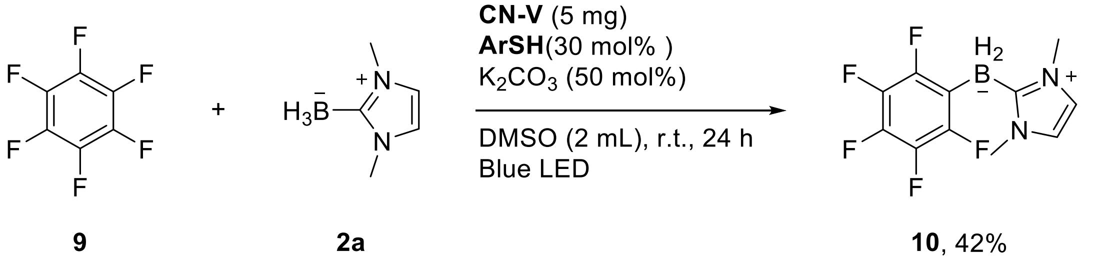

9 **2a** **10**, 42%

(1,3-Dimethyl-1H-imidazol-3-ium-2-yl)(perfluorophenyl)dihydroborate (10): In a 20 mL Schlenk tube with a magnetic stir bar were placed **CN-V** (5 mg), K_2CO_3 (6.9 mg, 0.05 mmol, 50 mol%), and NHC-BH_3 (**2a**, 0.15 mmol, 1.5 equiv). Under

nitrogen atmosphere, perfluorobenzene (**9**, 0.1 mmol, 1 equiv), 4-methoxybenzenethiol (**ArSH**, 4 μL, 0.03 mmol, 30 mol%), DMSO (2 mL) were added, subsequently. The resulting mixture was sealed and degassed via freeze-pump-thaw three times. Then, the reaction was placed under a blue LED (2-meter strips, 20 W) and irradiated for 24 hrs at room temperature. To the resulting mixture was added water (3 mL), followed by extraction with diethyl ether (5 mL × 3). The combined organic layer was washed with brine (10 mL × 3). The solvent was removed under vacuum. Silica gel chromatography (eluent: PE/EtOAc = 2/1) of the crude product to afford colorless oil (11.6 mg, 42% yield); ^{1}H NMR (400 MHz, $CDCl_3$) δ 6.87 (s, 2H), 3.74 (s, 6H), 2.17 (dd, J = 179.2, 88.2 Hz, 2H)[9].

7. Additional Experiments to Elucidate the Mechanism

7.1 Radical Inhibition Experiments

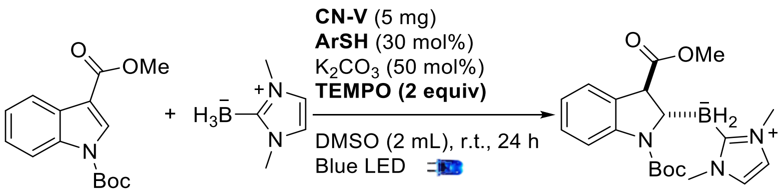

1a **2a** **3a**, 37%

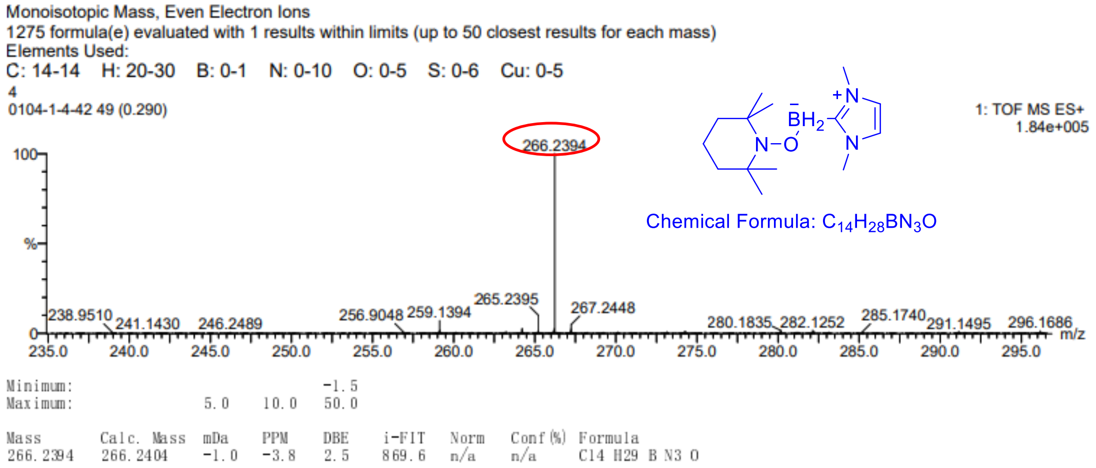

Supplementary Figure 15. HRMS spectrum of radical adducts.

After adding the radical initiator (TEMPO), the yield was greatly reduced, thus supporting a radical-based mechanism. The existence of boron radicals was confirmed

through the detection of the radical adduct by HRMS (Calcd for $C_{14}H_{29}BN_3O$ $[M+H]^+$ 266.2404, found 266.2394).

7.2 Evidence for the Presence of Carbon Radical

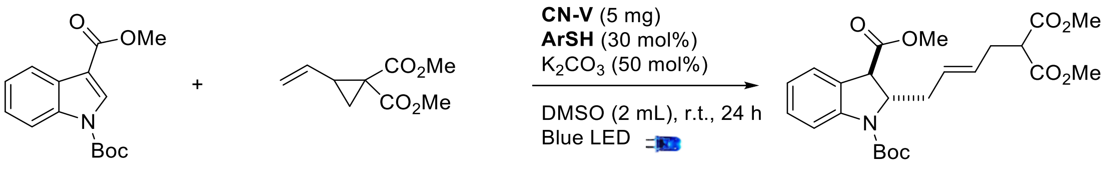

1a

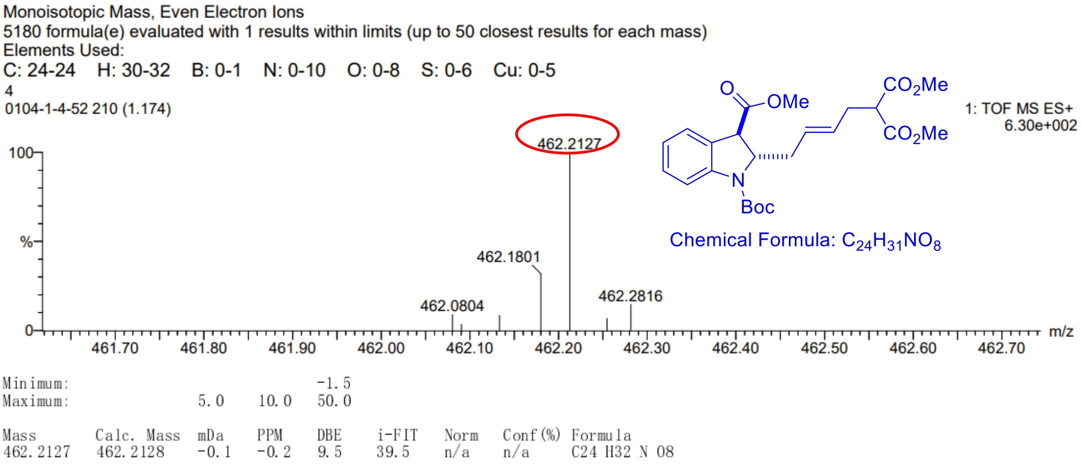

Supplementary Figure 16. HRMS spectrum of radical adducts.

To a sealed tube equipped with a magnetic stir bar were added **1a** (27.6 mg, 0.1 mmol, 1.0 equiv), dimethyl 2-vinylcyclopropane-1,1-dicarboxylate (27.6 mg, 0.15 mmol, 1.5 equiv), **CN-V** (5 mg), K_2CO_3 (13.8 mg, 0.05 mmol, 0.5 equiv) 4-methoxybenzenethiol (**ArSH**, 4 μL, 0.03 mmol, 30 mol%), and DMSO (2 mL). The reaction mixture was degassed via freeze-pump-thaw for 3 cycles. After the mixture was thoroughly degassed, the tube was sealed and placed under a blue LED (2-meter strips, 20 W). After irradiation for 24 hrs at room temperature, the hydroalkylation adduct was confirmed by HRMS (Calcd for $C_{24}H_{32}NO_8$ $[M+H]^+$ 462.2128, found 462.2127.)

7.3 Deuterium-Labeling Experiments

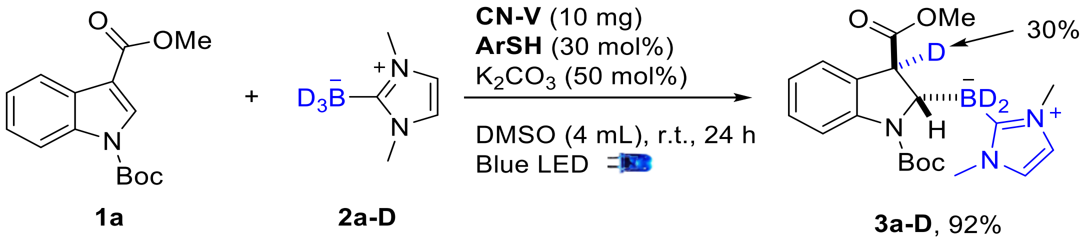

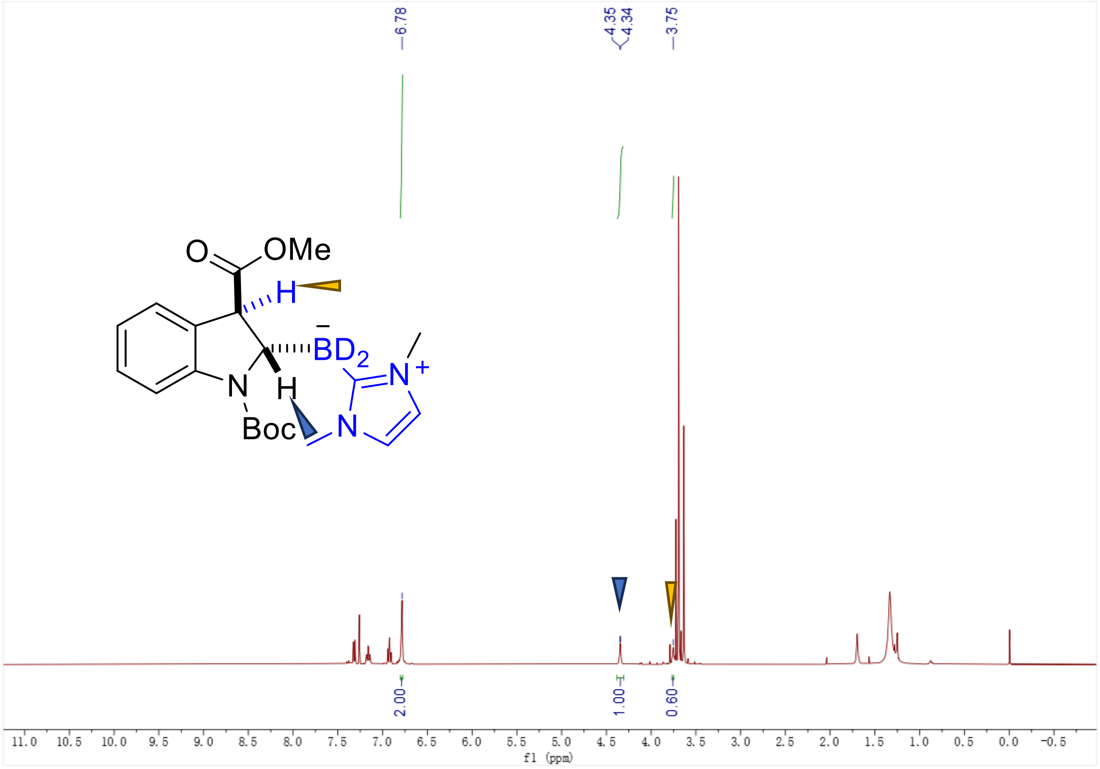

Supplementary Figure 17. Crude ^{1}H NMR spectrum for the reaction of **1a** and **2a-D** under standard conditions.

2a-D (16.5 mg, 0.15 mmol) was used to perform dearomatization under the standard conditions. The product (**3a-D**) was analyzed by ^{1}H NMR to determine the ratio of H-D exchange. It was found that 30% of the hydrogen at the C3 position was deuterated, which proved that part of the hydrogen protons came from NHC–BH_3.

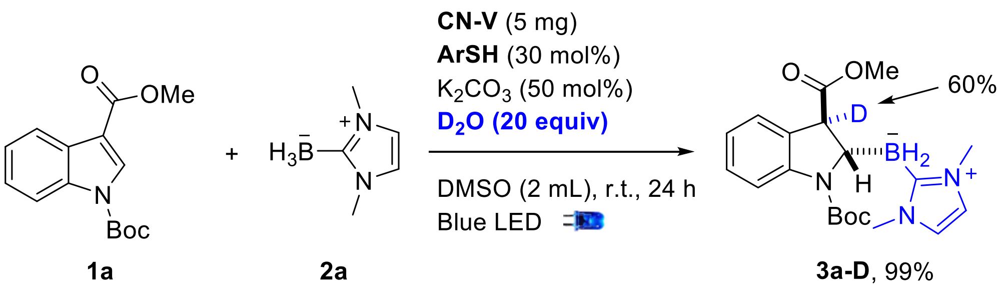

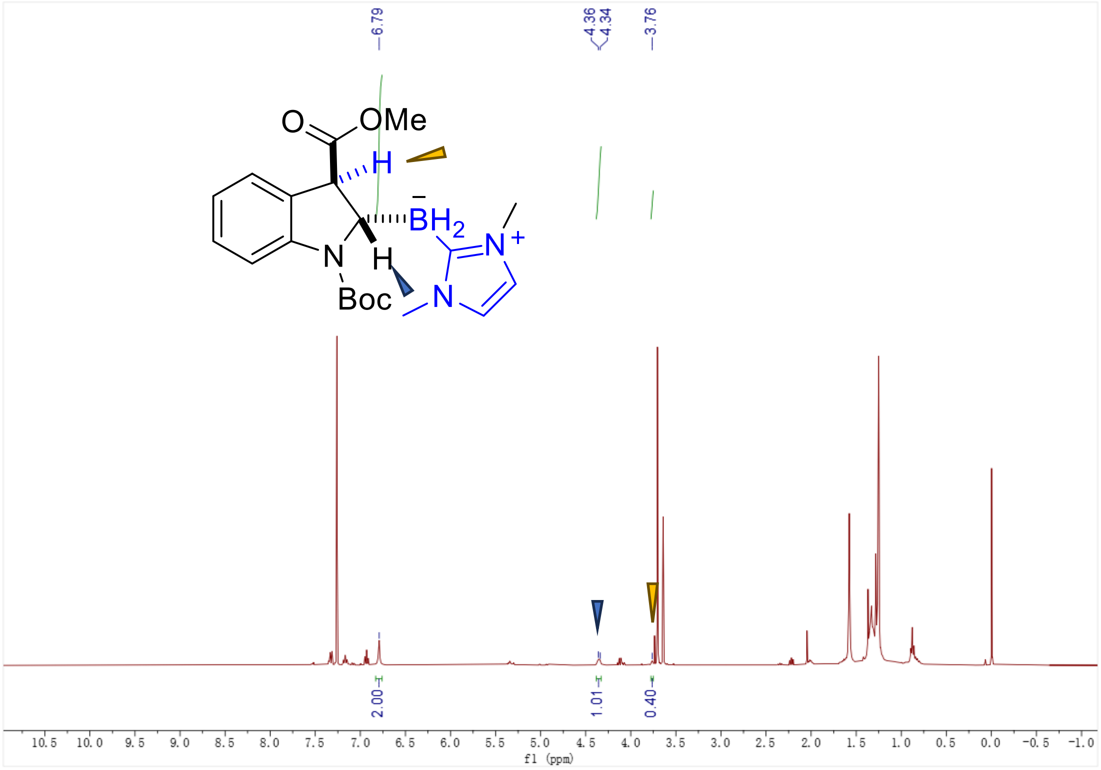

Supplementary Figure 18. Crude ^{1}H NMR spectrum of for the reaction of **1a** and **2a-D** under standard conditions with D_2O as the additives.

Under standard reaction conditions, 20 equivalents of deuterated water was added to the system. It was found from the ^{1}H NMR spectrum that 60% of the hydrogen at the C3 position was deuterated, which proved that a carbanion intermediate were generated during the reaction.

7.4 Cyclic Voltammetry Experiments

Cyclic Voltammograms were collected using Vertex. C. EIS Chenhua (China) with a typical three-electrode cell. Sample 0.01 M and tetrabutylammonium tetrafluoroborate 0.1 M in DMSO were used for tests. Measurements were performed using glassy carbon

working electrode, platinum wire counter electrode, and KCl-saturated Ag/AgCl reference electrode at a scan rate of 0.1 V/s.

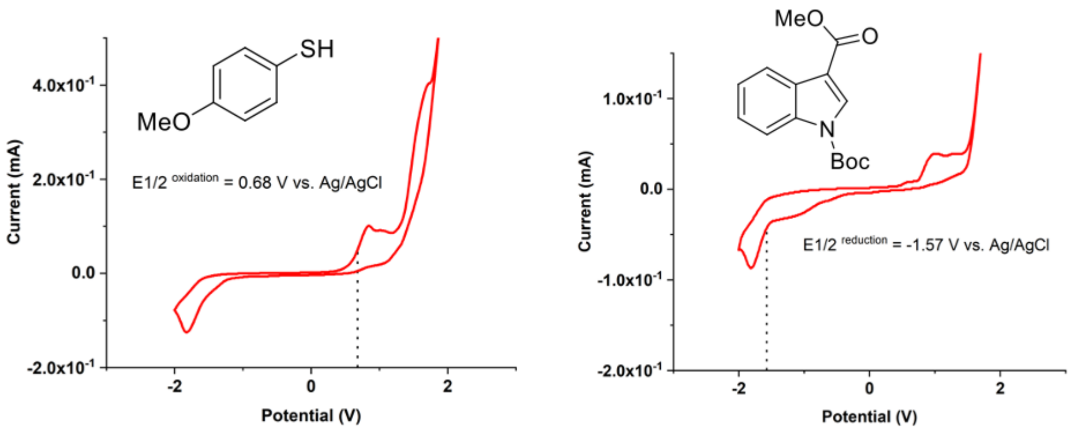

Supplementary Figure 19. Cyclic voltammetry (CV) of **1a** and **ArSH**

7.5 Stern-Volmer Quenching Experiments

Supplementary Figure 20. Luminescence quenching of **CN-V**

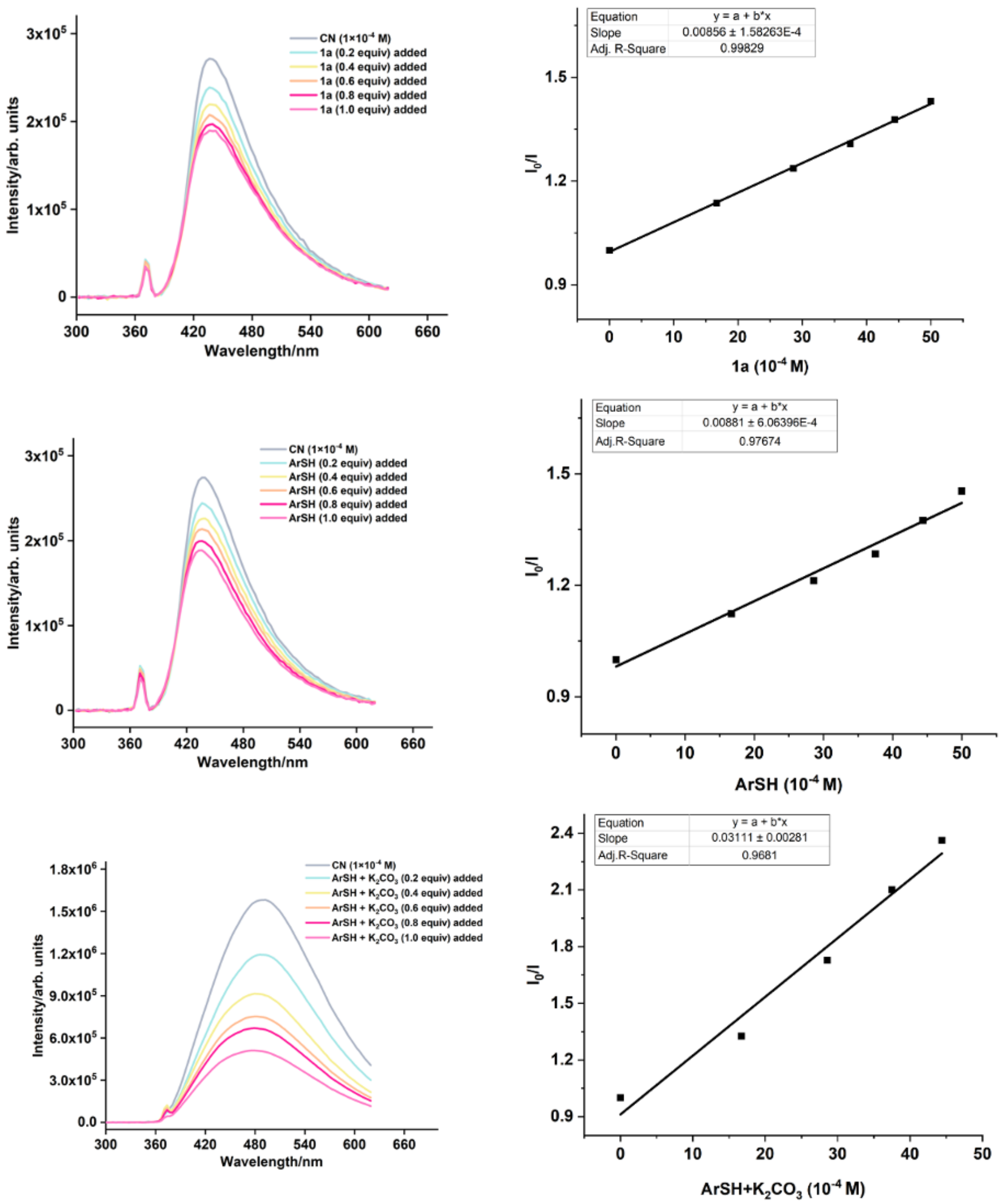

8. Quantum Yield Measurement

Determination of the light intensity at 470 nm: Following Yoon's protocol,[2] the photon flux of the spectrophotometer was determined by standard ferrioxalate actinometry. A 0.15 M solution of ferrioxalate was prepared by dissolving 2.21 g of potassium ferrioxalate hydrate in 30 mL of 0.05 M H_2SO_4. A buffered solution of phenanthroline

was prepared by dissolving 50 mg of phenanthroline and 11.25 g of sodium acetate in 50 mL of 0.5 M H_2SO_4. Both solutions were stored in the dark. To determine the photon flux of the spectrophotometer, 2.0 mL of the ferrioxalate solution was placed in a cuvette and irradiated for 90.0 seconds at λ = 470 nm with an emission slit width at 10.0 nm. After irradiation, 0.35 mL of the phenanthroline solution was added to the cuvette. The solution was then allowed to rest for 1 h to allow the ferrous ions to completely coordinate to the phenanthroline. The absorbance of the solution was measured at 510 nm. A non-irradiated sample was also prepared and the absorbance at 510 nm measured. Conversion was calculated using eq (1).

$$\text{mol Fe}^{2+} = \frac{\text{V}\cdot\Delta\text{A}}{1\cdot\varepsilon} \quad (1)$$

Where V is the total volume (0.00235 L) of the solution after addition of phenanthroline, ΔA is the difference in absorbance at 510 nm between the irradiated and non-irradiated solutions, l is the path length (1.000 cm), and ε is the molar absorptivity at 510 nm (11,100 L mol^{-1} cm^{-1}). The photon flux can be calculated using eq (2).

$$Photon\ flux = \frac{\text{mol Fe}^{2+}}{\Phi\cdot\text{t}\cdot\text{f}} \quad (2)$$

Where Φ is the quantum yield for the ferrioxalate actinometer (0.92 for a 0.15 M solution at λ = 468 nm),[3] t is the time (90.0 s), and f is the fraction of light absorbed at λ = 470 nm (0.14, vide infra).[4] The photon flux was calculated (average of three experiments) to be 3.22 × 10^{-8} einstein s^{-1}.

$$\text{mol Fe}^{2+} = \frac{0.00235\ \text{L}\cdot 1.76}{1.000\ \text{cm}\cdot 11100\ \text{L mol}^{-1}\text{cm}^{-1}} = 3.73\ \times\ 10^{-7}\ \text{mol}$$

$$Photon\ flux = \frac{3.73\times 10^{-7}\ \text{mol}}{0.92\cdot 90.0\ \text{s}\cdot 0.14} = 3.22\times 10^{-8}\ \text{mol}$$

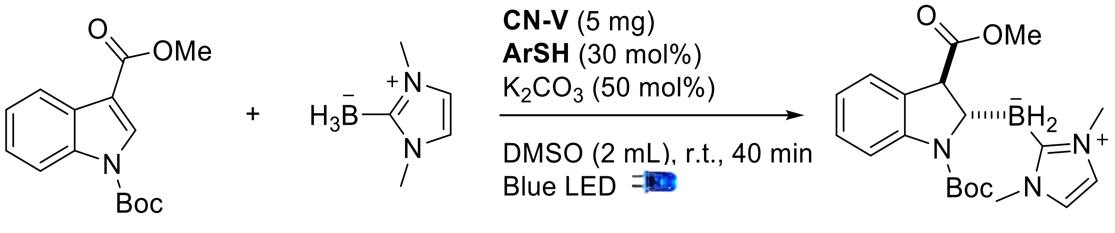

1a (0.1 mmol) **2a** (1.5 equiv) **3a**, 10%

In a 20 mL Schlenk tube with a magnetic stir bar were placed **CN-V** (5 mg), K_2CO_3 (6.9 mg, 0.05 mmol, 50 mol%), and NHC-BH_3 (**2a**, 0.15 mmol, 1.5 equiv). Under

nitrogen atmosphere, Indole Ester (**1a**, 0.1 mmol, 1 equiv), 4-Methoxybenzenethiol (**S1**, 4 μL, 0.03 mmol, 30 mol%), DMSO (2 mL) were added, subsequently. The sample was stirred and irradiated (λ = 470 nm) for 2400 s (40 min). After irradiation, the solvent was removed. The yield of the product (**3a**) formed was determined as 10% by crude ^{1}H NMR using 1,3,5-trimethoxybenzene as the internal standard. The quantum yield was determined using eq (3). Essentially all incident light (f > 0.999, vide infra) is absorbed by **CN-V** at the reaction conditions described above.

$$\Phi = \frac{mol\ product}{flux \cdot \mathrm{t} \cdot \mathrm{f}} \quad (3)$$

$$\Phi = \frac{0.1 \times 0.1 \times 10^{-3}\ mol}{3.21 \times 10^{-8}\ \mathrm{mol} \cdot 2400\ \mathrm{s} \cdot 1.00} = 0.13$$

9. X-Ray Crystal Data

Supplementary Table 5. Crystal data and structure refinement for **3a**

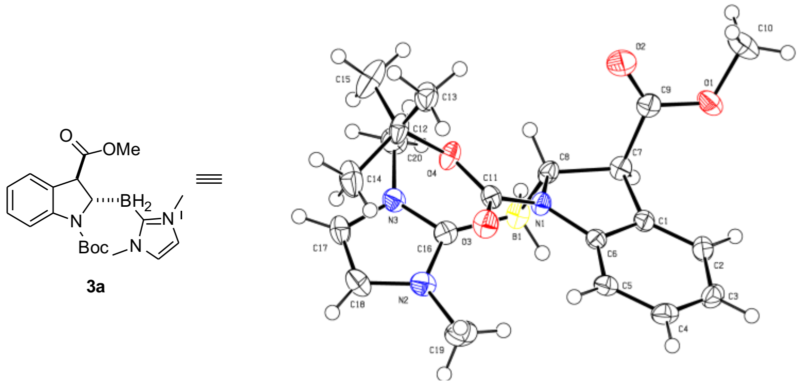

Identification code	2204097
Empirical formula	$C_{20}H_{28}BN_3O_4$
Formula weight	385.26
Temperature/K	149.94(13)
Crystal system	orthorhombic
Space group	Pbca
a/Å	15.2966(10)
b/Å	14.4211(12)
c/Å	18.6967(16)
α/°	90
β/°	90
γ/°	90
Volume/Å^3	4124.4(6)
Z	8
ρ_{calc}g/cm^3	1.241
μ/mm^{-1}	0.086
F(000)	1648.0

Crystal size/mm^3	0.14 × 0.12 × 0.1
Radiation	Mo Kα (λ = 0.71073)
2Θ range for data collection/°	4.358 to 49.994
Index ranges	-13 ≤ h ≤ 18, -11 ≤ k ≤ 17, -21 ≤ l ≤ 22
Reflections collected	12514
Independent reflections	3624 [R_{int} = 0.0363, R_{sigma} = 0.0380]
Data/restraints/parameters	3624/0/259
Goodness-of-fit on F^2	1.025
Final R indexes [I>=2σ (I)]	R_1 = 0.0474, wR_2 = 0.1106
Final R indexes [all data]	R_1 = 0.0626, wR_2 = 0.1207
Largest diff. peak/hole / e Å^{-3}	0.25/-0.23

**Atom**	**Atom**	**Length/Å**
O1	C9	1.343(2)
O1	C10	1.446(2)
O2	C9	1.203(2)
O3	C11	1.218(2)
O4	C11	1.348(2)
O4	C12	1.482(2)
N1	C6	1.417(2)
N1	C8	1.501(2)
N1	C11	1.359(2)
N2	C16	1.349(2)
N2	C18	1.378(3)
N2	C19	1.460(3)
N3	C16	1.350(2)
N3	C17	1.378(3)
N3	C20	1.459(3)
C1	C2	1.385(3)
C1	C6	1.393(3)
C1	C7	1.520(3)
C2	C3	1.391(3)
C3	C4	1.380(3)
C4	C5	1.392(3)
C5	C6	1.387(3)
C7	C8	1.548(3)
C7	C9	1.513(3)
C8	B1	1.636(3)
C12	C13	1.515(3)
C12	C14	1.510(3)
C12	C15	1.515(3)
C16	B1	1.608(3)
C17	C18	1.334(3)

Supplementary Table 6. Crystal data and structure refinement for **3a'**

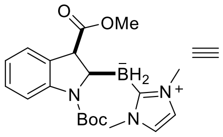

3a'

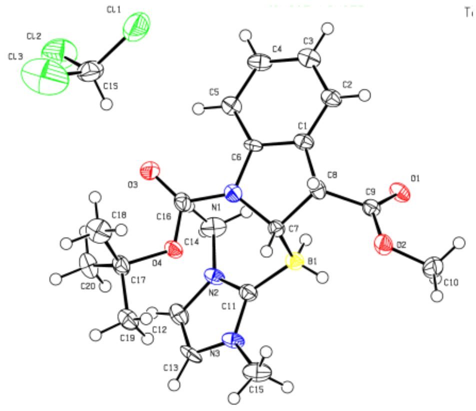

Identification code	**2204094**
Empirical formula	$C_{21}H_{29}BCl_3N_3O_4$
Formula weight	504.63
Temperature/K	150.00(10)
Crystal system	orthorhombic
Space group	$P2_12_12_1$
a/Å	7.4134(4)
b/Å	14.2825(8)
c/Å	23.8656(14)
α/°	90
β/°	90
γ/°	90
Volume/Å^3	2527.0(3)
Z	4
ρ_{calc}g/cm^3	1.326
μ/mm^{-1}	3.546
F(000)	1056.0
Crystal size/mm^3	0.14 × 0.1 × 0.08
Radiation	Cu Kα (λ = 1.54184)
2Θ range for data collection/°	7.214 to 149.506
Index ranges	$-9 \leq h \leq 8, -14 \leq k \leq 17, -29 \leq l \leq 20$
Reflections collected	6930
Independent reflections	4153 [R_{int} = 0.0615, R_{sigma} = 0.0850]
Data/restraints/parameters	4153/27/303
Goodness-of-fit on F^2	1.098
Final R indexes [I>=2σ (I)]	R_1 = 0.0882, wR_2 = 0.2522
Final R indexes [all data]	R_1 = 0.1079, wR_2 = 0.2637
Largest diff. peak/hole / e Å^{-3}	0.56/-0.44
Flack/Hooft parameter	0.08(3)/0.084(19)

**Atom**	**Atom**	**Length/Å**
Cl1	C1S	1.733(7)
Cl2	C1S	1.744(7)
Cl3	C1S	1.737(8)
O1	C9	1.180(6)
O2	C9	1.348(6)

Atom	Atom	Length/Å
O2	C10	1.449(6)
O3	C16	1.219(6)
O4	C16	1.346(7)
O4	C17	1.475(6)
N1	C6	1.413(7)
N1	C7	1.511(6)
N1	C16	1.356(7)
N2	C11	1.369(7)
N2	C12	1.393(7)
N2	C14	1.443(7)
N3	C11	1.353(7)
N3	C13	1.372(7)
N3	C15	1.449(7)
C1	C2	1.380(8)
C1	C6	1.400(7)
C1	C8	1.502(7)
C2	C3	1.388(8)
C3	C4	1.387(8)
C4	C5	1.385(8)
C5	C6	1.393(7)
C7	C8	1.580(7)
C7	B1	1.643(7)
C8	C9	1.512(7)
C11	B1	1.586(7)
C12	C13	1.348(8)
C17	C18	1.516(8)
C17	C19	1.528(8)

10. Computational Calculation Details on Reaction Pathways

Density functional theory (DFT) calculations were performed using the Gaussian09 program package[10, 11]. Geometries were optimized using the unrestricted hybrid density functional (UB3LYP) including empirical dispersion correction computed with Grimme's D3 formula and the def2-SVP basis set. Vibrational frequency analyses were conducted at the same theoretical level to obtain the thermodynamic energy corrections. The solvation model based on density (SMD) was employed to consider the solvent effect of DMSO[12].

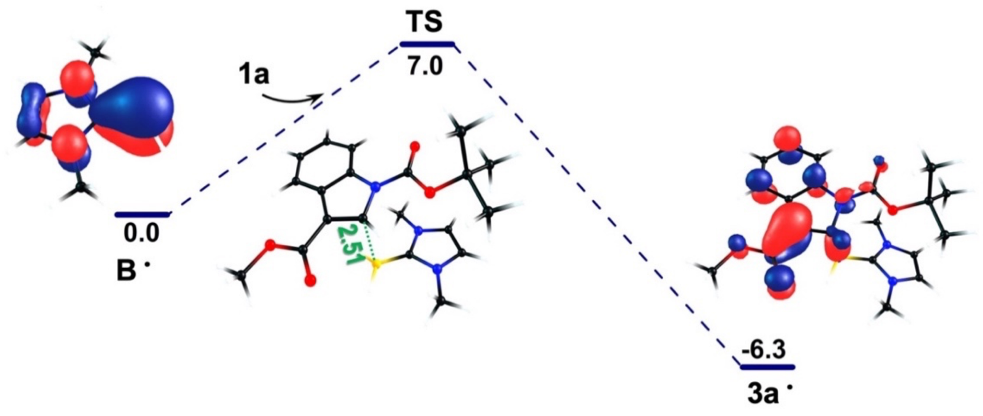

Supplementary Figure 21. Gibbs free energy change for the radical addition process.

^{1}H, ^{19}F, ^{11}B, ^{13}C-NMR Spectra

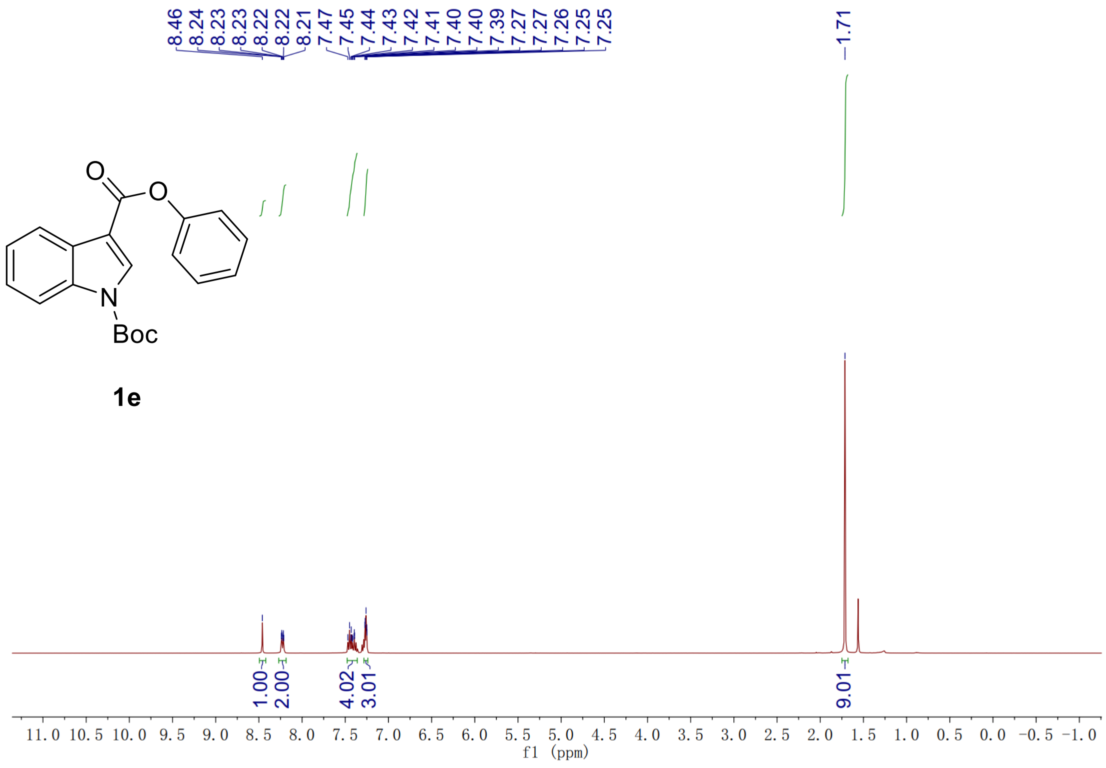

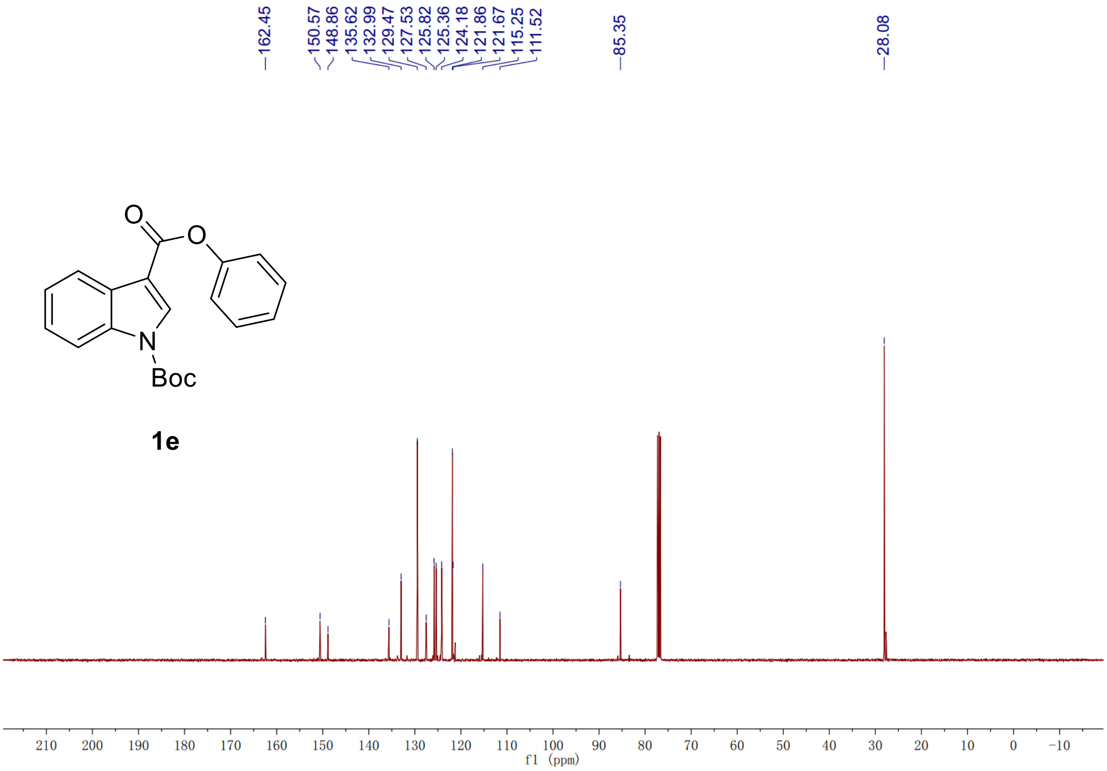
162.45
150.57
148.86
135.62
132.99
129.47
127.53
125.82
125.36
124.18
121.86
121.67
115.25
111.52
85.35
28.08
O
O
N
Boc
f1 (ppm)

1e

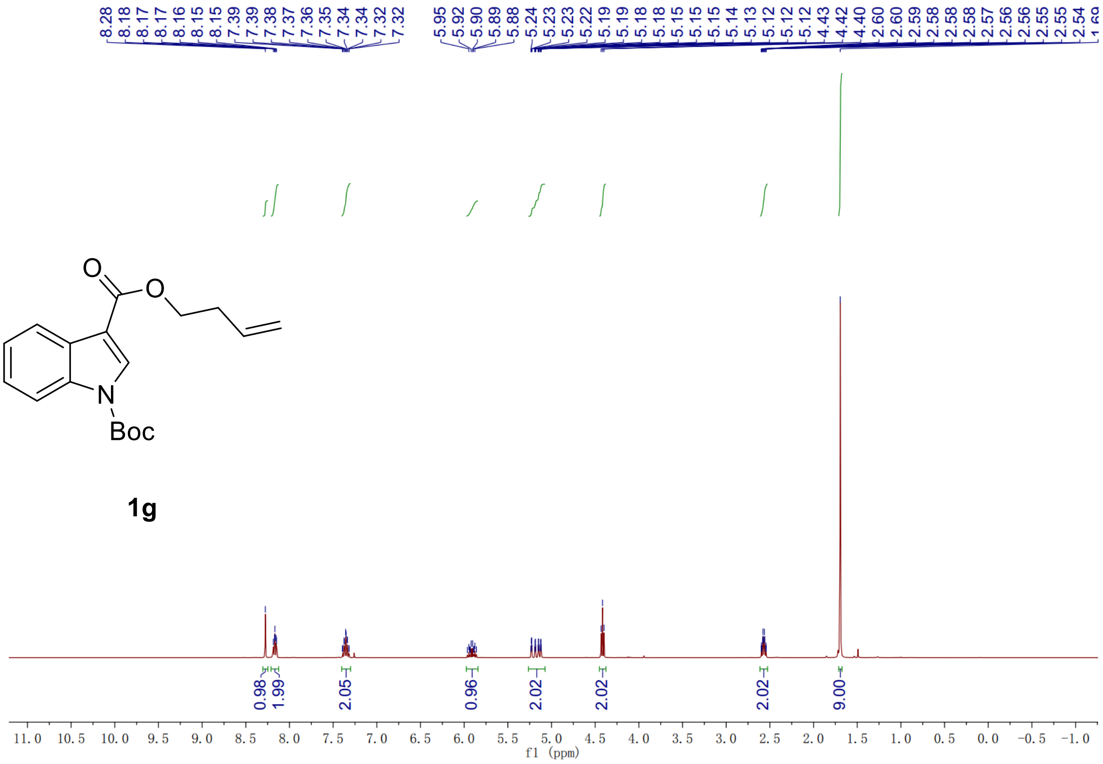
8.28
8.18
8.17
8.16
8.15
8.15
7.39
7.39
7.38
7.37
7.36
7.35
7.34
7.34
7.32
7.32
5.95
5.92
5.90
5.89
5.88
5.24
5.23
5.23
5.22
5.19
5.19
5.18
5.18
5.15
5.15
5.15
5.14
5.13
5.12
5.12
5.12
4.43
4.42
4.40
2.60
2.60
2.59
2.58
2.58
2.58
2.57
2.56
2.56
2.55
2.55
2.54
0.98
1.99
2.05
0.96
2.02
2.02
2.02
9.00
O
O
N
Boc
f1 (ppm)

1g

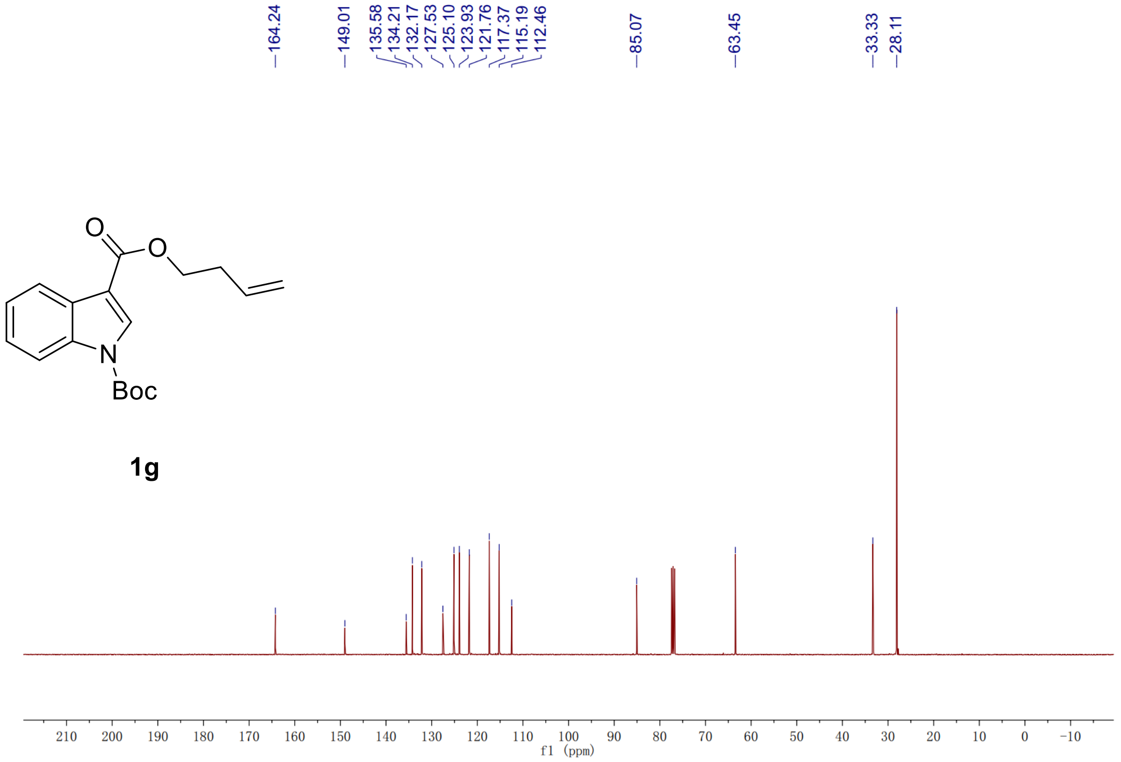
164.24
149.01
135.58
134.21
132.17
127.53
125.10
123.93
121.76
117.37
115.19
112.46
85.07
63.45
33.33
28.11
O
O
N
Boc
210 200 190 180 170 160 150 140 130 120 110 100 90 80 70 60 50 40 30 20 10 0 -10
f1 (ppm)

1g

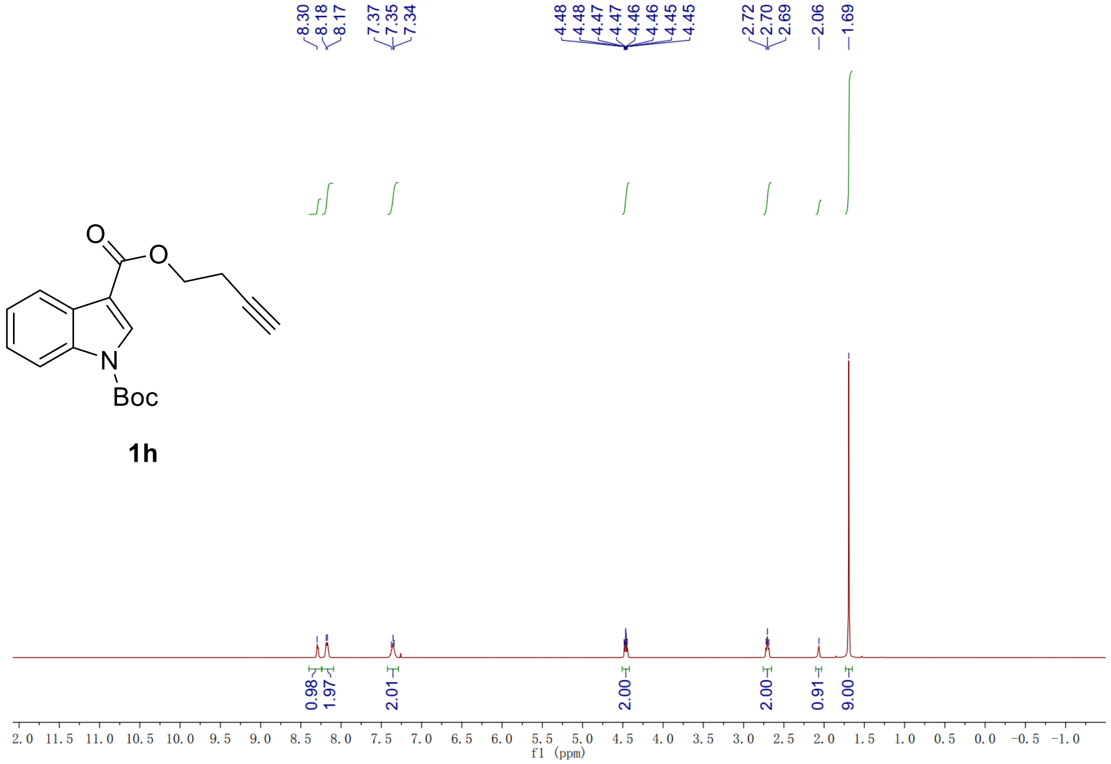
8.30
8.18
8.17
7.37
7.35
7.34
4.48
4.48
4.47
4.47
4.46
4.46
4.45
4.45
2.72
2.70
2.69
2.06
1.69
O
O
N
Boc
0.98
1.97
2.01
2.00
2.00
0.91
9.00
2.0 11.5 11.0 10.5 10.0 9.5 9.0 8.5 8.0 7.5 7.0 6.5 6.0 5.5 5.0 4.5 4.0 3.5 3.0 2.5 2.0 1.5 1.0 0.5 0.0 -0.5 -1.0
f1 (ppm)

1h

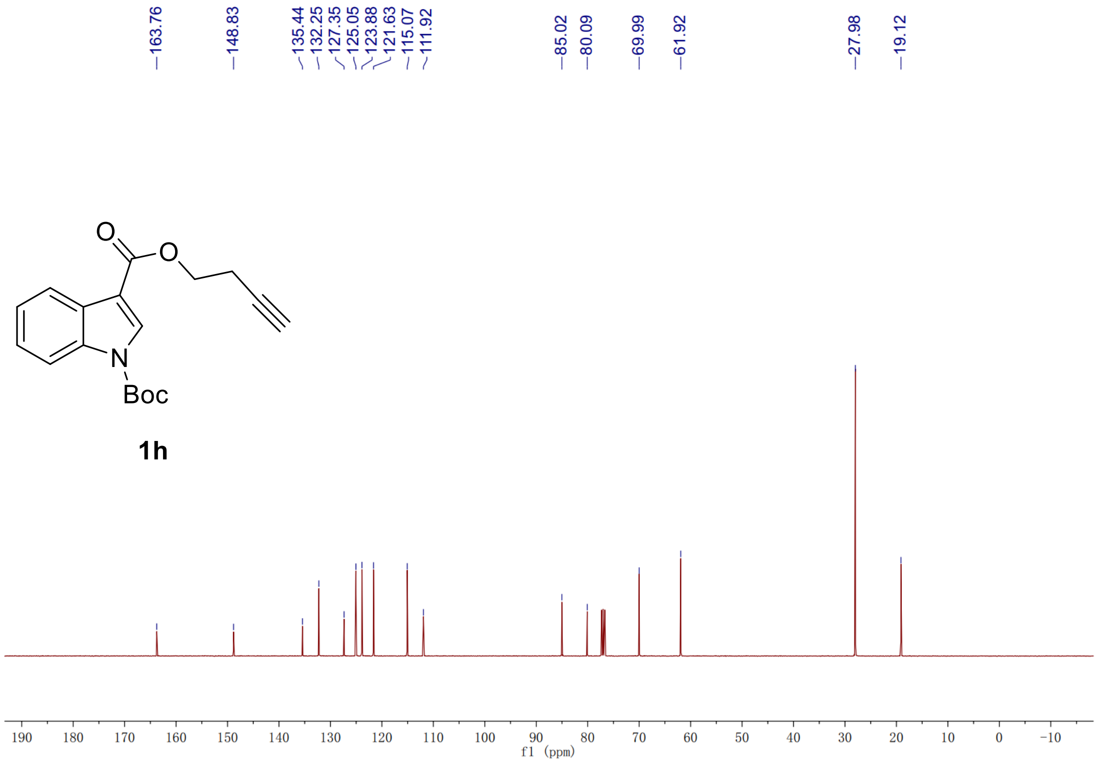

163.76
148.83
135.44
132.25
127.35
125.05
123.88
121.63
115.07
111.92
85.02
80.09
69.99
61.92
27.98
19.12
O
O
N
Boc
1h
190 180 170 160 150 140 130 120 110 100 90 80 70 60 50 40 30 20 10 0 -10
f1 (ppm)

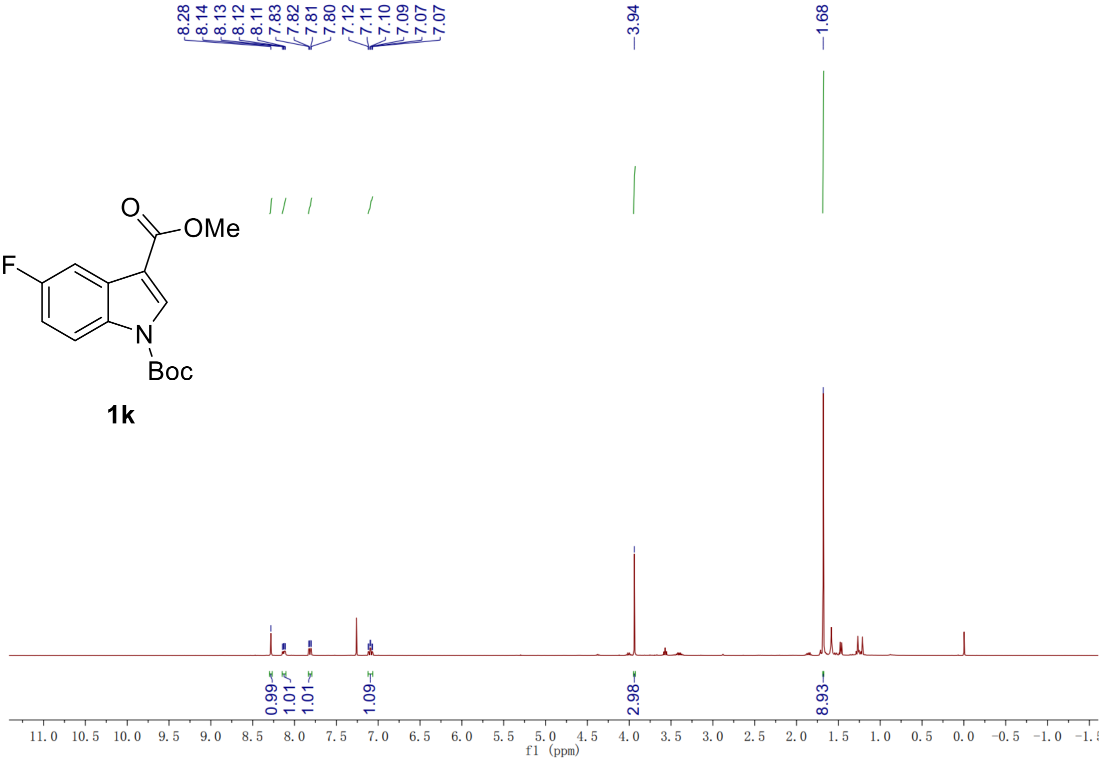

8.28
8.14
8.13
8.12
8.11
7.83
7.82
7.81
7.80
7.12
7.11
7.10
7.09
7.07
7.07
3.94
1.68
O
OMe
F
N
Boc
1k
0.99
1.01
1.01
1.09
2.98
8.93
11.0 10.5 10.0 9.5 9.0 8.5 8.0 7.5 7.0 6.5 6.0 5.5 5.0 4.5 4.0 3.5 3.0 2.5 2.0 1.5 1.0 0.5 0.0 -0.5 -1.0
f1 (ppm)

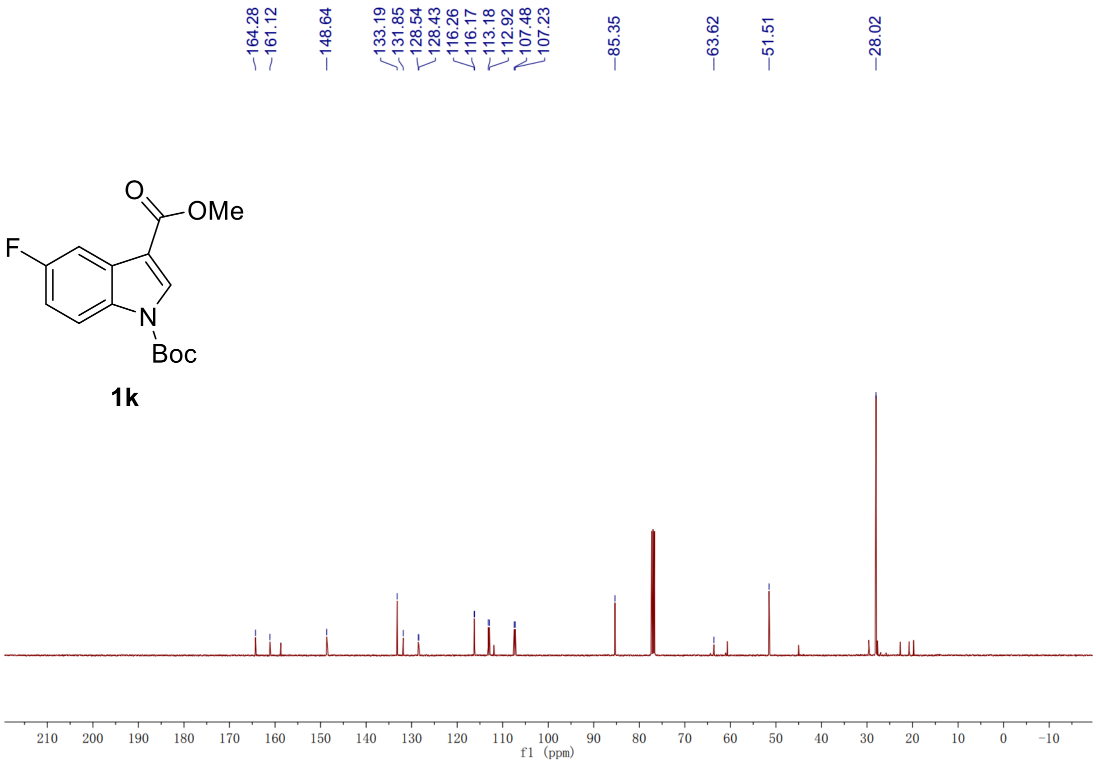
164.28
161.12
148.64
133.19
131.85
128.54
128.43
116.26
116.17
113.18
112.92
107.48
107.23
85.35
63.62
51.51
28.02
O
OMe
F
N
Boc
1k
210 200 190 180 170 160 150 140 130 120 110 100 90 80 70 60 50 40 30 20 10 0 -10
f1 (ppm)

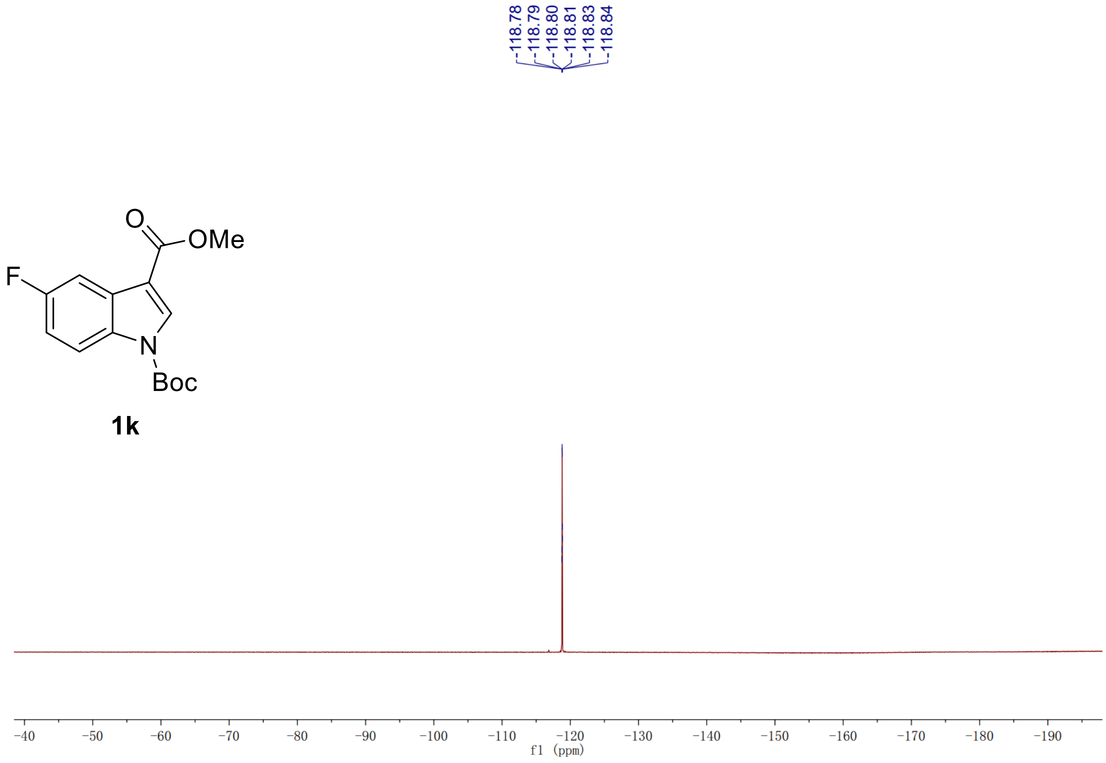
-118.78
-118.79
-118.80
-118.81
-118.83
-118.84
O
OMe
F
N
Boc
1k
-40 -50 -60 -70 -80 -90 -100 -110 -120 -130 -140 -150 -160 -170 -180 -190
f1 (ppm)

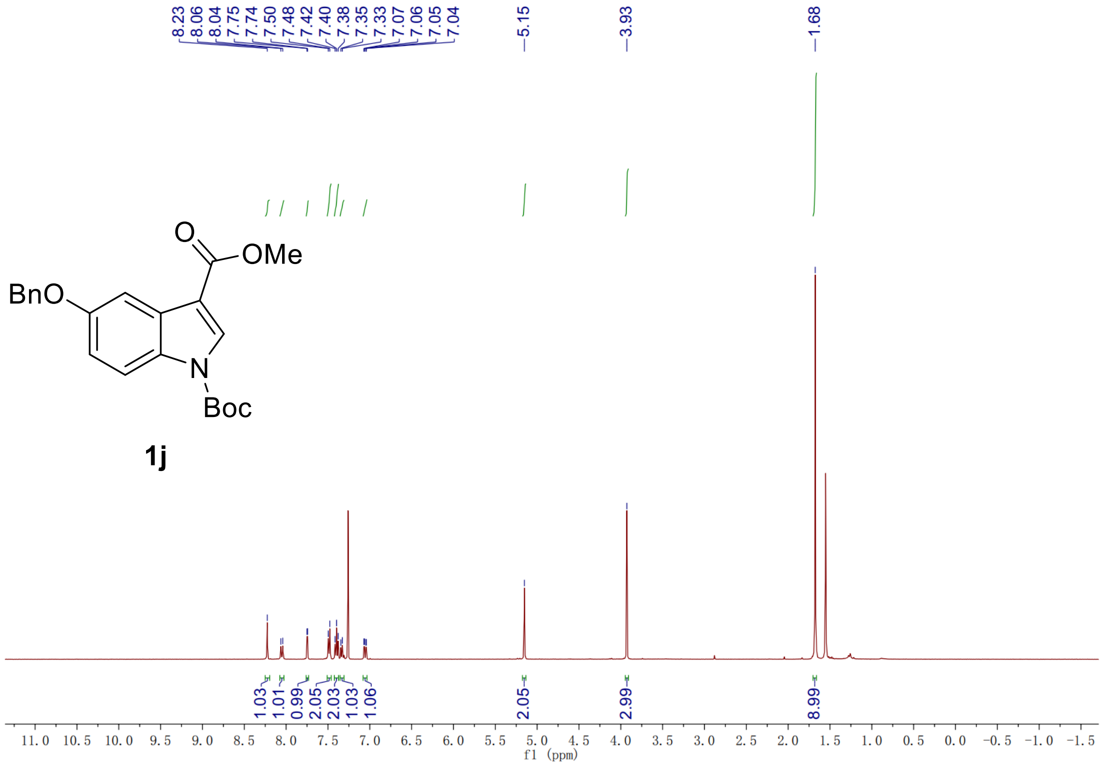

8.23
8.06
8.04
7.75
7.74
7.50
7.48
7.42
7.40
7.38
7.35
7.33
7.07
7.06
7.05
7.04
5.15
3.93
1.68
O
OMe
BnO
N
Boc
1j
1.03
1.01
0.99
2.05
2.03
1.03
1.06
2.05
2.99
8.99
11.0 10.5 10.0 9.5 9.0 8.5 8.0 7.5 7.0 6.5 6.0 5.5 5.0 4.5 4.0 3.5 3.0 2.5 2.0 1.5 1.0 0.5 0.0 -0.5 -1.0 -1.5
f1 (ppm)

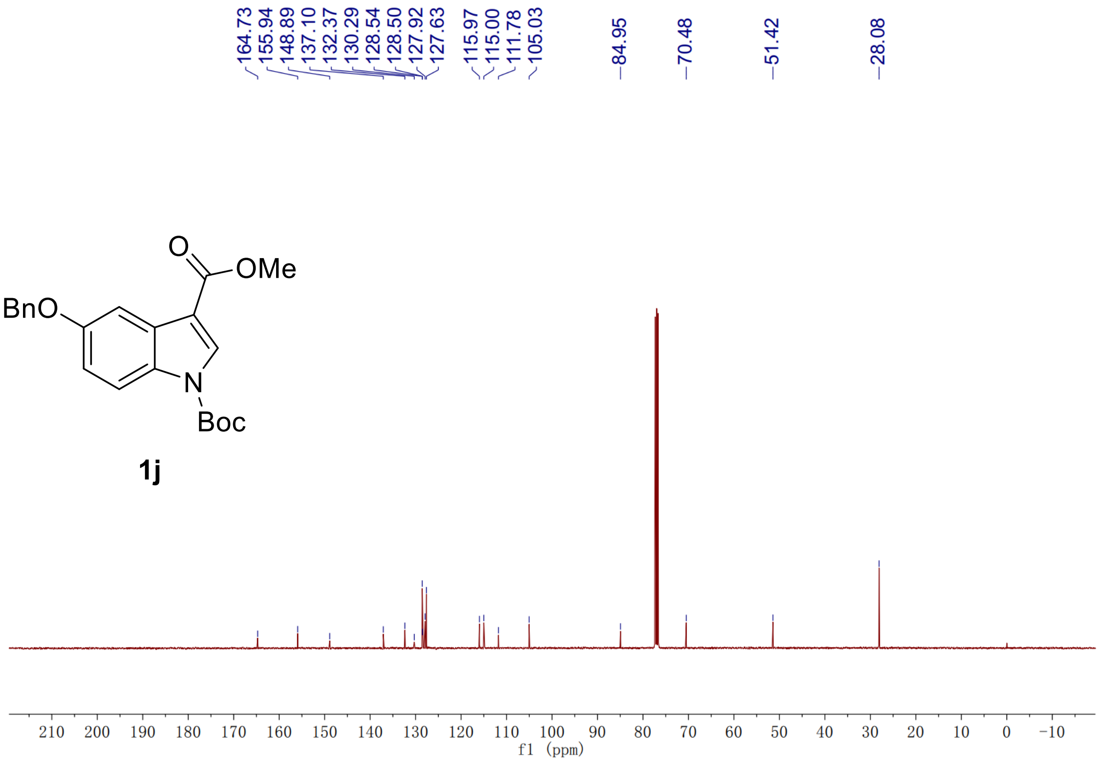

164.73
155.94
148.89
137.10
132.37
130.29
128.54
128.50
127.92
127.63
115.97
115.00
111.78
105.03
84.95
70.48
51.42
28.08
O
OMe
BnO
N
Boc
1j
210 200 190 180 170 160 150 140 130 120 110 100 90 80 70 60 50 40 30 20 10 0 -10
f1 (ppm)

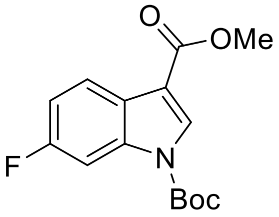

1n

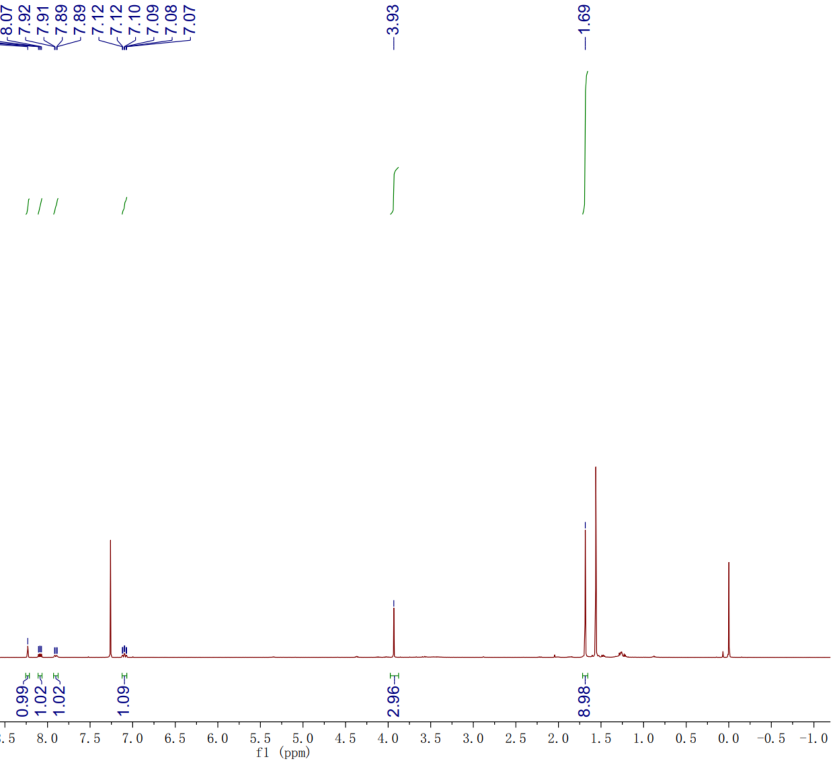
8.23 8.11 8.09 8.08 8.07 7.92 7.91 7.89 7.89 7.12 7.12 7.10 7.09 7.08 7.07
3.93
1.69
0.99 1.02 1.02 1.09 2.96 8.98
f1 (ppm)

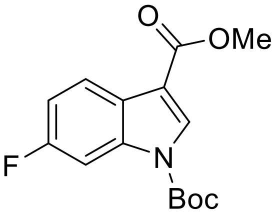

1n

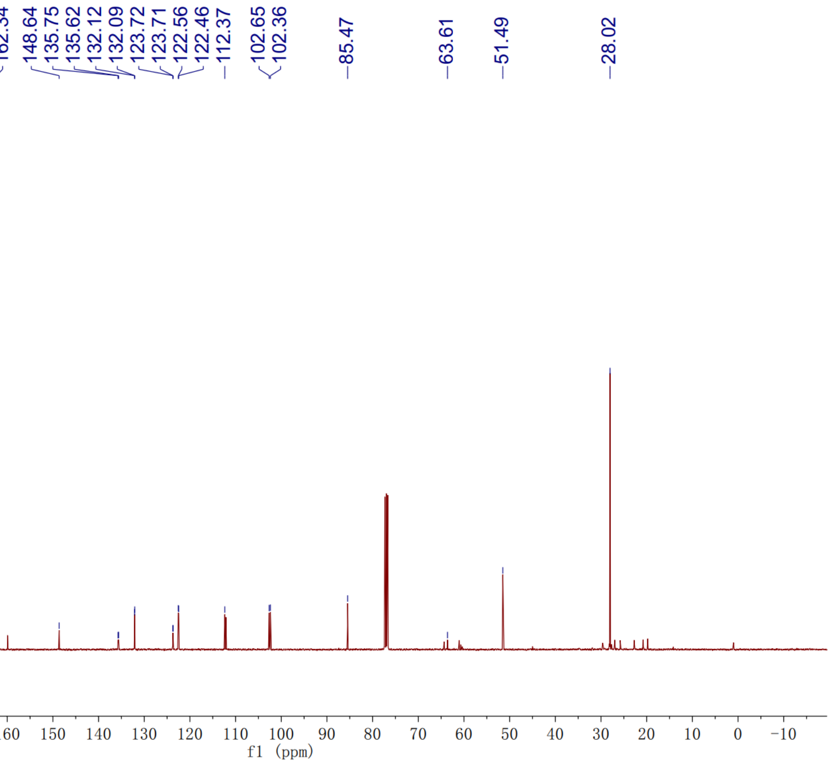
164.38 162.34 148.64 135.75 135.62 132.12 132.09 123.72 123.71 122.56 122.46 112.37 102.65 102.36
85.47
63.61
51.49
28.02
f1 (ppm)

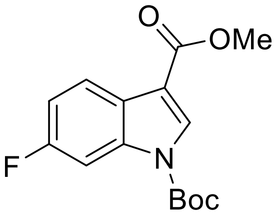
O
OMe
F
N
Boc

1n

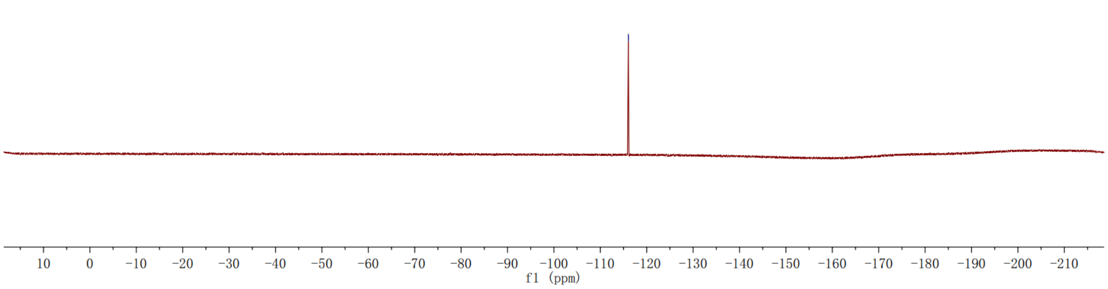
-116.03
-116.05
10 0 −10 −20 −30 −40 −50 −60 −70 −80 −90 −100 −110 −120 −130 −140 −150 −160 −170 −180 −190 −200 −210
f1 (ppm)

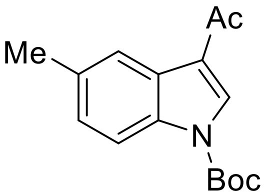
Ac
Me
N
Boc

1r

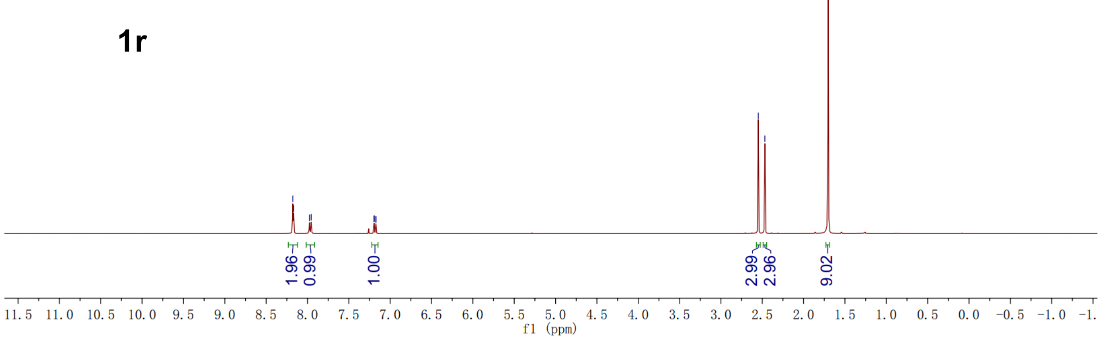
8.18
8.17
7.98
7.96
7.20
7.19
7.18
7.17
2.55
2.47
1.70
1.96
0.99
1.00
2.99
2.96
9.02
11.5 11.0 10.5 10.0 9.5 9.0 8.5 8.0 7.5 7.0 6.5 6.0 5.5 5.0 4.5 4.0 3.5 3.0 2.5 2.0 1.5 1.0 0.5 0.0 −0.5 −1.0 −1.
f1 (ppm)

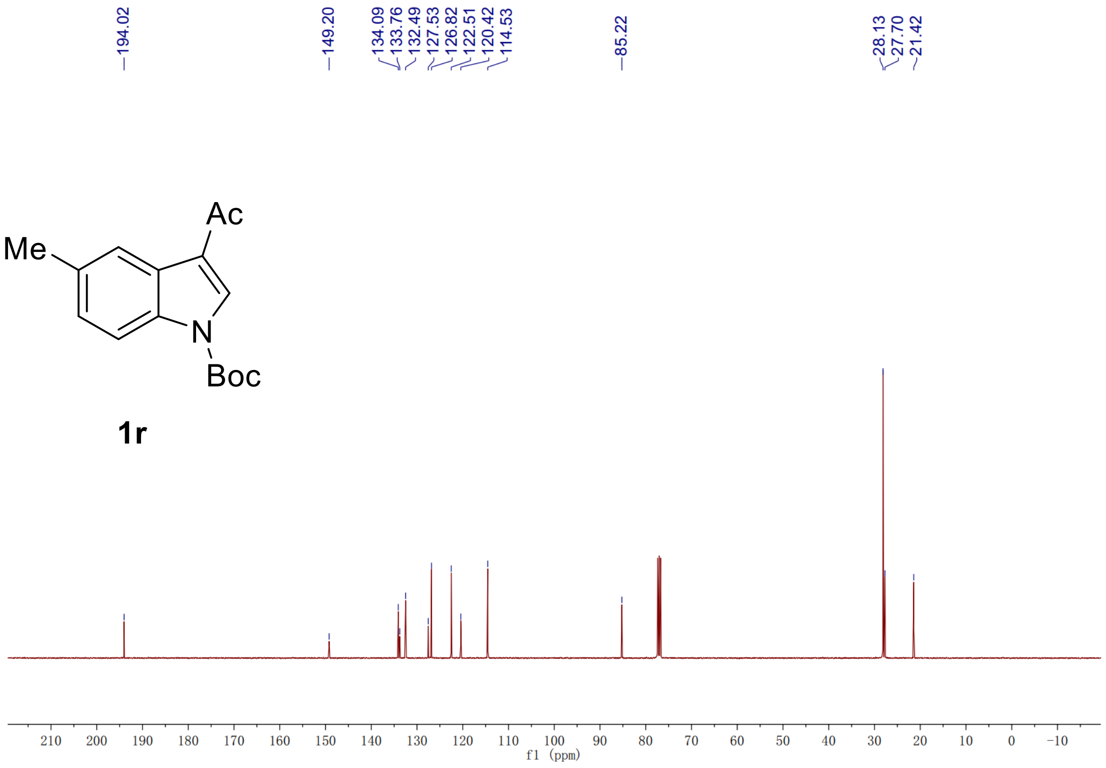
194.02
149.20
134.09
133.76
132.49
127.53
126.82
122.51
120.42
114.53
85.22
28.13
27.70
21.42
Ac
Me
N
Boc
210 200 190 180 170 160 150 140 130 120 110 100 90 80 70 60 50 40 30 20 10 0 −10
f1 (ppm)

1r

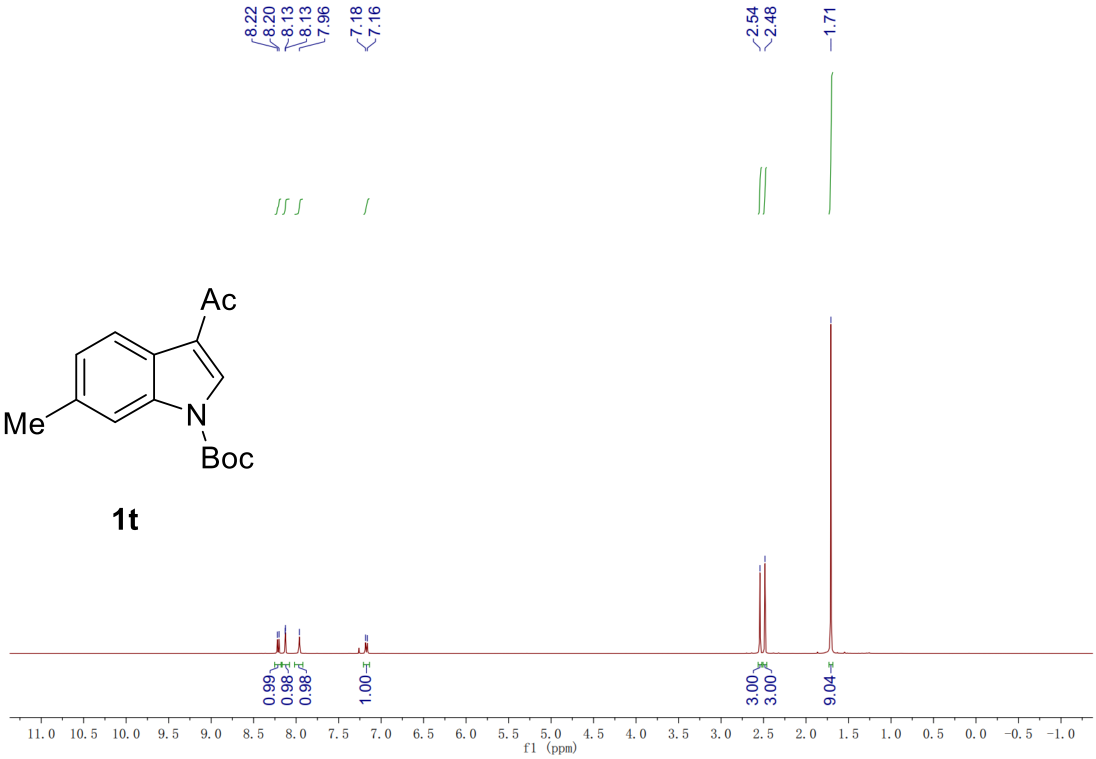
8.22
8.20
8.13
8.13
7.96
7.18
7.16
2.54
2.48
1.71
Ac
Me
N
Boc
0.99
0.98
0.98
1.00
3.00
3.00
9.04
11.0 10.5 10.0 9.5 9.0 8.5 8.0 7.5 7.0 6.5 6.0 5.5 5.0 4.5 4.0 3.5 3.0 2.5 2.0 1.5 1.0 0.5 0.0 −0.5 −1.0
f1 (ppm)

1t

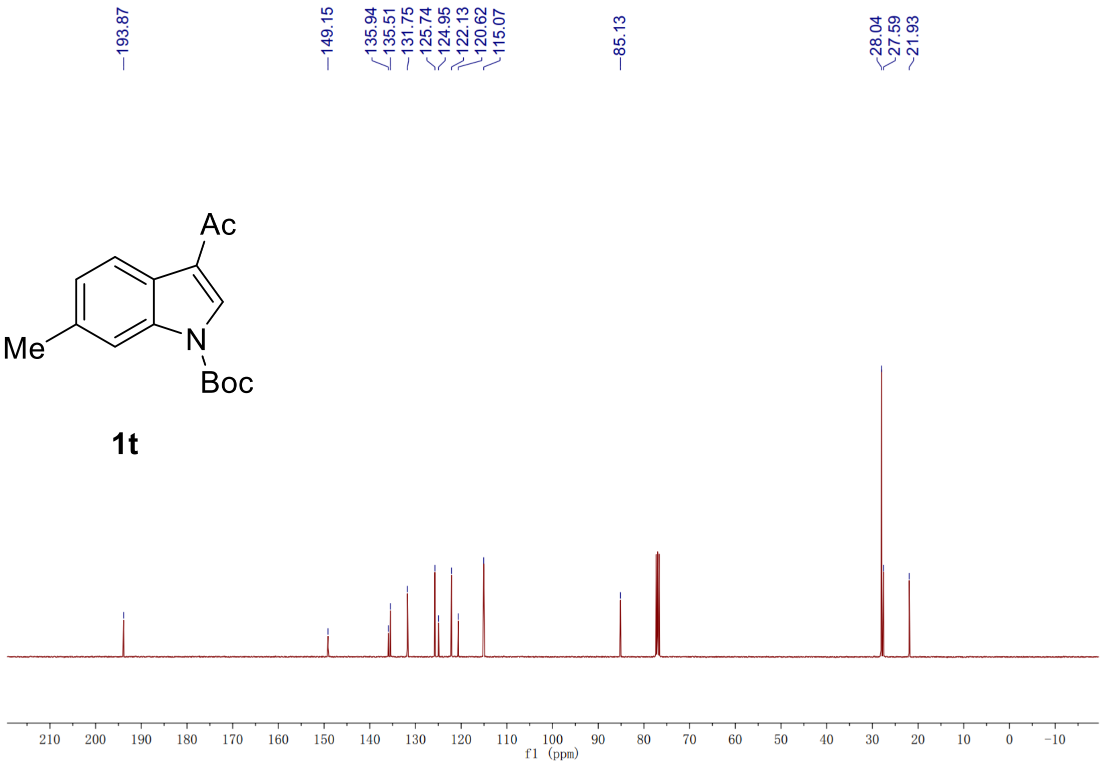
193.87
149.15
135.94
135.51
131.75
125.74
124.95
122.13
120.62
115.07
85.13
28.04
27.59
21.93
Ac
Me
N
Boc
1t
210 200 190 180 170 160 150 140 130 120 110 100 90 80 70 60 50 40 30 20 10 0 -10
f1 (ppm)

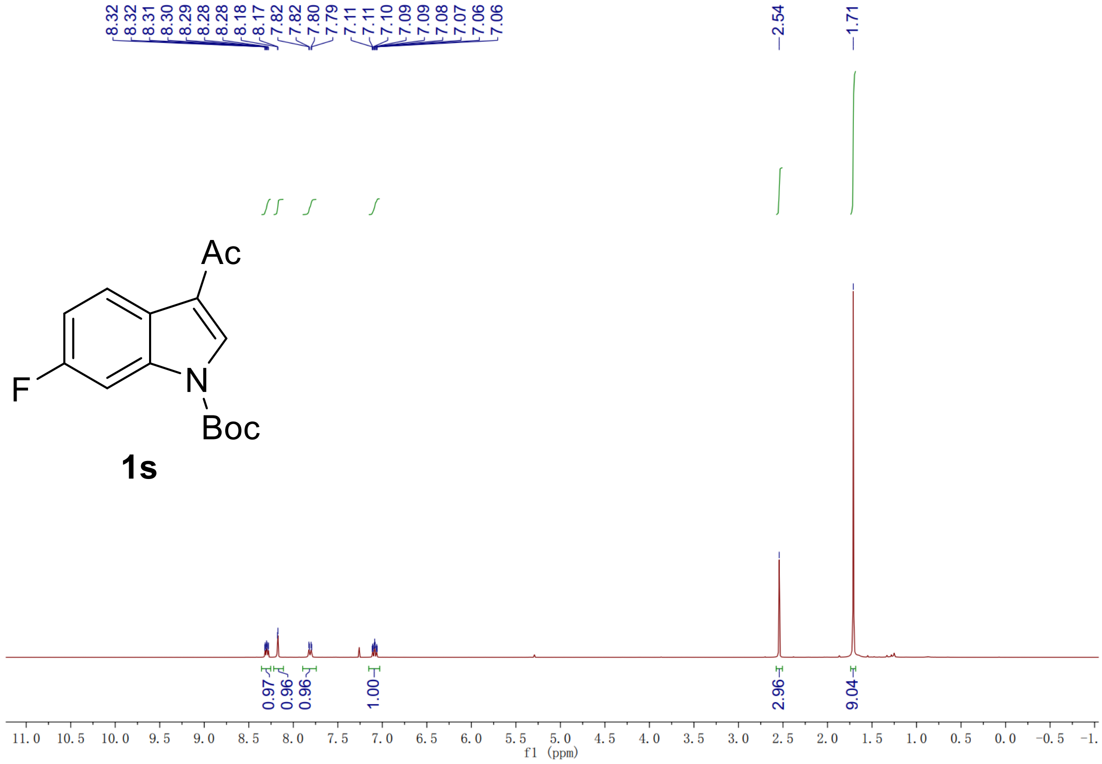
8.32
8.32
8.31
8.30
8.29
8.28
8.28
8.18
8.17
7.82
7.82
7.80
7.79
7.11
7.11
7.10
7.09
7.09
7.08
7.07
7.06
7.06
2.54
1.71
Ac
F
N
Boc
1s
0.97
0.96
0.96
1.00
2.96
9.04
11.0 10.5 10.0 9.5 9.0 8.5 8.0 7.5 7.0 6.5 6.0 5.5 5.0 4.5 4.0 3.5 3.0 2.5 2.0 1.5 1.0 0.5 0.0 -0.5 -1.
f1 (ppm)

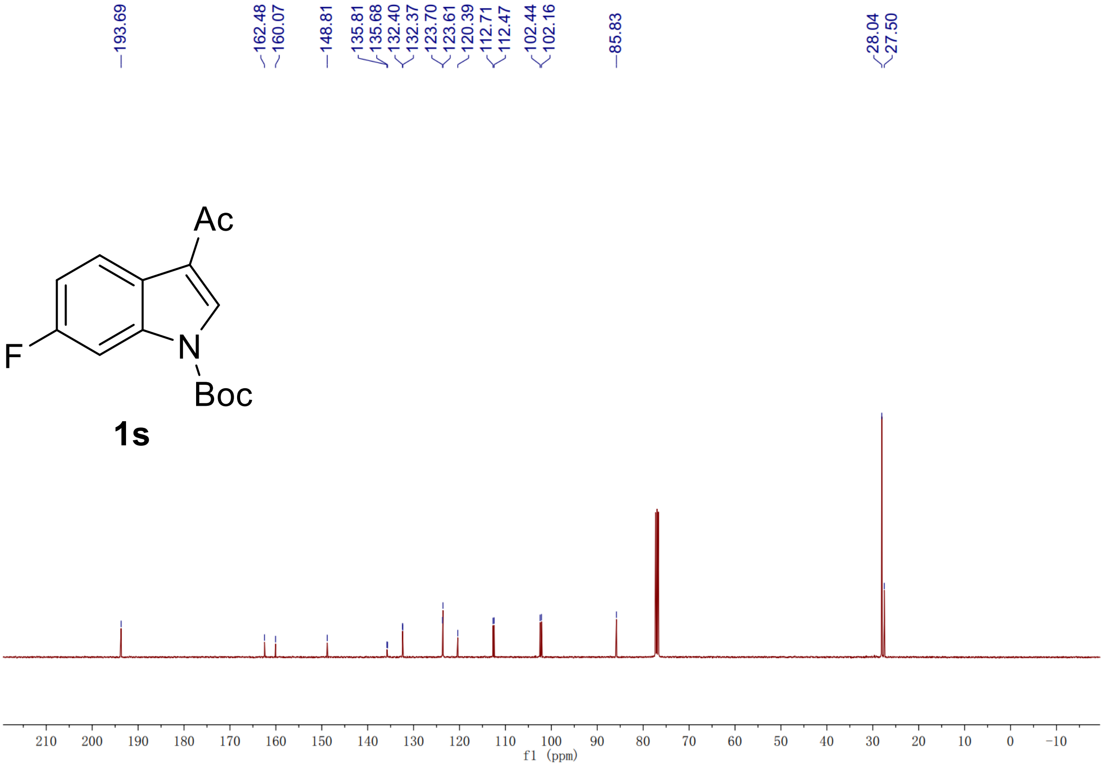
193.69
162.48
160.07
148.81
135.81
135.68
132.40
132.37
123.70
123.61
120.39
112.71
112.47
102.44
102.16
85.83
28.04
27.50
Ac
F
N
Boc
1s
210 200 190 180 170 160 150 140 130 120 110 100 90 80 70 60 50 40 30 20 10 0 −10
f1 (ppm)

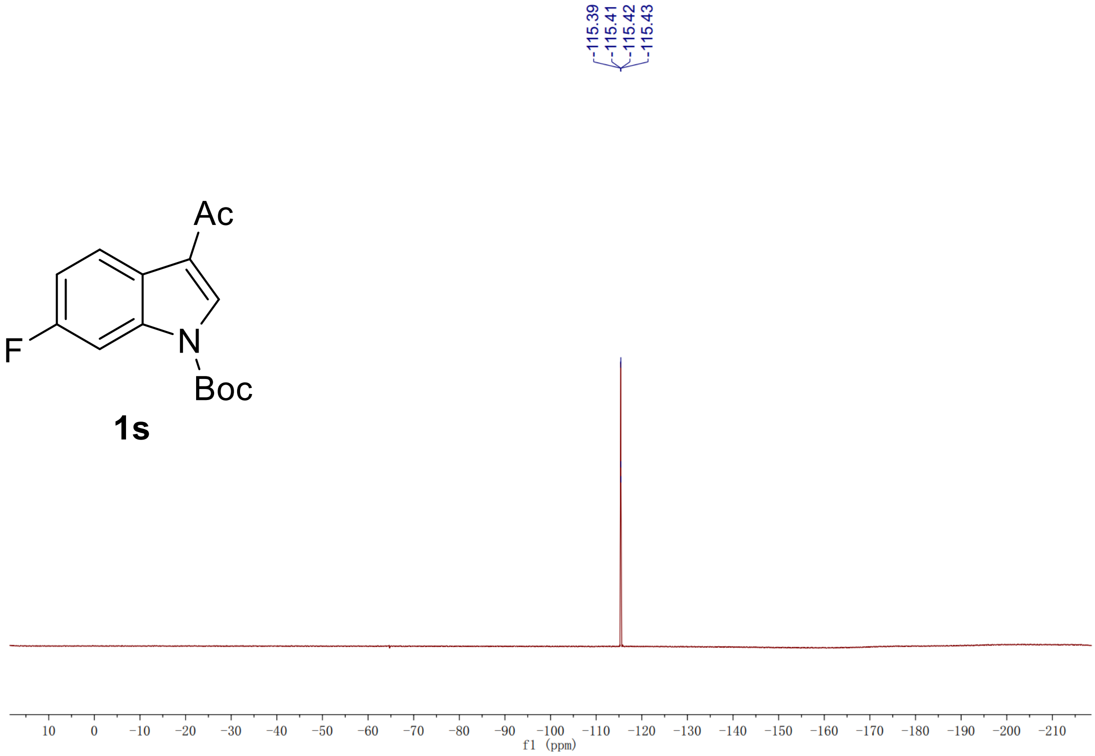
-115.39
-115.41
-115.42
-115.43
Ac
F
N
Boc
1s
10 0 −10 −20 −30 −40 −50 −60 −70 −80 −90 −100 −110 −120 −130 −140 −150 −160 −170 −180 −190 −200 −210
f1 (ppm)

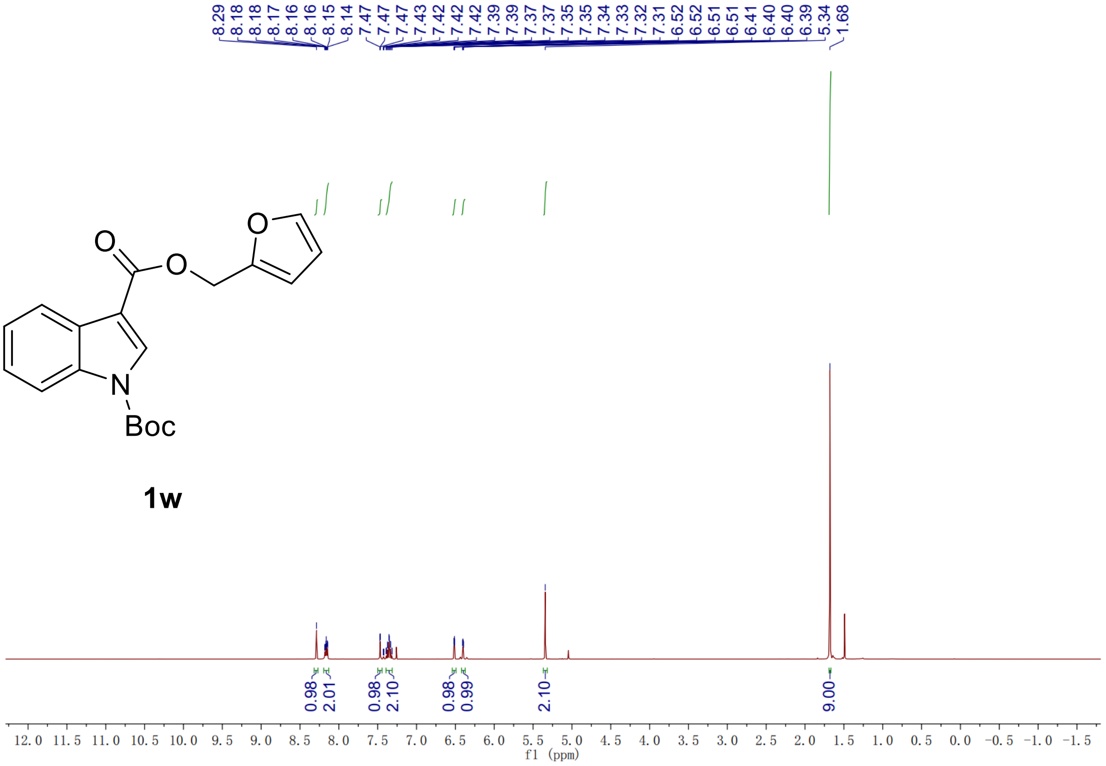
8.29
8.18
8.18
8.17
8.16
8.16
8.15
8.14
7.47
7.47
7.47
7.43
7.42
7.42
7.42
7.39
7.39
7.37
7.37
7.35
7.35
7.34
7.33
7.32
7.31
6.52
6.52
6.51
6.51
6.41
6.40
6.40
6.39
5.34
1.68
O
O
O
N
Boc
1w
0.98
2.01
0.98
2.10
0.98
0.99
2.10
9.00
12.0 11.5 11.0 10.5 10.0 9.5 9.0 8.5 8.0 7.5 7.0 6.5 6.0 5.5 5.0 4.5 4.0 3.5 3.0 2.5 2.0 1.5 1.0 0.5 0.0 -0.5 -1.0 -1.5
f1 (ppm)

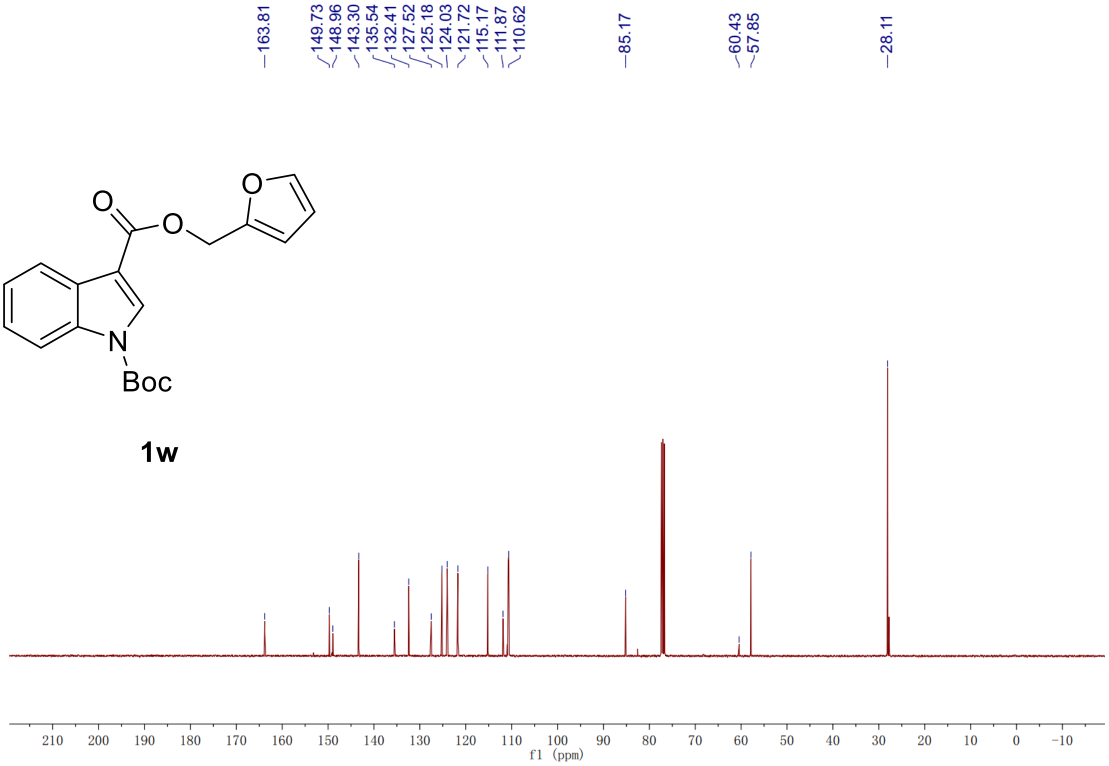
163.81
149.73
148.96
143.30
135.54
132.41
127.52
125.18
124.03
121.72
115.17
111.87
110.62
85.17
60.43
57.85
28.11
O
O
O
N
Boc
1w
210 200 190 180 170 160 150 140 130 120 110 100 90 80 70 60 50 40 30 20 10 0 -10
f1 (ppm)

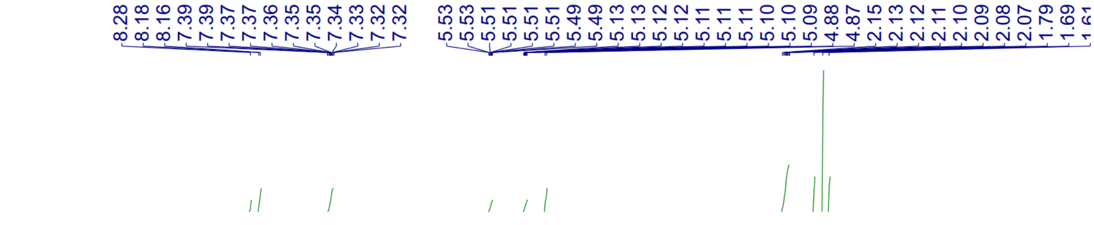

1x

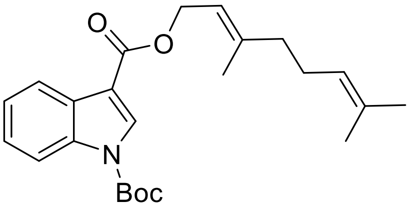

1x

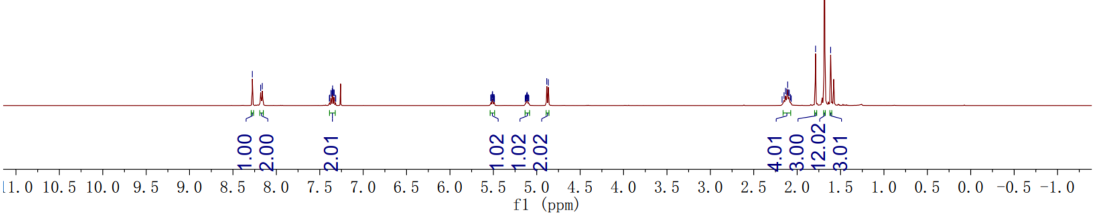

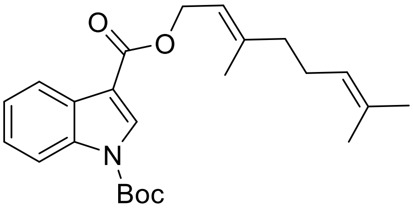

1x

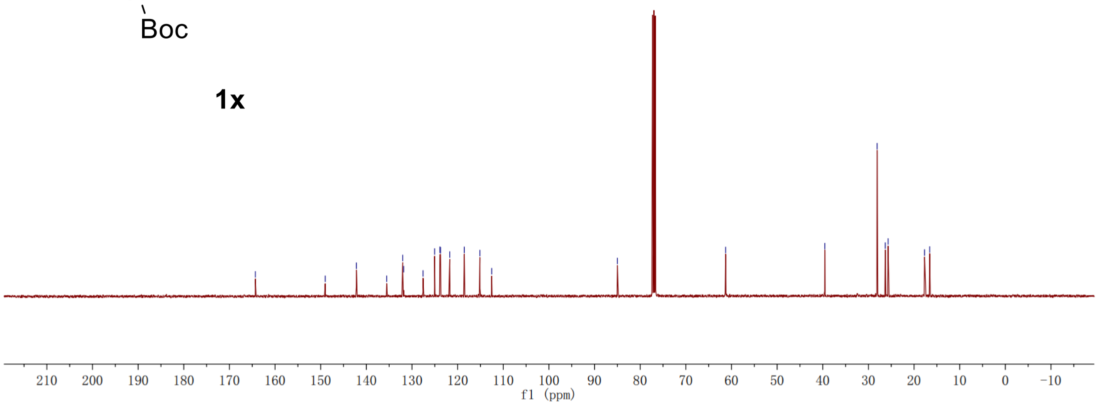

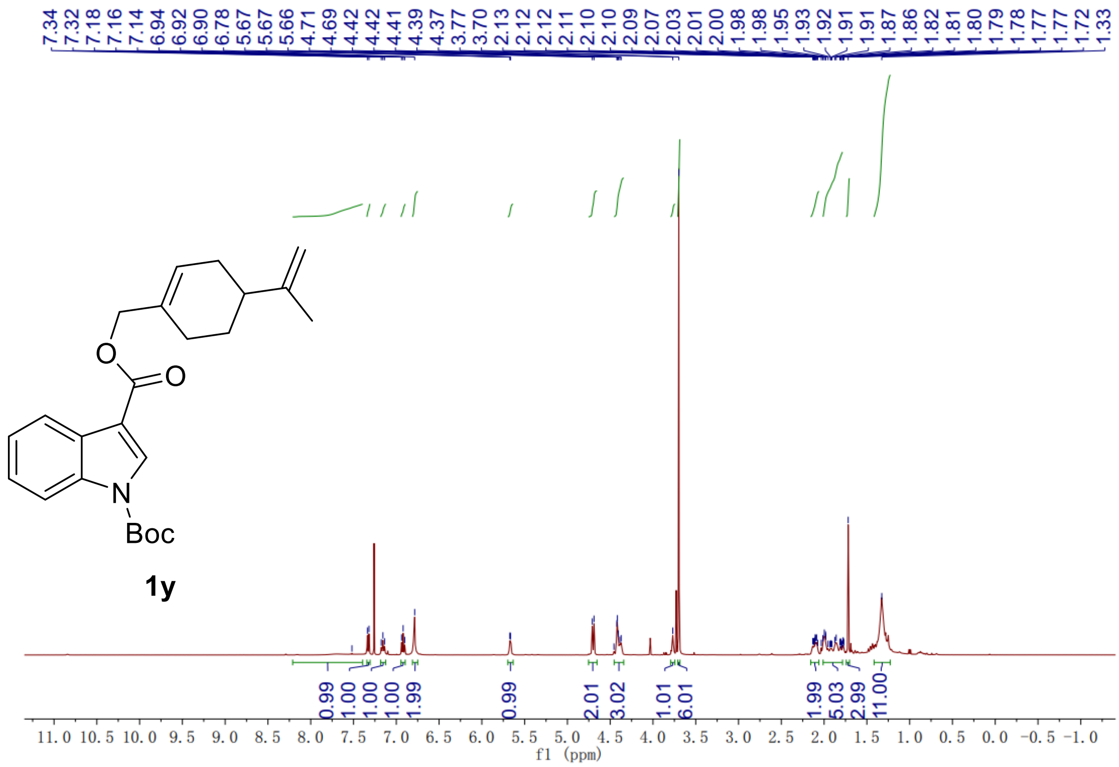
7.34
7.32
7.18
7.16
7.14
6.94
6.92
6.90
6.78
5.67
5.67
5.66
4.71
4.69
4.42
4.42
4.41
4.39
4.37
3.77
3.70
2.13
2.12
2.12
2.11
2.10
2.10
2.09
2.07
2.03
2.01
2.00
1.98
1.98
1.95
1.93
1.92
1.91
1.91
1.87
1.86
1.82
1.81
1.80
1.79
1.78
1.77
1.77
1.72
1.33
O
O
N
Boc
1y
0.99
1.00
1.00
1.00
1.99
0.99
2.01
3.02
1.01
6.01
1.99
5.03
2.99
11.00
11.0 10.5 10.0 9.5 9.0 8.5 8.0 7.5 7.0 6.5 6.0 5.5 5.0 4.5 4.0 3.5 3.0 2.5 2.0 1.5 1.0 0.5 0.0 -0.5 -1.0
f1 (ppm)

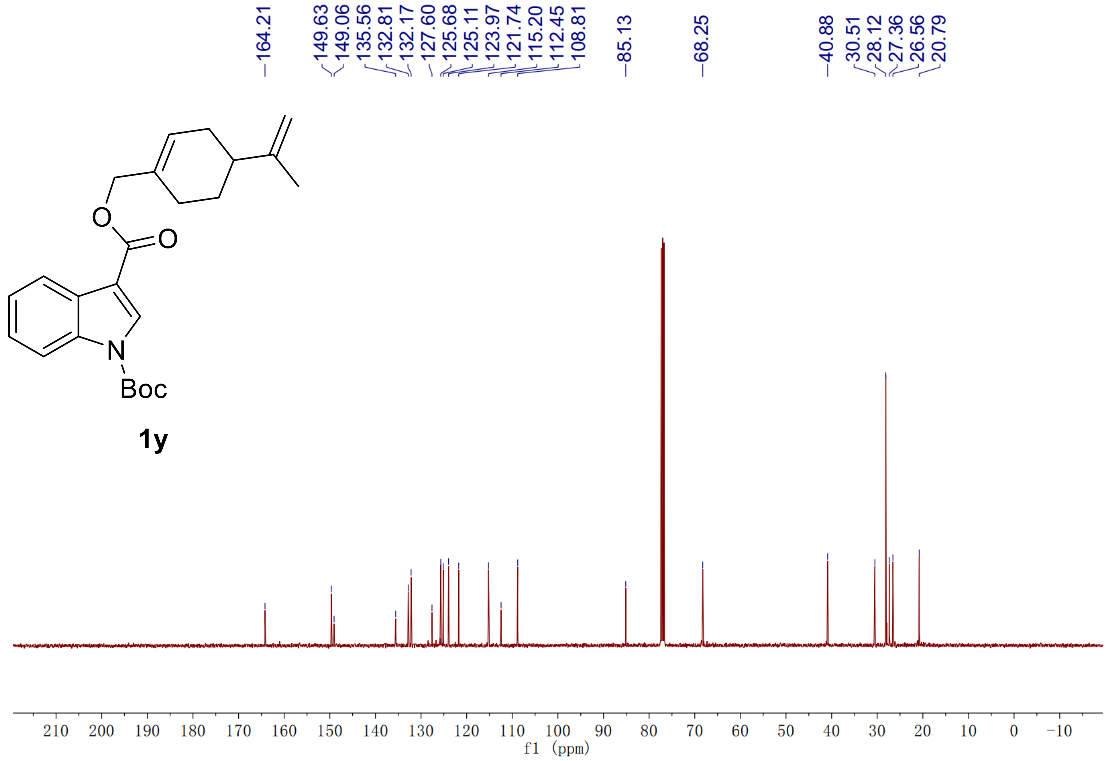
164.21
149.63
149.06
135.56
132.81
132.17
127.60
125.68
125.11
123.97
121.74
115.20
112.45
108.81
85.13
68.25
40.88
30.51
28.12
27.36
26.56
20.79
O
O
N
Boc
1y
210 200 190 180 170 160 150 140 130 120 110 100 90 80 70 60 50 40 30 20 10 0 -10
f1 (ppm)

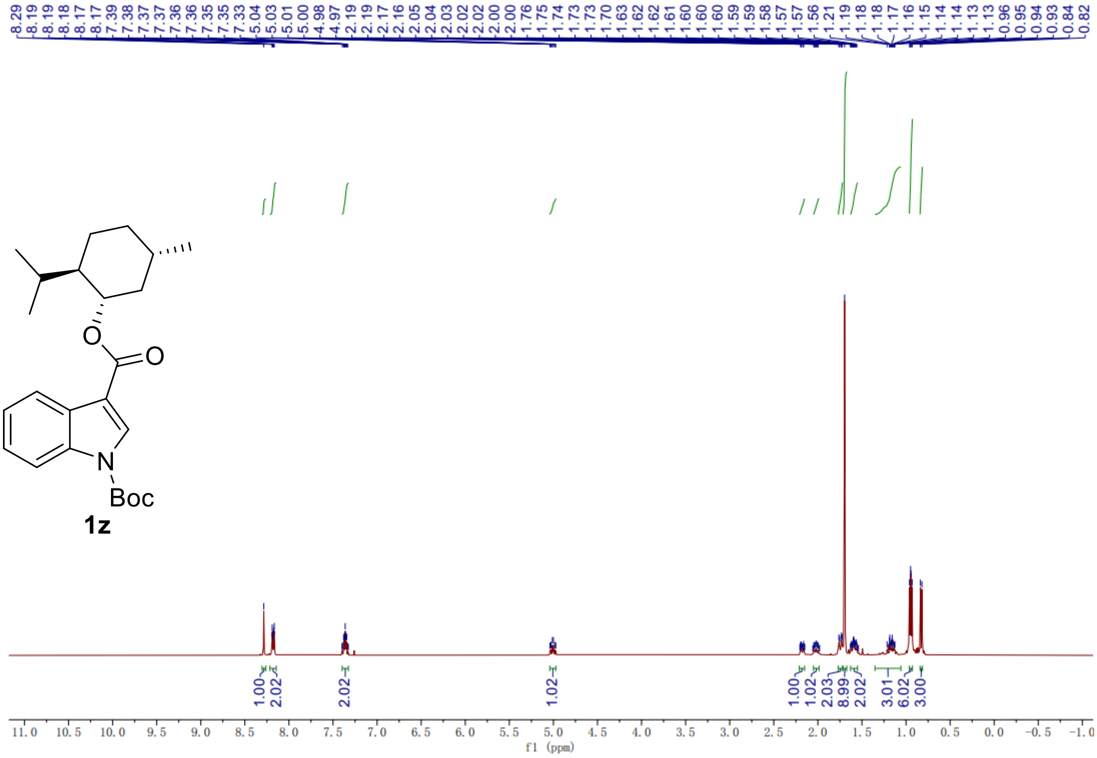
8.29 8.19 8.19 8.18 8.17 8.17 7.39 7.38 7.37 7.37 7.36 7.36 7.35 7.35 7.33 5.04 5.03 5.01 5.00 4.98 4.97 2.19 2.19 2.17 2.16 2.05 2.04 2.03 2.02 2.02 2.00 2.00 1.76 1.75 1.74 1.73 1.73 1.70 1.63 1.62 1.62 1.61 1.60 1.60 1.60 1.59 1.59 1.58 1.57 1.57 1.56 1.21 1.19 1.18 1.18 1.17 1.16 1.15 1.14 1.13 1.13 0.96 0.95 0.94 0.93 0.84 0.82
Boc
1z
1.00 2.02 2.02 1.02 1.00 1.02 2.03 8.99 2.02 3.01 6.02 3.00
11.0 10.5 10.0 9.5 9.0 8.5 8.0 7.5 7.0 6.5 6.0 5.5 5.0 4.5 4.0 3.5 3.0 2.5 2.0 1.5 1.0 0.5 0.0 -0.5 -1.0
f1 (ppm)

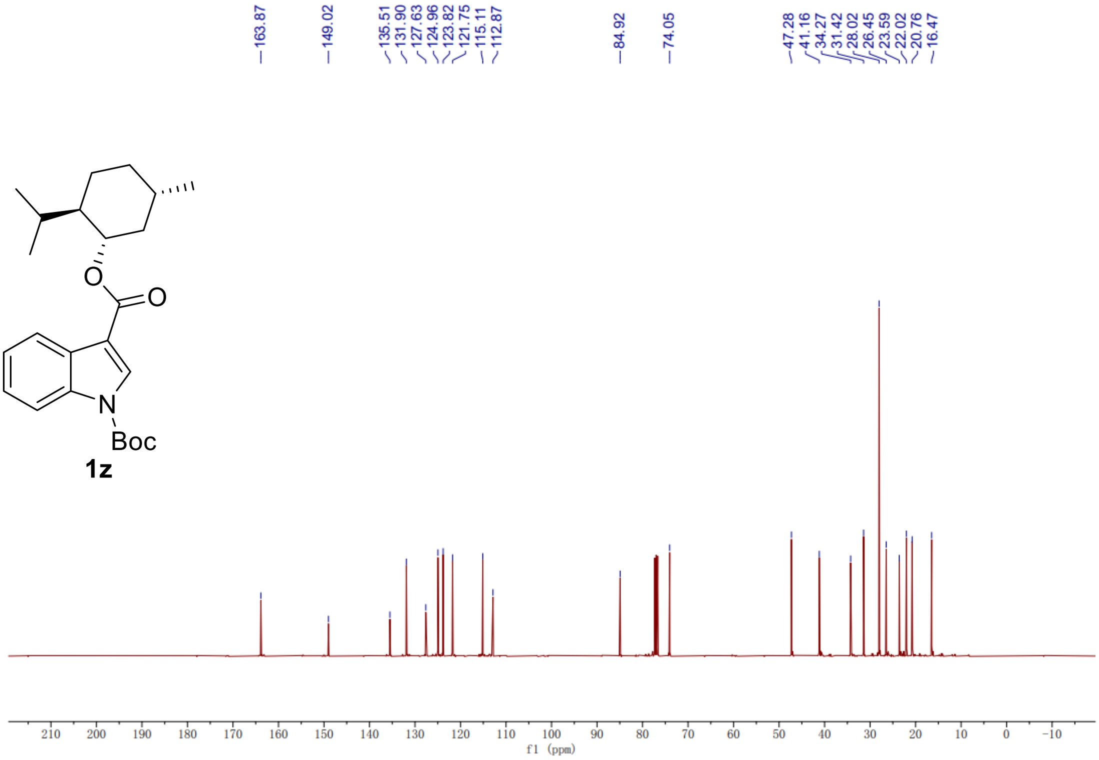
163.87 149.02 135.51 131.90 127.63 124.96 123.82 121.75 115.11 112.87 84.92 74.05 47.28 41.16 34.27 31.42 28.02 26.45 23.59 22.02 20.76 16.47
Boc
1z
210 200 190 180 170 160 150 140 130 120 110 100 90 80 70 60 50 40 30 20 10 0 -10
f1 (ppm)

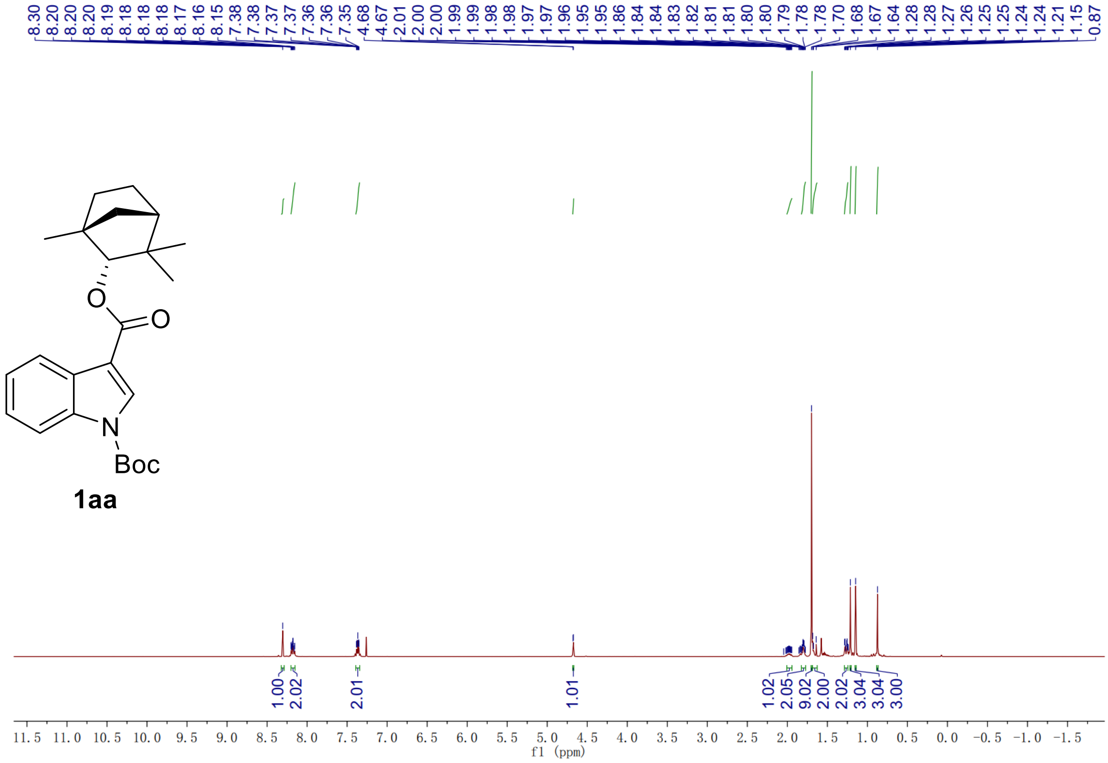

8.30 8.20 8.20 8.20 8.19 8.18 8.18 8.18 8.17 8.16 8.15 7.38 7.38 7.37 7.37 7.36 7.35 4.68 4.67 2.01 2.00 2.00 1.99 1.99 1.98 1.98 1.97 1.97 1.96 1.95 1.95 1.86 1.84 1.84 1.83 1.82 1.81 1.81 1.80 1.80 1.79 1.78 1.78 1.70 1.68 1.67 1.64 1.28 1.28 1.27 1.26 1.25 1.25 1.24 1.24 1.21 1.15 0.87
N
Boc
O
O
1aa
1.00
2.02
2.01
1.01
1.02
2.05
9.02
2.00
2.02
3.04
3.04
3.00
f1 (ppm)

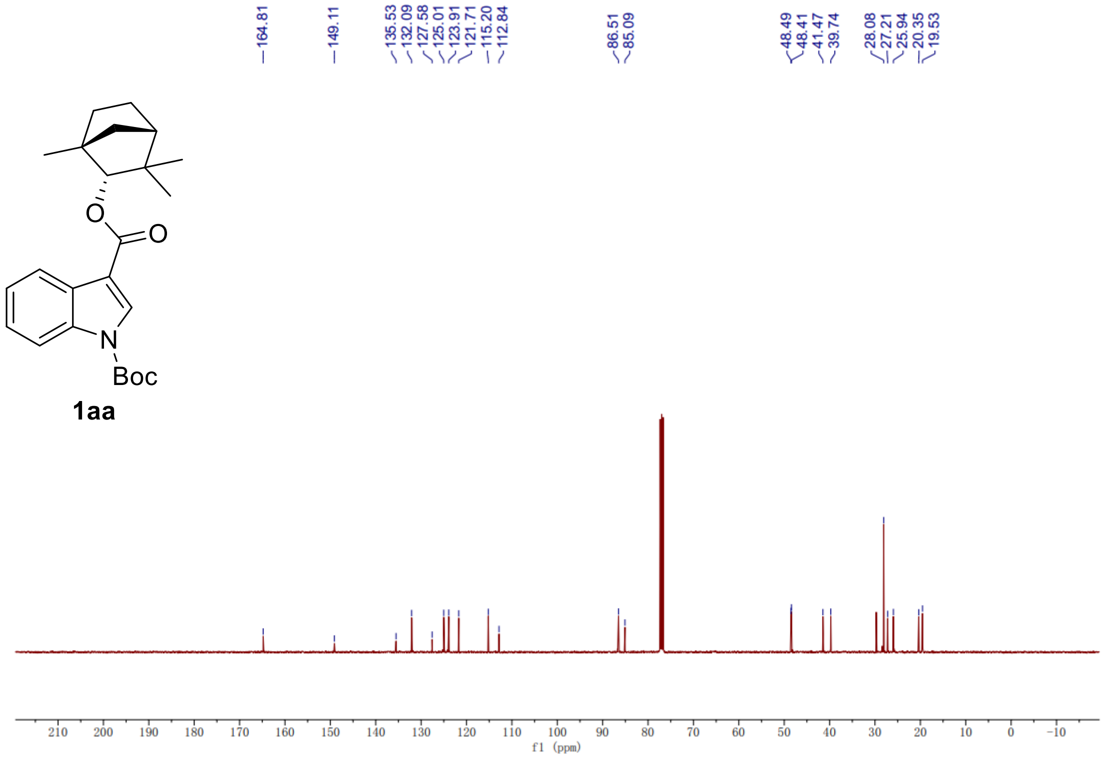

164.81
149.11
135.53 132.09 127.58 125.01 123.91 121.71 115.20 112.84
86.51 85.09
48.49 48.41 41.47 39.74
28.08 27.21 25.94 20.35 19.53
N
Boc
O
O
1aa
f1 (ppm)

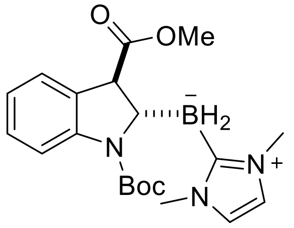
OMe
O
BH2
N
N+
Boc
N

3a

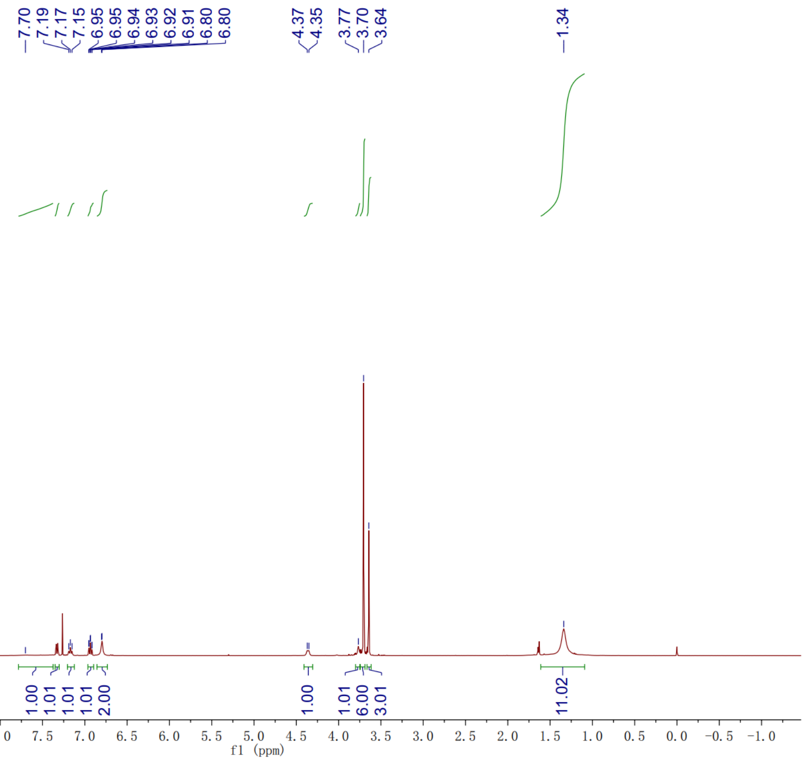
7.70
7.19
7.17
7.15
6.95
6.95
6.94
6.93
6.92
6.91
6.80
6.80
4.37
4.35
3.77
3.70
3.64
1.34
1.00
1.01
1.01
1.01
2.00
1.00
1.01
6.00
3.01
11.02
f1 (ppm)

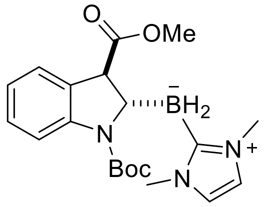
OMe
O
BH2
N
N+
Boc
N

3a

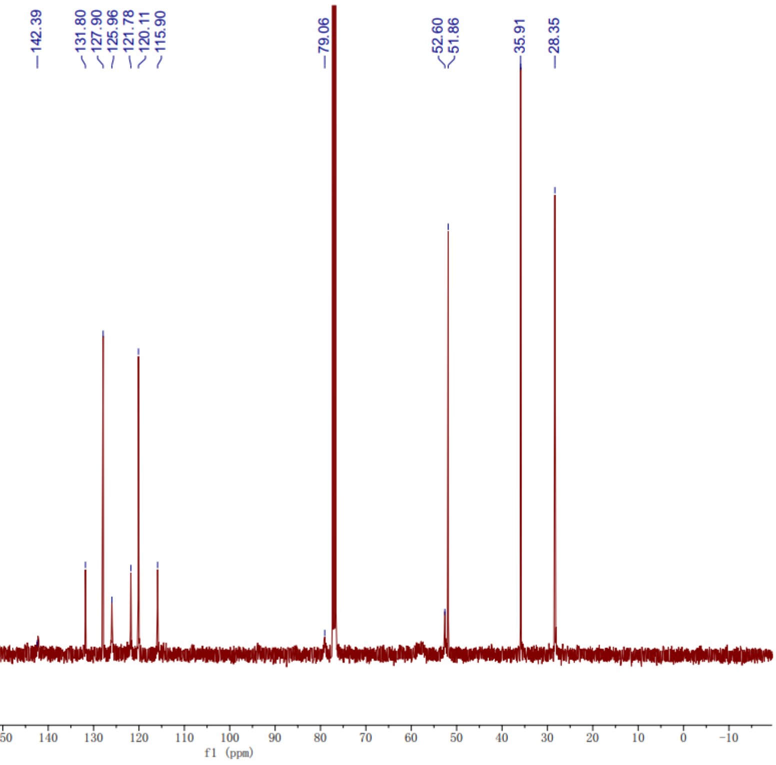
174.55
152.03
142.39
131.80
127.90
125.96
121.78
120.11
115.90
79.06
52.60
51.86
35.91
28.35
f1 (ppm)

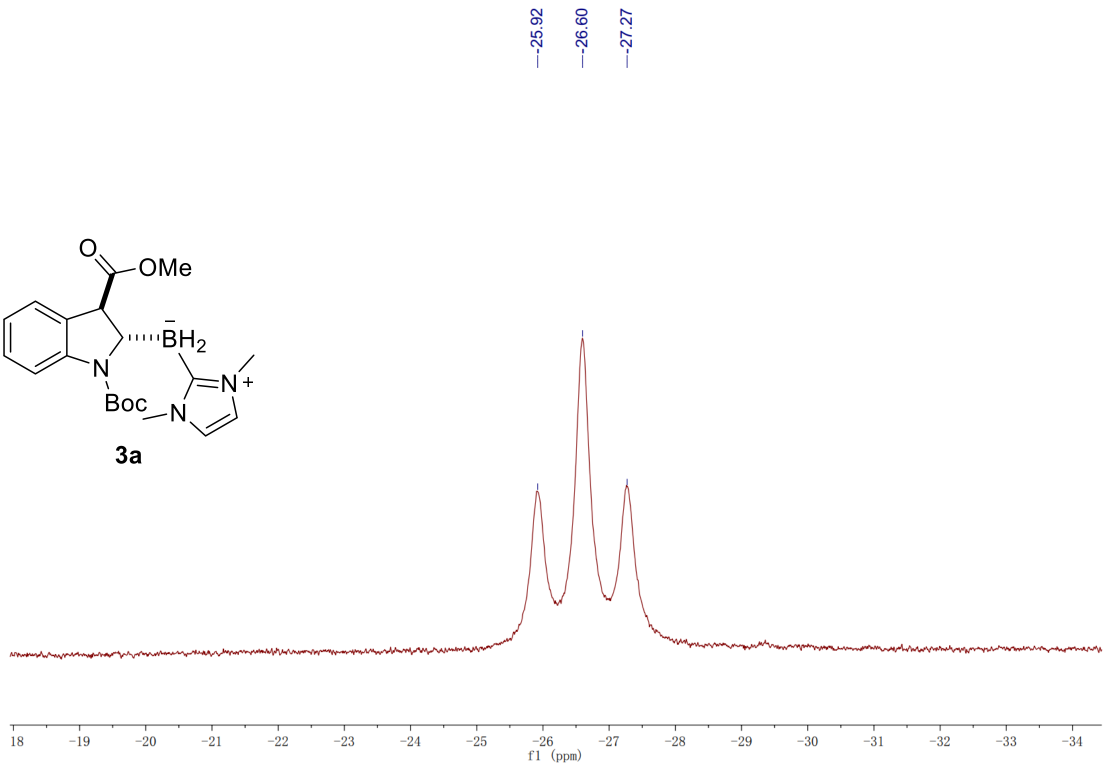
-25.92
-26.60
-27.27
OMe
O
BH2
N
Boc
N+
N
3a
18 −19 −20 −21 −22 −23 −24 −25 −26 −27 −28 −29 −30 −31 −32 −33 −34
f1 (ppm)

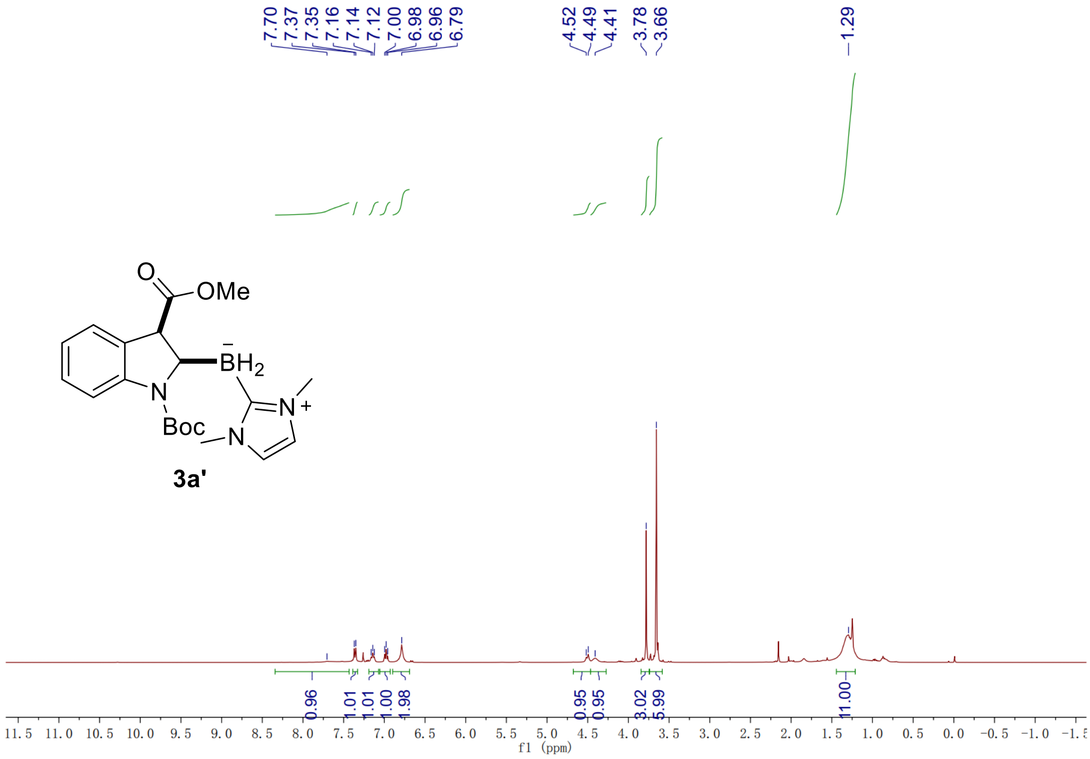
7.70
7.37
7.35
7.16
7.14
7.12
7.00
6.98
6.96
6.79
4.52
4.49
4.41
3.78
3.66
1.29
O
OMe
BH2
N
Boc
N+
N
3a'
0.96
1.01
1.01
1.00
1.98
0.95
0.95
3.02
5.99
11.00
11.5 11.0 10.5 10.0 9.5 9.0 8.5 8.0 7.5 7.0 6.5 6.0 5.5 5.0 4.5 4.0 3.5 3.0 2.5 2.0 1.5 1.0 0.5 0.0 −0.5 −1.0
f1 (ppm)

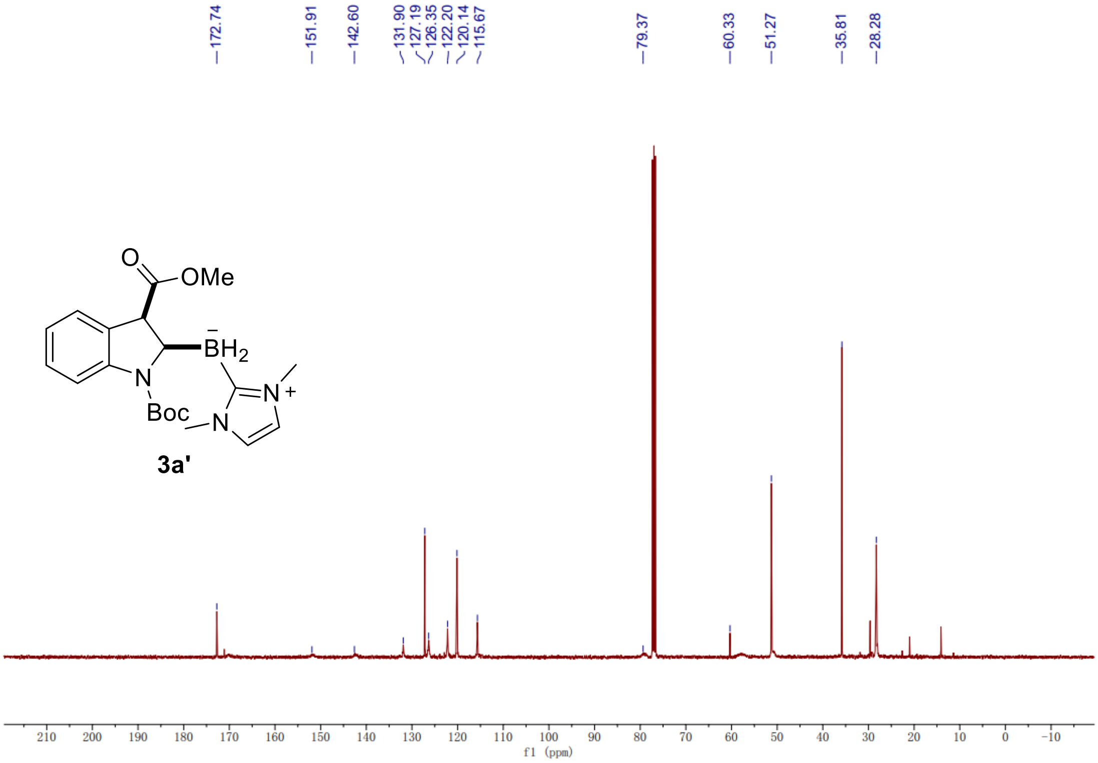
172.74
151.91
142.60
131.90
127.19
126.35
122.20
120.14
115.67
79.37
60.33
51.27
35.81
28.28
OMe
O
BH2
N
Boc
N
N+
3a'
210 200 190 180 170 160 150 140 130 120 110 100 90 80 70 60 50 40 30 20 10 0 -10
f1 (ppm)

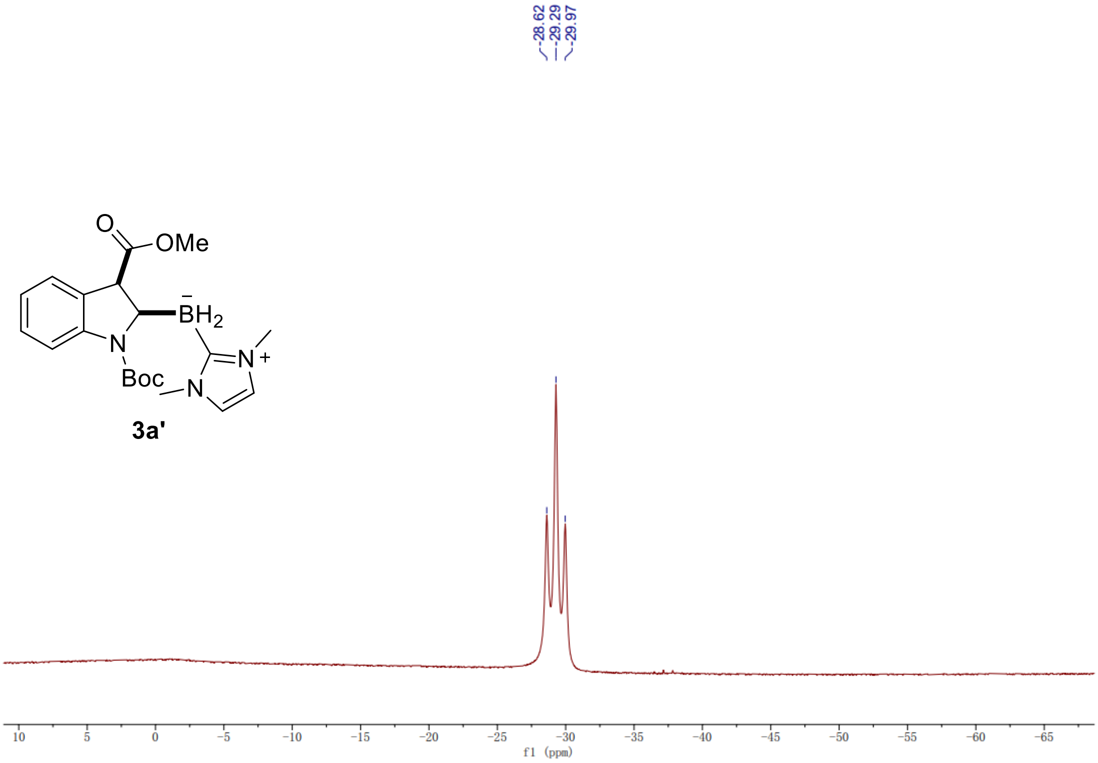
-28.62
-29.29
-29.97
OMe
O
BH2
N
Boc
N
N+
3a'
10 5 0 -5 -10 -15 -20 -25 -30 -35 -40 -45 -50 -55 -60 -65
f1 (ppm)

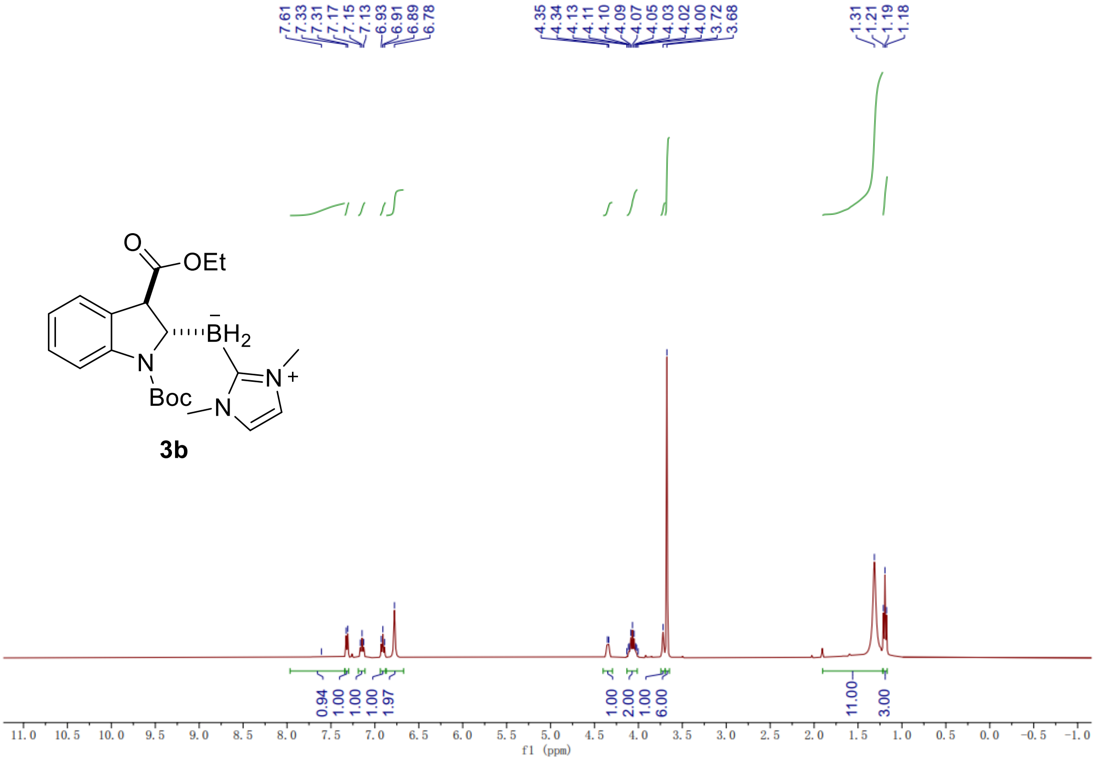
7.61
7.33
7.31
7.17
7.15
7.13
6.93
6.91
6.89
6.78
4.35
4.34
4.13
4.11
4.10
4.09
4.07
4.05
4.03
4.02
4.00
3.72
3.68
1.31
1.21
1.19
1.18
O
OEt
BH2
N
N+
Boc
N
0.94
1.00
1.00
1.00
1.97
1.00
2.00
1.00
6.00
11.00
3.00
11.0 10.5 10.0 9.5 9.0 8.5 8.0 7.5 7.0 6.5 6.0 5.5 5.0 4.5 4.0 3.5 3.0 2.5 2.0 1.5 1.0 0.5 0.0 -0.5 -1.0
f1 (ppm)

3b

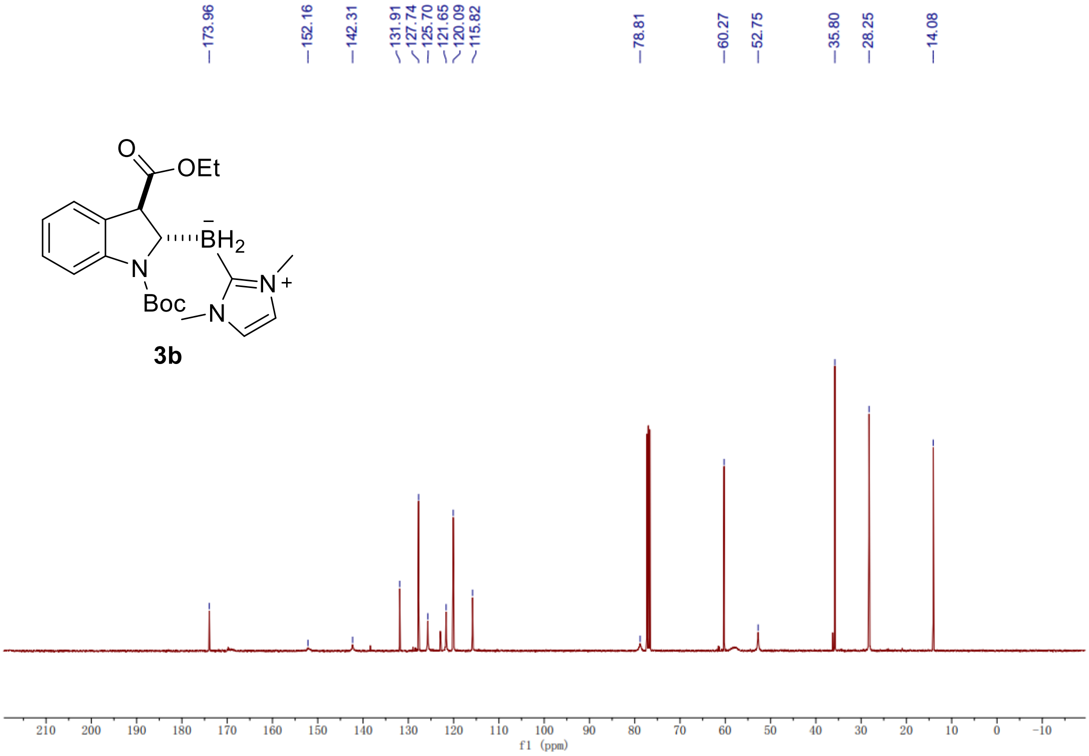
173.96
152.16
142.31
131.91
127.74
125.70
121.65
120.09
115.82
78.81
60.27
52.75
35.80
28.25
14.08
O
OEt
BH2
N
N+
Boc
N
210 200 190 180 170 160 150 140 130 120 110 100 90 80 70 60 50 40 30 20 10 0 -10
f1 (ppm)

3b

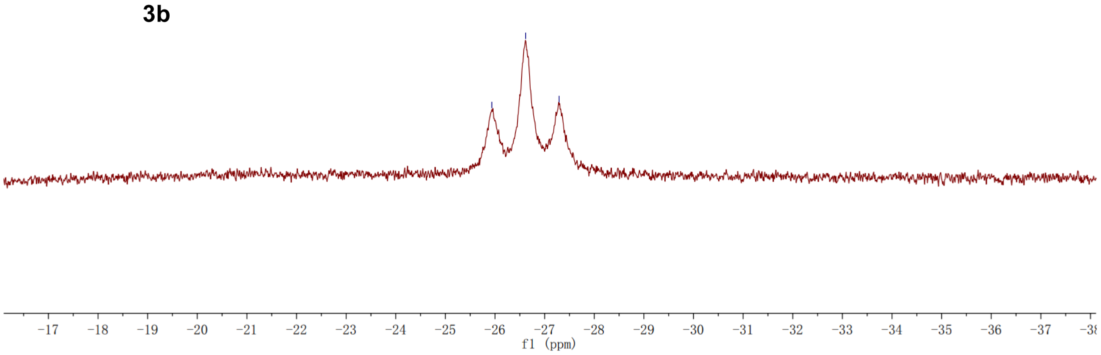
-25.94
-26.62
-27.29
f1 (ppm)

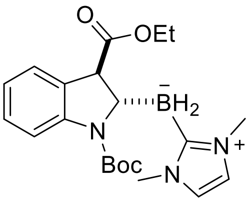
O
OEt
BH2
N
Boc
N
N+

3b

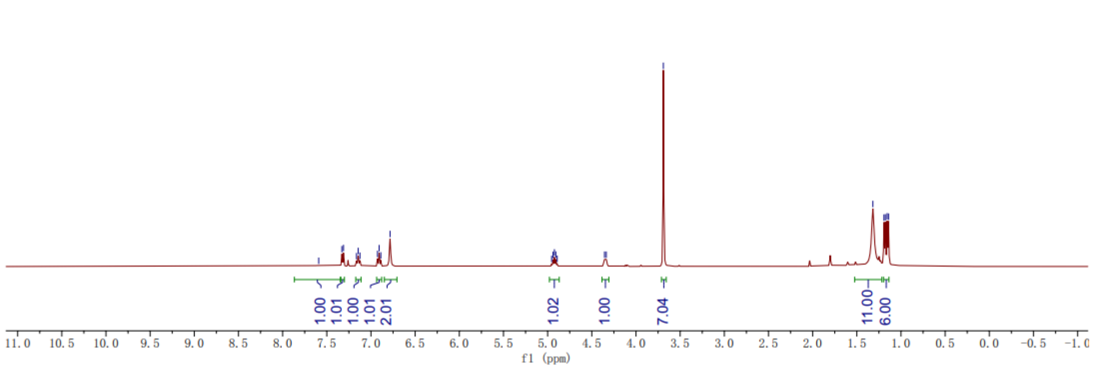
7.59
7.33
7.31
7.16
7.14
7.13
6.93
6.91
6.89
6.78
4.95
4.94
4.92
4.91
4.89
4.36
4.34
3.69
1.32
1.19
1.18
1.16
1.14
1.00
1.01
1.00
1.01
2.01
1.02
1.00
7.04
11.00
6.00
f1 (ppm)

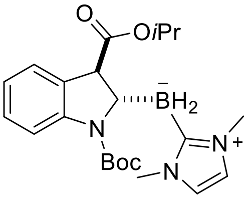
O
OiPr
BH2
N
Boc
N
N+

3c

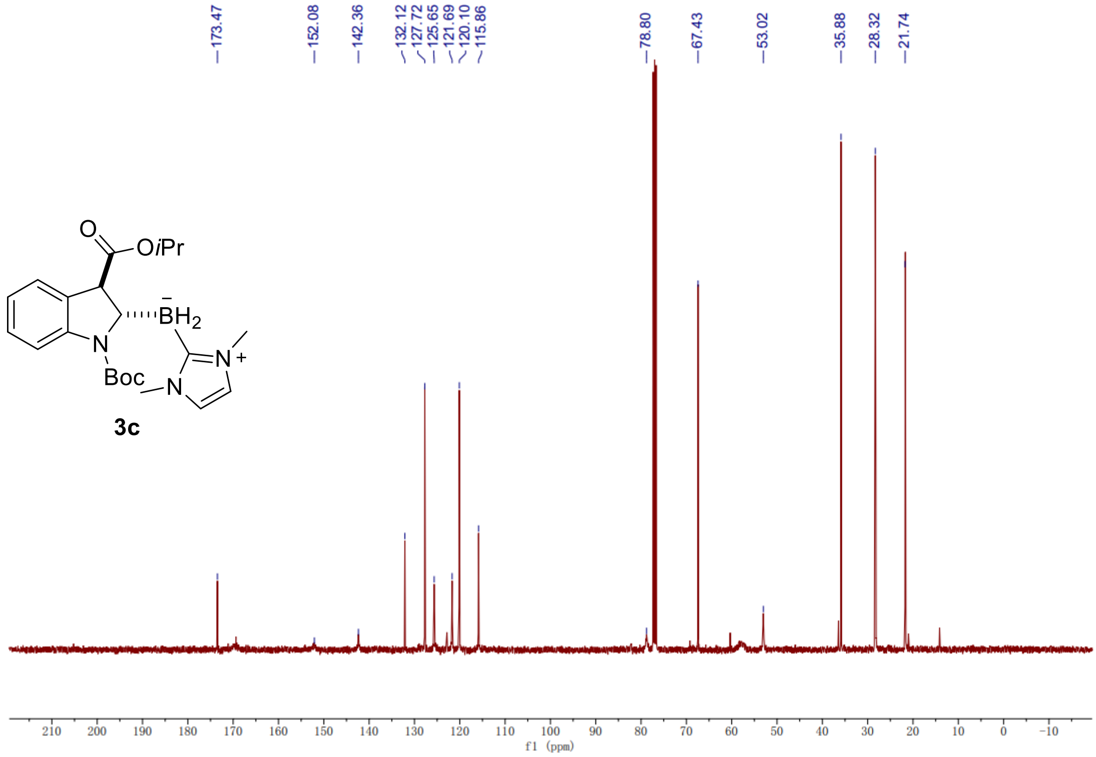

173.47
152.08
142.36
132.12
127.72
125.65
121.69
120.10
115.86
78.80
67.43
53.02
35.88
28.32
21.74
O
OiPr
BH2
N
Boc
N+
N
3c
210 200 190 180 170 160 150 140 130 120 110 100 90 80 70 60 50 40 30 20 10 0 -10
f1 (ppm)

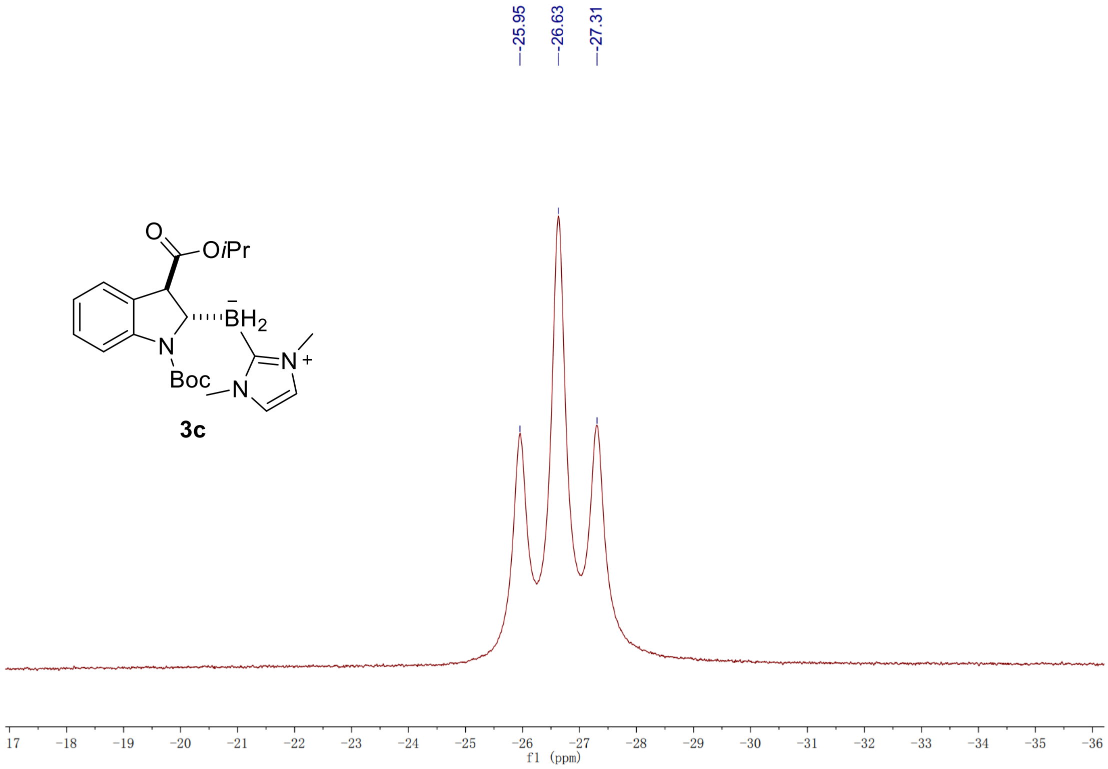

-25.95
-26.63
-27.31
O
OiPr
BH2
N
Boc
N+
N
3c
-17 -18 -19 -20 -21 -22 -23 -24 -25 -26 -27 -28 -29 -30 -31 -32 -33 -34 -35 -36
f1 (ppm)

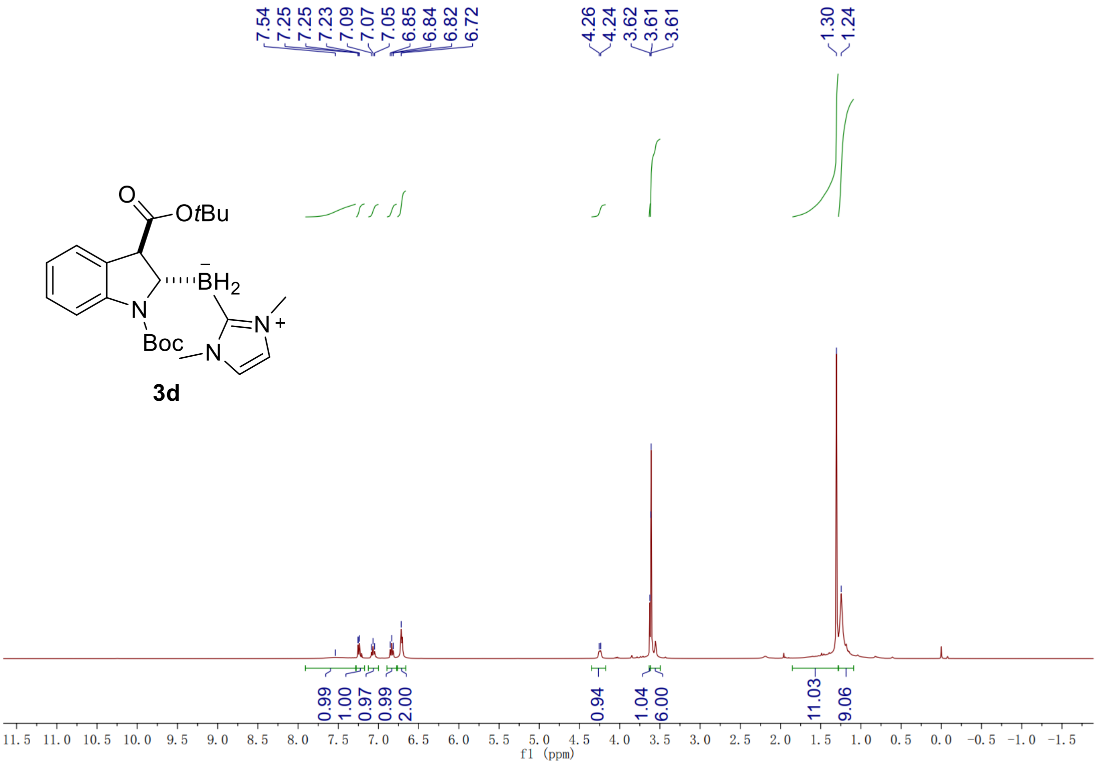

7.54
7.25
7.25
7.23
7.09
7.07
7.05
6.85
6.84
6.82
6.72
4.26
4.24
3.62
3.61
3.61
1.30
1.24
OtBu
O
BH2
N
Boc
N
N+
3d
0.99
1.00
0.97
0.99
2.00
0.94
1.04
6.00
11.03
9.06
11.5 11.0 10.5 10.0 9.5 9.0 8.5 8.0 7.5 7.0 6.5 6.0 5.5 5.0 4.5 4.0 3.5 3.0 2.5 2.0 1.5 1.0 0.5 0.0 -0.5 -1.0 -1.5
f1 (ppm)

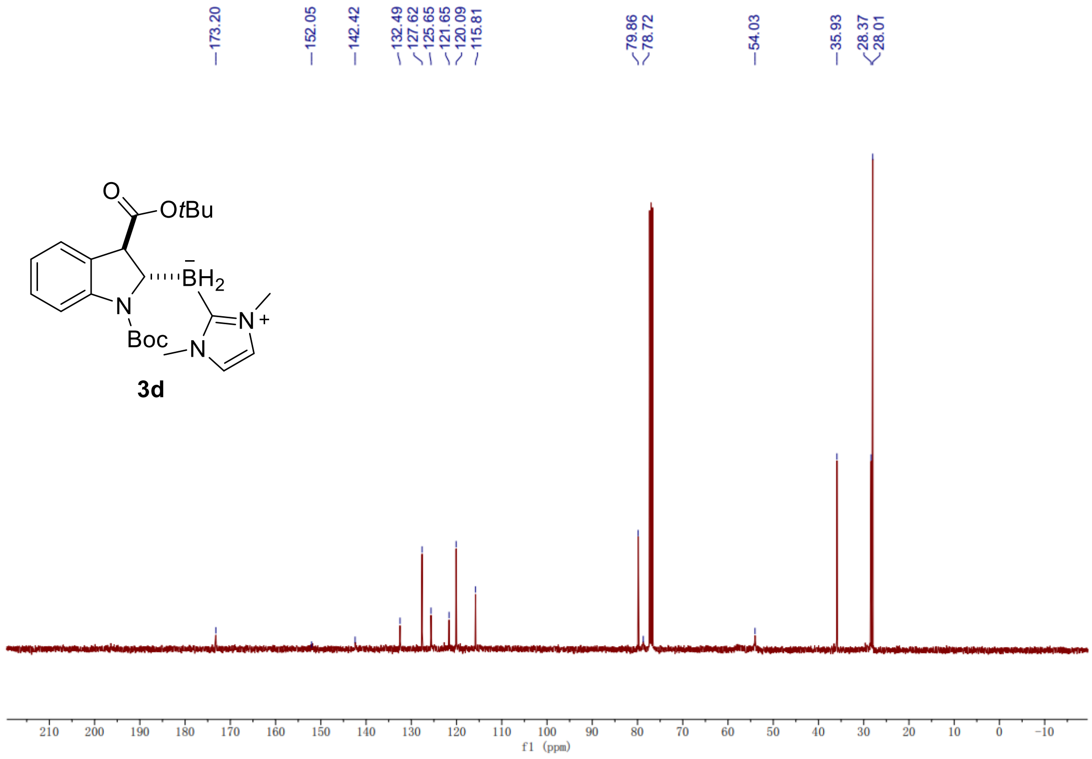

173.20
152.05
142.42
132.49
127.62
125.65
121.65
120.09
115.81
79.86
78.72
54.03
35.93
28.37
28.01
OtBu
O
BH2
N
Boc
N
N+
3d
210 200 190 180 170 160 150 140 130 120 110 100 90 80 70 60 50 40 30 20 10 0 -10
f1 (ppm)

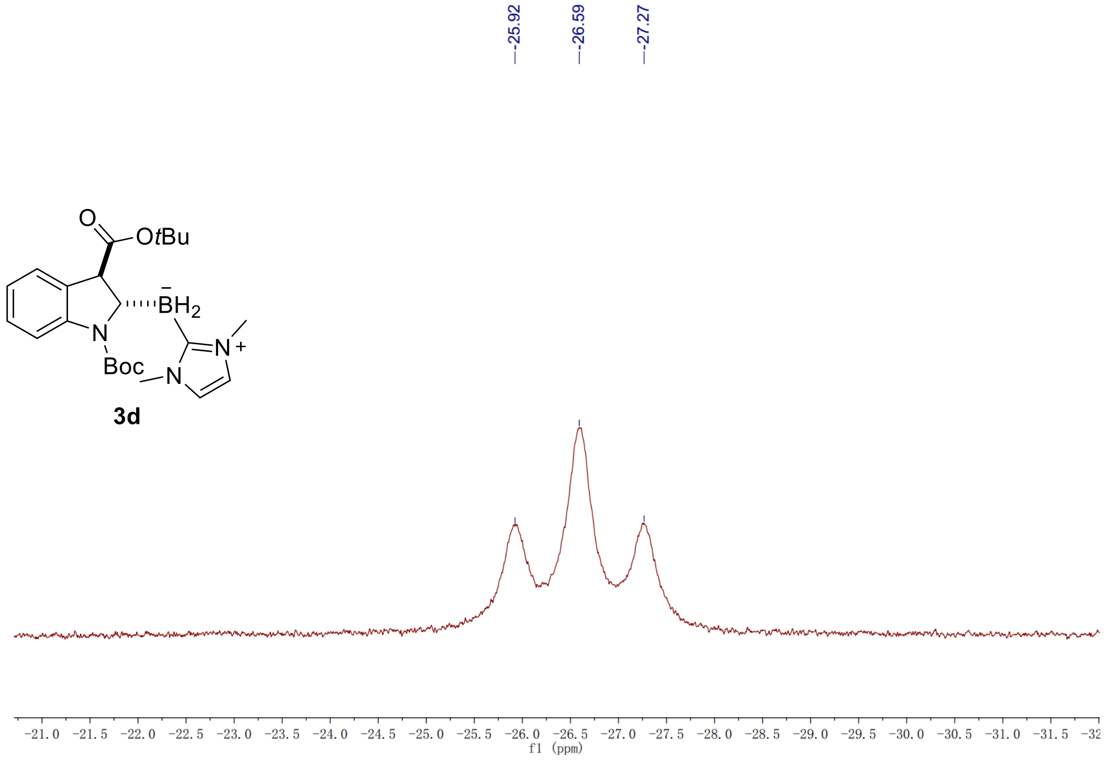
-25.92
-26.59
-27.27
-21.0 -21.5 -22.0 -22.5 -23.0 -23.5 -24.0 -24.5 -25.0 -25.5 -26.0 -26.5 -27.0 -27.5 -28.0 -28.5 -29.0 -29.5 -30.0 -30.5 -31.0 -31.5 -32
f1 (ppm)
O
OtBu
BH2
N
Boc
N+
N

3d

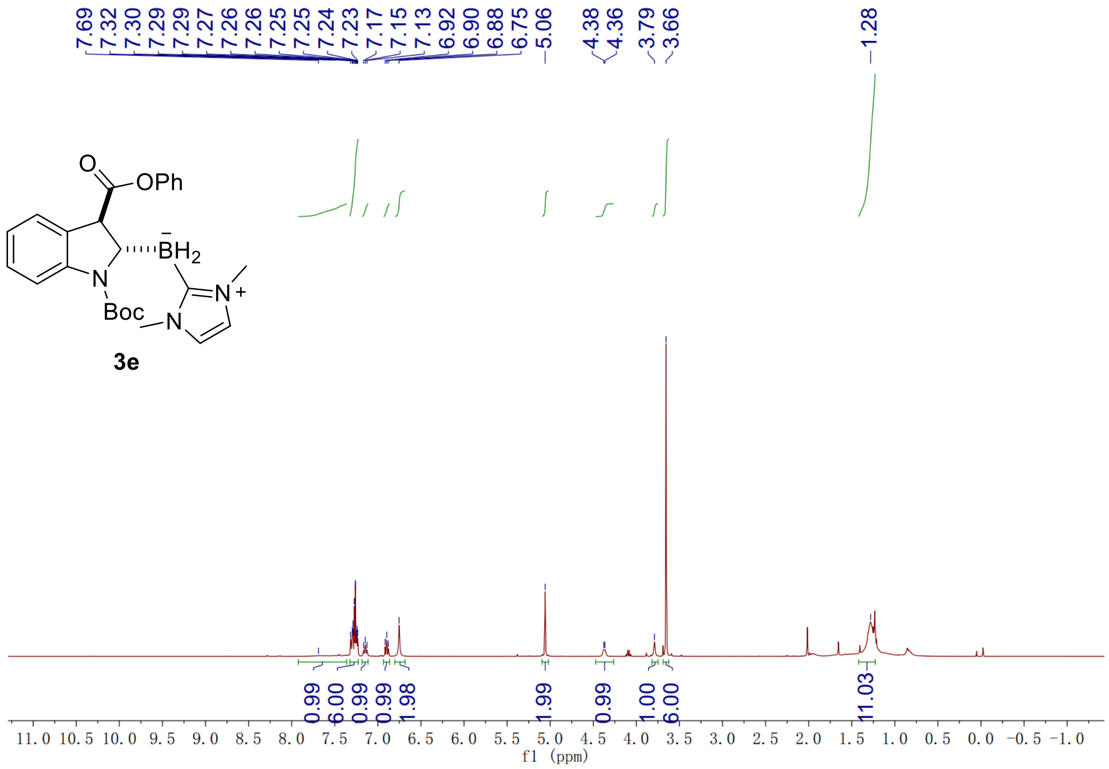
7.69 7.32 7.30 7.29 7.29 7.27 7.26 7.26 7.25 7.25 7.24 7.23 7.17 7.15 7.13 6.92 6.90 6.88 6.75 5.06 4.38 4.36 3.79 3.66 1.28
0.99 6.00 0.99 0.99 1.98 1.99 0.99 1.00 6.00 11.03
11.0 10.5 10.0 9.5 9.0 8.5 8.0 7.5 7.0 6.5 6.0 5.5 5.0 4.5 4.0 3.5 3.0 2.5 2.0 1.5 1.0 0.5 0.0 -0.5 -1.0
f1 (ppm)
O
OPh
BH2
N
Boc
N+
N

3e

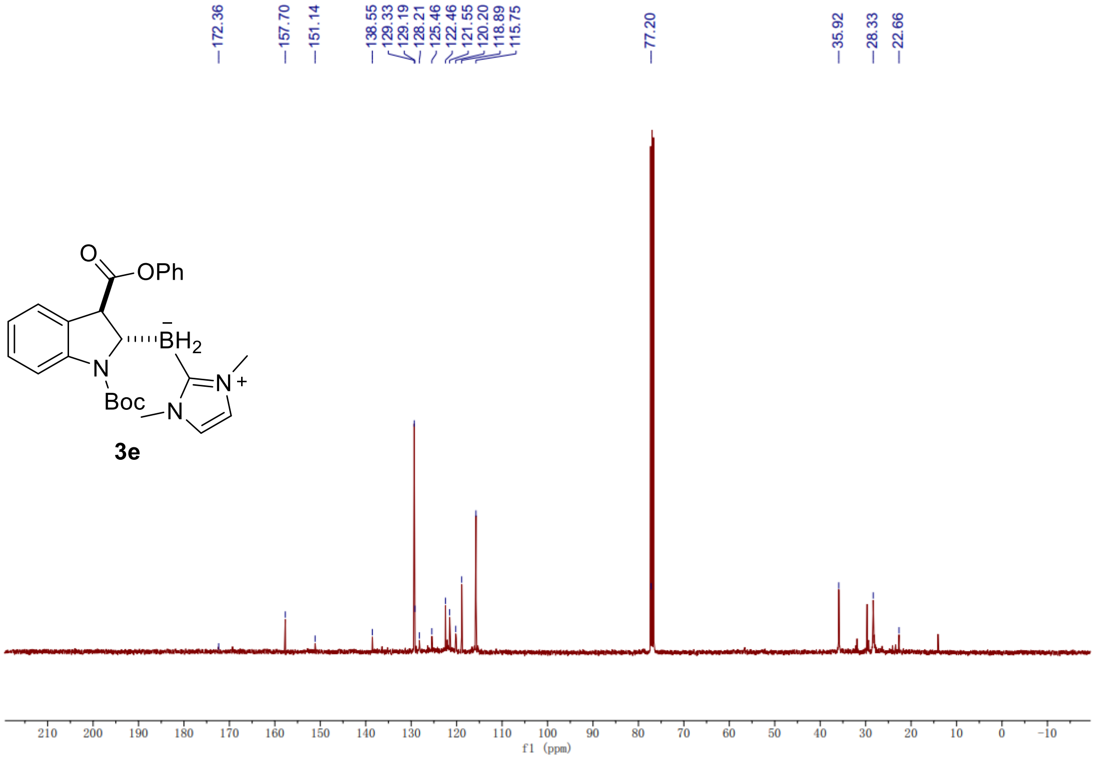
172.36
157.70
151.14
138.55
129.33
129.19
128.21
125.46
122.46
121.55
120.20
118.89
115.75
77.20
35.92
28.33
22.66
O
OPh
BH2
N
Boc
N
N+
3e
210 200 190 180 170 160 150 140 130 120 110 100 90 80 70 60 50 40 30 20 10 0 -10
f1 (ppm)

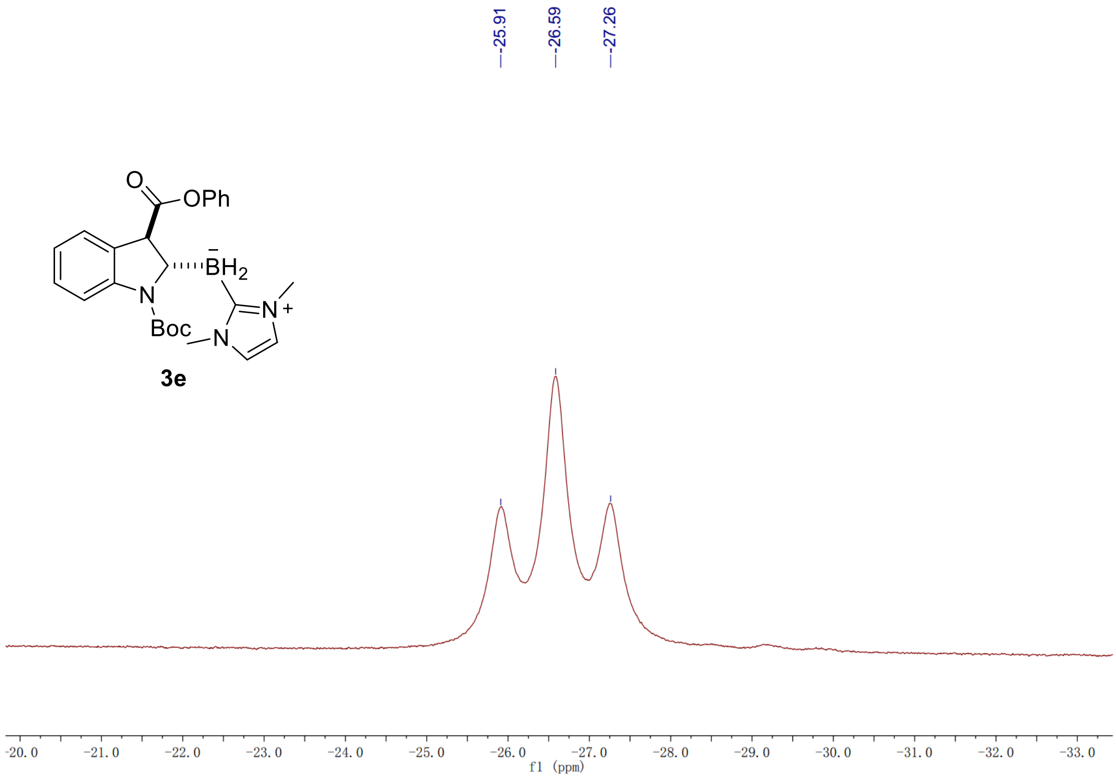
-25.91
-26.59
-27.26
O
OPh
BH2
N
Boc
N
N+
3e
-20.0 -21.0 -22.0 -23.0 -24.0 -25.0 -26.0 -27.0 -28.0 -29.0 -30.0 -31.0 -32.0 -33.0
f1 (ppm)

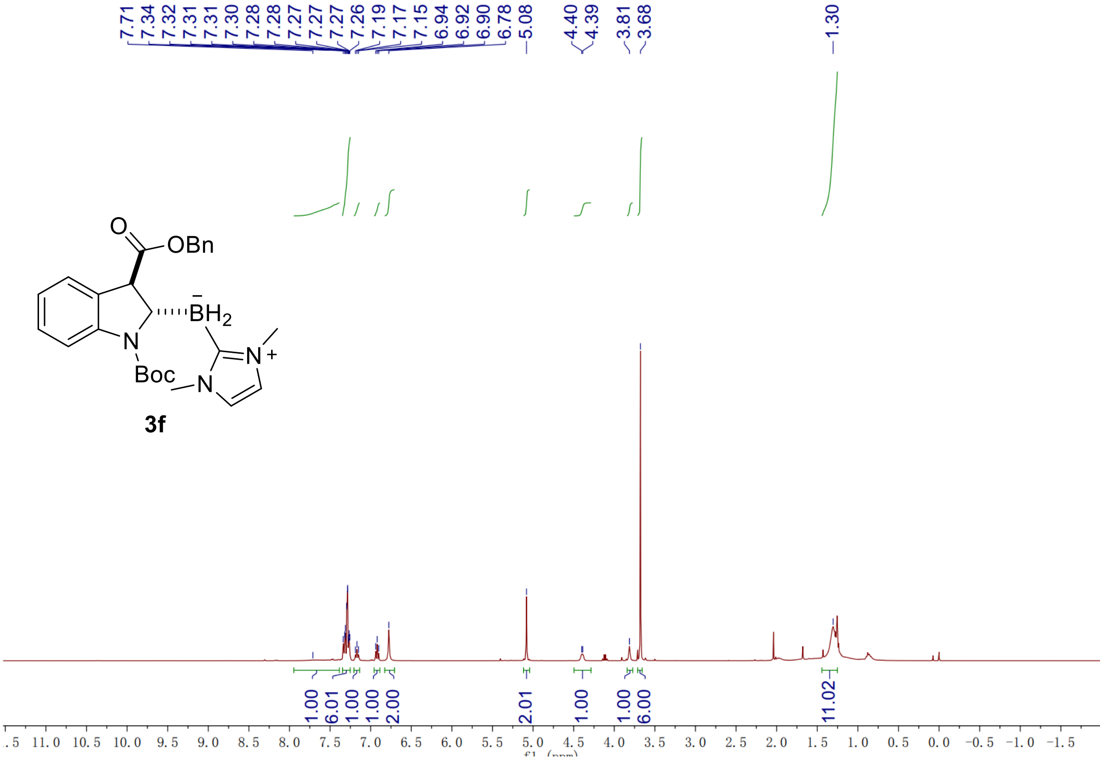

7.71
7.34
7.32
7.31
7.30
7.28
7.28
7.27
7.27
7.26
7.19
7.17
7.15
6.94
6.92
6.90
6.78
5.08
4.40
4.39
3.81
3.68
1.30
1.00
6.01
1.00
1.00
2.00
2.01
1.00
1.00
6.00
11.02
f1 (ppm)
O
OBn
BH2
N
Boc
N+
N

3f

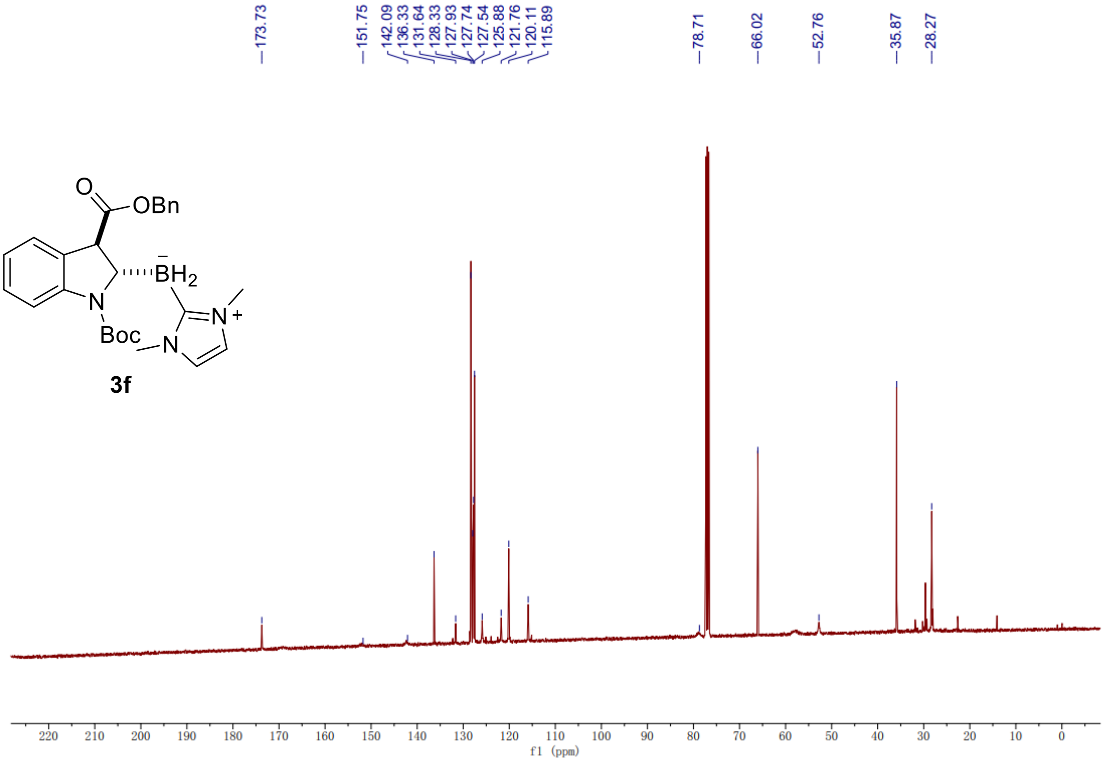

173.73
151.75
142.09
136.33
131.64
128.33
127.93
127.74
127.54
125.88
121.76
120.11
115.89
78.71
66.02
52.76
35.87
28.27
f1 (ppm)
O
OBn
BH2
N
Boc
N+
N

3f

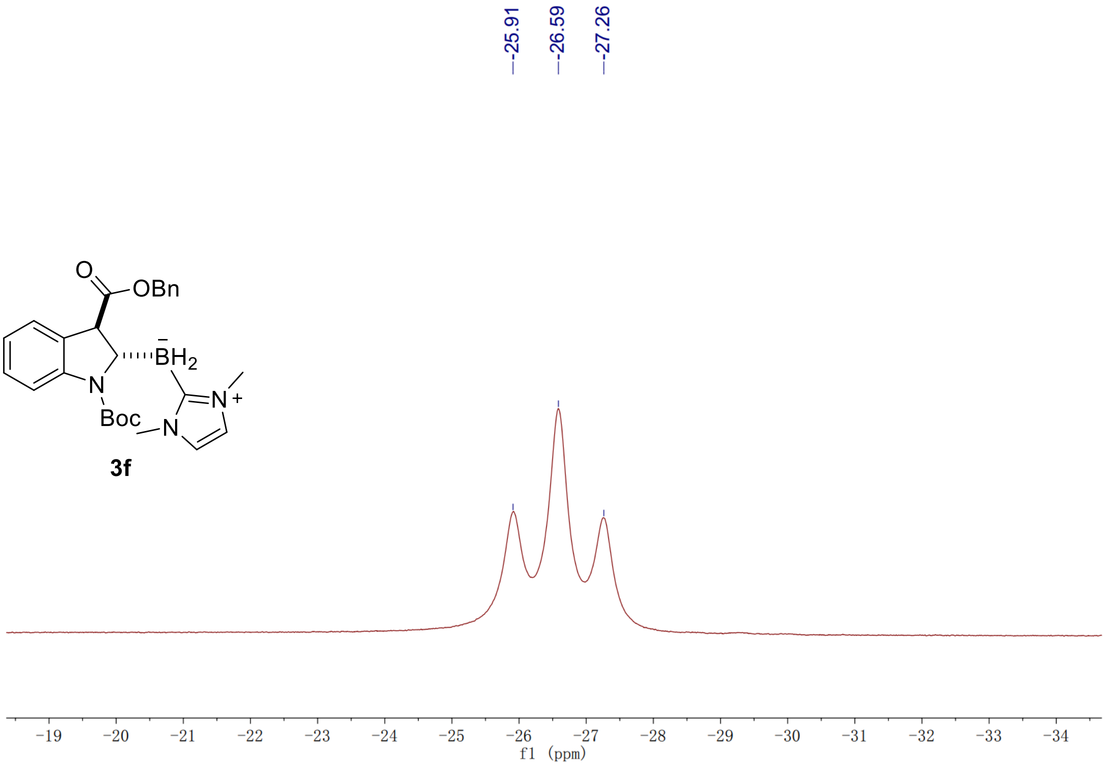

O
OBn
BH2
N
Boc
N+
N
-25.91
-26.59
-27.26
-19 -20 -21 -22 -23 -24 -25 -26 -27 -28 -29 -30 -31 -32 -33 -34
f1 (ppm)

3f

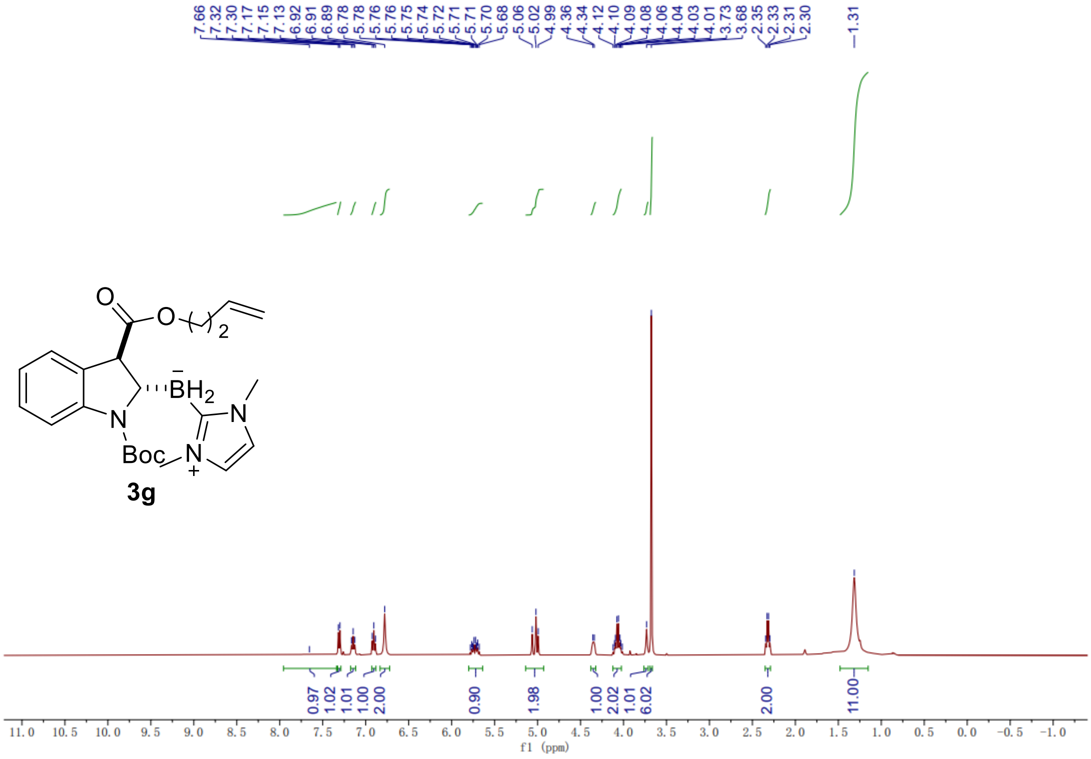

O
O
2
BH2
N
Boc
N
N+
7.66 7.32 7.30 7.17 7.15 7.13 6.92 6.91 6.89 6.78 5.78 5.76 5.75 5.74 5.72 5.71 5.71 5.70 5.68 5.06 5.02 4.99 4.36 4.34 4.12 4.10 4.09 4.08 4.06 4.04 4.03 4.01 3.73 3.68 2.35 2.33 2.31 2.30
1.31
0.97 1.02 1.01 1.00 2.00 0.90 1.98 1.00 2.02 1.01 6.02 2.00 11.00
11.0 10.5 10.0 9.5 9.0 8.5 8.0 7.5 7.0 6.5 6.0 5.5 5.0 4.5 4.0 3.5 3.0 2.5 2.0 1.5 1.0 0.5 0.0 -0.5 -1.0
f1 (ppm)

3g

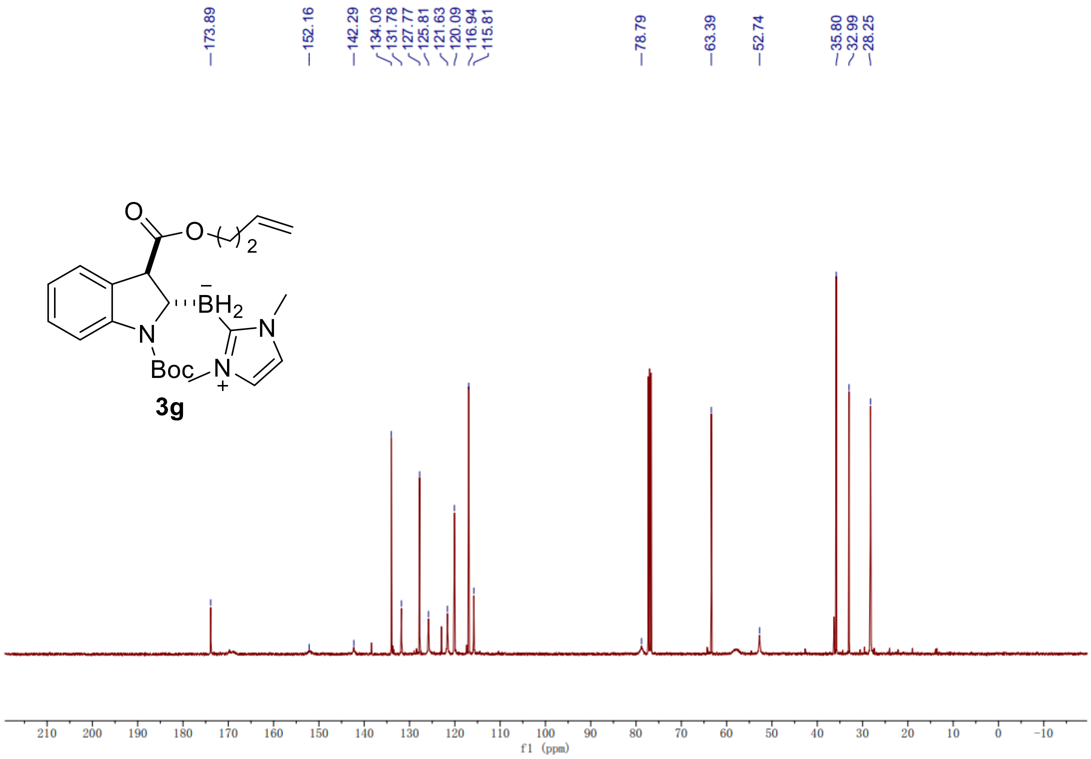
173.89
152.16
142.29
134.03
131.78
127.77
125.81
121.63
120.09
116.94
115.81
78.79
63.39
52.74
35.80
32.99
28.25
3g
210 200 190 180 170 160 150 140 130 120 110 100 90 80 70 60 50 40 30 20 10 0 -10
f1 (ppm)

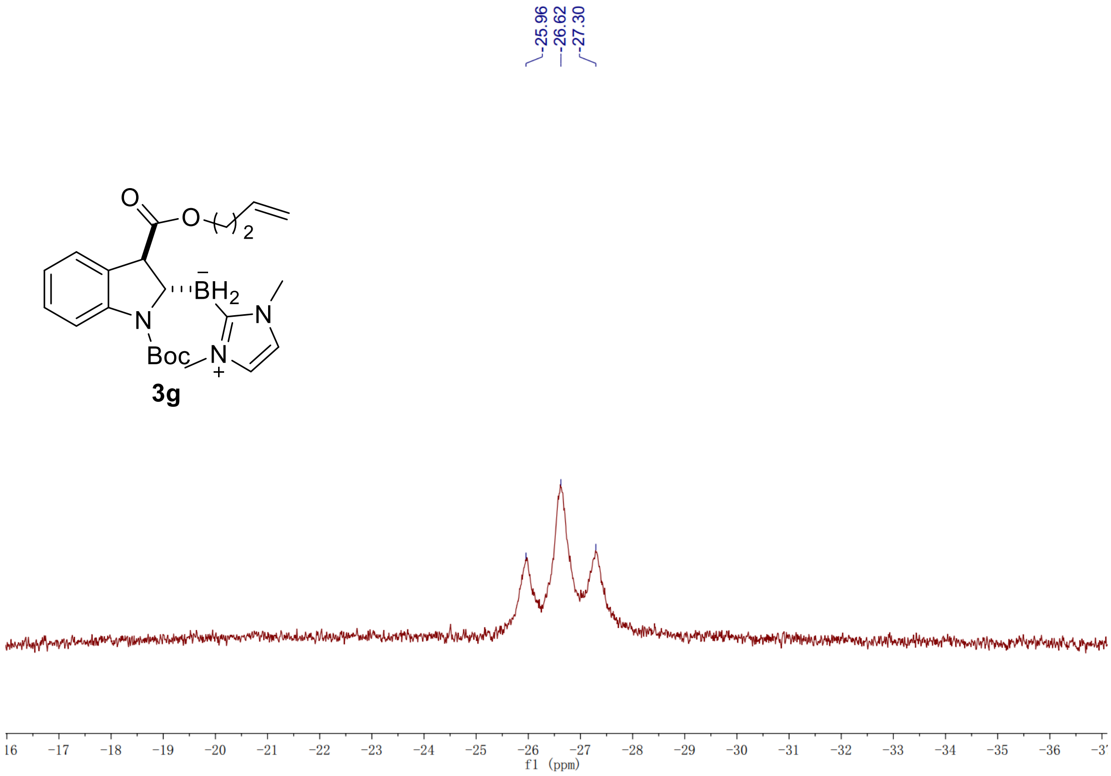
-25.96
-26.62
-27.30
3g
f1 (ppm)

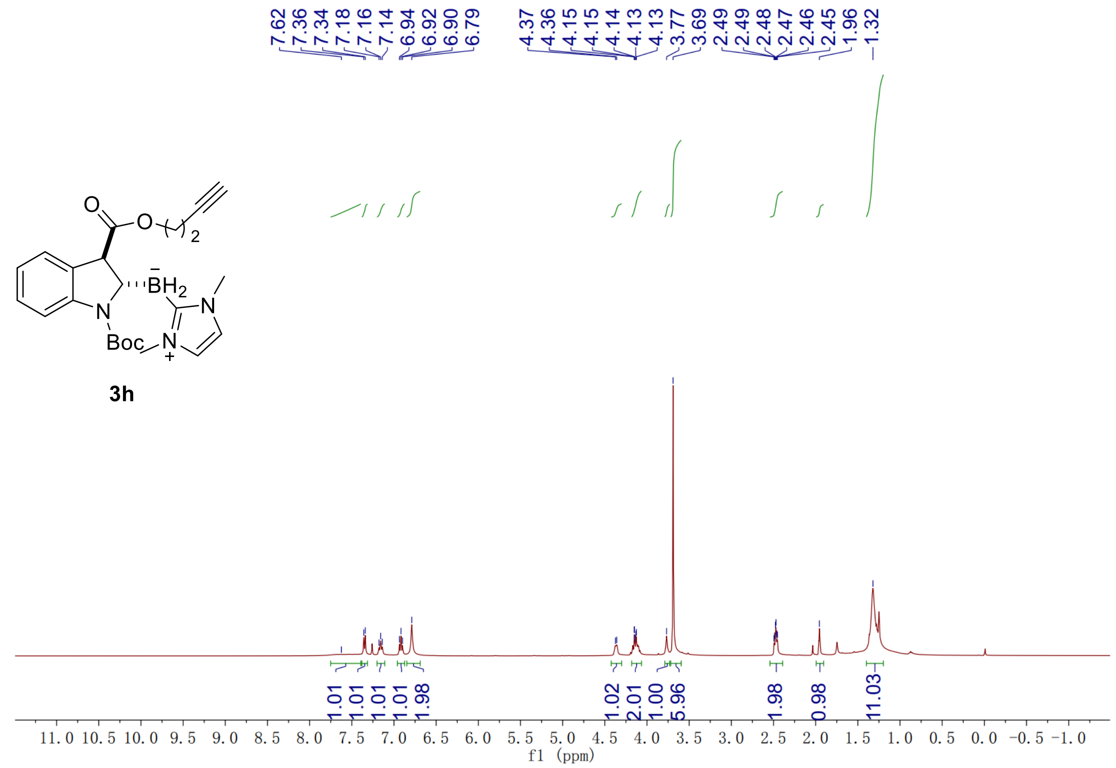
7.62
7.36
7.34
7.18
7.16
7.14
6.94
6.92
6.90
6.79
4.37
4.36
4.15
4.15
4.14
4.13
4.13
3.77
3.69
2.49
2.49
2.48
2.47
2.46
2.45
1.96
1.32
O
O
2
BH2
N
Boc
N
N
3h
1.01
1.01
1.01
1.01
1.98
1.02
2.01
1.00
5.96
1.98
0.98
11.03
11.0 10.5 10.0 9.5 9.0 8.5 8.0 7.5 7.0 6.5 6.0 5.5 5.0 4.5 4.0 3.5 3.0 2.5 2.0 1.5 1.0 0.5 0.0 -0.5 -1.0
f1 (ppm)

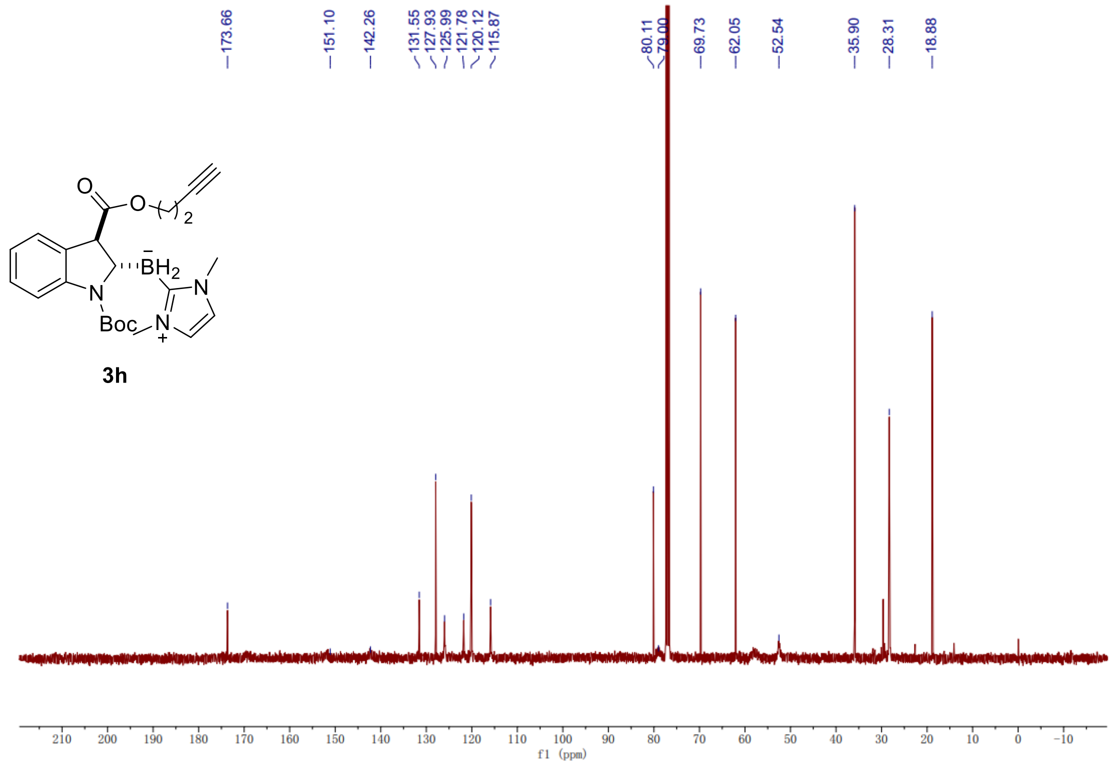
173.66
151.10
142.26
131.55
127.93
125.99
121.78
120.12
115.87
80.11
79.00
69.73
62.05
52.54
35.90
28.31
18.88
O
O
2
BH2
N
Boc
N
N
3h
210 200 190 180 170 160 150 140 130 120 110 100 90 80 70 60 50 40 30 20 10 0 -10
f1 (ppm)

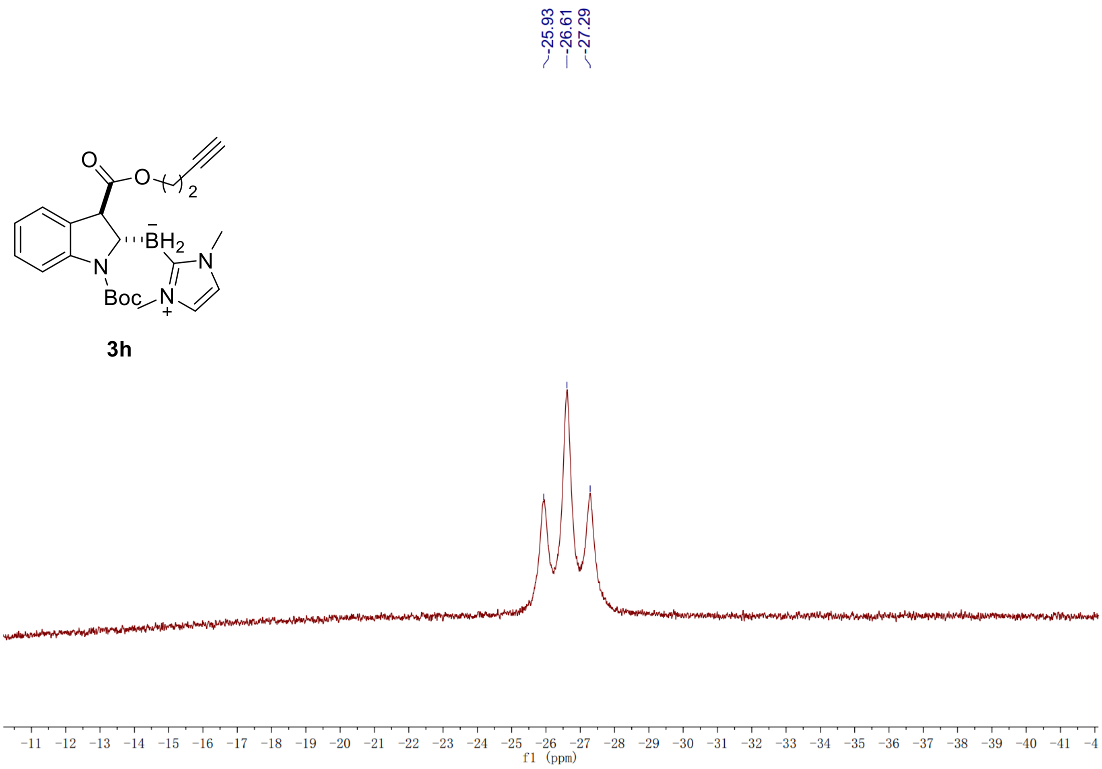
-25.93
-26.61
-27.29
f1 (ppm)

3h

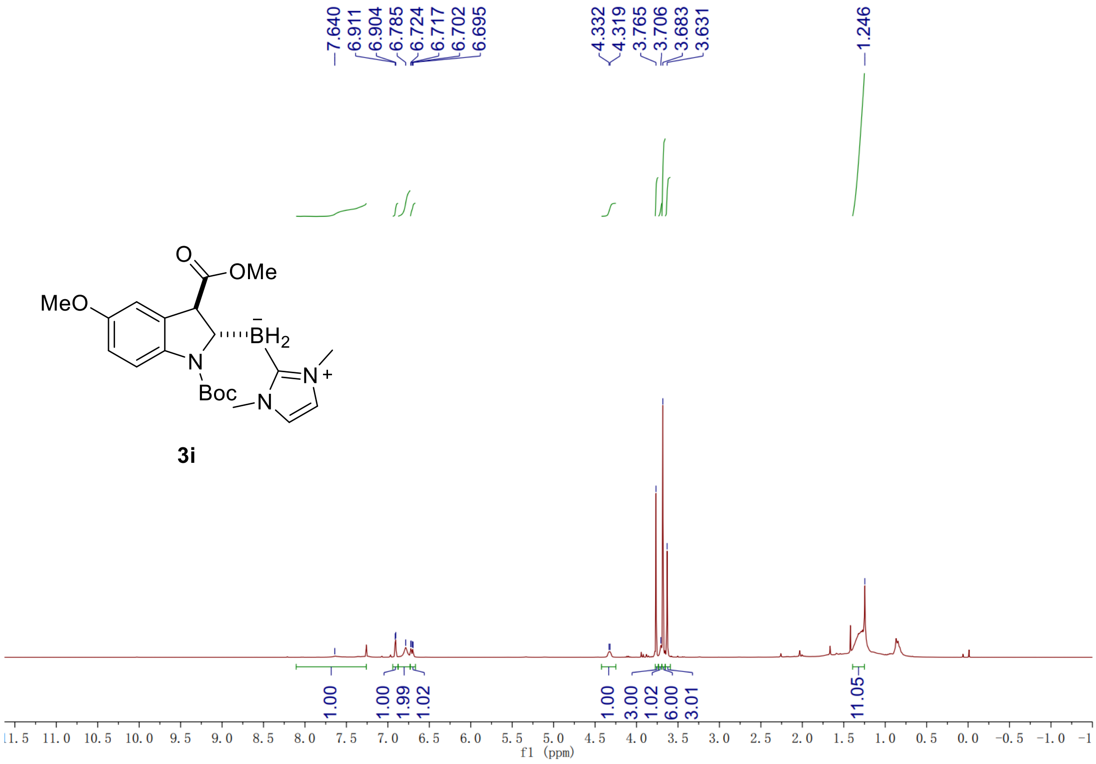
7.640
6.911
6.904
6.785
6.724
6.717
6.702
6.695
4.332
4.319
3.765
3.706
3.683
3.631
1.246
1.00
1.00
1.99
1.02
1.00
3.00
1.02
6.00
3.01
11.05
f1 (ppm)

3i

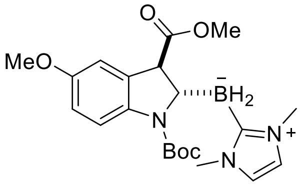

3i

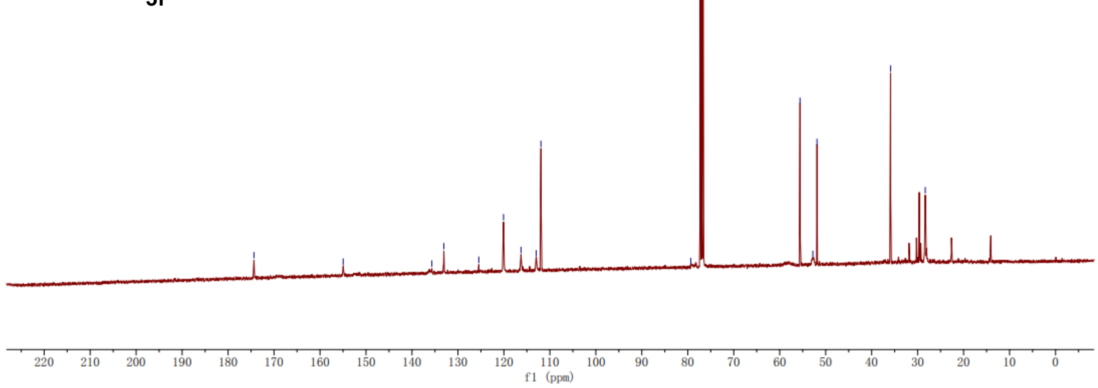

174.37
154.96
135.68
133.06
125.46
120.08
116.27
112.97
111.94
79.35
55.58
52.78
51.88
35.88
28.34
220 210 200 190 180 170 160 150 140 130 120 110 100 90 80 70 60 50 40 30 20 10 0
f1 (ppm)

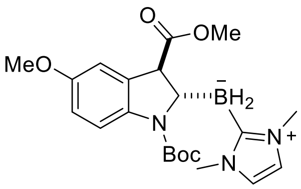

3i

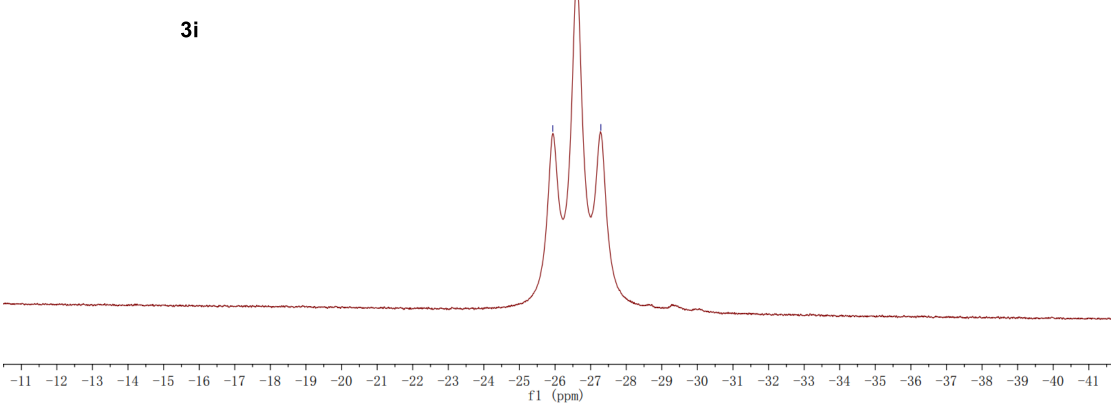

-25.93
-26.61
-27.29
−11 −12 −13 −14 −15 −16 −17 −18 −19 −20 −21 −22 −23 −24 −25 −26 −27 −28 −29 −30 −31 −32 −33 −34 −35 −36 −37 −38 −39 −40 −41
f1 (ppm)

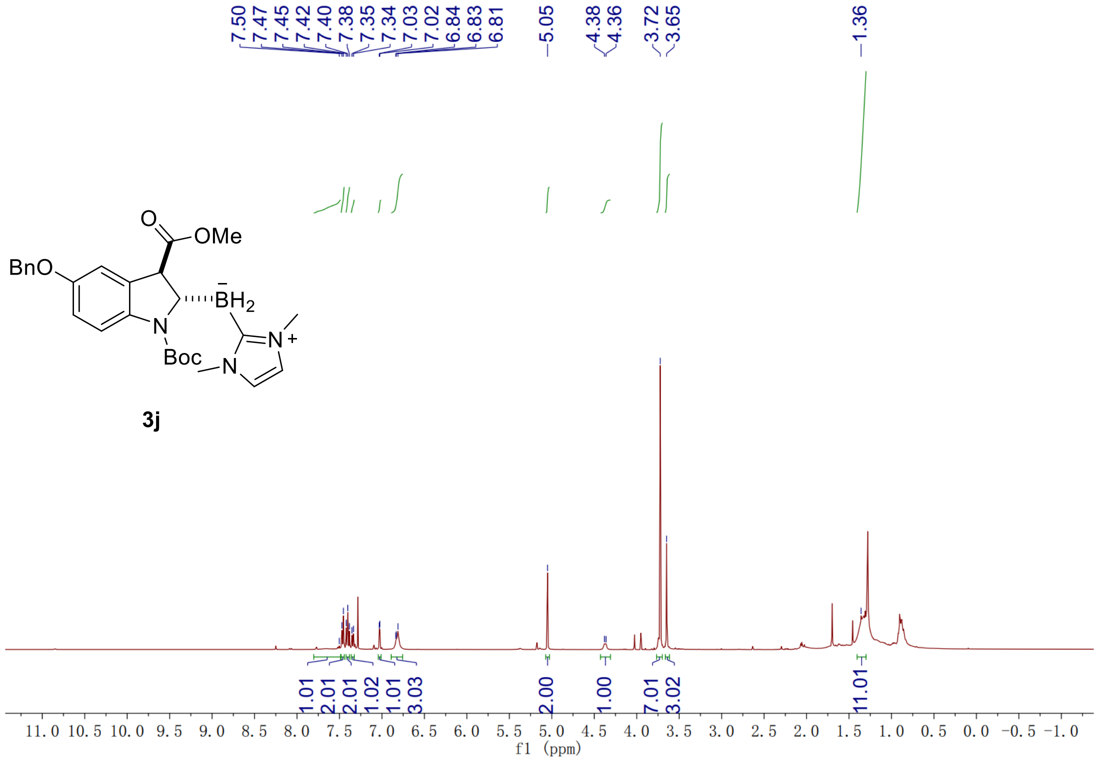
7.50 7.47 7.45 7.42 7.40 7.38 7.35 7.34 7.03 7.02 6.84 6.83 6.81
5.05
4.38 4.36
3.72 3.65
1.36
BnO
OMe
$\bar{B}H_2$
N
Boc
N+
N
3j
1.01 2.01 2.01 1.02 1.01 3.03
2.00
1.00
7.01 3.02
11.01
11.0 10.5 10.0 9.5 9.0 8.5 8.0 7.5 7.0 6.5 6.0 5.5 5.0 4.5 4.0 3.5 3.0 2.5 2.0 1.5 1.0 0.5 0.0 -0.5 -1.0
f1 (ppm)

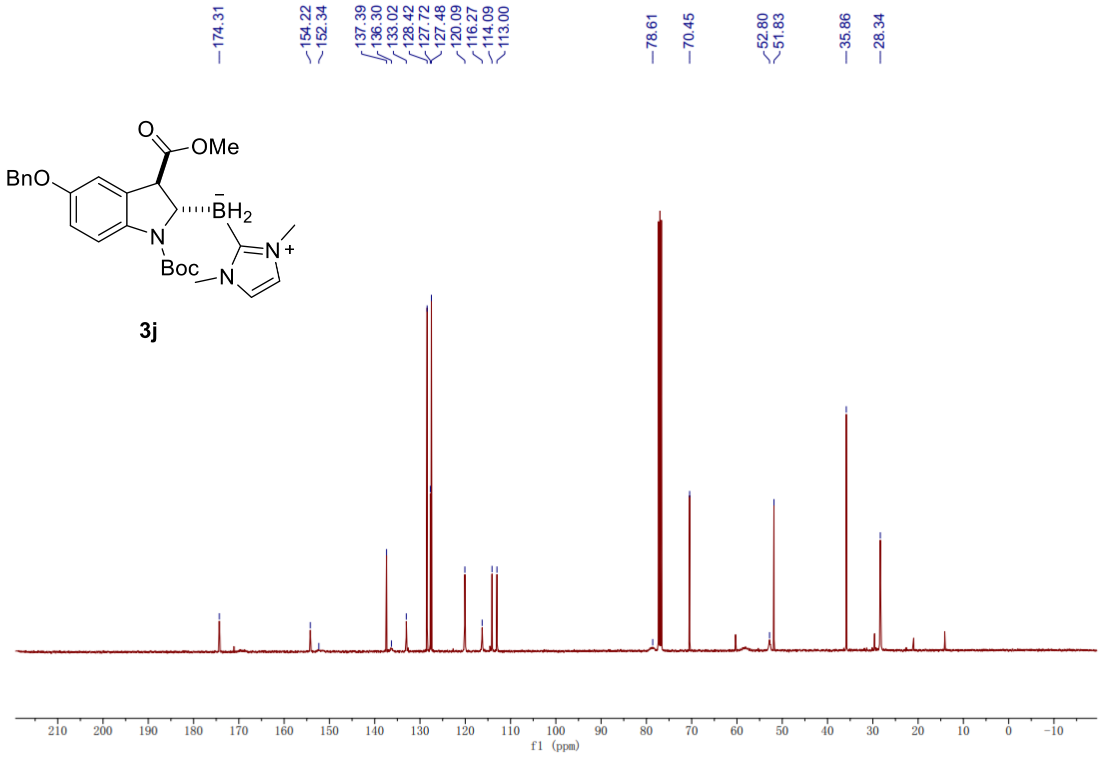
174.31
154.22 152.34
137.39 136.30 133.02 128.42 127.72 127.48 120.09 116.27 114.09 113.00
78.61
70.45
52.80 51.83
35.86
28.34
BnO
OMe
$\bar{B}H_2$
N
Boc
N+
N
3j
210 200 190 180 170 160 150 140 130 120 110 100 90 80 70 60 50 40 30 20 10 0 -10
f1 (ppm)

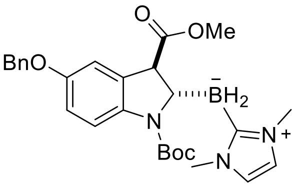

3j

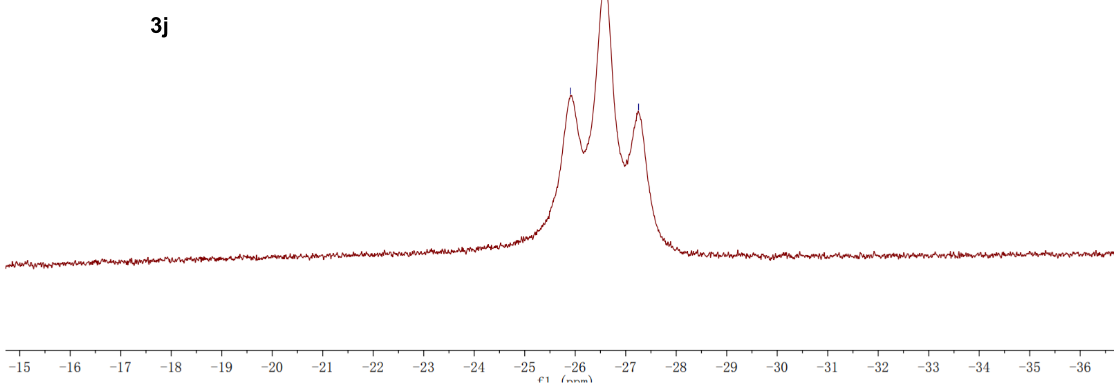

-25.91
-26.58
-27.25
f1 (ppm)

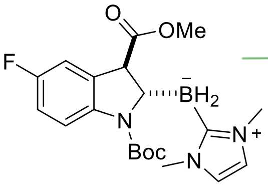

3k

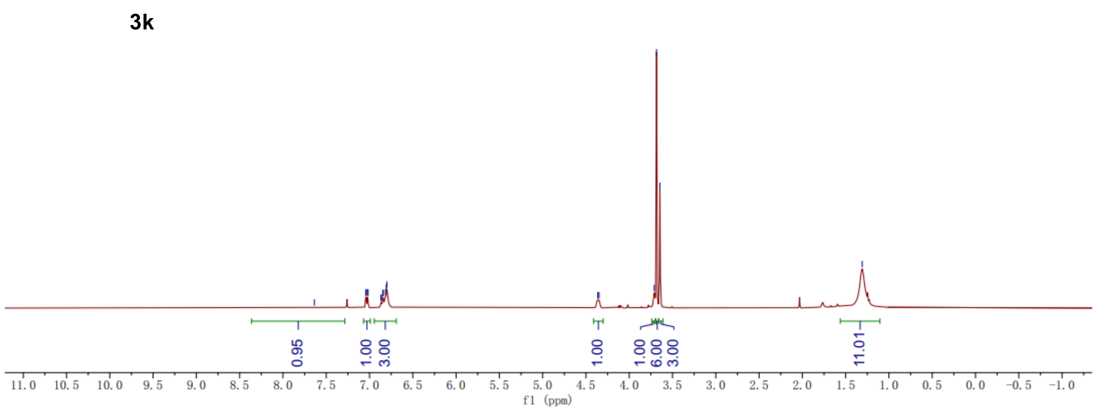

7.64
7.04
7.04
7.02
7.02
6.87
6.86
6.85
6.84
6.81
6.81
6.80
4.36
4.35
3.71
3.68
3.65
1.31
0.95
1.00
3.00
1.00
1.00
6.00
3.00
11.01
f1 (ppm)

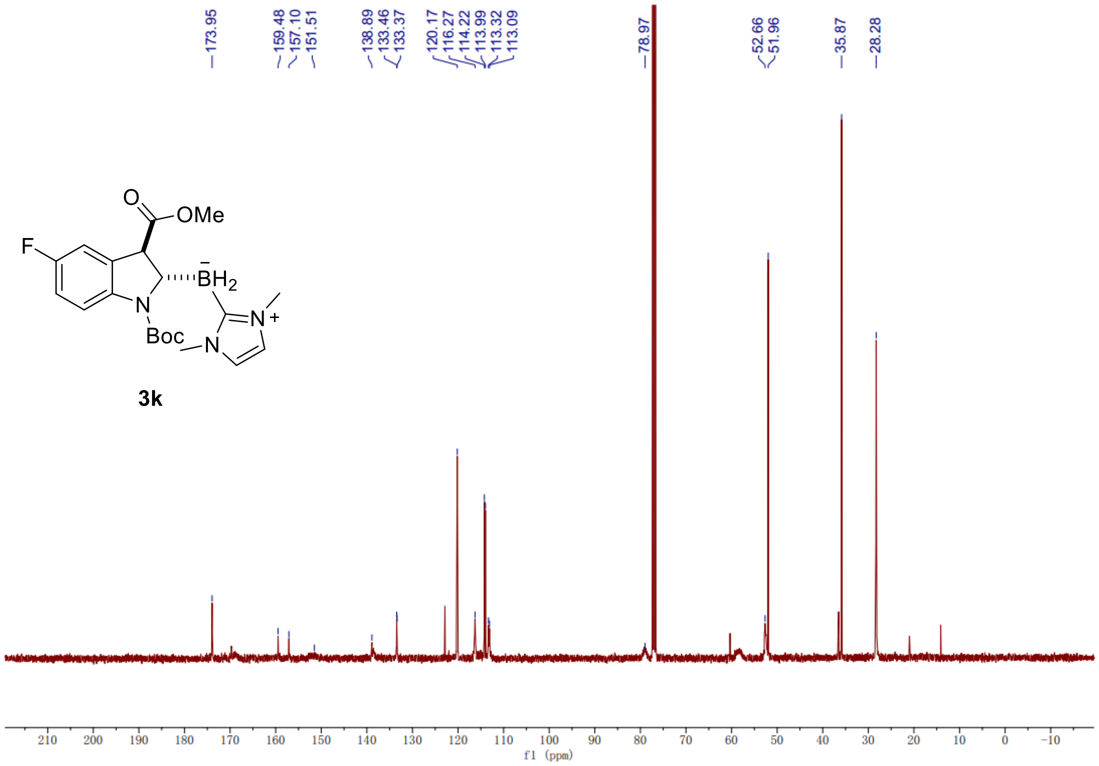
173.95
159.48
157.10
151.51
138.89
133.46
133.37
120.17
116.27
114.22
113.99
113.32
113.09
78.97
52.66
51.96
35.87
28.28
O
OMe
F
BH2
N
N+
Boc
N
3k
210 200 190 180 170 160 150 140 130 120 110 100 90 80 70 60 50 40 30 20 10 0 -10
f1 (ppm)

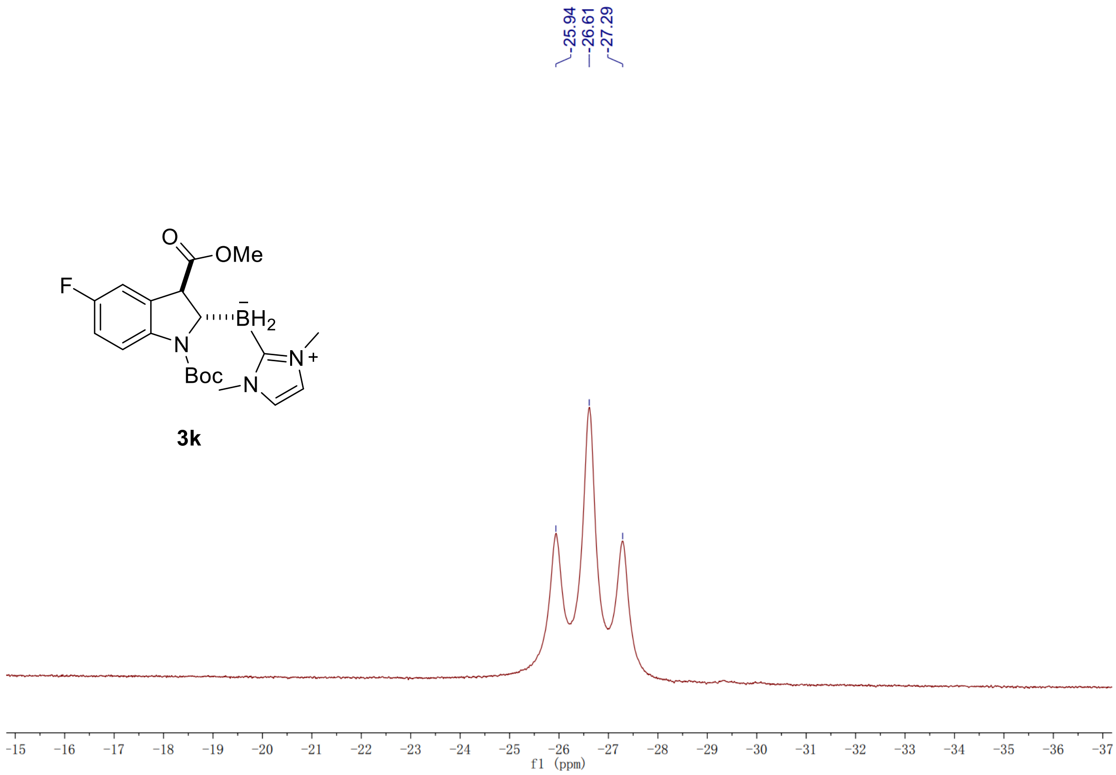
-25.94
-26.61
-27.29
O
OMe
F
BH2
N
N+
Boc
N
3k
-15 -16 -17 -18 -19 -20 -21 -22 -23 -24 -25 -26 -27 -28 -29 -30 -31 -32 -33 -34 -35 -36 -37
f1 (ppm)

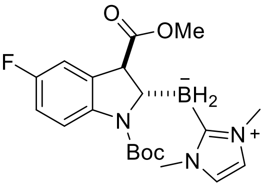

O
OMe
F
BH2
N
N+
N
Boc

3k

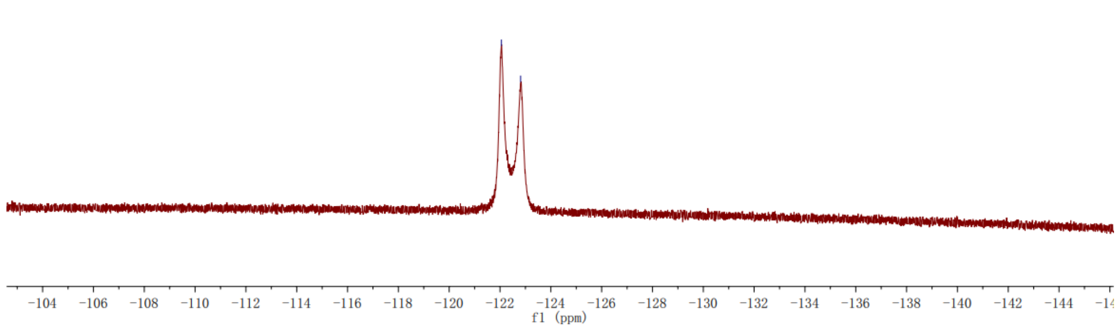

-122.05
-122.81
f1 (ppm)

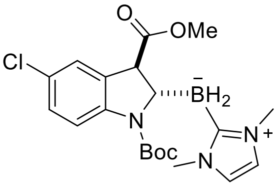

O
OMe
Cl
BH2
N
N+
N
Boc

3l

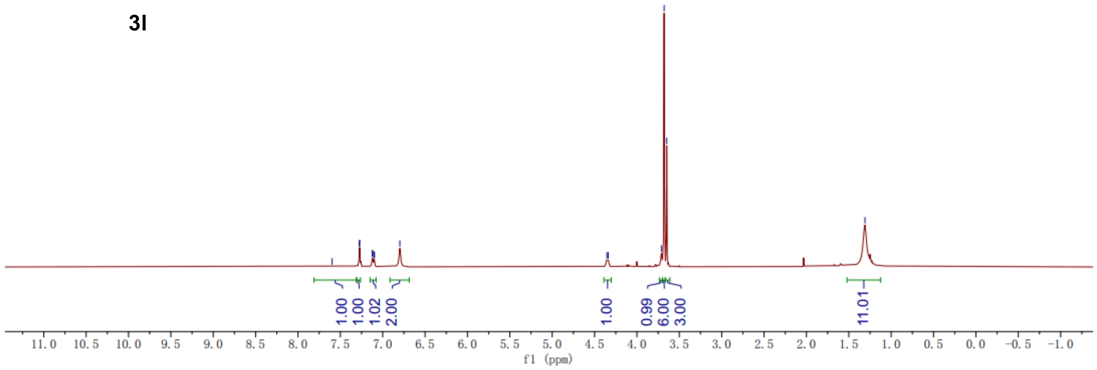

7.60
7.28
7.27
7.12
7.12
7.10
7.10
6.80
4.35
4.34
3.71
3.68
3.65
1.31
1.00
1.00
1.02
2.00
1.00
0.99
6.00
3.00
11.01
f1 (ppm)

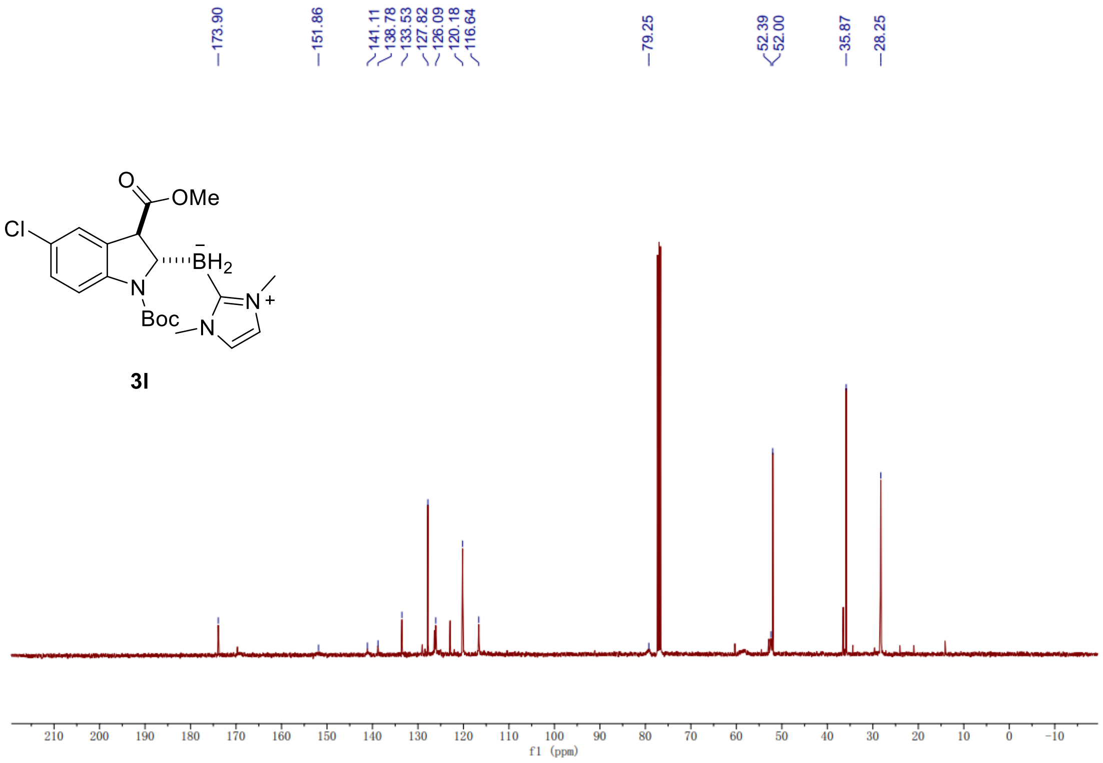
173.90
151.86
141.11
138.78
133.53
127.82
126.09
120.18
116.64
79.25
52.39
52.00
35.87
28.25
Cl
O
OMe
BH2
N
Boc
N
N+
f1 (ppm)
210 200 190 180 170 160 150 140 130 120 110 100 90 80 70 60 50 40 30 20 10 0 -10

3l

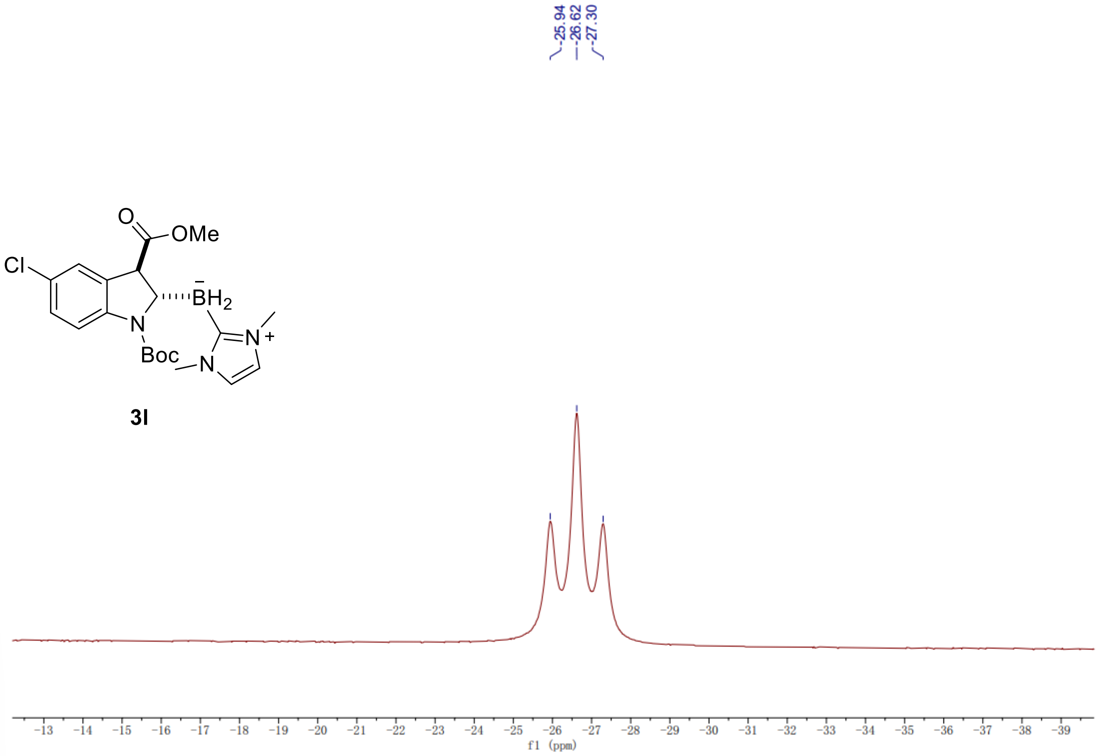
-25.94
-26.62
-27.30
Cl
O
OMe
BH2
N
Boc
N
N+
f1 (ppm)
-13 -14 -15 -16 -17 -18 -19 -20 -21 -22 -23 -24 -25 -26 -27 -28 -29 -30 -31 -32 -33 -34 -35 -36 -37 -38 -39

3l

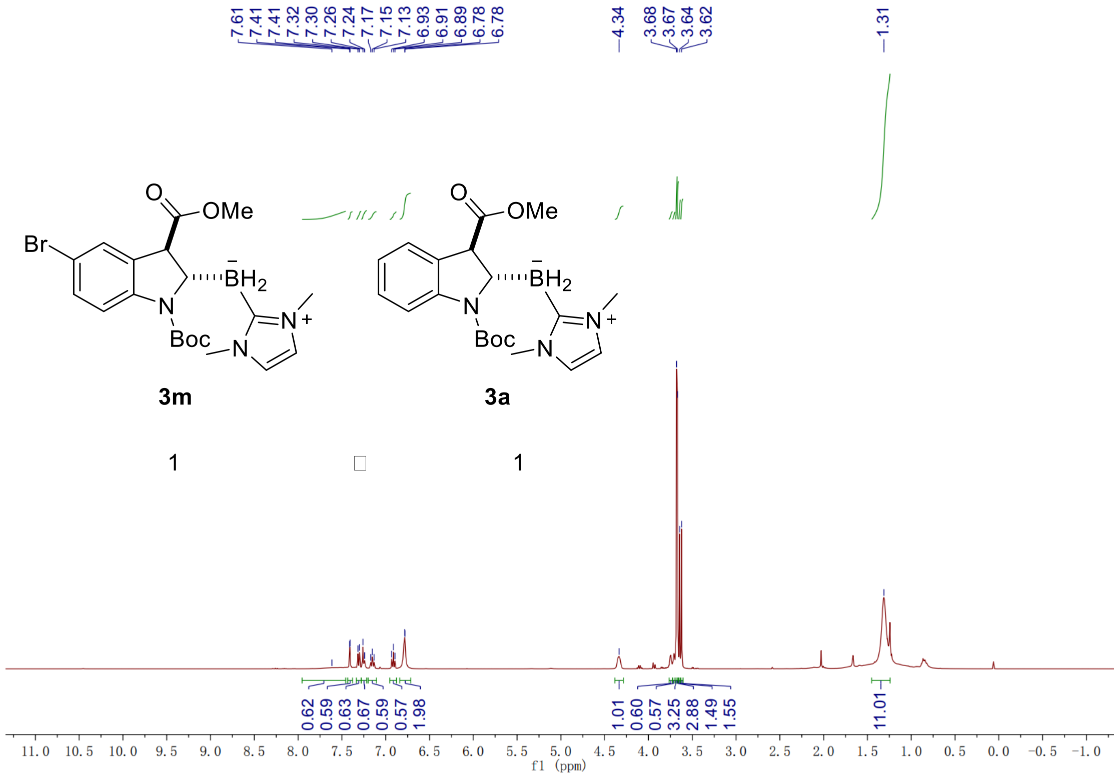
7.61 7.41 7.41 7.32 7.30 7.26 7.24 7.17 7.15 7.13 6.93 6.91 6.89 6.78 6.78
4.34 3.68 3.67 3.64 3.62
1.31
3m
3a
1 □ 1
0.62 0.59 0.63 0.67 0.59 0.57 1.98
1.01 0.60 0.57 3.25 2.88 1.49 1.55
11.01
f1 (ppm)

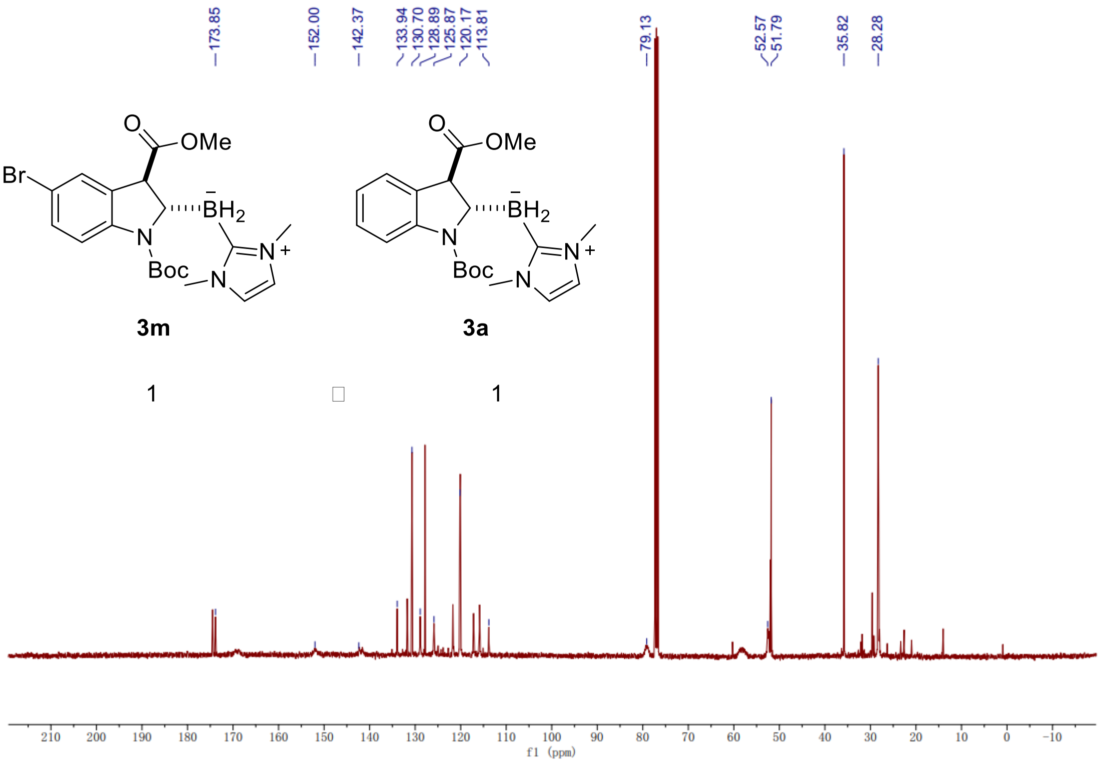
173.85
152.00
142.37
133.94 130.70 128.89 125.87 120.17 113.81
79.13
52.57 51.79
35.82
28.28
3m
3a
1 □ 1
f1 (ppm)

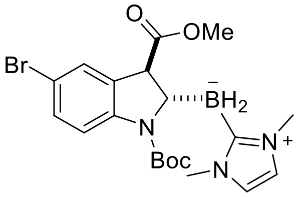

3m

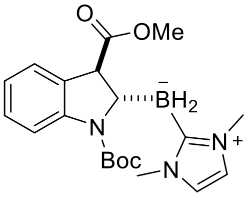

3a

1 □ 1

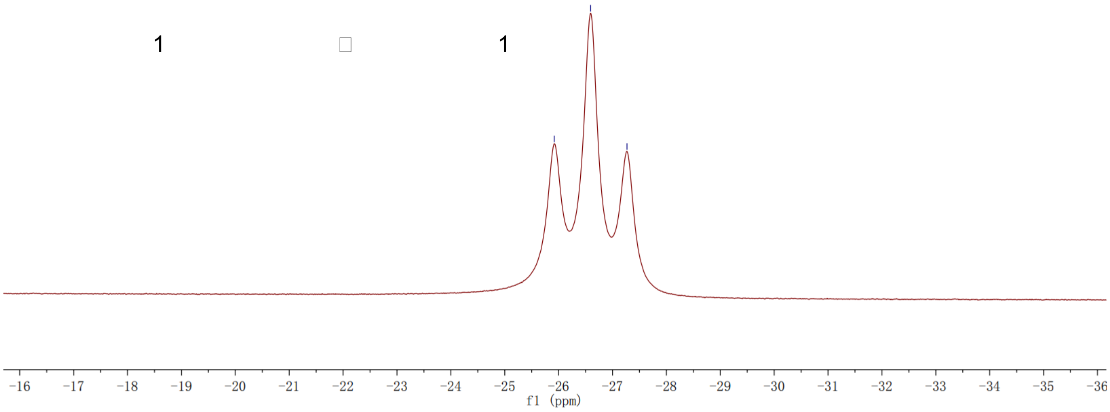

-25.92
-26.59
-27.27
f1 (ppm)

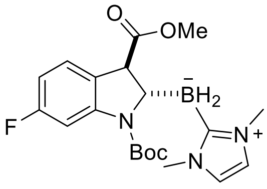

3n

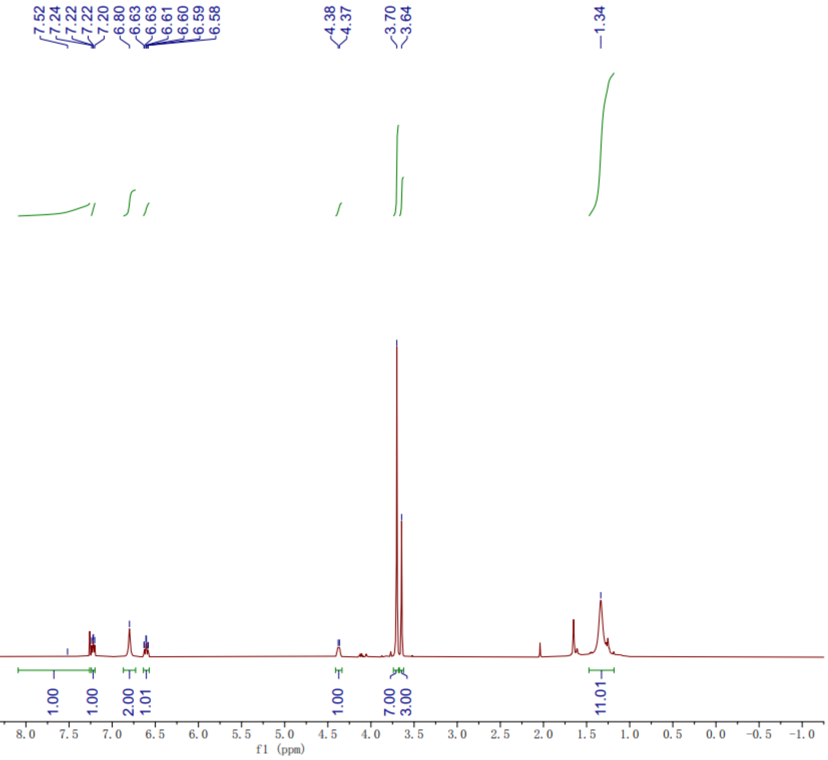

7.52
7.24
7.22
7.22
7.20
6.80
6.63
6.63
6.61
6.60
6.59
6.58
4.38
4.37
3.70
3.64
1.34
1.00
1.00
2.00
1.01
1.00
7.00
3.00
11.01
f1 (ppm)

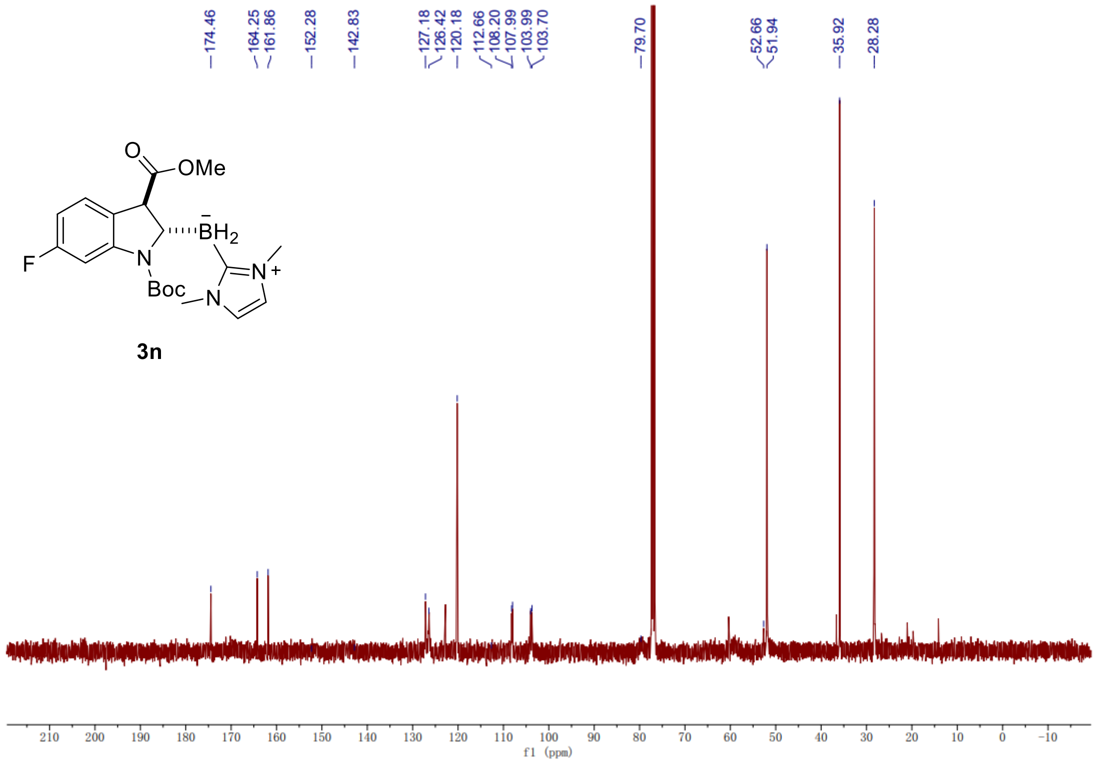
174.46
164.25
161.86
152.28
142.83
127.18
126.42
120.18
112.66
108.20
107.99
103.99
103.70
79.70
52.66
51.94
35.92
28.28
O
OMe
BH2
F
N
Boc
N
N+
3n
210 200 190 180 170 160 150 140 130 120 110 100 90 80 70 60 50 40 30 20 10 0 -10
f1 (ppm)

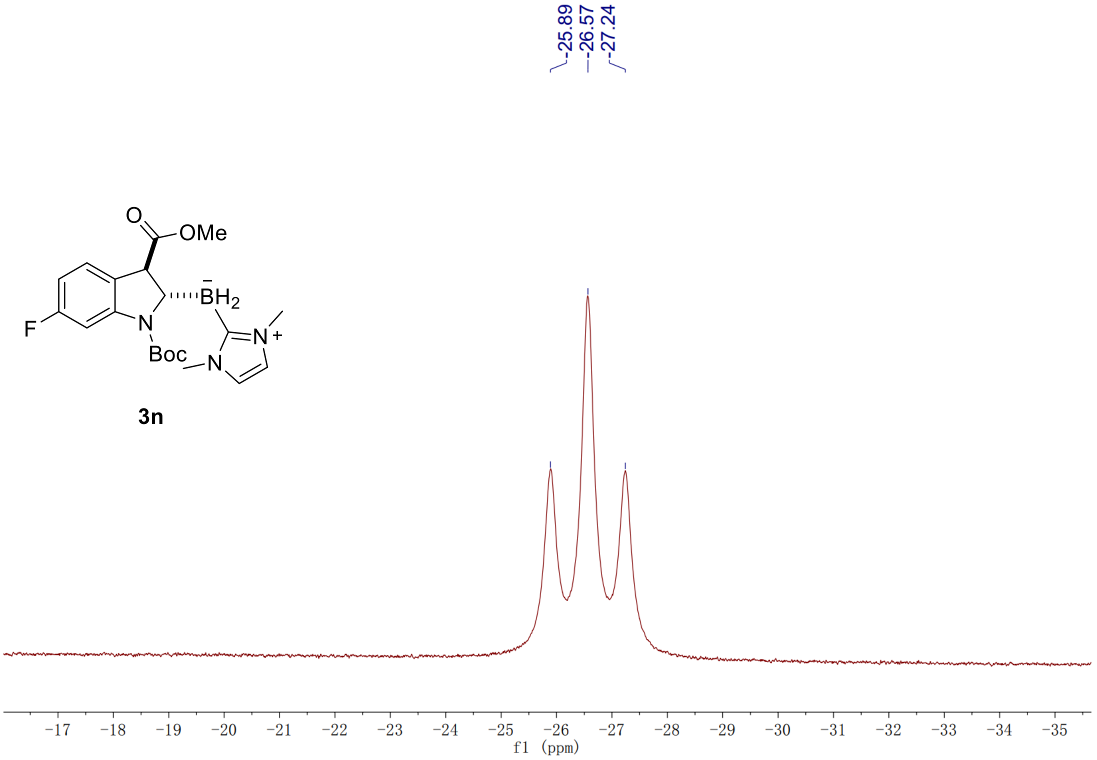
-25.89
-26.57
-27.24
O
OMe
BH2
F
N
Boc
N
N+
3n
-17 -18 -19 -20 -21 -22 -23 -24 -25 -26 -27 -28 -29 -30 -31 -32 -33 -34 -35
f1 (ppm)

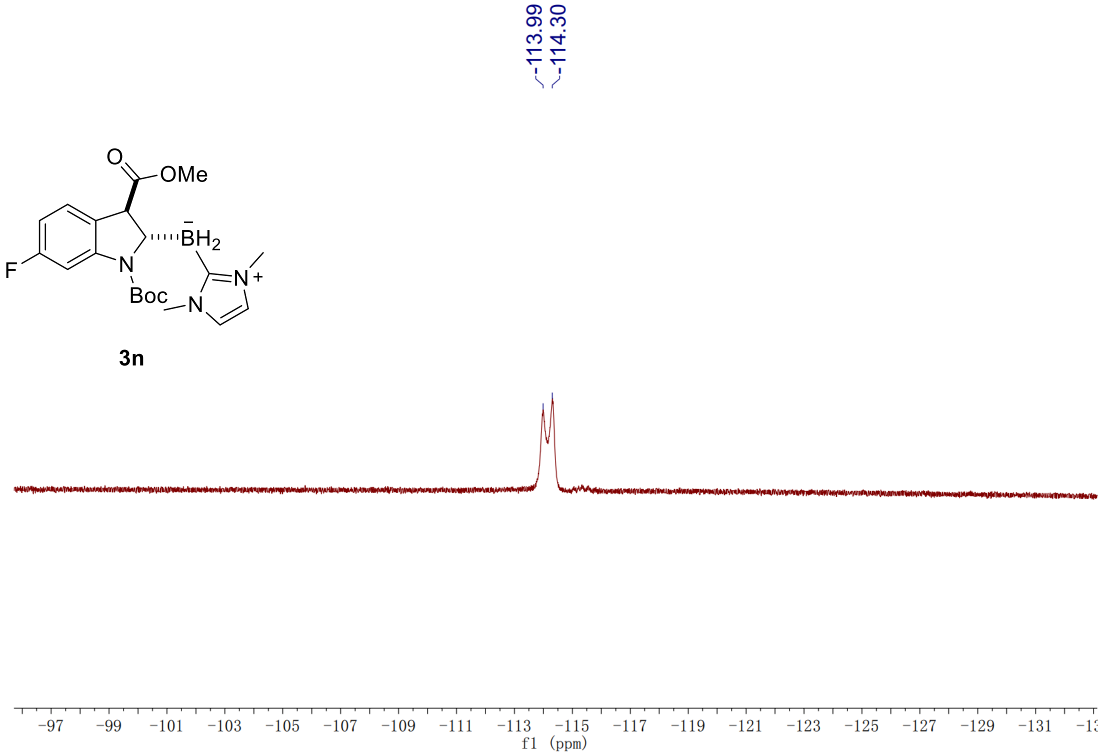
-113.99
-114.30
O
OMe
BH2
F
N
Boc
N
N+
3n
-97 -99 -101 -103 -105 -107 -109 -111 -113 -115 -117 -119 -121 -123 -125 -127 -129 -131
f1 (ppm)

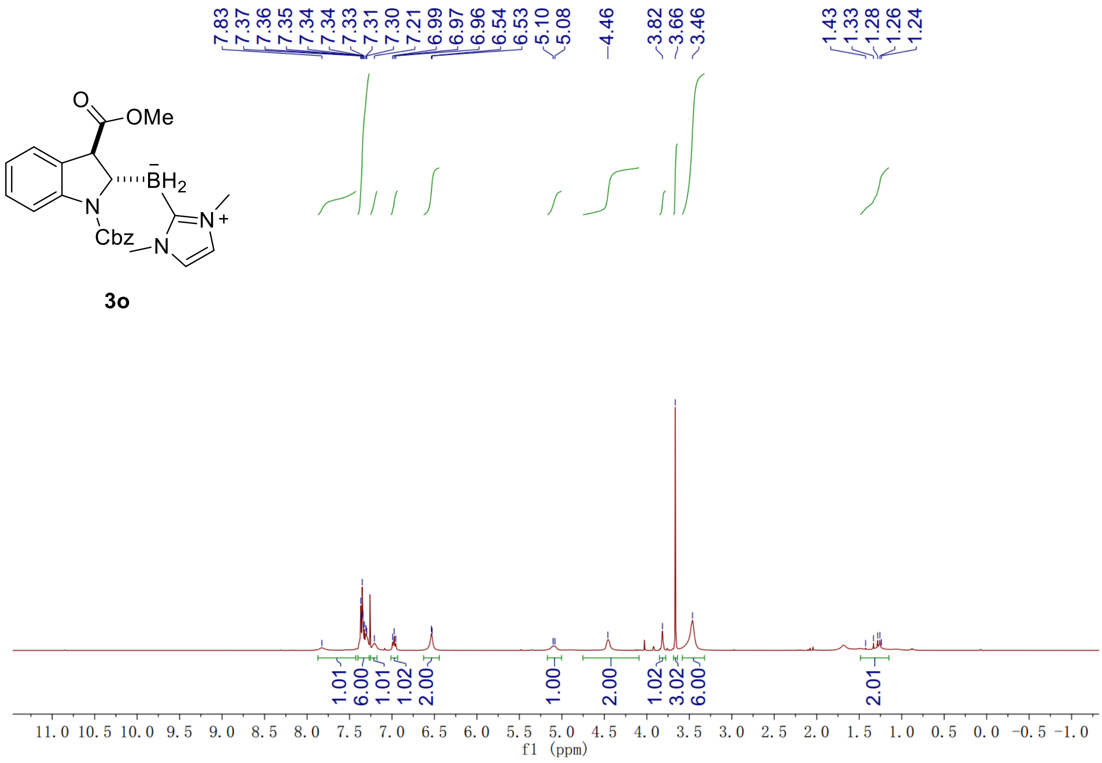
7.83 7.37 7.36 7.35 7.34 7.34 7.33 7.31 7.30 7.21 6.99 6.97 6.96 6.54 6.53 5.10 5.08 4.46 3.82 3.66 3.46 1.43 1.33 1.28 1.26 1.24
O
OMe
BH2
N
Cbz
N
N+
3o
1.01 6.00 1.01 1.02 2.00 1.00 2.00 1.02 3.02 6.00 2.01
11.0 10.5 10.0 9.5 9.0 8.5 8.0 7.5 7.0 6.5 6.0 5.5 5.0 4.5 4.0 3.5 3.0 2.5 2.0 1.5 1.0 0.5 0.0 -0.5 -1.0
f1 (ppm)

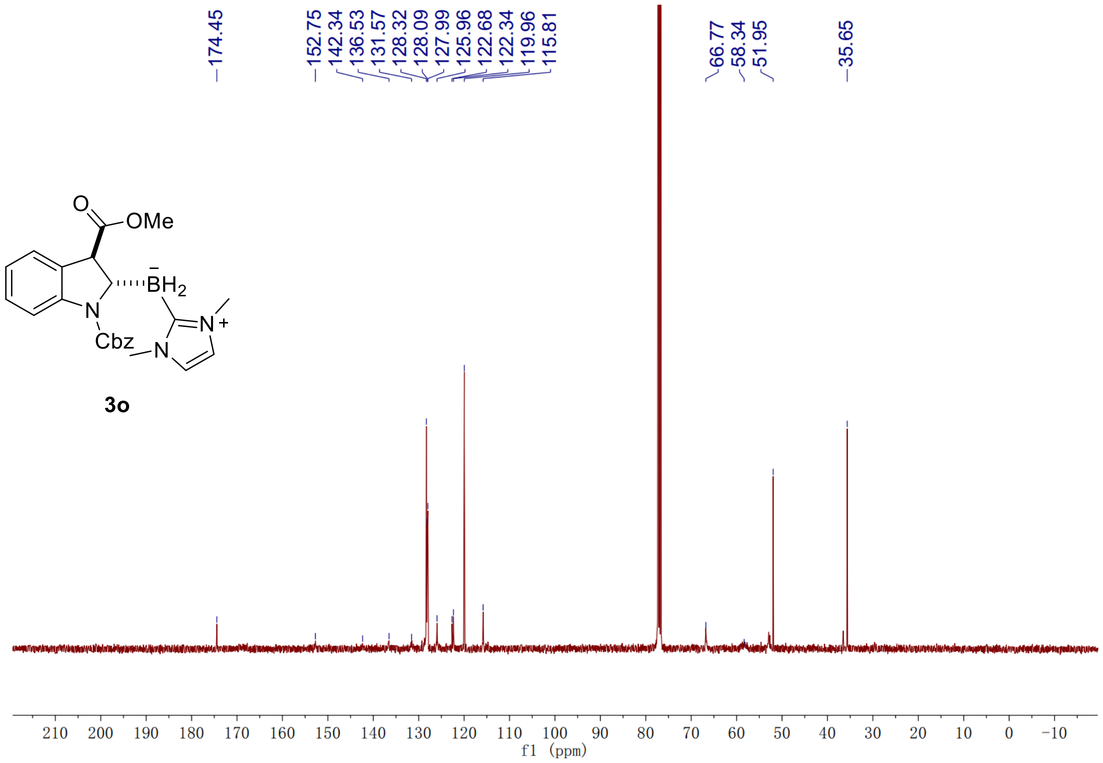
174.45
152.75
142.34
136.53
131.57
128.32
128.09
127.99
125.96
122.68
122.34
119.96
115.81
66.77
58.34
51.95
35.65
OMe
O
BH2
N
Cbz
N+
N
210 200 190 180 170 160 150 140 130 120 110 100 90 80 70 60 50 40 30 20 10 0 −10
f1 (ppm)

3o

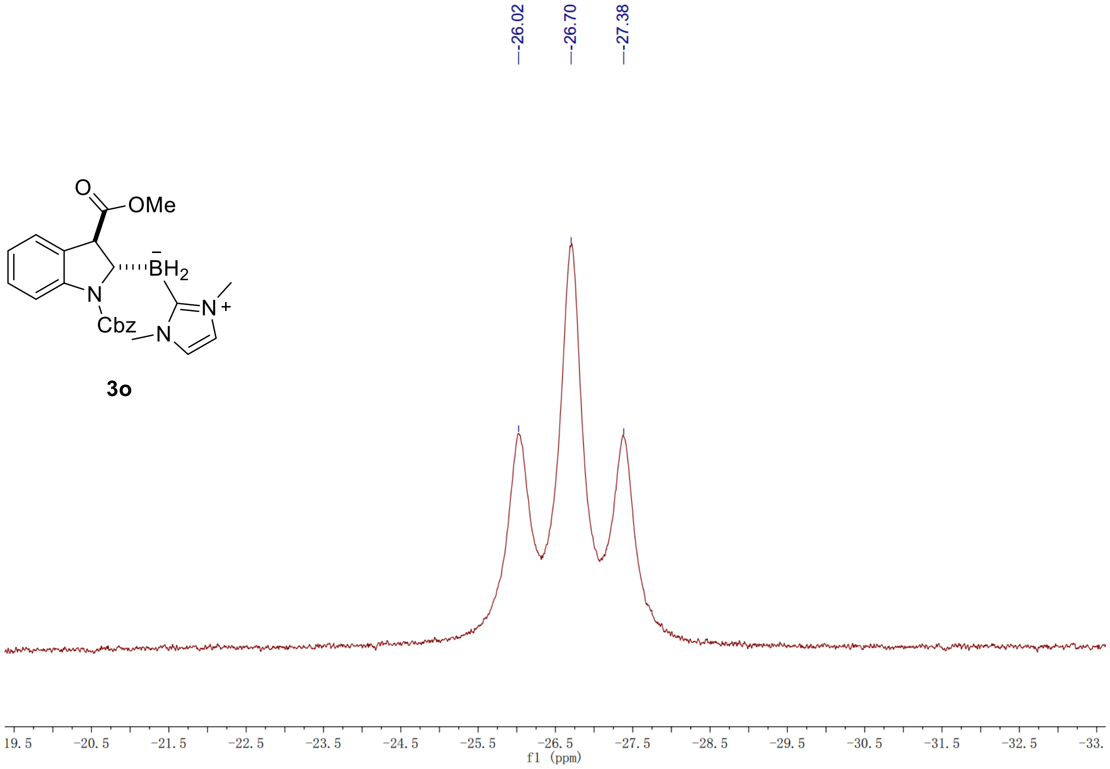
-26.02
-26.70
-27.38
OMe
O
BH2
N
Cbz
N+
N
19.5 −20.5 −21.5 −22.5 −23.5 −24.5 −25.5 −26.5 −27.5 −28.5 −29.5 −30.5 −31.5 −32.5 −33.
f1 (ppm)

3o

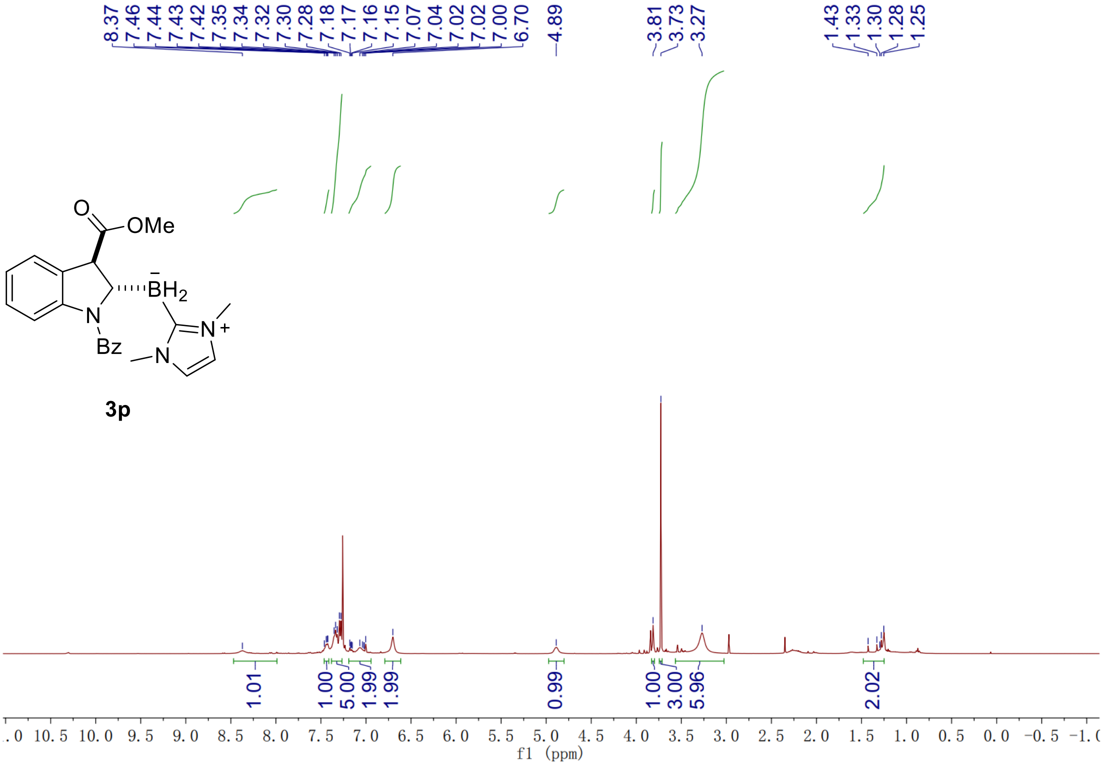
8.37
7.46
7.44
7.43
7.42
7.35
7.34
7.32
7.30
7.28
7.18
7.17
7.16
7.15
7.07
7.04
7.02
7.02
7.00
6.70
4.89
3.81
3.73
3.27
1.43
1.33
1.30
1.28
1.25
OMe
BH2
N
Bz
N+
N
1.01
1.00
5.00
1.99
1.99
0.99
1.00
3.00
5.96
2.02
f1 (ppm)

3p

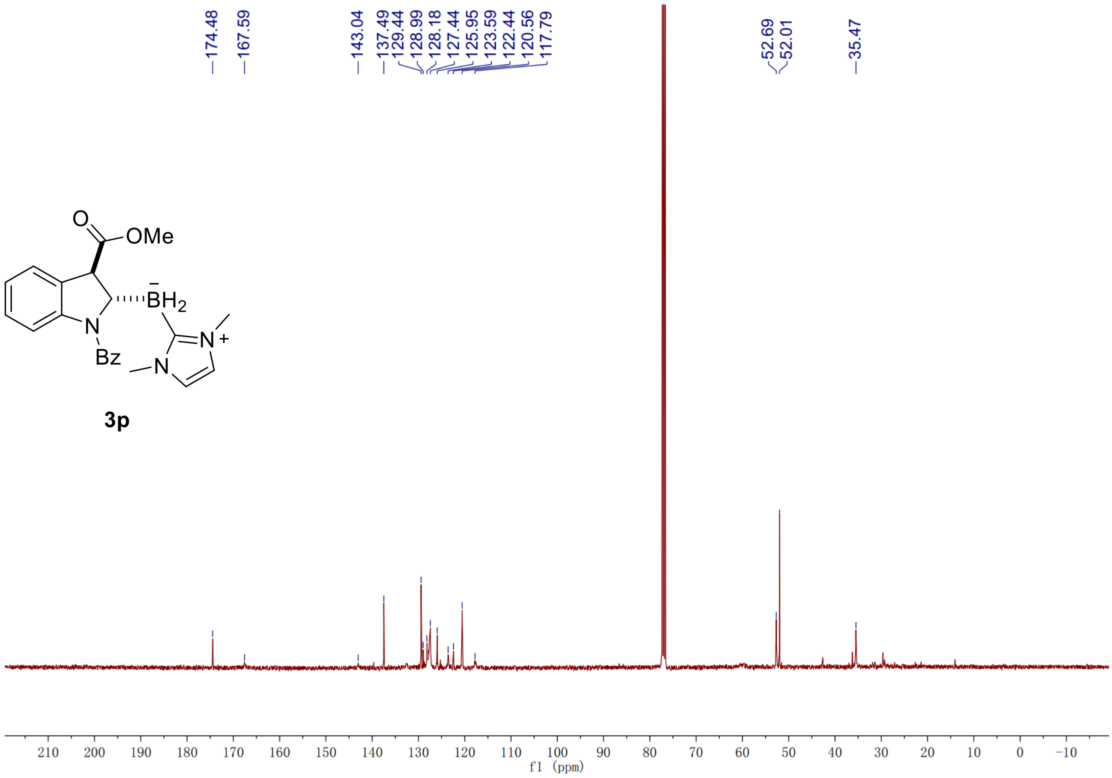
174.48
167.59
143.04
137.49
129.44
128.99
128.18
127.44
125.95
123.59
122.44
120.56
117.79
52.69
52.01
35.47
OMe
BH2
N
Bz
N+
N
f1 (ppm)

3p

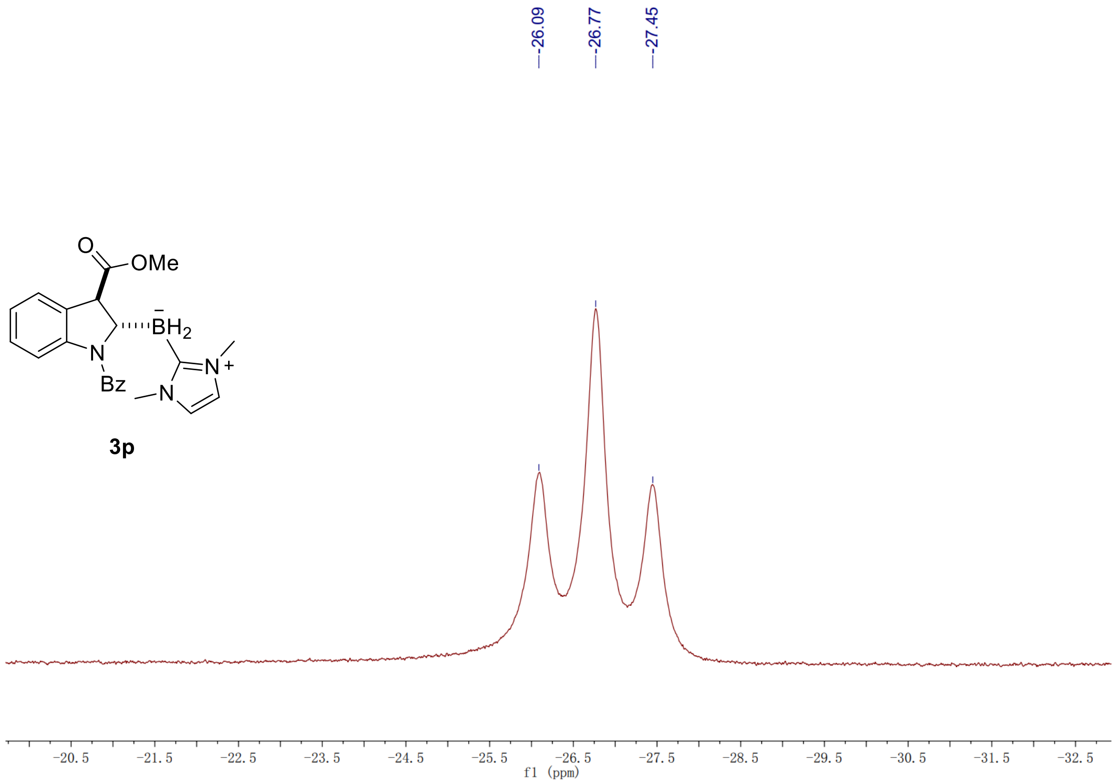

OMe
O
BH2
N
Bz
N+
N
-26.09
-26.77
-27.45
-20.5
-21.5
-22.5
-23.5
-24.5
-25.5
-26.5
-27.5
-28.5
-29.5
-30.5
-31.5
-32.5
f1 (ppm)

3p

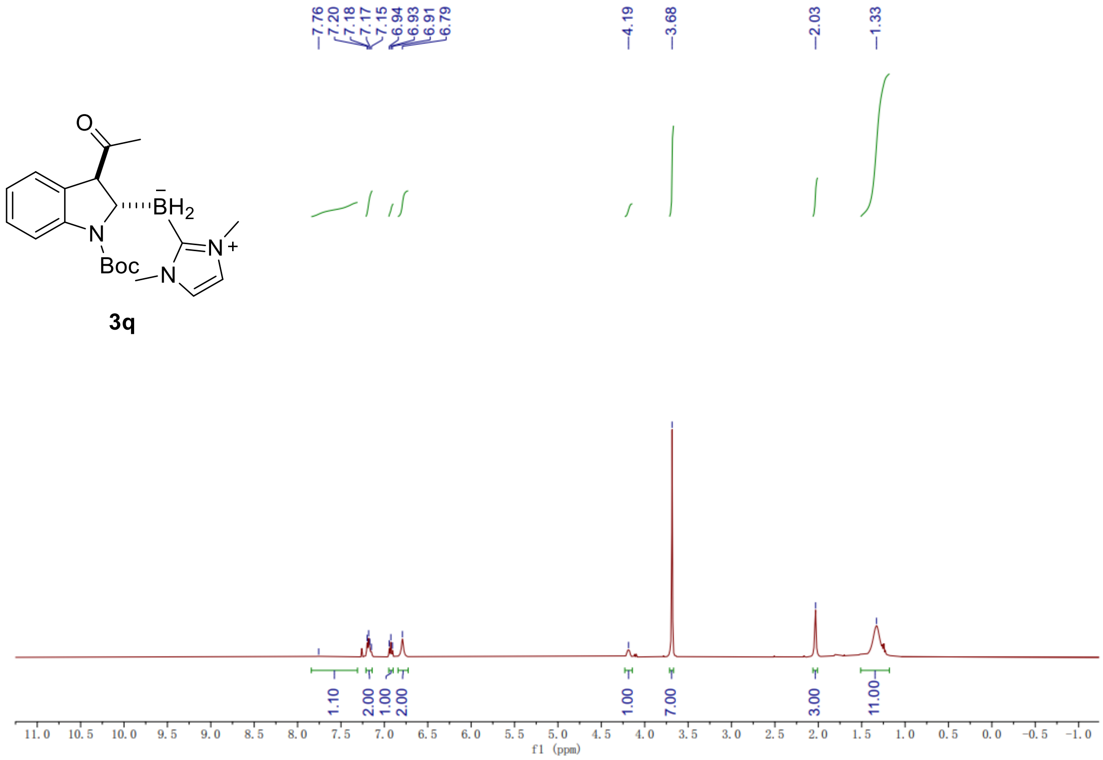

O
BH2
N
Boc
N+
N
7.76
7.20
7.18
7.17
7.15
6.94
6.93
6.91
6.79
4.19
3.68
2.03
1.33
1.10
2.00
1.00
2.00
1.00
7.00
3.00
11.00
11.0
10.5
10.0
9.5
9.0
8.5
8.0
7.5
7.0
6.5
6.0
5.5
5.0
4.5
4.0
3.5
3.0
2.5
2.0
1.5
1.0
0.5
0.0
-0.5
-1.0
f1 (ppm)

3q

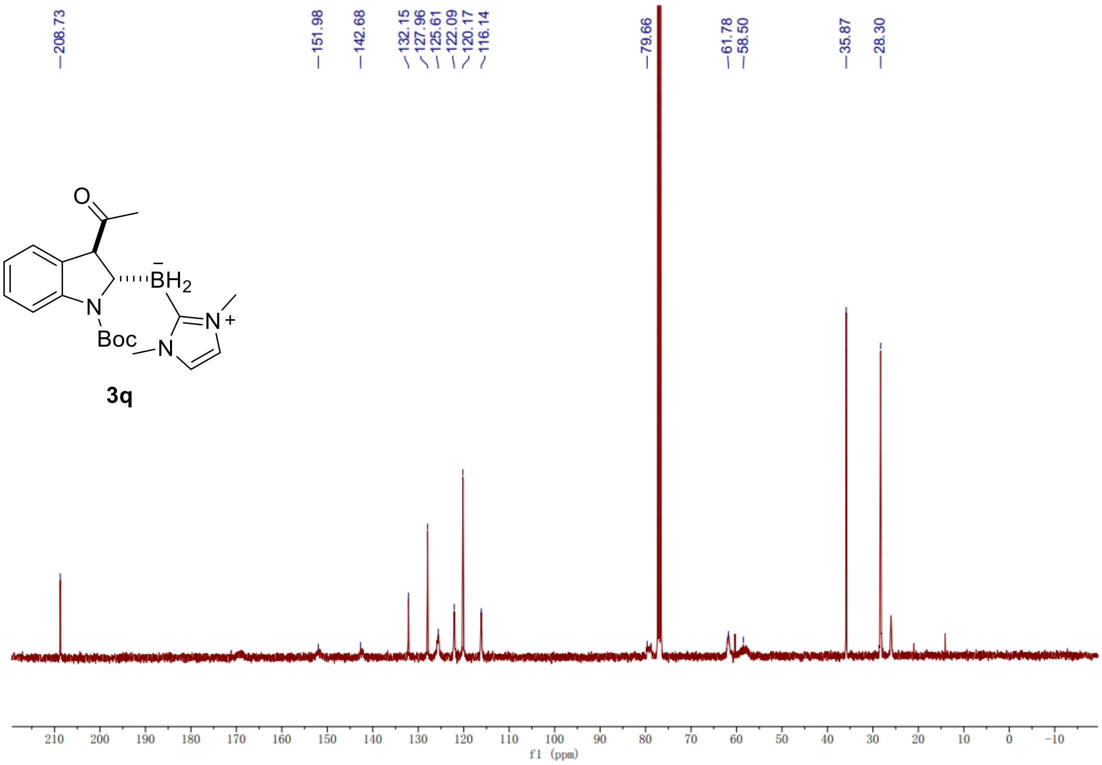
208.73
151.98
142.68
132.15
127.96
125.61
122.09
120.17
116.14
79.66
61.78
58.50
35.87
28.30
O
BH2
N
Boc
N
N+
3q
210 200 190 180 170 160 150 140 130 120 110 100 90 80 70 60 50 40 30 20 10 0 -10
f1 (ppm)

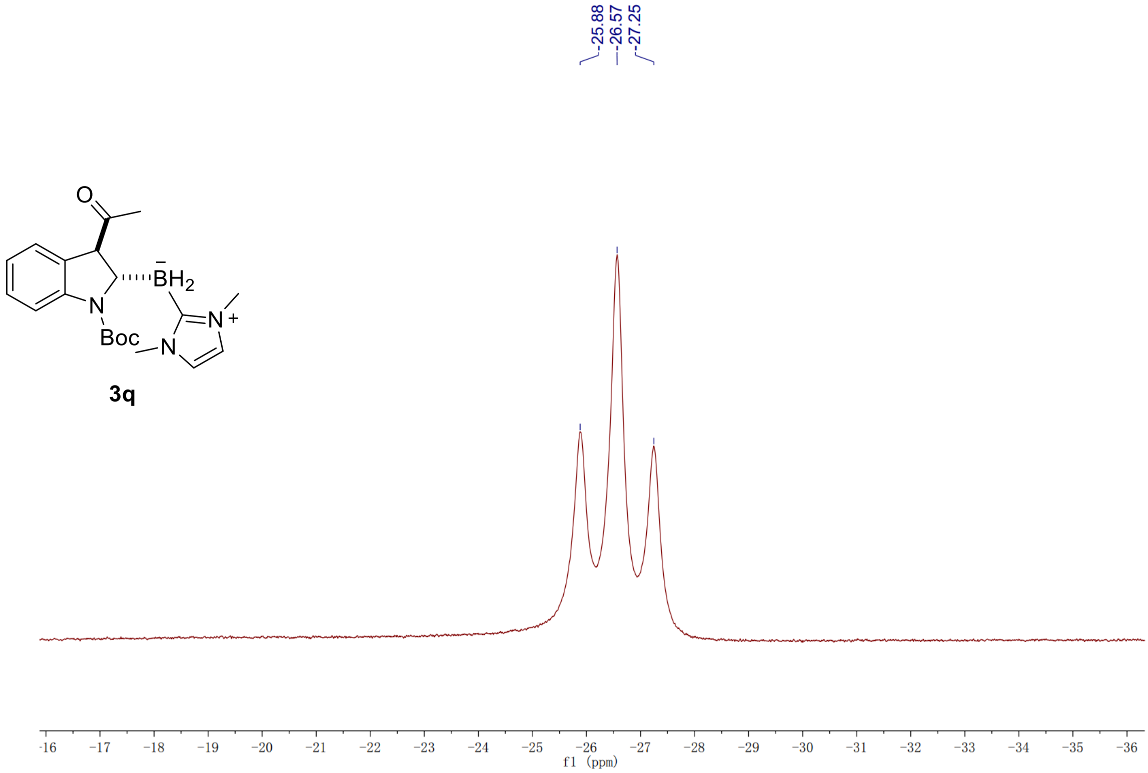
-25.88
-26.57
-27.25
O
BH2
N
Boc
N
N+
3q
-16 -17 -18 -19 -20 -21 -22 -23 -24 -25 -26 -27 -28 -29 -30 -31 -32 -33 -34 -35 -36
f1 (ppm)

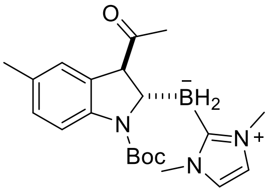
O
BH2
N
Boc
N+
N

3r

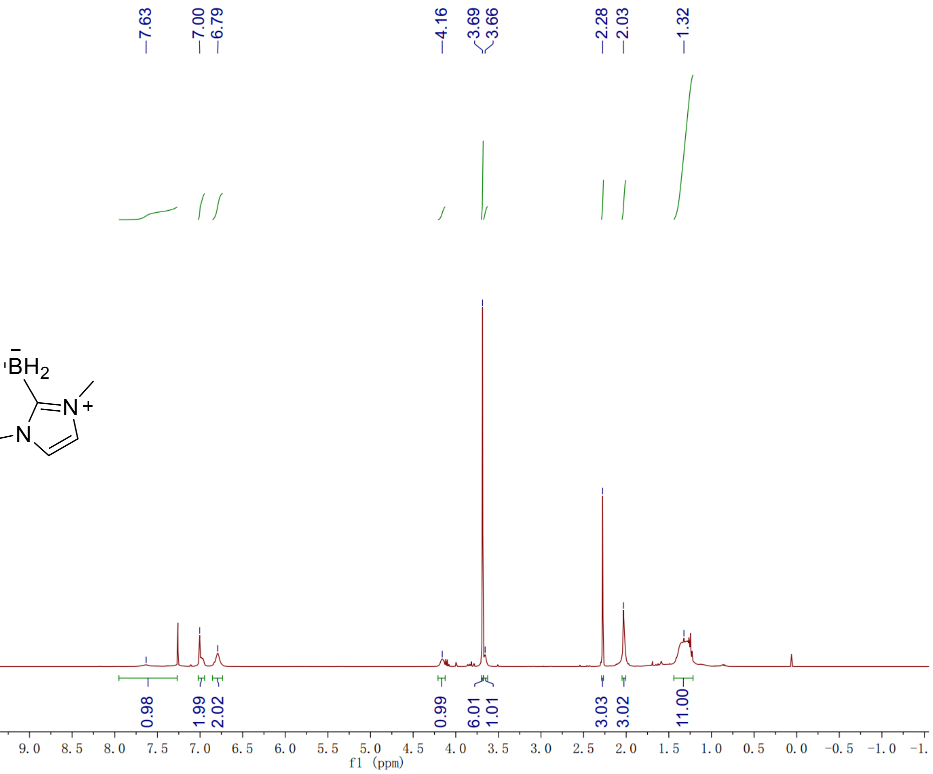
7.63
7.00
6.79
4.16
3.69
3.66
2.28
2.03
1.32
0.98
1.99
2.02
0.99
6.01
1.01
3.03
3.02
11.00
f1 (ppm)

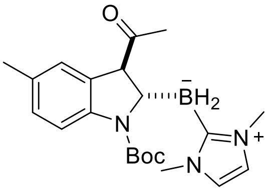
O
BH2
N
Boc
N+
N

3r

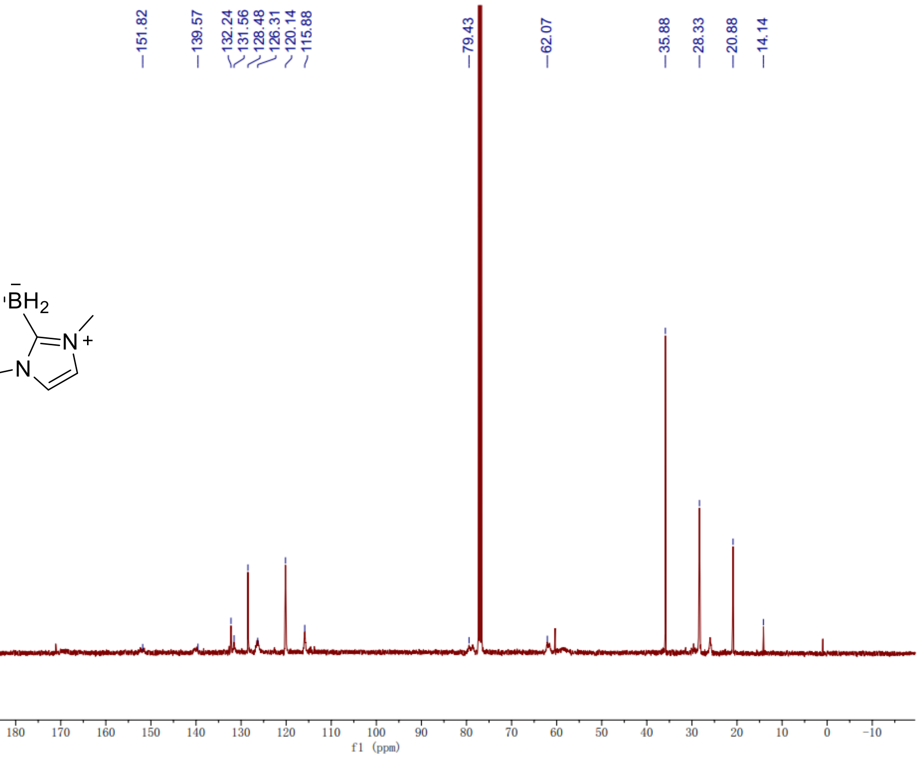
208.97
151.82
139.57
132.24
131.56
128.48
126.31
120.14
115.88
79.43
62.07
35.88
28.33
20.88
14.14
f1 (ppm)

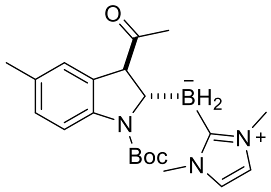
O
BH2
N
Boc
N
N+

3r

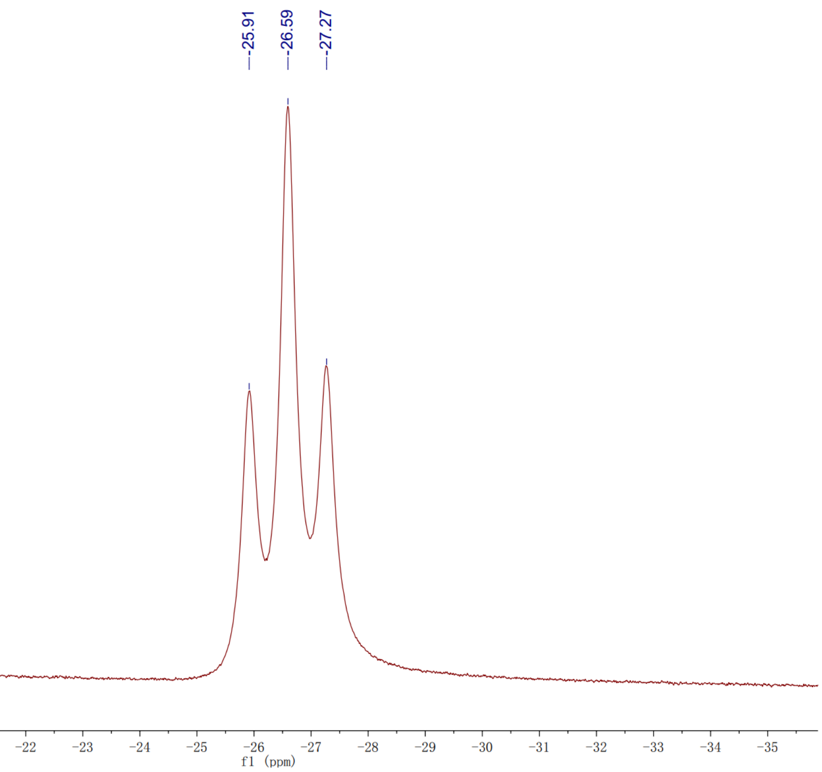
-25.91
-26.59
-27.27
f1 (ppm)

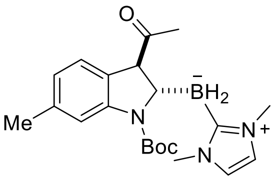
O
BH2
Me
N
Boc
N
N+

3s

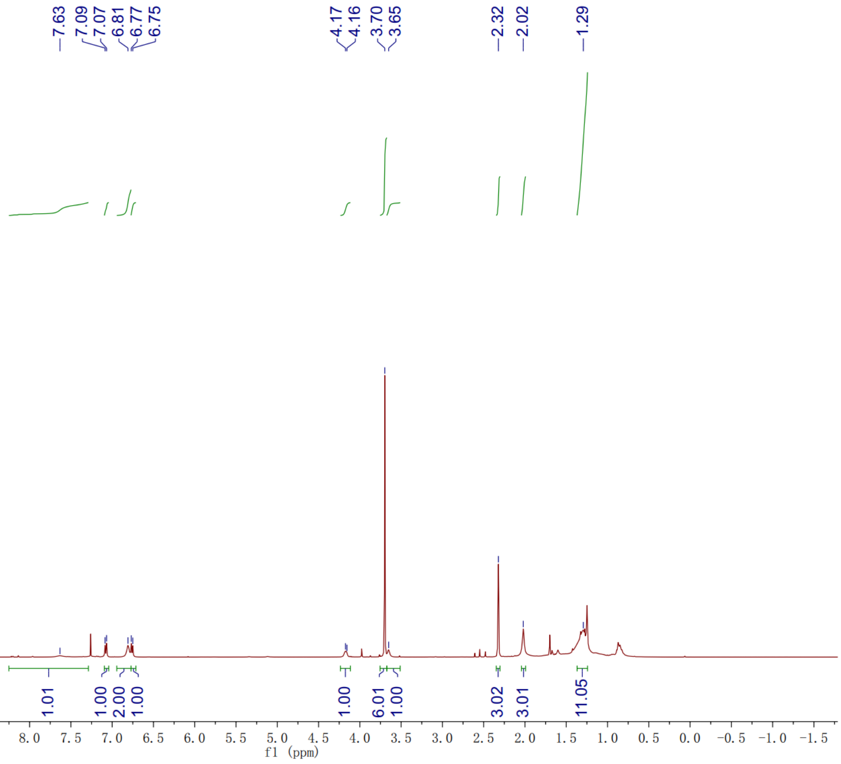
7.63
7.09
7.07
6.81
6.77
6.75
4.17
4.16
3.70
3.65
2.32
2.02
1.29
1.01
1.00
2.00
1.00
1.00
6.01
1.00
3.02
3.01
11.05
f1 (ppm)

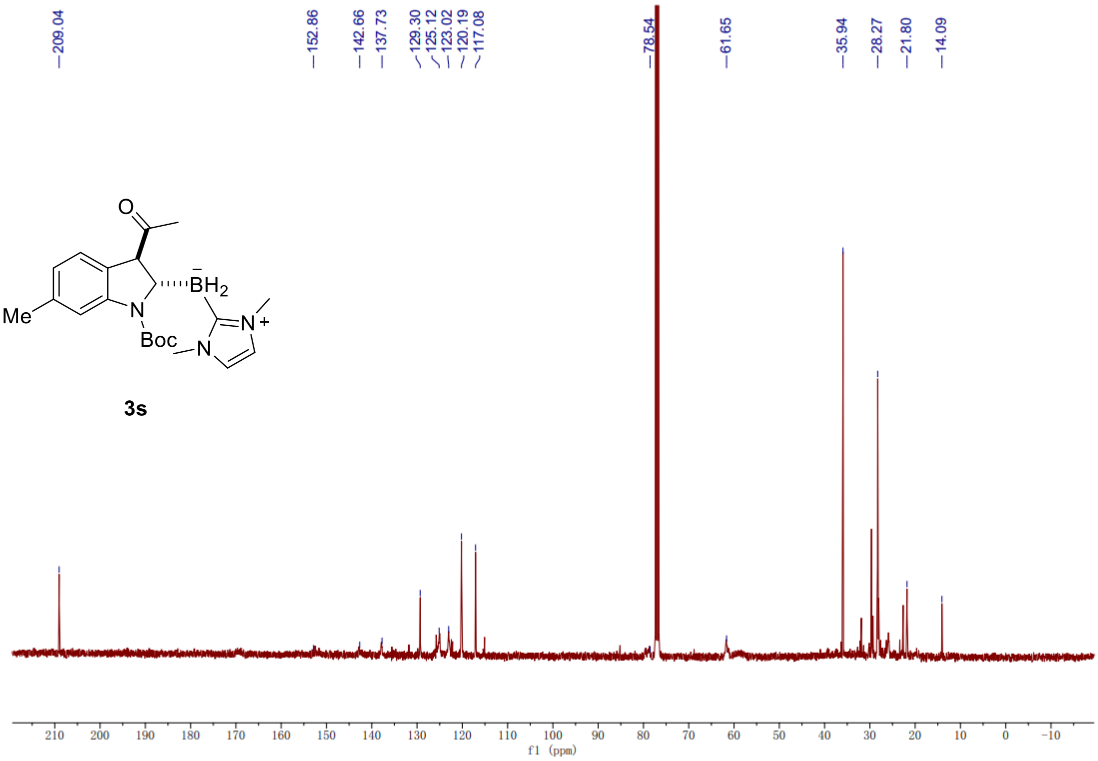

209.04
152.86
142.66
137.73
129.30
125.12
123.02
120.19
117.08
78.54
61.65
35.94
28.27
21.80
14.09
f1 (ppm)

3s

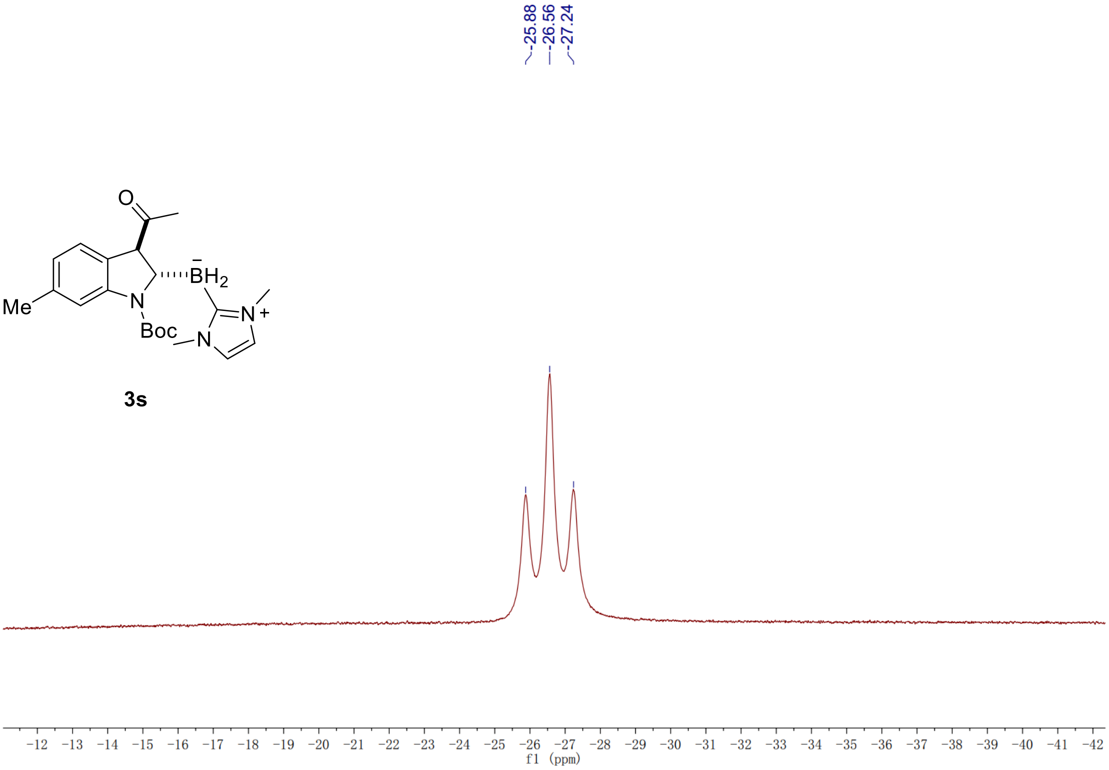

-25.88
-26.56
-27.24
f1 (ppm)

3s

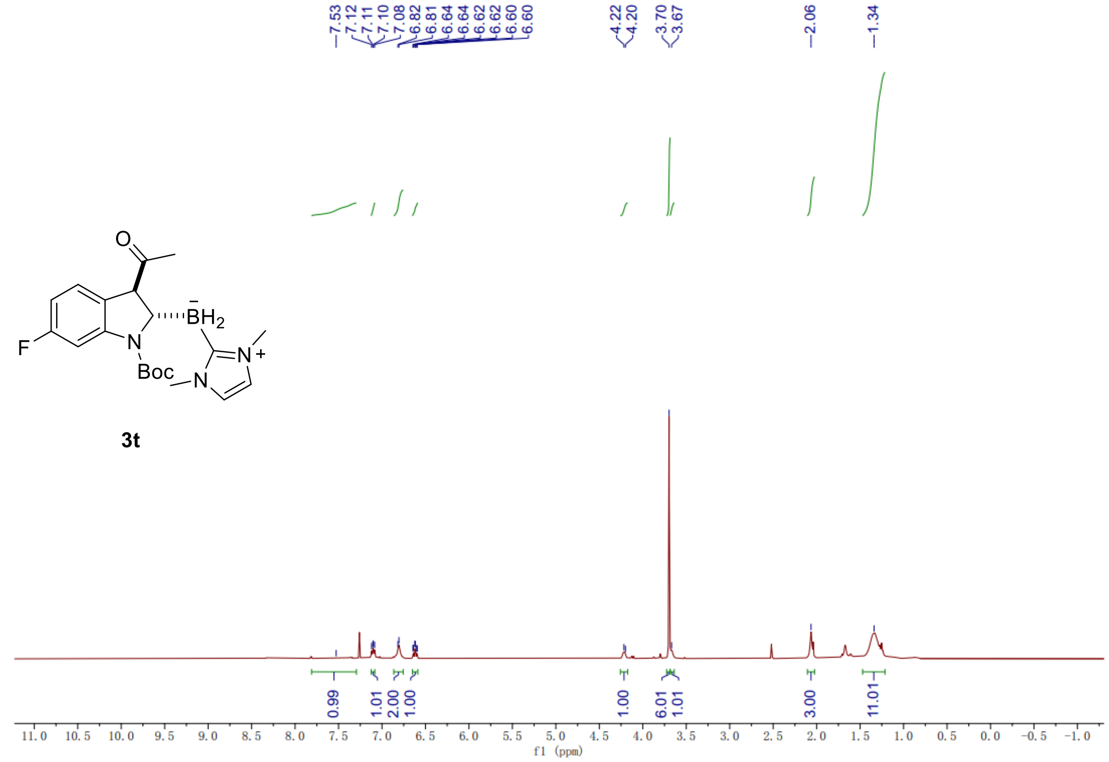
7.53
7.12
7.11
7.10
7.08
6.82
6.81
6.64
6.64
6.62
6.62
6.60
6.60
4.22
4.20
3.70
3.67
2.06
1.34
O
F
N
Boc
BH2
N+
N
0.99
1.01
2.00
1.00
1.00
6.01
1.01
3.00
11.01
f1 (ppm)

3t

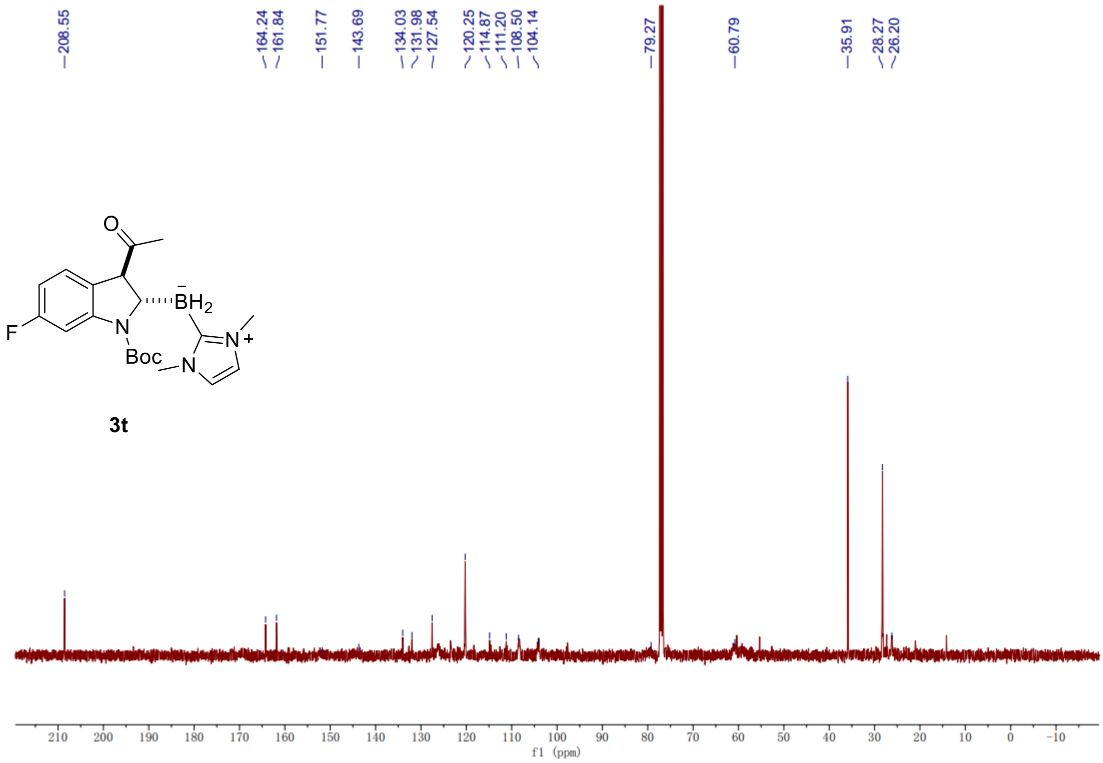
208.55
164.24
161.84
151.77
143.69
134.03
131.98
127.54
120.25
114.87
111.20
108.50
104.14
79.27
60.79
35.91
28.27
26.20
O
F
N
Boc
BH2
N+
N
f1 (ppm)

3t

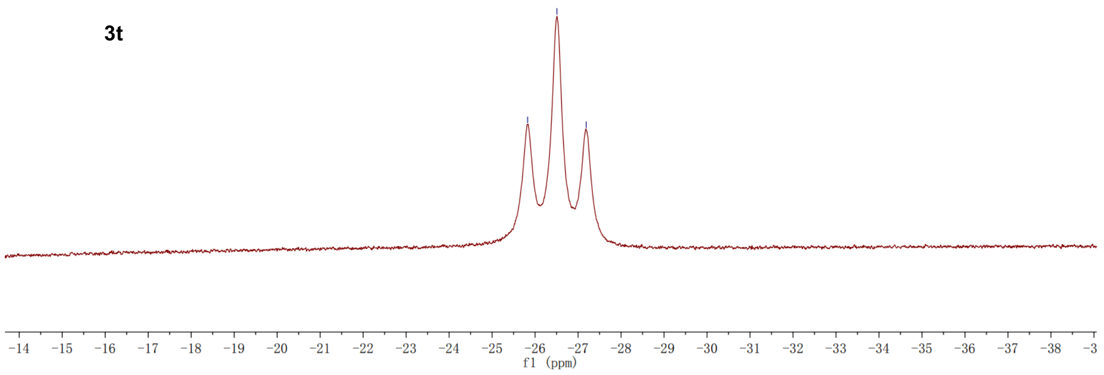
-25.83
-26.51
-27.19
f1 (ppm)

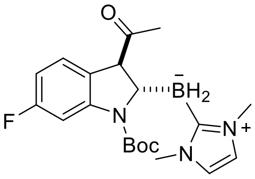
O
F
N
Boc
BH2
N+
N

3t

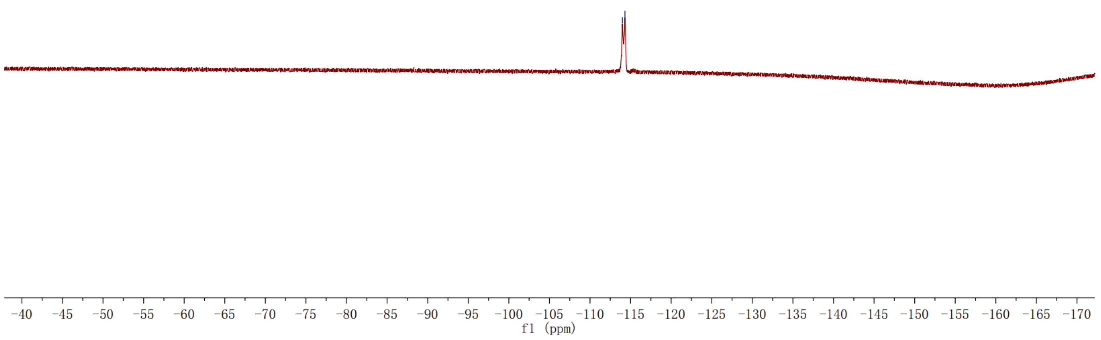
-113.99
-114.30
f1 (ppm)

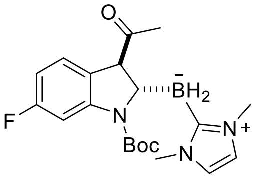
O
F
N
Boc
BH2
N+
N

3t

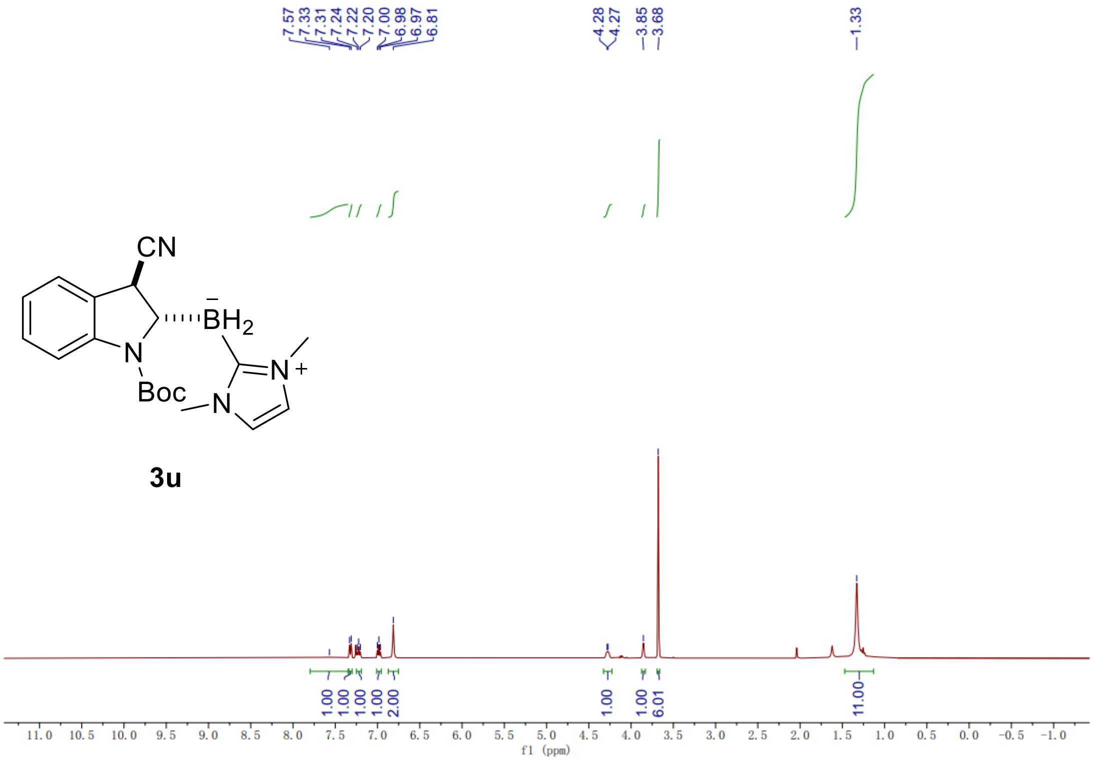
7.57
7.33
7.31
7.24
7.22
7.20
7.00
6.98
6.97
6.81
4.28
4.27
3.85
3.68
1.33
CN
BH2
N
Boc
N+
N
3u
1.00
1.00
1.00
1.00
2.00
1.00
1.00
6.01
11.00
f1 (ppm)

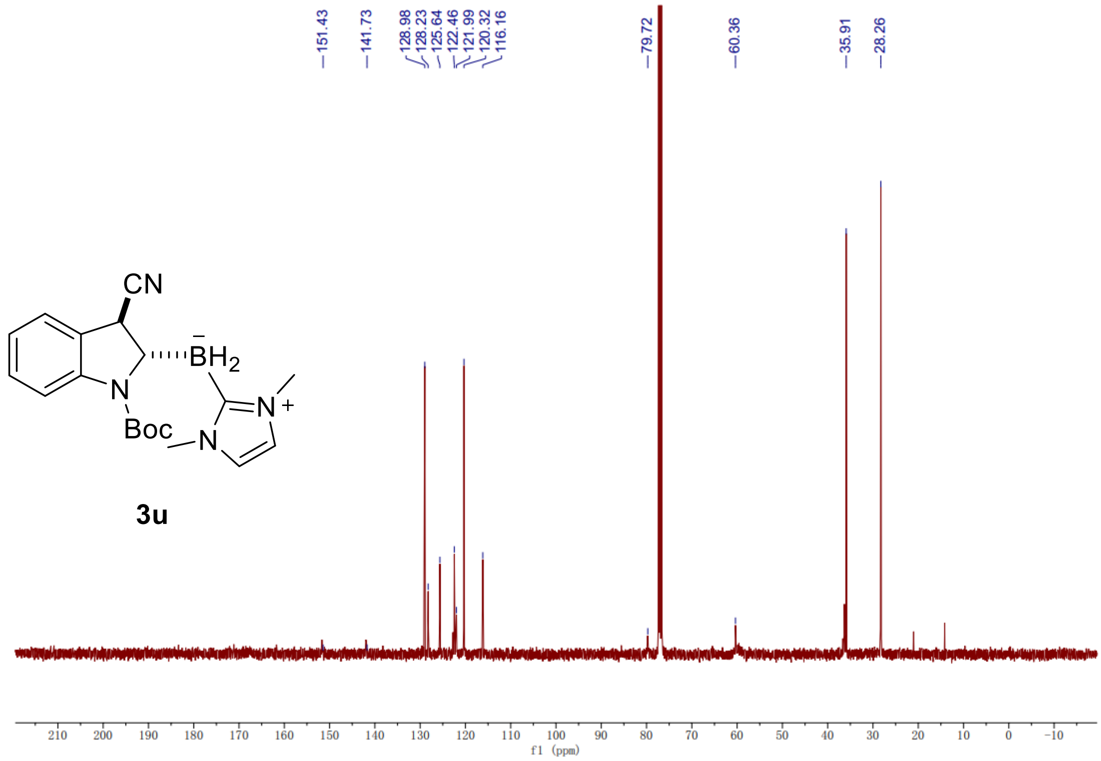
151.43
141.73
128.98
128.23
125.64
122.46
121.99
120.32
116.16
79.72
60.36
35.91
28.26
CN
BH2
N
Boc
N+
N
3u
f1 (ppm)

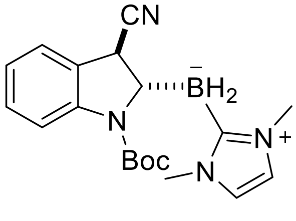

CN
BH2
N
N+
Boc
N

3u

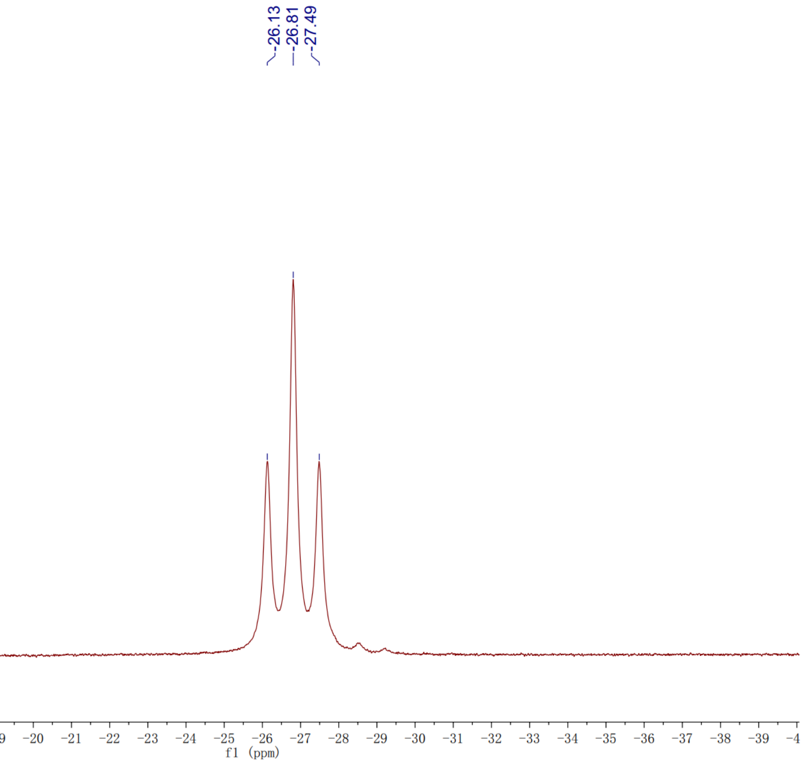

-26.13
-26.81
-27.49
-12 -13 -14 -15 -16 -17 -18 -19 -20 -21 -22 -23 -24 -25 -26 -27 -28 -29 -30 -31 -32 -33 -34 -35 -36 -37 -38 -39
f1 (ppm)

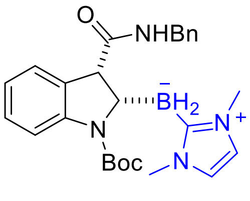

O
NHBn
BH2
N+
N
Boc
N

3v

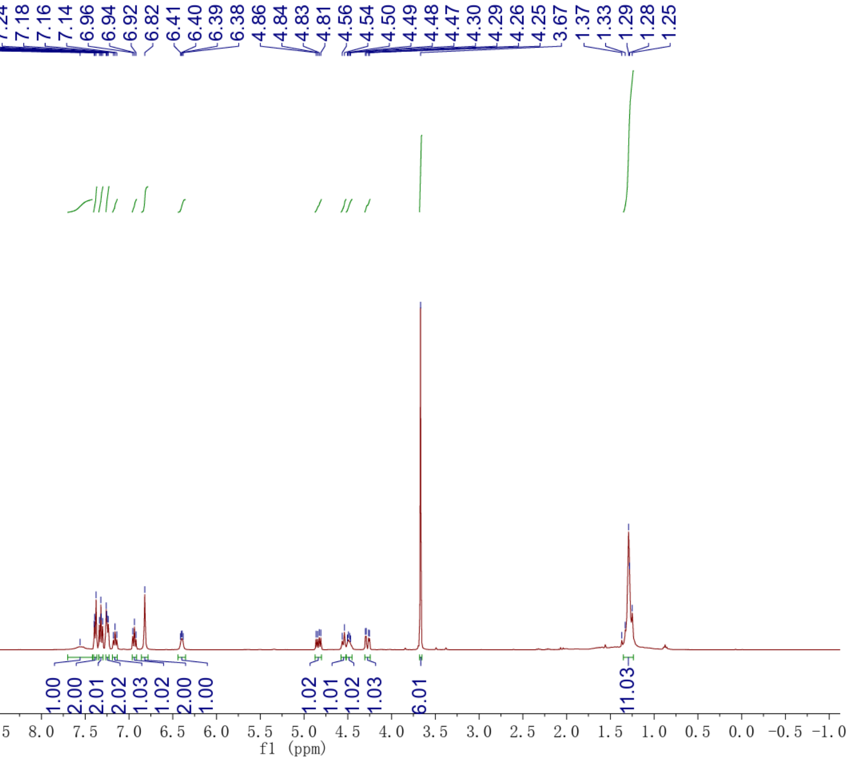

7.56 7.40 7.39 7.37 7.34 7.32 7.32 7.30 7.26 7.25 7.25 7.24 7.18 7.16 7.14 6.96 6.94 6.92 6.82 6.41 6.40 6.39 6.38 4.86 4.84 4.83 4.81 4.56 4.54 4.50 4.49 4.48 4.47 4.30 4.29 4.26 4.25 3.67 1.37 1.33 1.29 1.28 1.25
1.00 2.00 2.01 2.02 1.03 1.02 2.00 1.00 1.02 1.01 1.02 1.03 6.01 11.03
11.0 10.5 10.0 9.5 9.0 8.5 8.0 7.5 7.0 6.5 6.0 5.5 5.0 4.5 4.0 3.5 3.0 2.5 2.0 1.5 1.0 0.5 0.0 -0.5 -1.0
f1 (ppm)

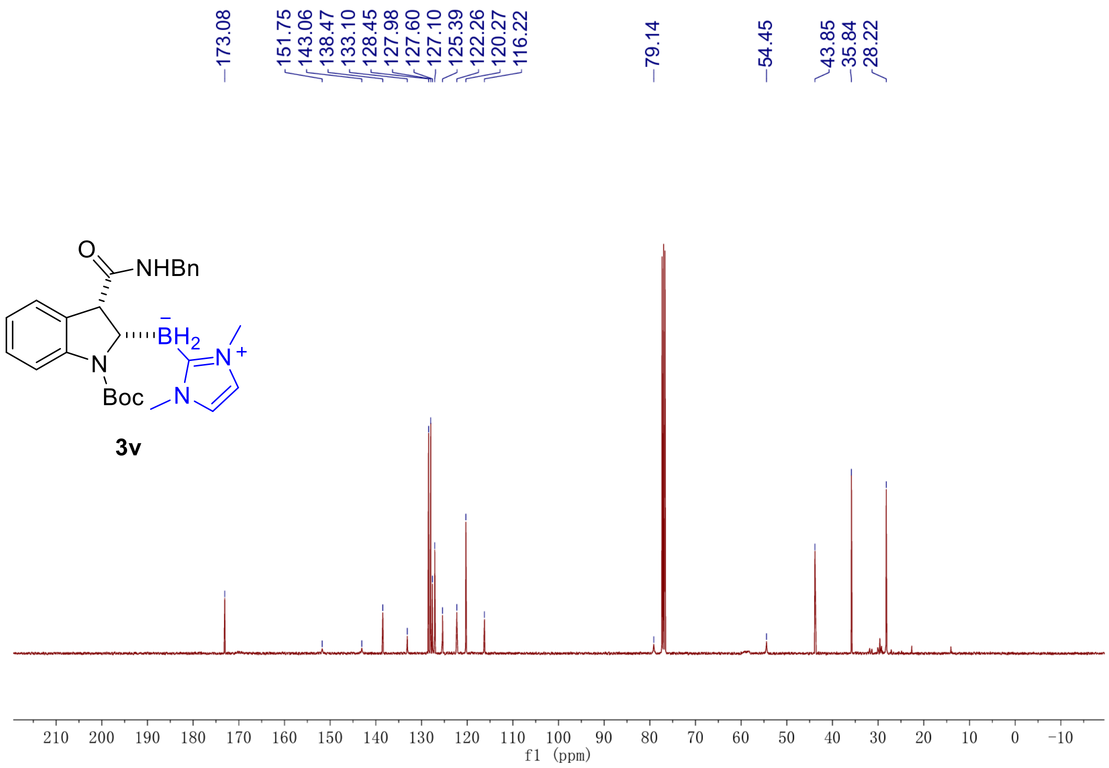
173.08
151.75
143.06
138.47
133.10
128.45
127.98
127.60
127.10
125.39
122.26
120.27
116.22
79.14
54.45
43.85
35.84
28.22
O
NHBn
BH2
N+
N
N
Boc
3v
210 200 190 180 170 160 150 140 130 120 110 100 90 80 70 60 50 40 30 20 10 0 -10
f1 (ppm)

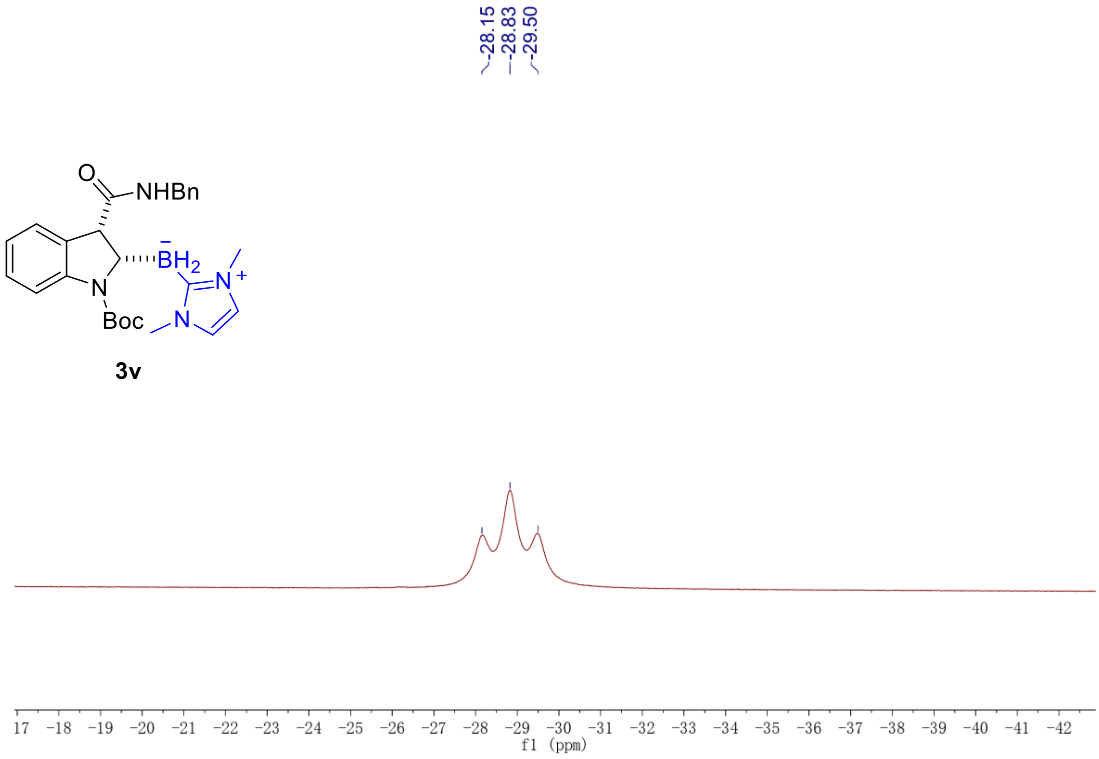
-28.15
-28.83
-29.50
O
NHBn
BH2
N+
N
N
Boc
3v
17 -18 -19 -20 -21 -22 -23 -24 -25 -26 -27 -28 -29 -30 -31 -32 -33 -34 -35 -36 -37 -38 -39 -40 -41 -42
f1 (ppm)

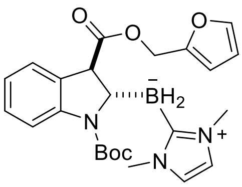

3w

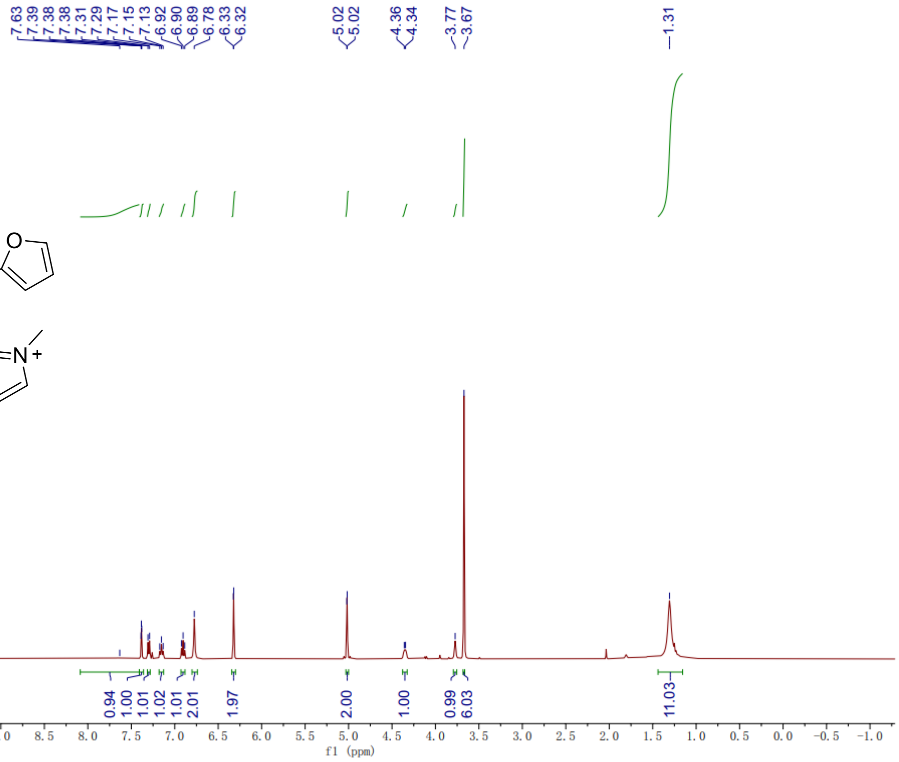

7.63
7.39
7.38
7.38
7.31
7.29
7.17
7.15
7.13
6.92
6.90
6.89
6.78
6.33
6.32
5.02
5.02
4.36
4.34
3.77
3.67
1.31
0.94
1.00
1.01
1.02
1.01
2.01
1.97
2.00
1.00
0.99
6.03
11.03
f1 (ppm)

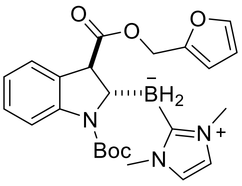

3w

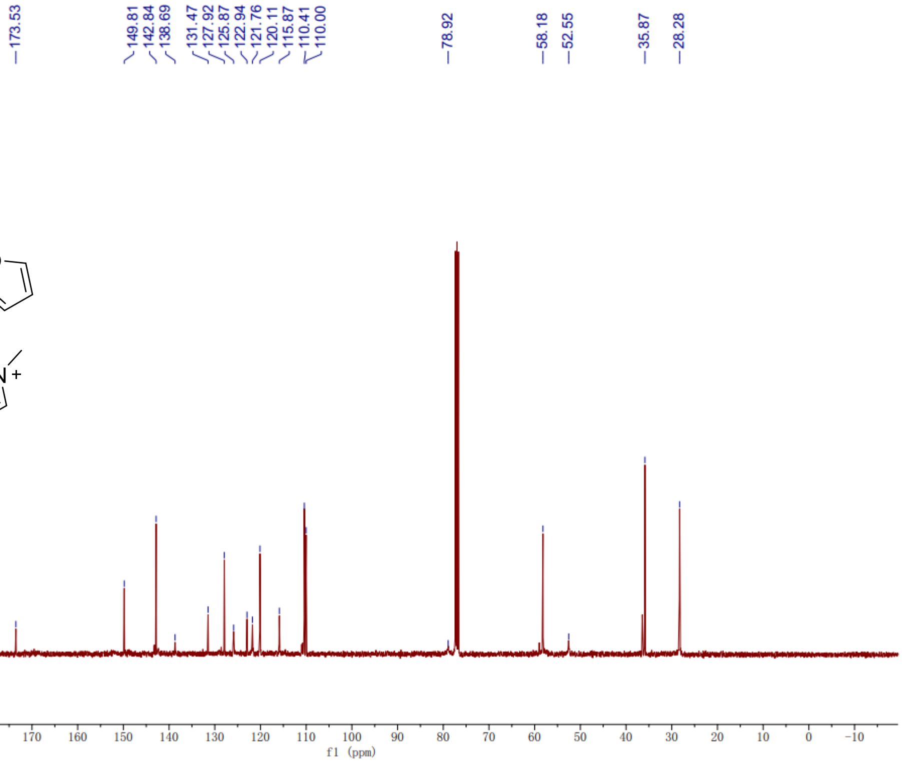

173.53
149.81
142.84
138.69
131.47
127.92
125.87
122.94
121.76
120.11
115.87
110.41
110.00
78.92
58.18
52.55
35.87
28.28
f1 (ppm)

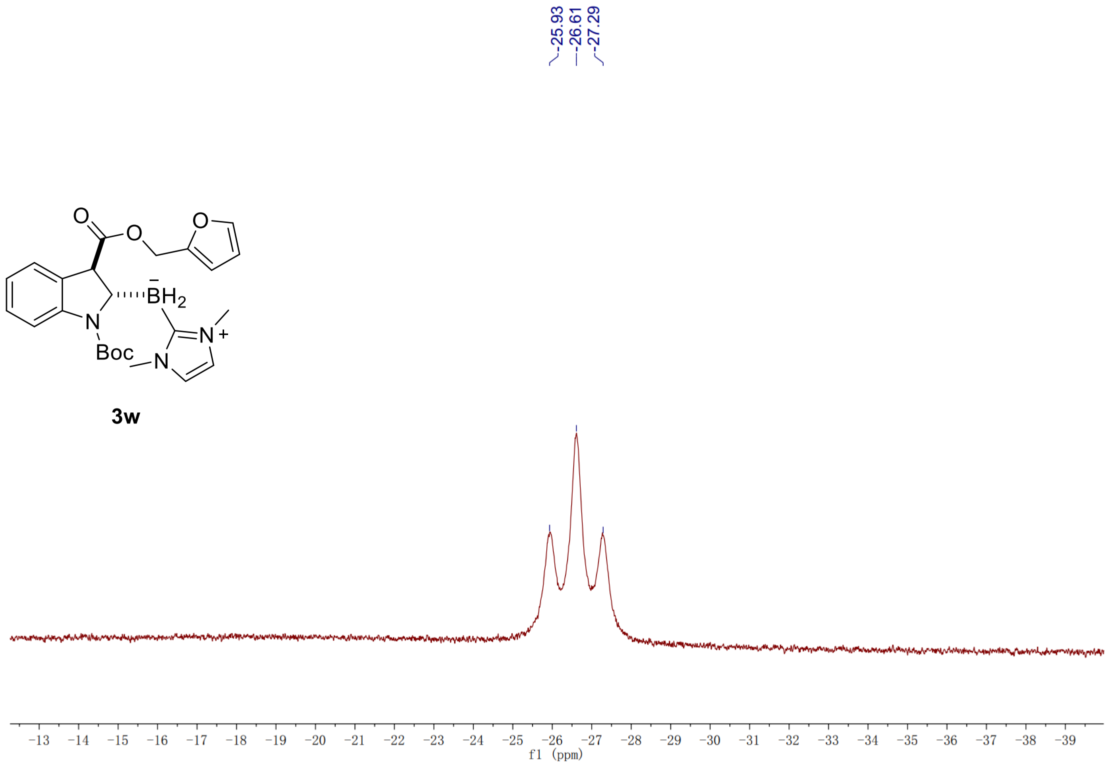
-25.93
-26.61
-27.29
O
O
O
BH2
N
Boc
N+
N
f1 (ppm)

3w

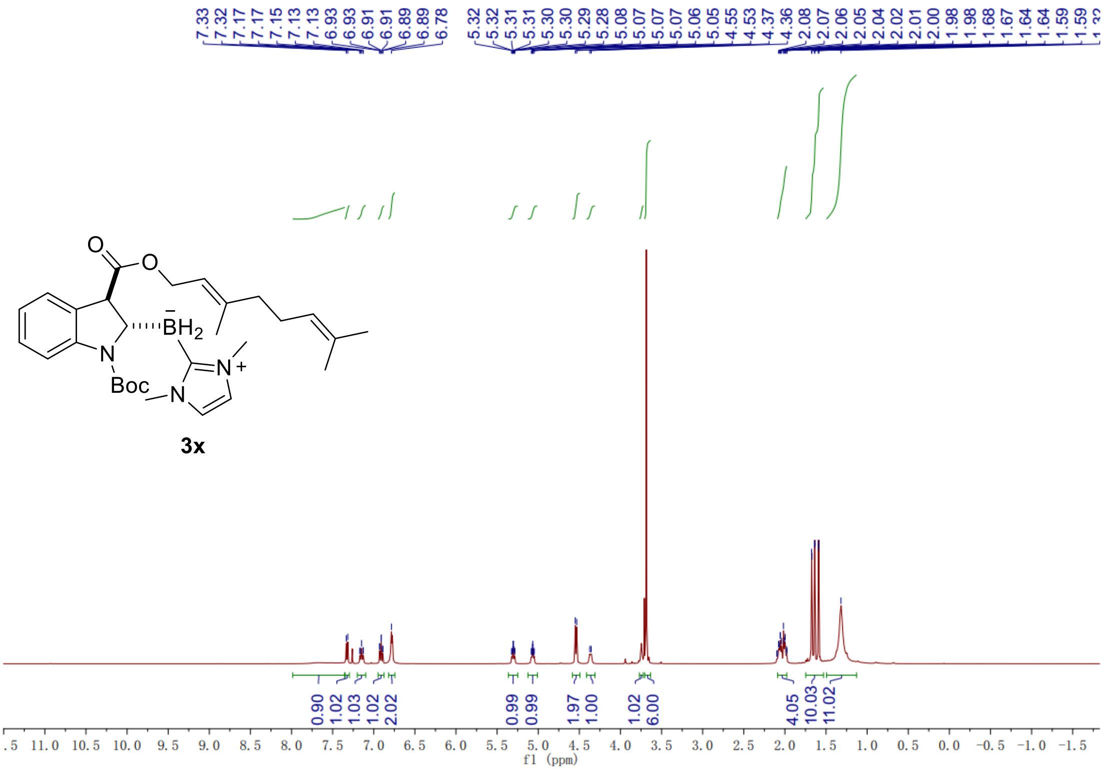
7.33
7.32
7.17
7.15
7.13
6.93
6.93
6.91
6.91
6.89
6.89
6.78
5.32
5.32
5.31
5.31
5.30
5.30
5.29
5.28
5.08
5.07
5.07
5.06
5.05
4.55
4.53
4.37
4.36
2.08
2.07
2.06
2.05
2.04
2.02
2.01
2.00
1.98
1.68
1.67
1.64
1.64
1.59
O
O
BH2
N
Boc
N+
N
0.90
1.02
1.03
1.02
2.02
0.99
0.99
1.97
1.00
1.02
6.00
4.05
10.03
11.02
f1 (ppm)

3x

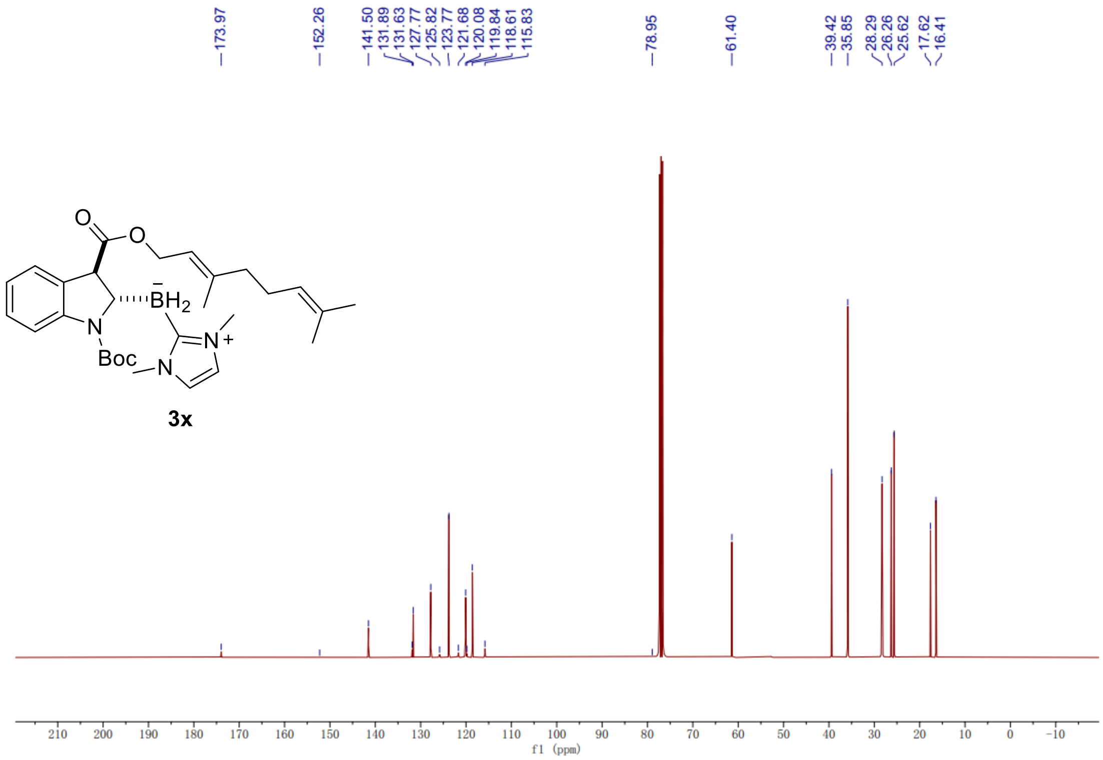
173.97
152.26
141.50
131.89
131.63
127.77
125.82
123.77
121.68
120.08
119.84
118.61
115.83
78.95
61.40
39.42
35.85
28.29
26.26
25.62
17.62
16.41
O
O
BH2
N
N+
Boc
N
3x
210 200 190 180 170 160 150 140 130 120 110 100 90 80 70 60 50 40 30 20 10 0 -10
f1 (ppm)

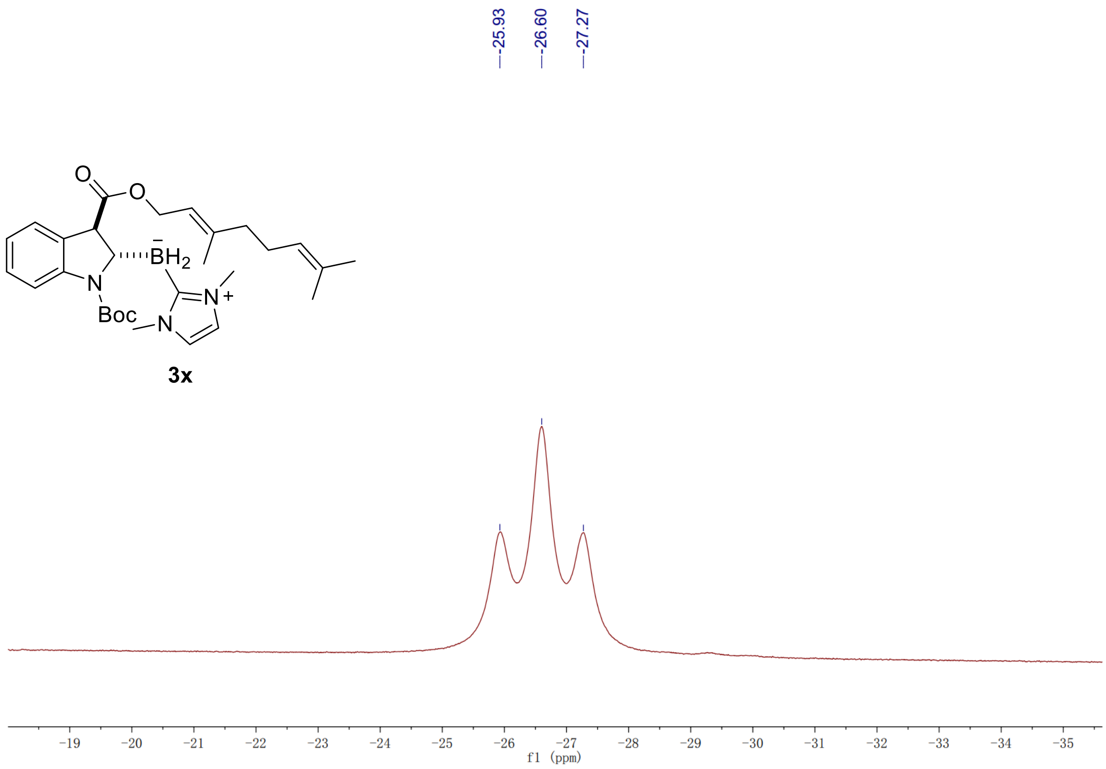
-25.93
-26.60
-27.27
O
O
BH2
N
N+
Boc
N
3x
-19 -20 -21 -22 -23 -24 -25 -26 -27 -28 -29 -30 -31 -32 -33 -34 -35
f1 (ppm)

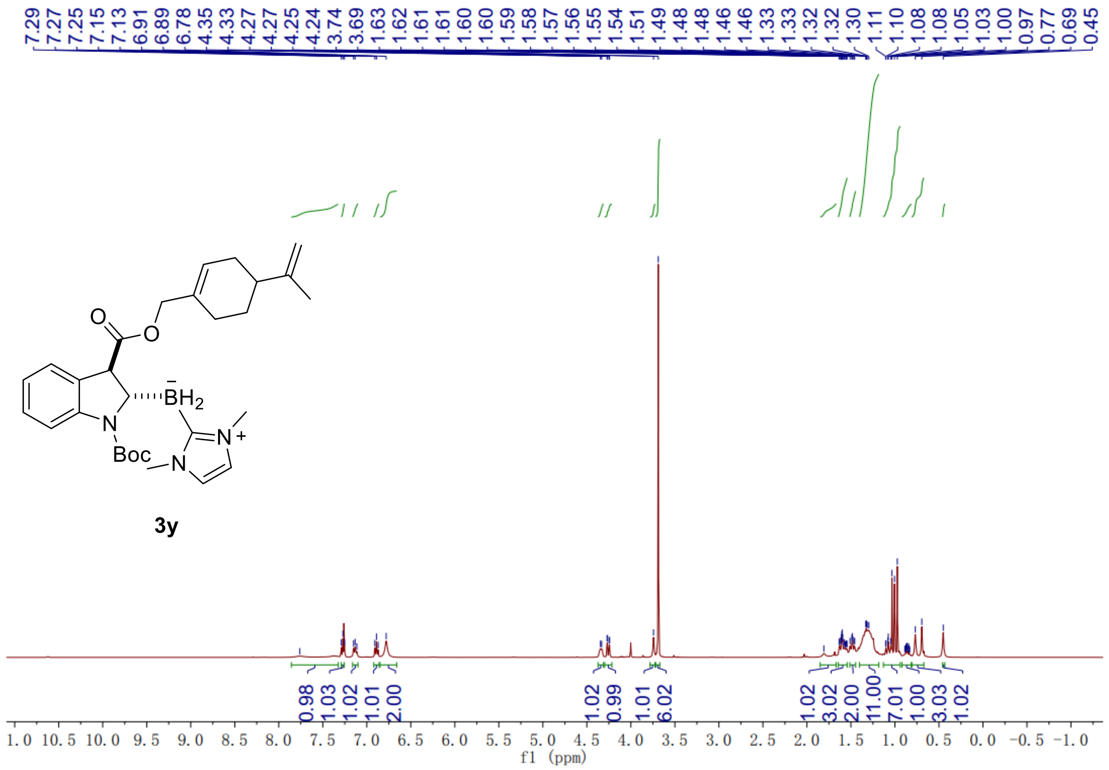

7.29 7.27 7.25 7.15 7.13 6.91 6.89 6.78 4.35 4.33 4.27 4.27 4.25 4.24 3.74 3.69 1.63 1.62 1.61 1.61 1.60 1.60 1.59 1.58 1.57 1.56 1.55 1.54 1.51 1.49 1.48 1.48 1.46 1.46 1.33 1.33 1.32 1.32 1.30 1.11 1.10 1.08 1.05 1.03 1.00 0.97 0.77 0.69 0.45
0.98 1.03 1.02 1.01 2.00 1.02 0.99 1.01 6.02 1.02 3.02 2.00 11.00 7.01 1.00 3.03 1.02
BH2
N
Boc
O
3y
f1 (ppm)

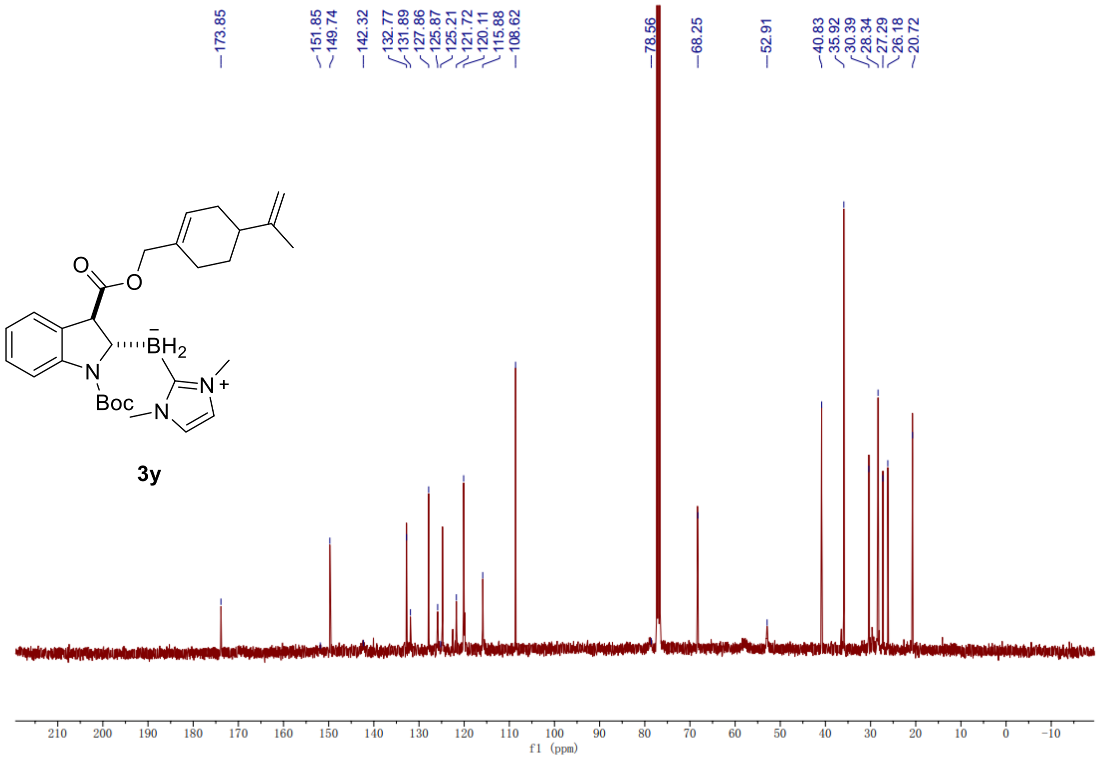

173.85 151.85 149.74 142.32 132.77 131.89 127.86 125.87 125.21 121.72 120.11 115.88 108.62 78.56 68.25 52.91 40.83 35.92 30.39 28.34 27.29 26.18 20.72
BH2
N
Boc
O
3y
f1 (ppm)

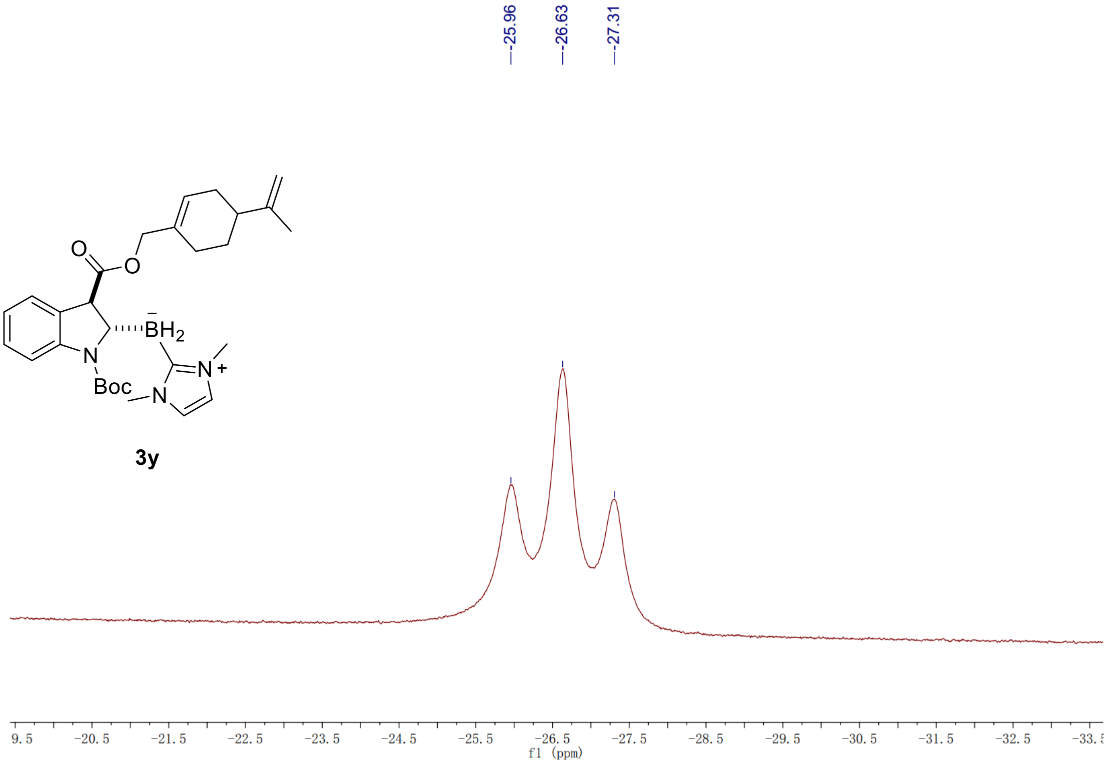

-25.96
-26.63
-27.31
f1 (ppm)

3y

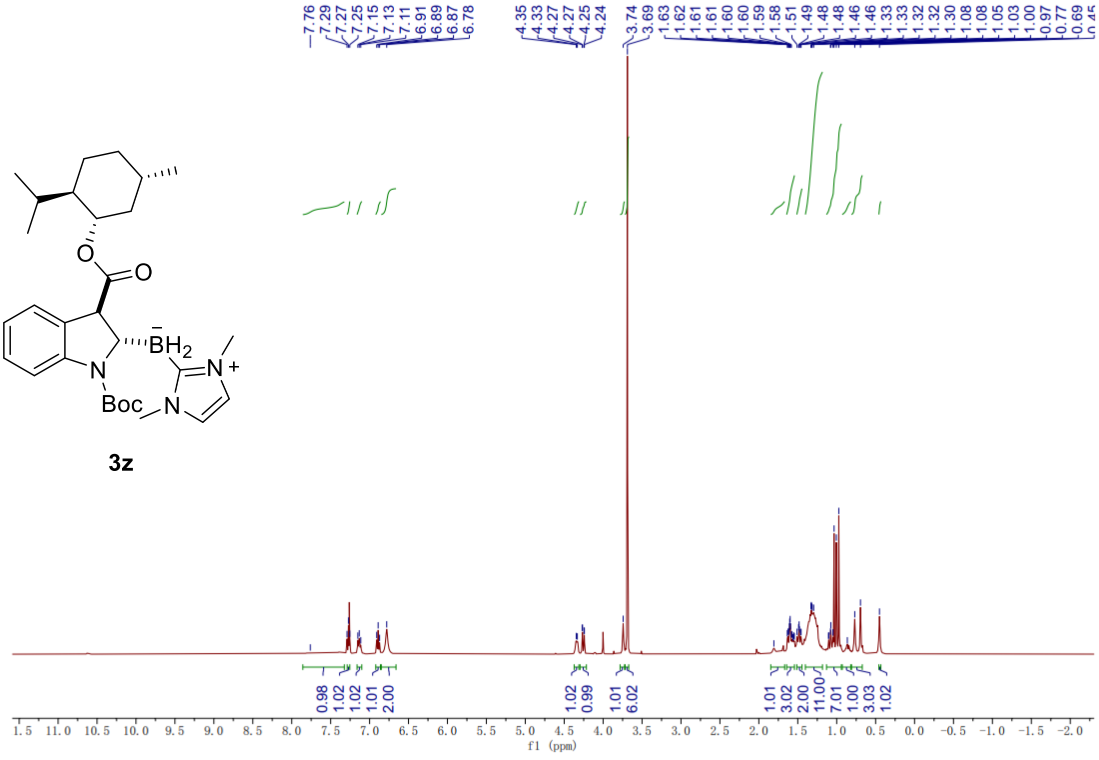

7.76
7.29
7.27
7.25
7.15
7.13
7.11
6.91
6.89
6.87
6.78
4.35
4.33
4.27
4.27
4.25
4.24
3.74
3.69
1.63
1.62
1.61
1.61
1.60
1.60
1.59
1.58
1.51
1.49
1.48
1.48
1.46
1.46
1.33
1.33
1.32
1.32
1.30
1.08
1.08
1.05
1.03
1.00
0.97
0.77
0.69
0.98
1.02
1.02
1.01
2.00
1.02
0.99
1.01
6.02
1.01
3.02
2.00
11.00
7.01
1.00
3.03
1.02
f1 (ppm)

3z

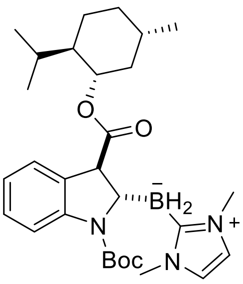

3z

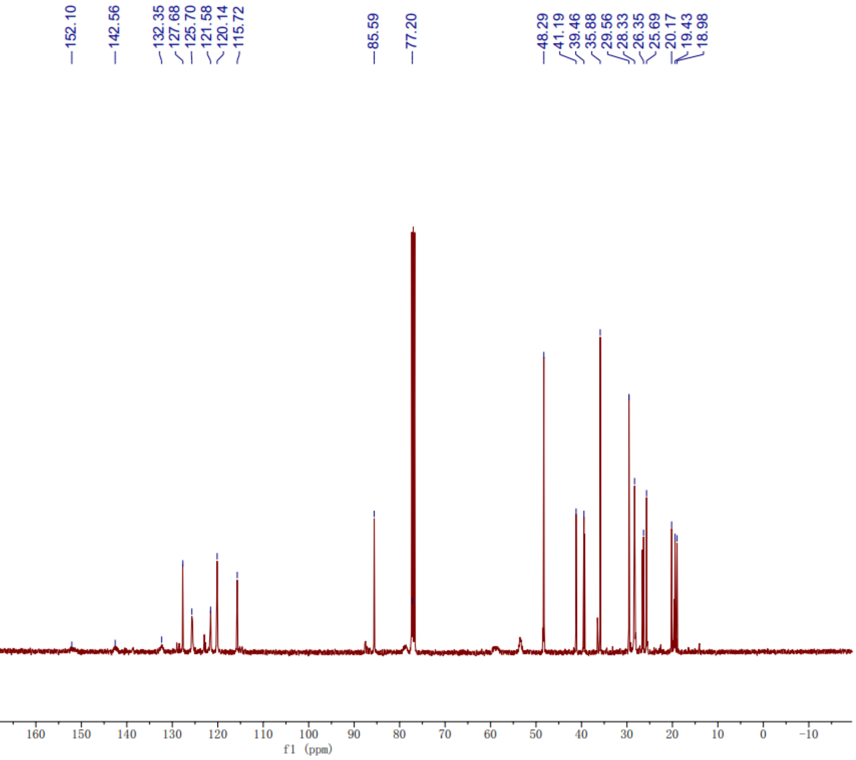
174.48
152.10
142.56
132.35
127.68
125.70
121.58
120.14
115.72
85.59
77.20
48.29
41.19
39.46
35.88
29.56
28.33
26.35
25.69
20.17
19.43
18.98
210 200 190 180 170 160 150 140 130 120 110 100 90 80 70 60 50 40 30 20 10 0 -10
f1 (ppm)

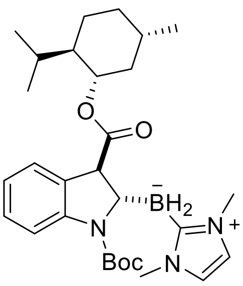

3z

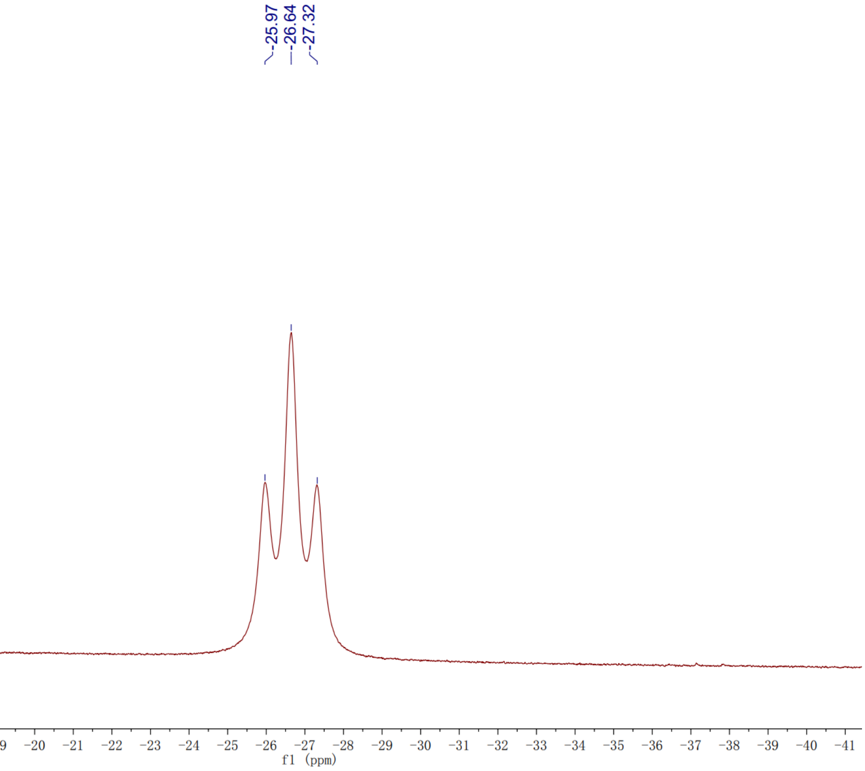
-25.97
-26.64
-27.32
-13 -14 -15 -16 -17 -18 -19 -20 -21 -22 -23 -24 -25 -26 -27 -28 -29 -30 -31 -32 -33 -34 -35 -36 -37 -38 -39 -40 -41
f1 (ppm)

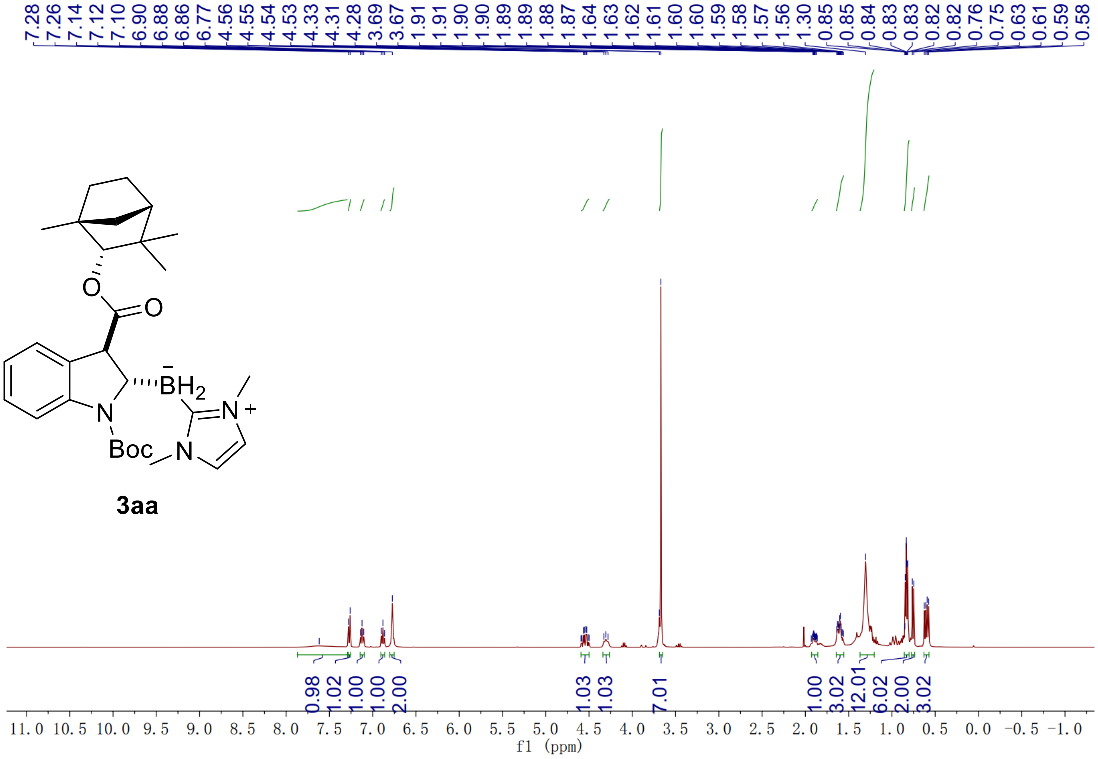
7.28 7.26 7.14 7.12 7.10 6.90 6.88 6.86 6.77 4.56 4.55 4.54 4.53 4.33 4.31 4.28 3.69 3.67 1.91 1.91 1.90 1.90 1.89 1.89 1.88 1.87 1.64 1.63 1.62 1.61 1.60 1.60 1.59 1.58 1.57 1.56 1.30 0.85 0.85 0.84 0.83 0.83 0.82 0.82 0.76 0.75 0.63 0.61 0.59 0.58
BH2
N+
N
Boc
N
O
O
3aa
0.98 1.02 1.00 1.00 2.00 1.03 1.03 7.01 1.00 3.02 12.01 6.02 2.00 3.02
11.0 10.5 10.0 9.5 9.0 8.5 8.0 7.5 7.0 6.5 6.0 5.5 5.0 4.5 4.0 3.5 3.0 2.5 2.0 1.5 1.0 0.5 0.0 -0.5 -1.0
f1 (ppm)

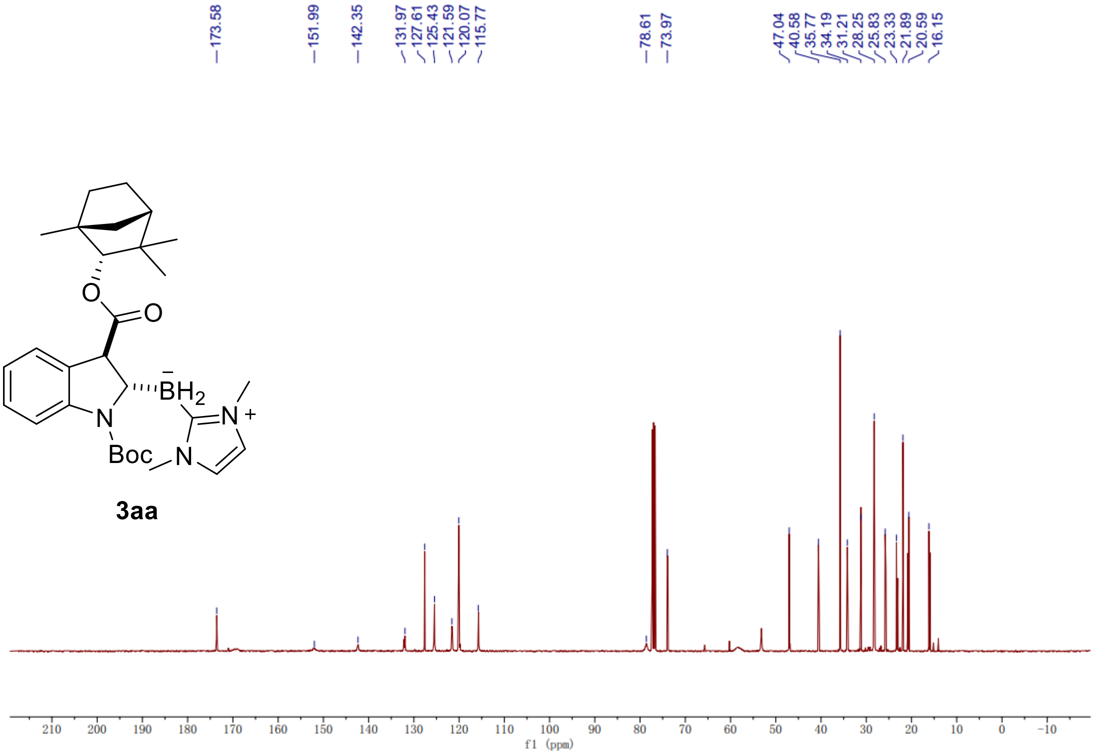
173.58 151.99 142.35 131.97 127.61 125.43 121.59 120.07 115.77 78.61 73.97 47.04 40.58 35.77 34.19 31.21 28.25 25.83 23.33 21.89 20.59 16.15
BH2
N+
N
Boc
N
O
O
3aa
210 200 190 180 170 160 150 140 130 120 110 100 90 80 70 60 50 40 30 20 10 0 -10
f1 (ppm)

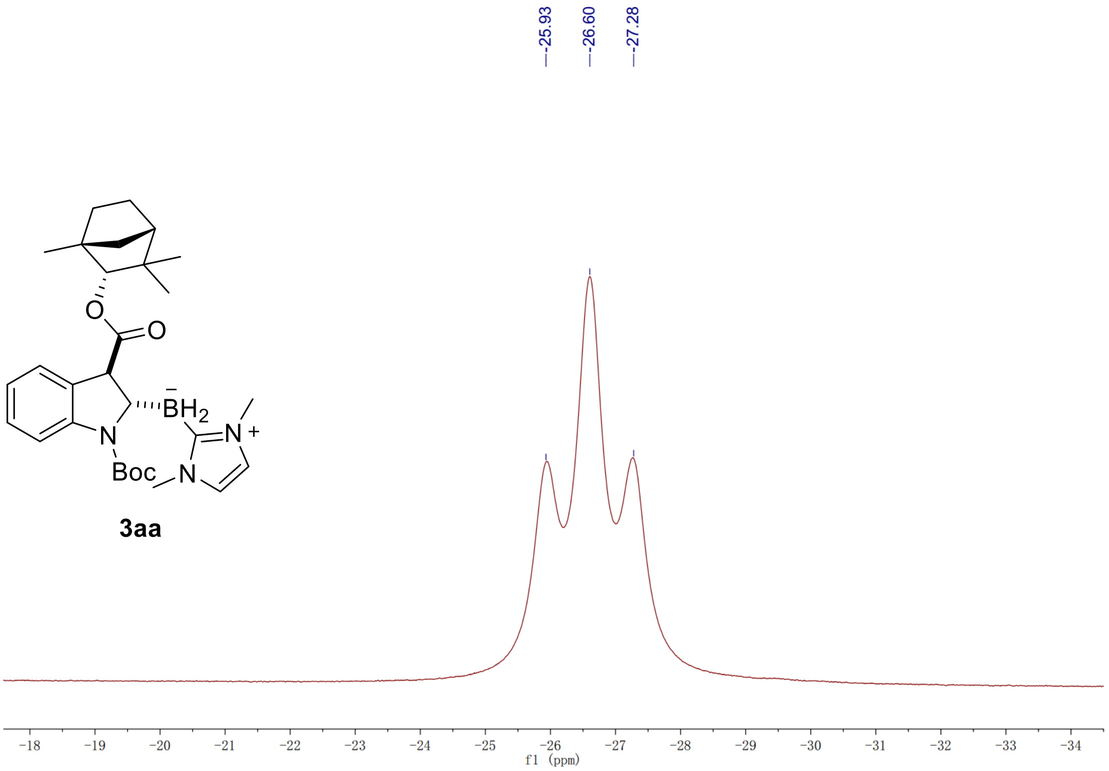

3aa

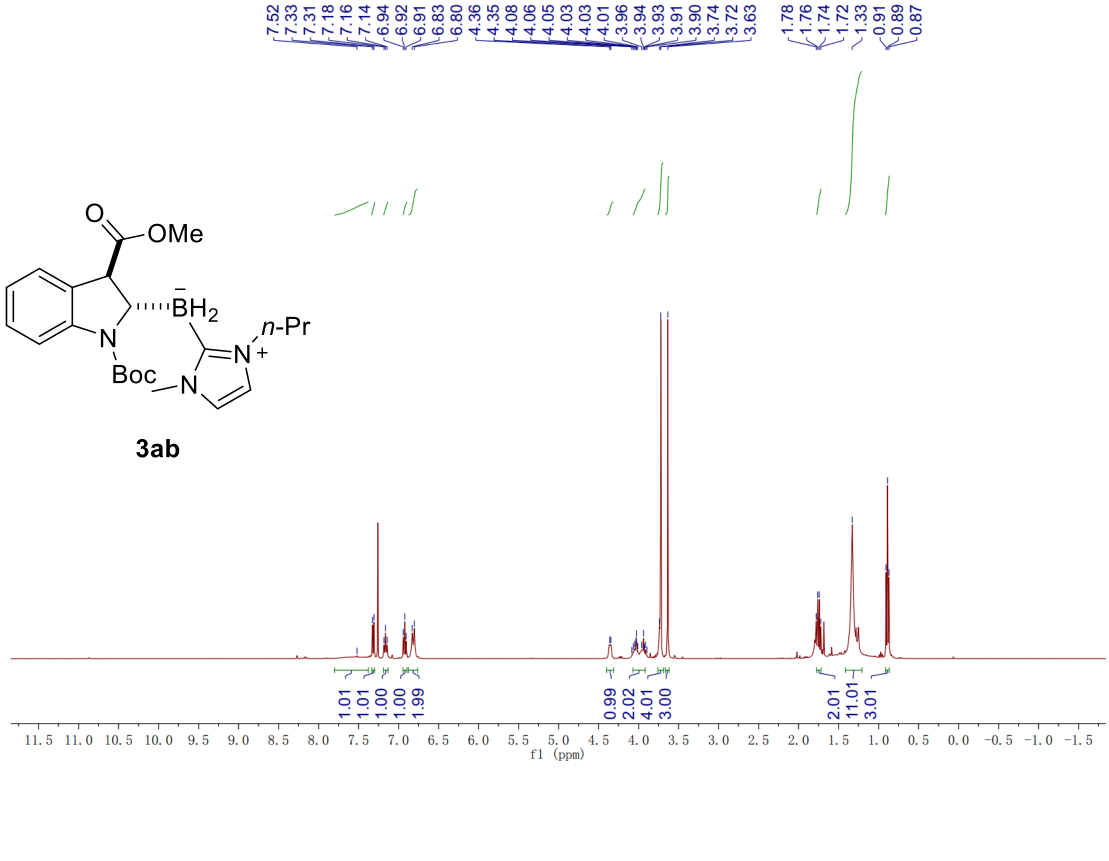

3ab

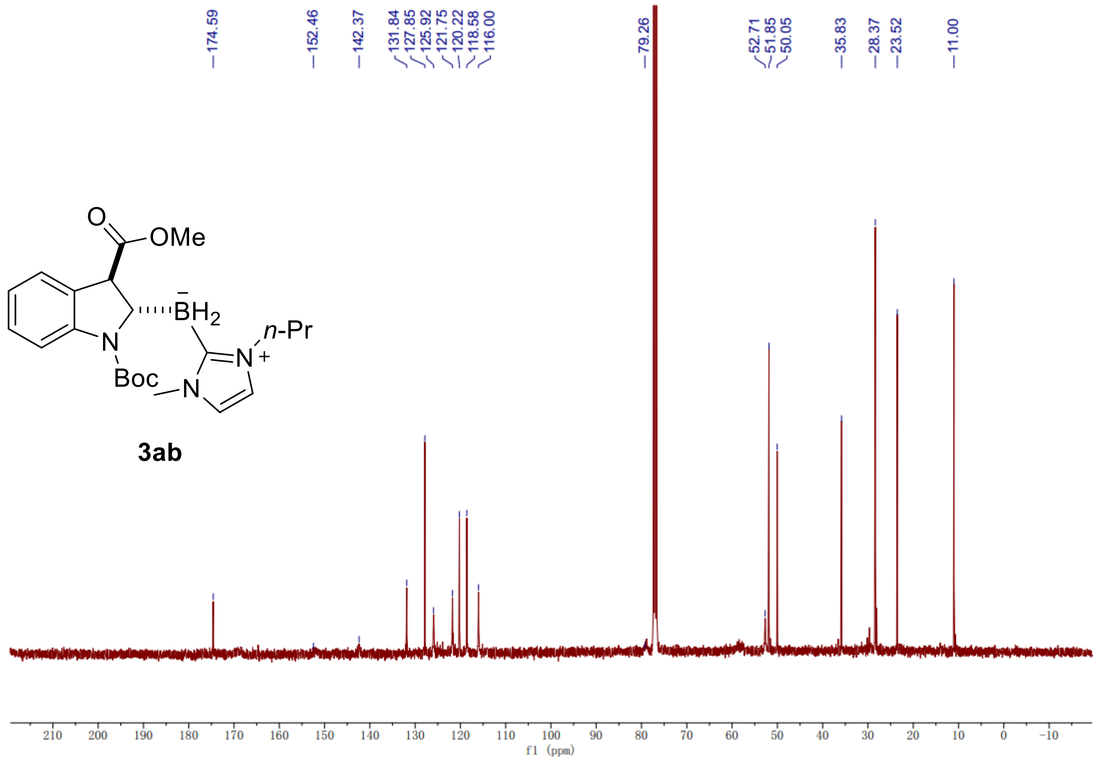
174.59
152.46
142.37
131.84
127.85
125.92
121.75
120.22
118.58
116.00
79.26
52.71
51.85
50.05
35.83
28.37
23.52
11.00
O
OMe
BH2
N
n-Pr
N+
Boc
N
3ab
210 200 190 180 170 160 150 140 130 120 110 100 90 80 70 60 50 40 30 20 10 0 -10
f1 (ppm)

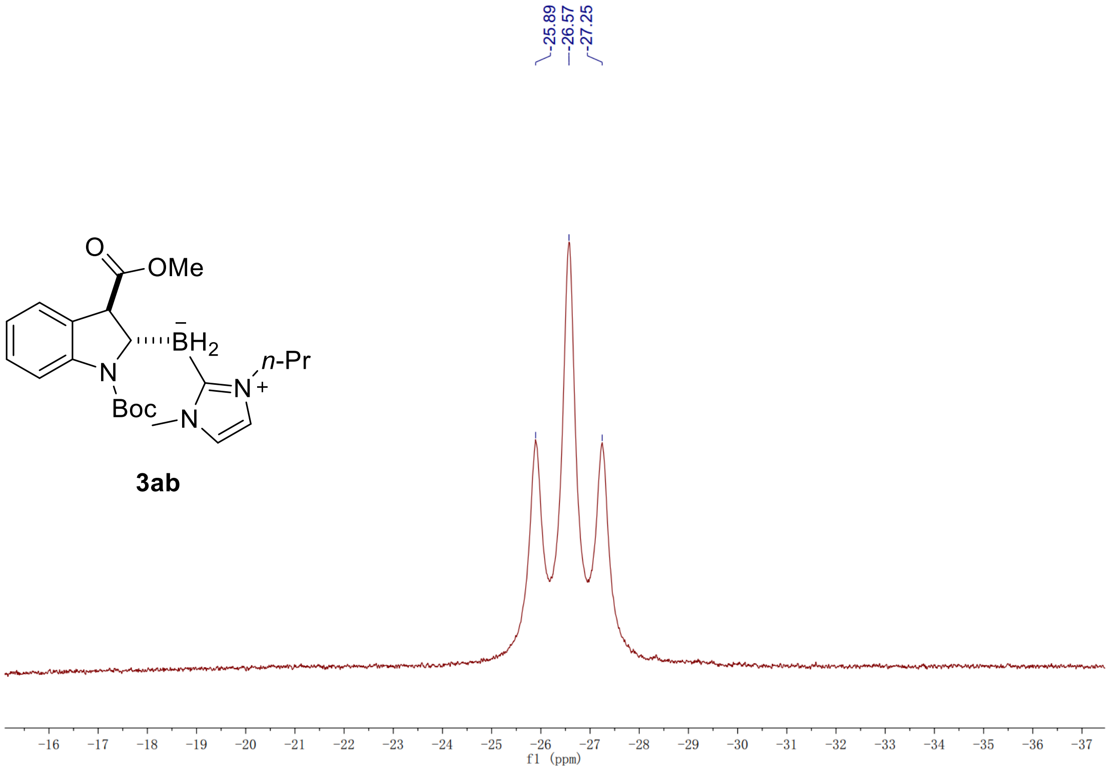
-25.89
-26.57
-27.25
O
OMe
BH2
N
n-Pr
N+
Boc
N
3ab
-16 -17 -18 -19 -20 -21 -22 -23 -24 -25 -26 -27 -28 -29 -30 -31 -32 -33 -34 -35 -36 -37
f1 (ppm)

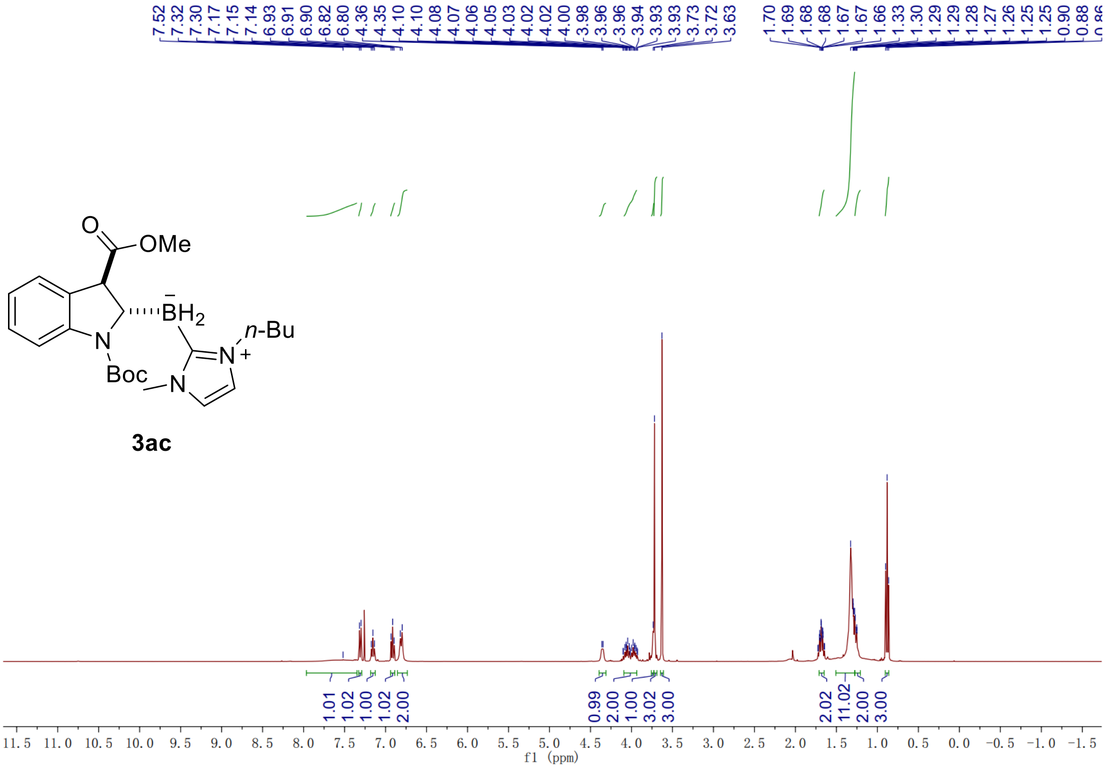
7.52 7.32 7.30 7.17 7.15 7.14 6.93 6.91 6.90 6.82 6.80 4.36 4.35 4.10 4.10 4.08 4.07 4.06 4.05 4.03 4.02 4.02 4.00 3.98 3.96 3.96 3.94 3.93 3.93 3.73 3.72 3.63
1.70 1.69 1.68 1.68 1.67 1.67 1.66 1.33 1.30 1.29 1.29 1.28 1.27 1.26 1.25 1.25 0.90 0.88 [illegible]
OMe
O
BH2
n-Bu
N
N+
Boc
N
3ac
1.01 1.02 1.00 1.02 2.00 0.99 2.00 1.00 3.02 3.00 2.02 11.02 2.00 3.00
11.5 11.0 10.5 10.0 9.5 9.0 8.5 8.0 7.5 7.0 6.5 6.0 5.5 5.0 4.5 4.0 3.5 3.0 2.5 2.0 1.5 1.0 0.5 0.0 -0.5 -1.0 -1.5
f1 (ppm)

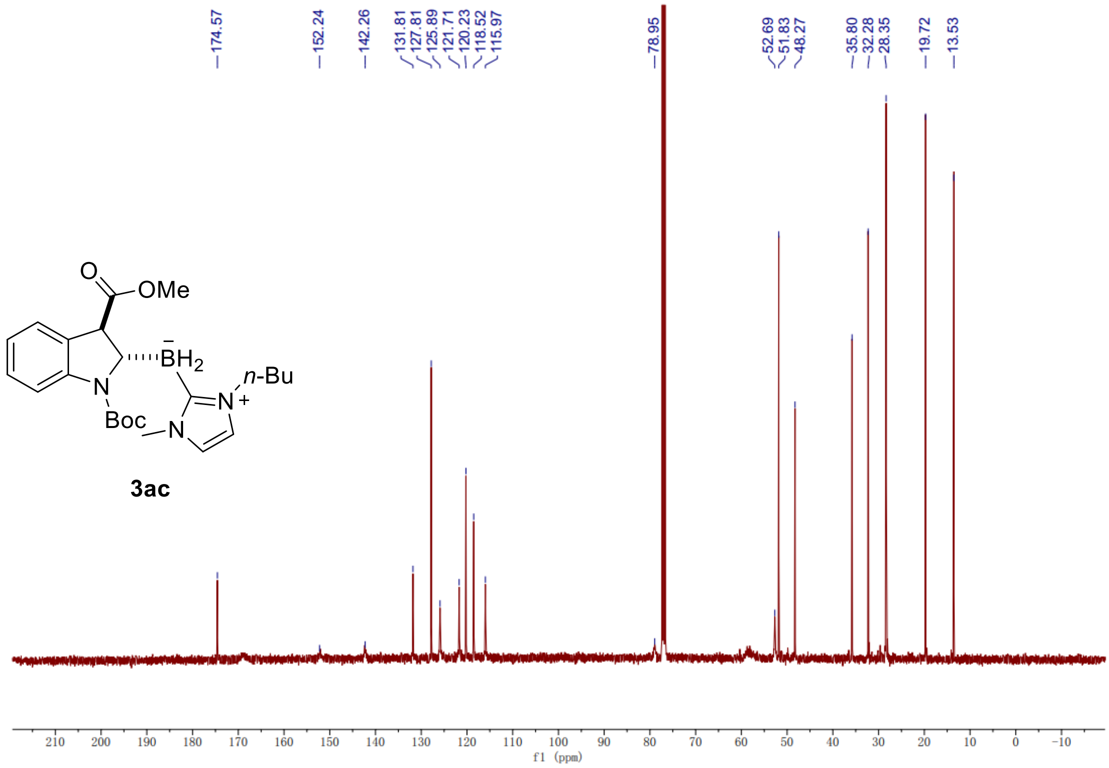
174.57
152.24
142.26
131.81 127.81 125.89 121.71 120.23 118.52 115.97
78.95
52.69 51.83 48.27
35.80 32.28 28.35
19.72
13.53
OMe
O
BH2
n-Bu
N
N+
Boc
N
3ac
210 200 190 180 170 160 150 140 130 120 110 100 90 80 70 60 50 40 30 20 10 0 -10
f1 (ppm)

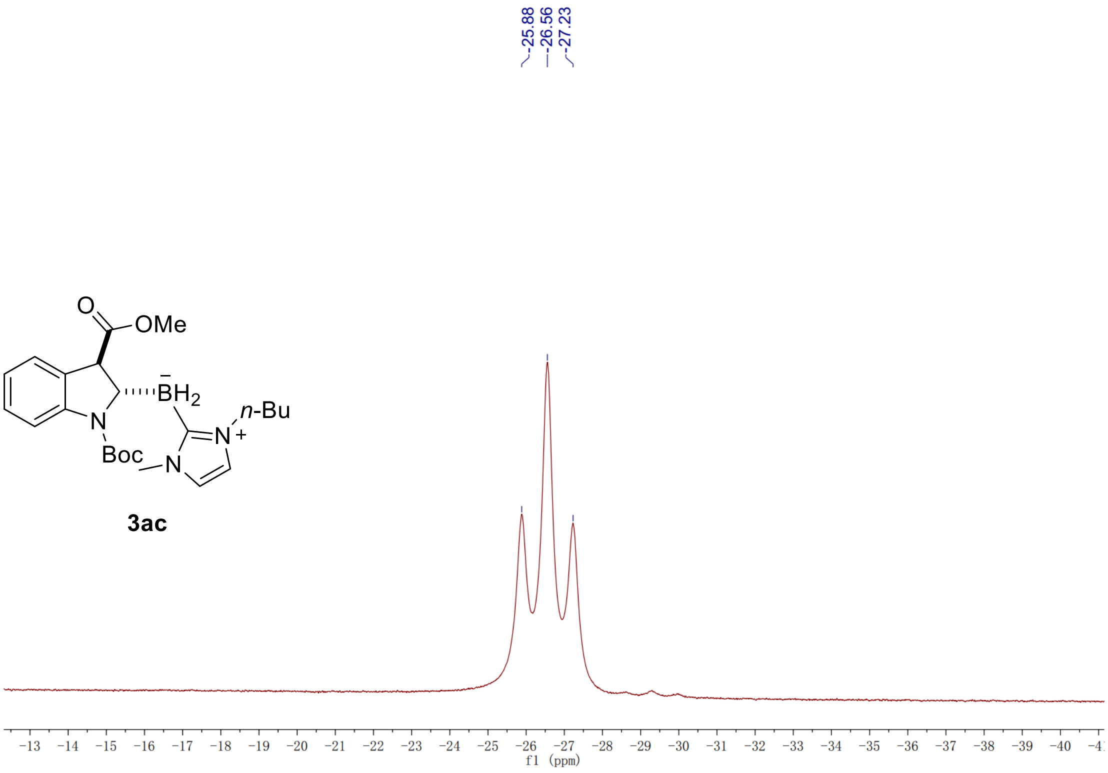
-25.88
-26.56
-27.23
OMe
O
BH2
n-Bu
N+
N
Boc
N
f1 (ppm)

3ac

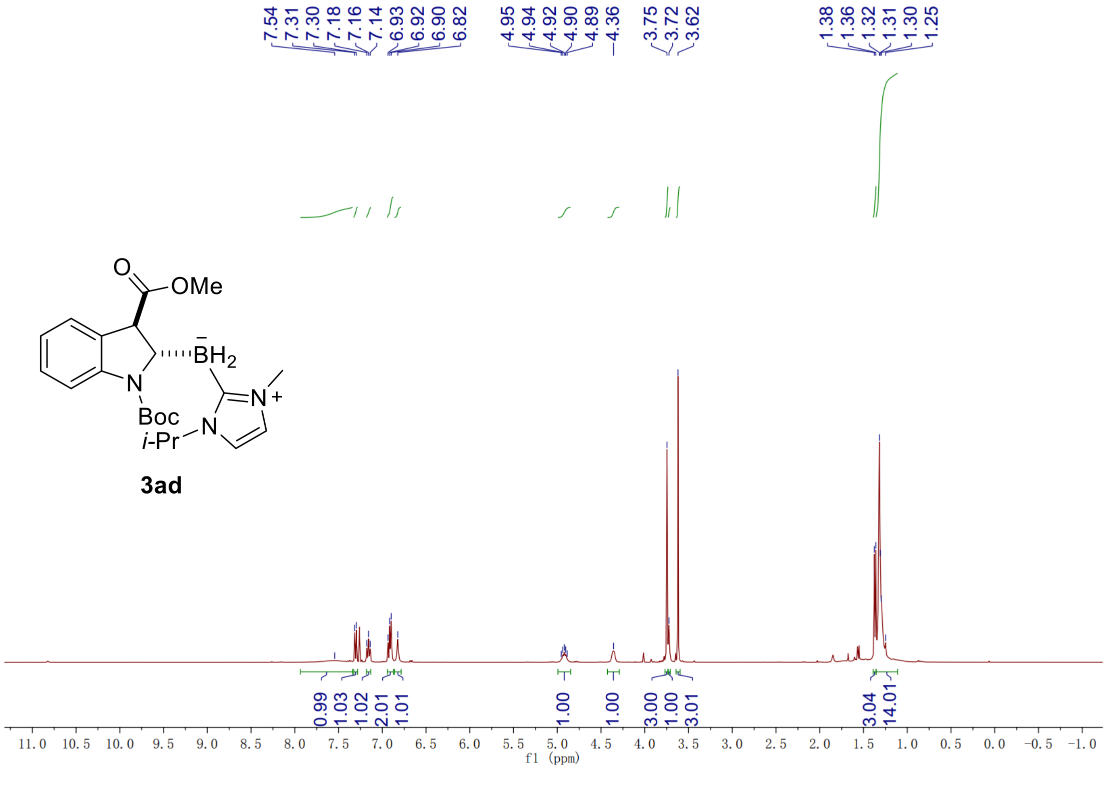
7.54 7.31 7.30 7.18 7.16 7.14 6.93 6.92 6.90 6.82
4.95 4.94 4.92 4.90 4.89 4.36
3.75 3.72 3.62
1.38 1.36 1.32 1.31 1.30 1.25
OMe
O
BH2
N+
N
Boc
i-Pr
N
0.99 1.03 1.02 2.01 1.01 1.00 1.00 3.00 1.00 3.01 3.04 14.01
f1 (ppm)

3ad

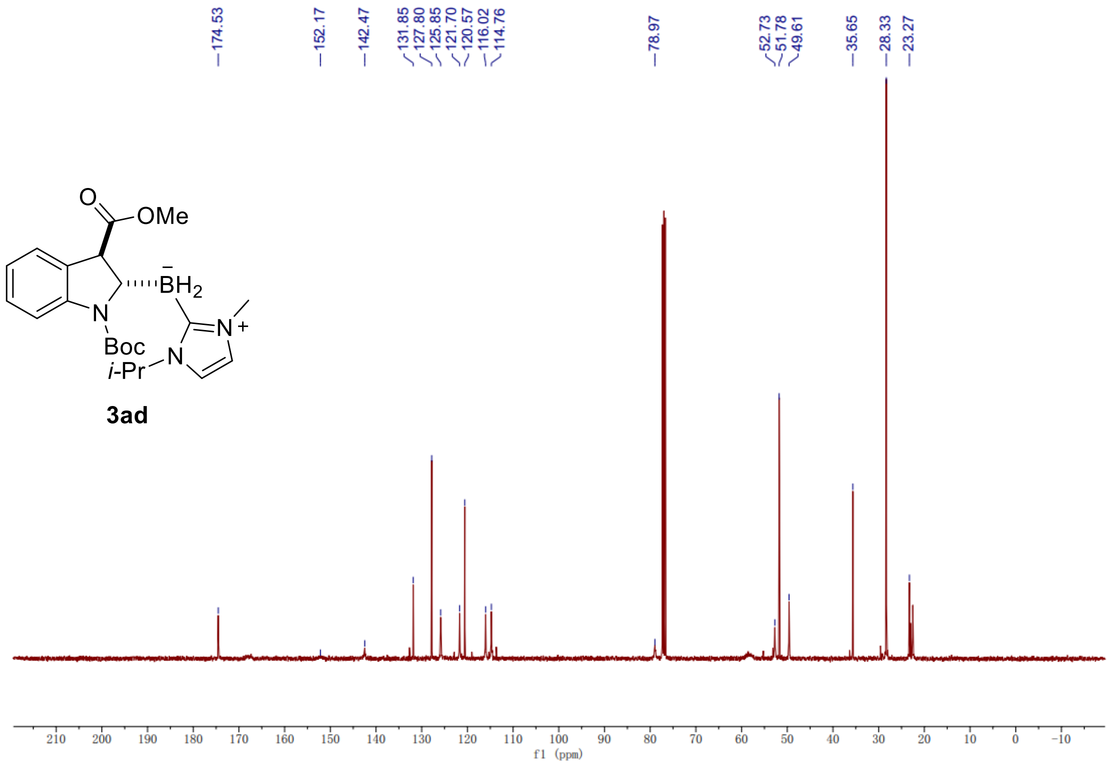

3ad

174.53
152.17
142.47
131.85
127.80
125.85
121.70
120.57
116.02
114.76
78.97
52.73
51.78
49.61
35.65
28.33
23.27
f1 (ppm)

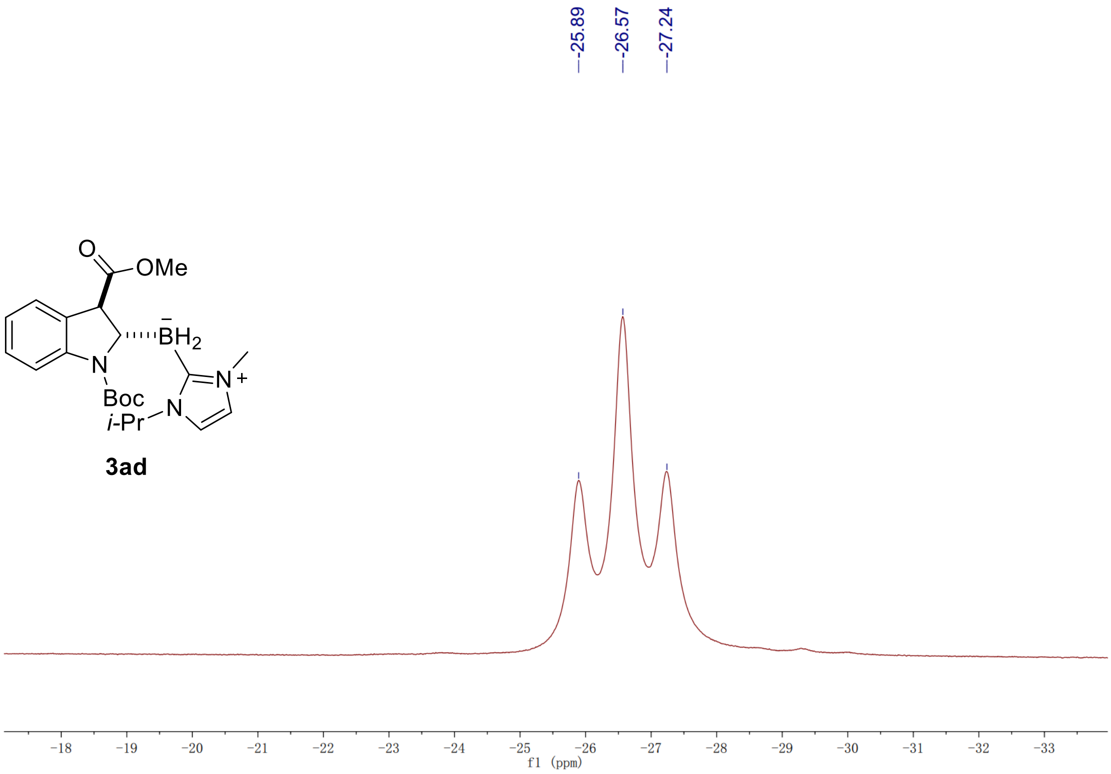

3ad

-25.89
-26.57
-27.24
f1 (ppm)

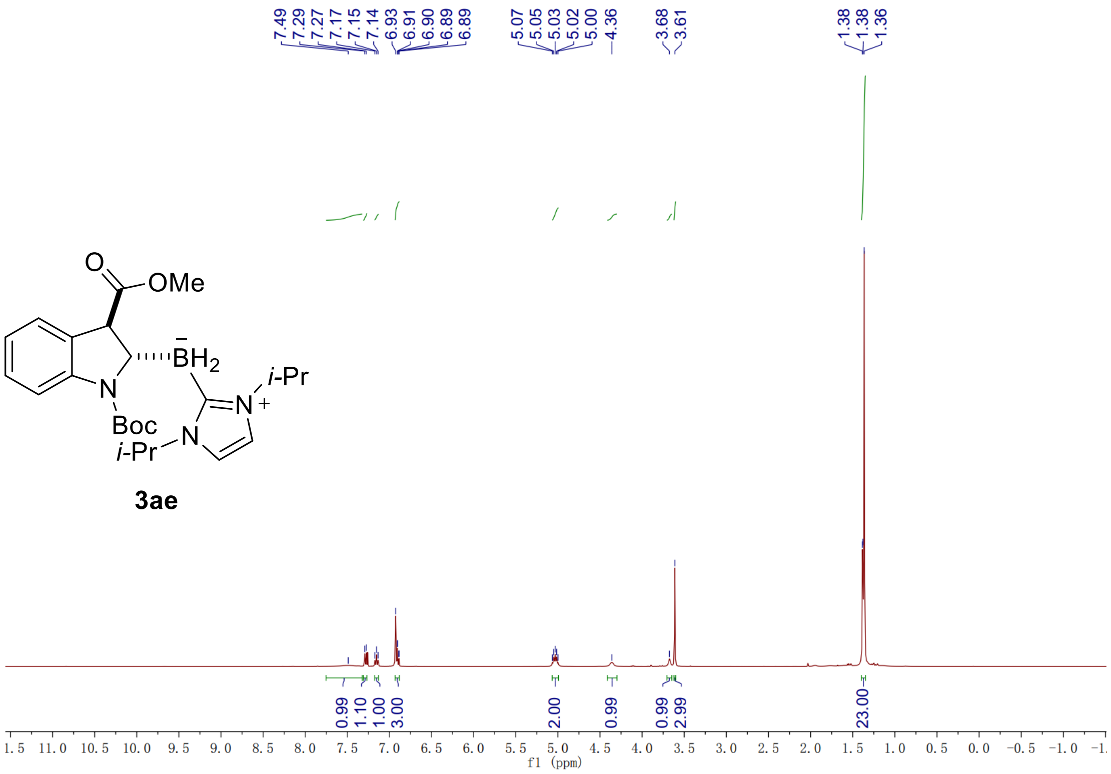
7.49 7.29 7.27 7.17 7.15 7.14 6.93 6.91 6.90 6.89 6.89
5.07 5.05 5.03 5.02 5.00 4.36
3.68 3.61
1.38 1.38 1.36
OMe
BH2
i-Pr
N+
N
Boc
i-Pr
N
3ae
0.99 1.10 1.00 3.00 2.00 0.99 0.99 2.99 23.00
f1 (ppm)

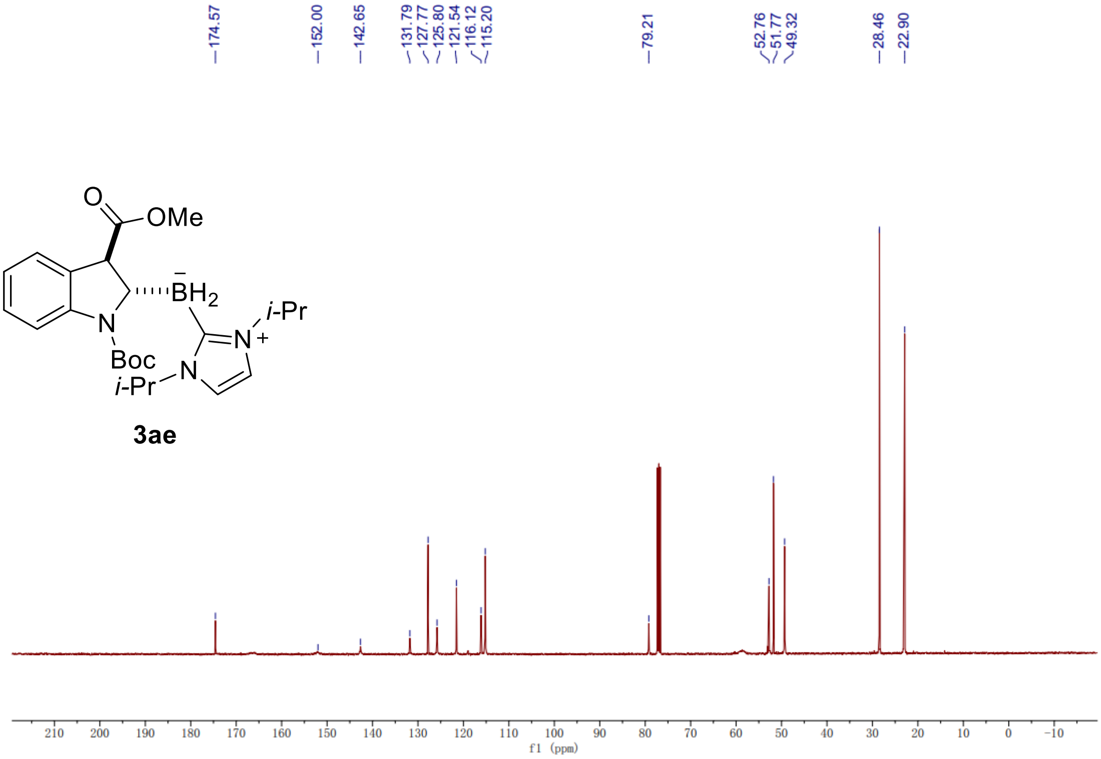
174.57
152.00
142.65
131.79 127.77 125.80 121.54 116.12 115.20
79.21
52.76 51.77 49.32
28.46 22.90
OMe
BH2
i-Pr
N+
N
Boc
i-Pr
N
3ae
f1 (ppm)

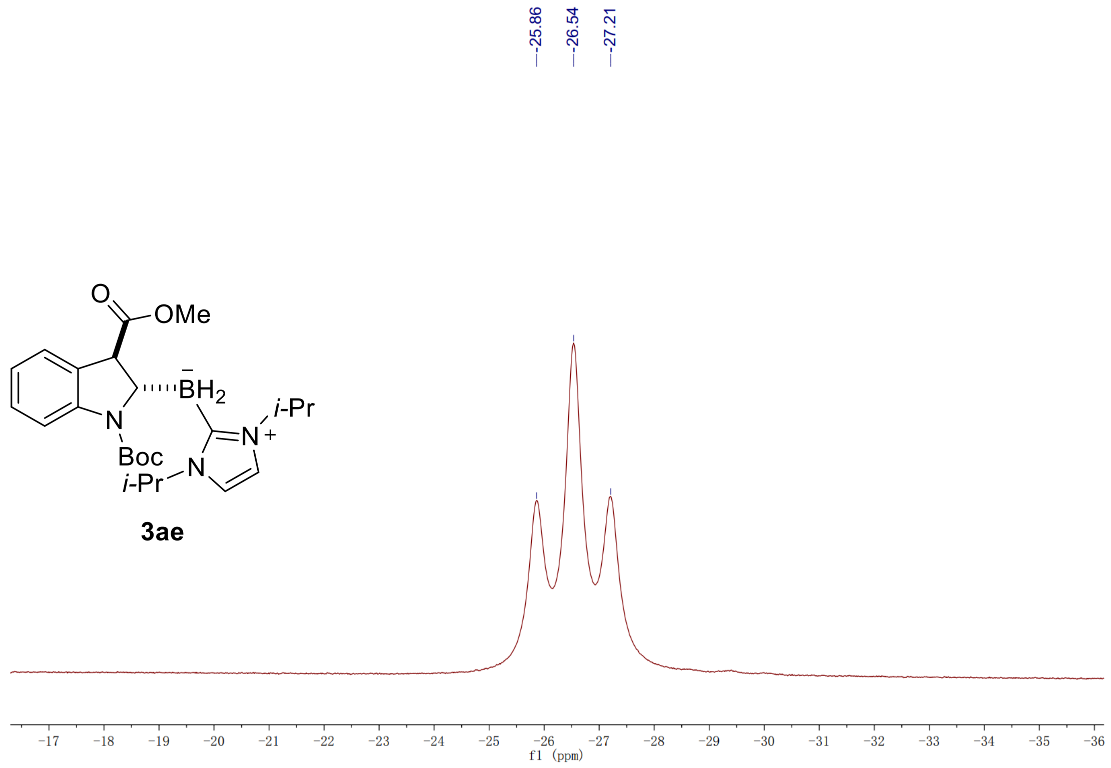

-25.86
-26.54
-27.21
O
OMe
BH2
i-Pr
N
N+
Boc
i-Pr
N
f1 (ppm)

3ae

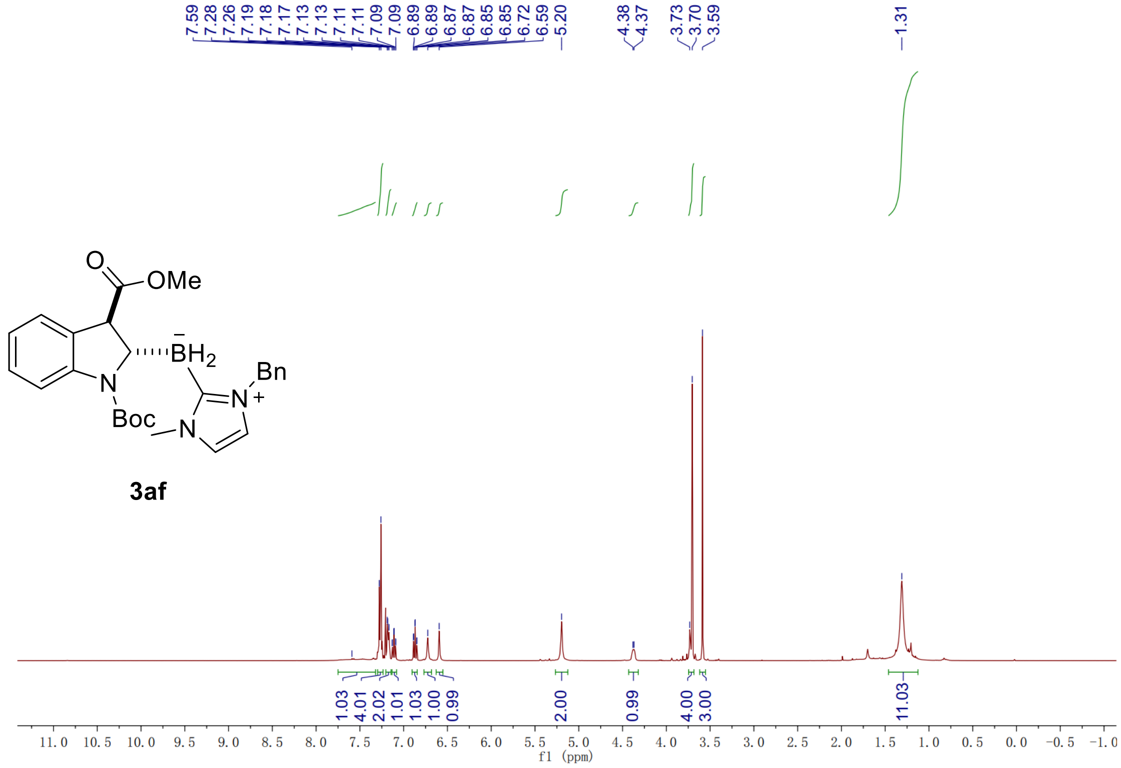

7.59
7.28
7.26
7.19
7.18
7.17
7.13
7.13
7.11
7.11
7.09
7.09
6.89
6.89
6.87
6.87
6.85
6.85
6.72
6.59
5.20
4.38
4.37
3.73
3.70
3.59
1.31
O
OMe
BH2
Bn
N
N+
Boc
N
1.03
4.01
2.02
1.01
1.03
1.00
0.99
2.00
0.99
4.00
3.00
11.03
f1 (ppm)

3af

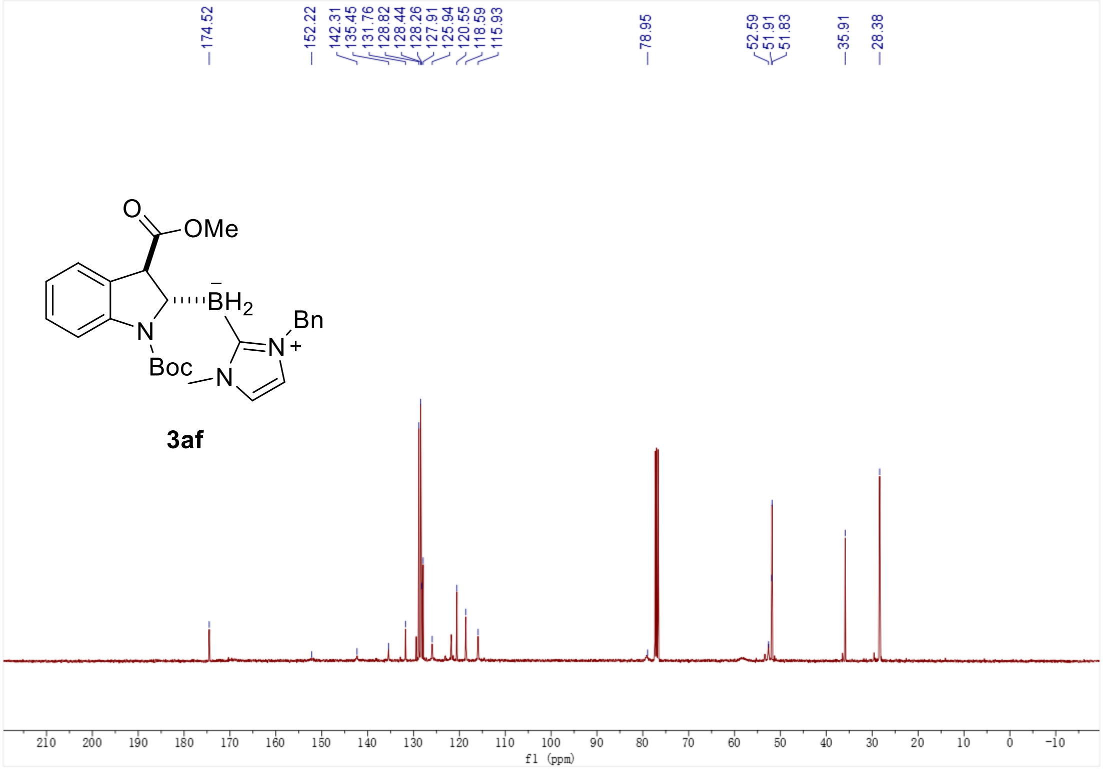

174.52
152.22
142.31
135.45
131.76
128.82
128.44
128.26
127.91
125.94
120.55
118.59
115.93
78.95
52.59
51.91
51.83
35.91
28.38
O
OMe
BH2
Bn
N
N+
Boc
N
3af
210 200 190 180 170 160 150 140 130 120 110 100 90 80 70 60 50 40 30 20 10 0 -10
f1 (ppm)

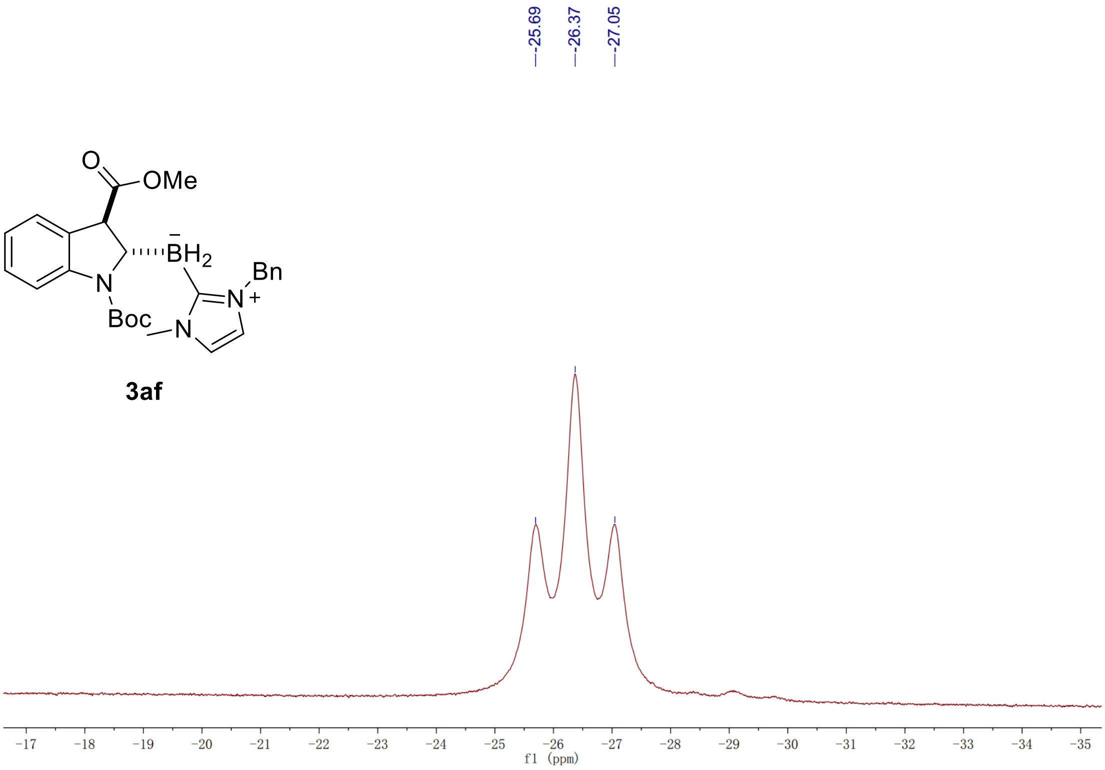

-25.69
-26.37
-27.05
O
OMe
BH2
Bn
N
N+
Boc
N
3af
-17 -18 -19 -20 -21 -22 -23 -24 -25 -26 -27 -28 -29 -30 -31 -32 -33 -34 -35
f1 (ppm)

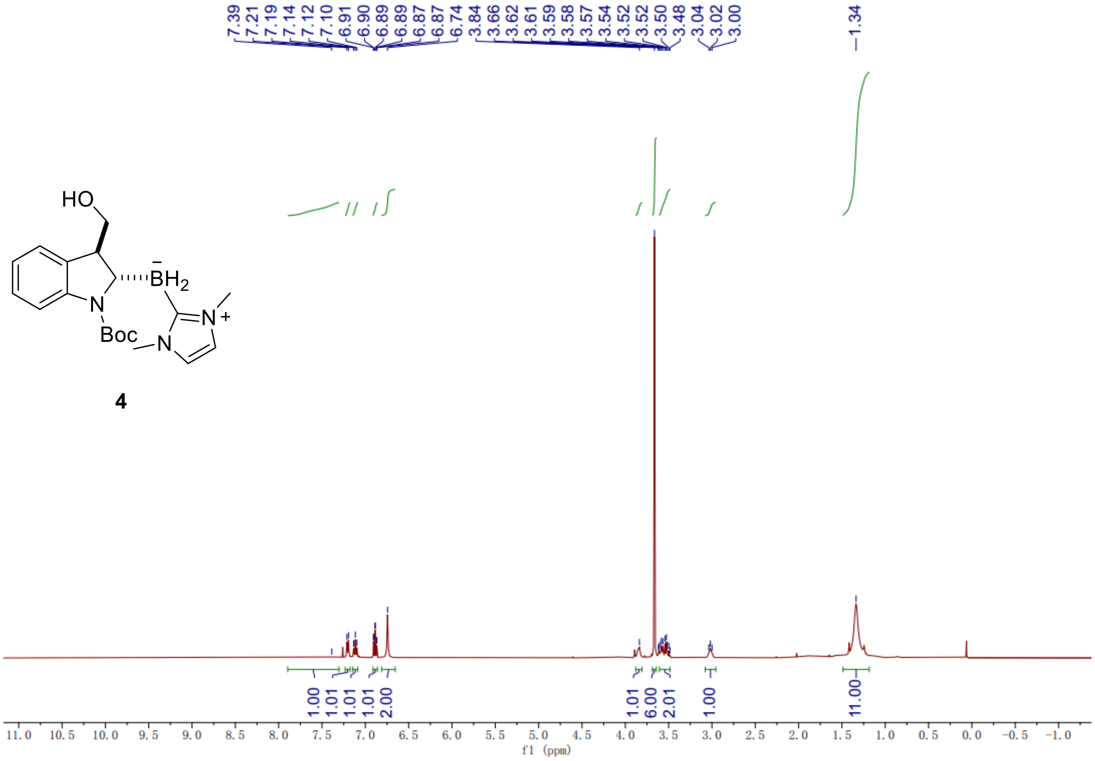

7.39
7.21
7.19
7.14
7.12
7.10
6.91
6.90
6.89
6.89
6.87
6.87
6.74
3.84
3.66
3.62
3.61
3.59
3.58
3.57
3.54
3.52
3.52
3.50
3.48
3.04
3.02
3.00
1.34
HO
BH2
N
Boc
N
N+
4
1.00
1.01
1.01
1.01
2.00
1.01
6.00
2.01
1.00
11.00
f1 (ppm)

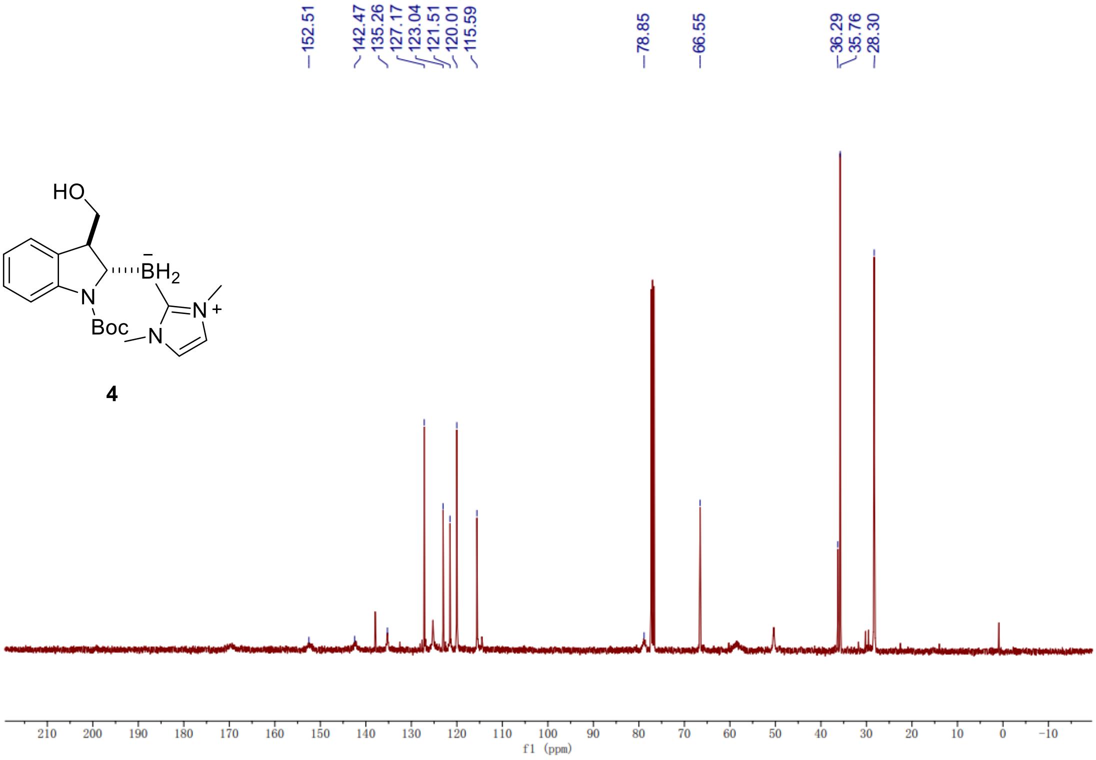

152.51
142.47
135.26
127.17
123.04
121.51
120.01
115.59
78.85
66.55
36.29
35.76
28.30
HO
BH2
N
Boc
N
N+
4
f1 (ppm)

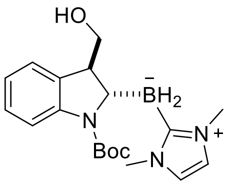
HO
BH2
N
Boc
N
N+

4

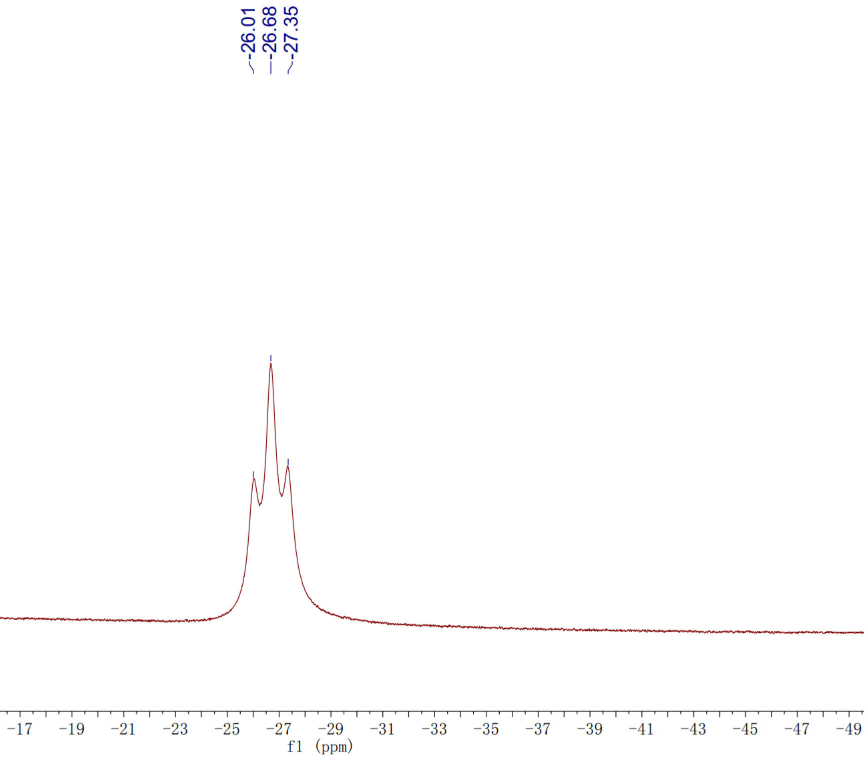
-26.01
-26.68
-27.35
-9 -11 -13 -15 -17 -19 -21 -23 -25 -27 -29 -31 -33 -35 -37 -39 -41 -43 -45 -47 -49
f1 (ppm)

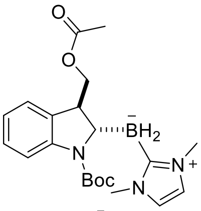
O
O
BH2
N
Boc
N
N+

5

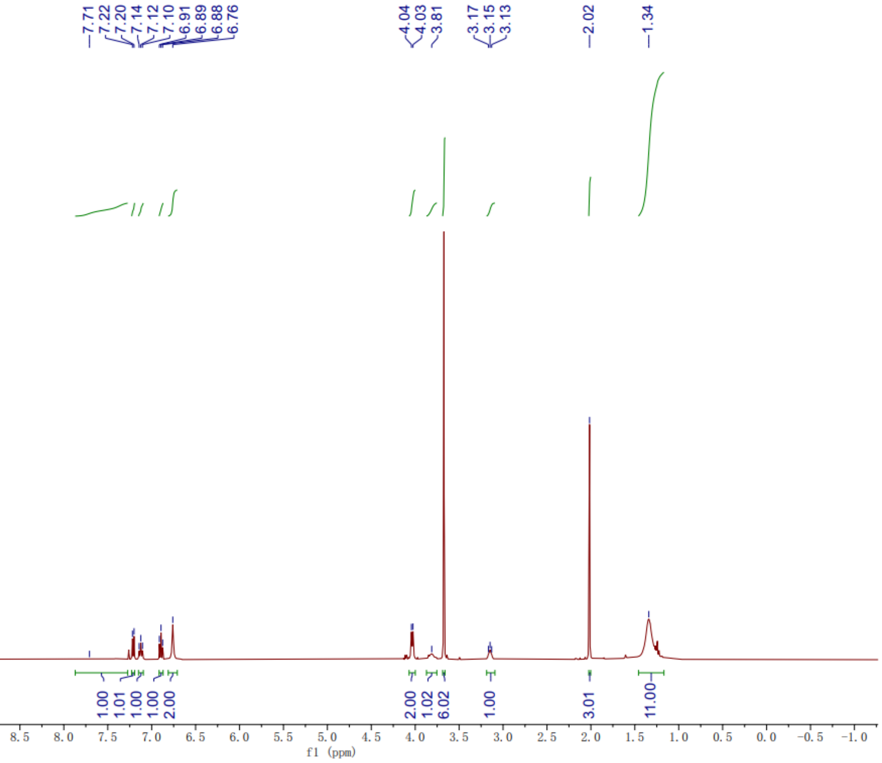
7.71
7.22
7.20
7.14
7.12
7.10
6.91
6.89
6.88
6.76
4.04
4.03
3.81
3.17
3.15
3.13
2.02
1.34
1.00
1.01
1.00
1.00
2.00
2.00
1.02
6.02
1.00
3.01
11.00
11.0 10.5 10.0 9.5 9.0 8.5 8.0 7.5 7.0 6.5 6.0 5.5 5.0 4.5 4.0 3.5 3.0 2.5 2.0 1.5 1.0 0.5 0.0 -0.5 -1.0
f1 (ppm)

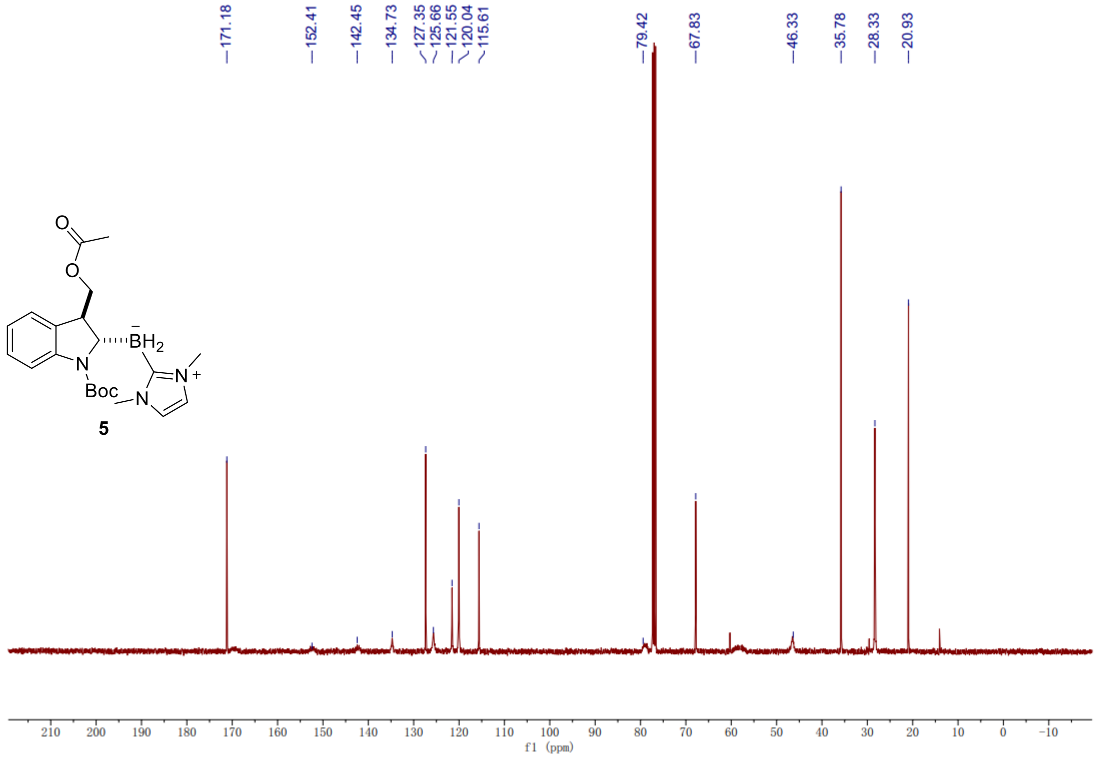
171.18
152.41
142.45
134.73
127.35
125.66
121.55
120.04
115.61
79.42
67.83
46.33
35.78
28.33
20.93
O
O
BH2
N
N+
Boc
N
5
210 200 190 180 170 160 150 140 130 120 110 100 90 80 70 60 50 40 30 20 10 0 -10
f1 (ppm)

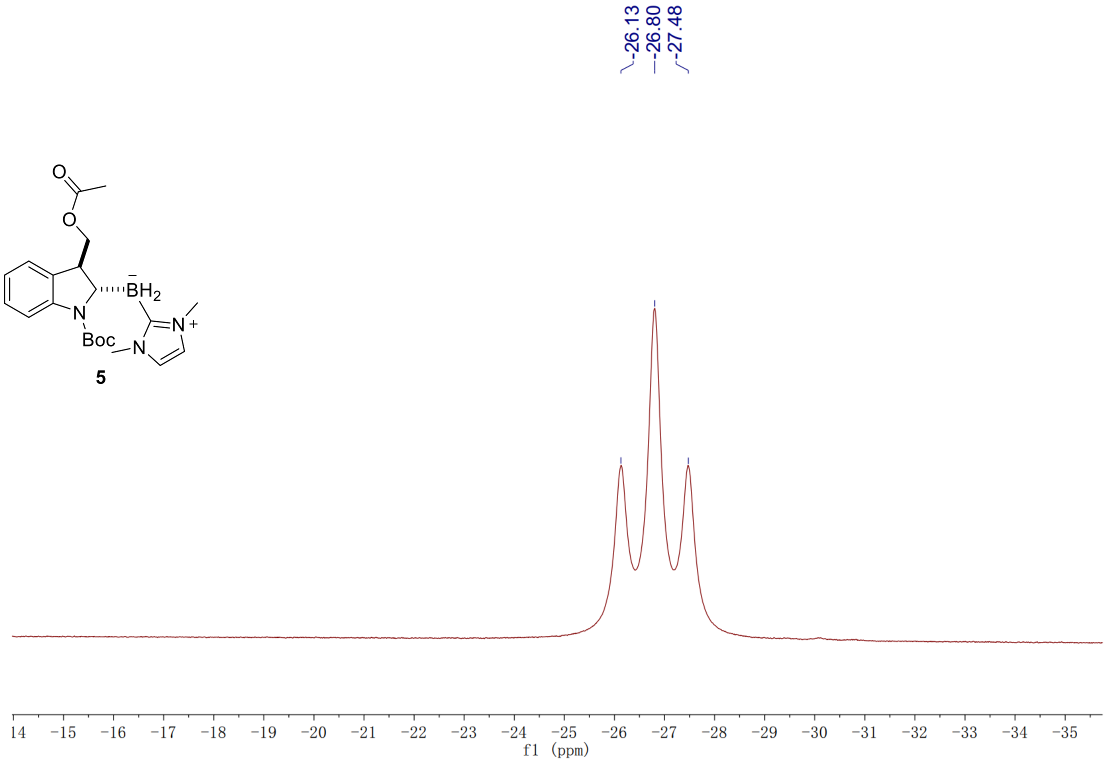
-26.13
-26.80
-27.48
O
O
BH2
N
N+
Boc
N
5
14 -15 -16 -17 -18 -19 -20 -21 -22 -23 -24 -25 -26 -27 -28 -29 -30 -31 -32 -33 -34 -35
f1 (ppm)

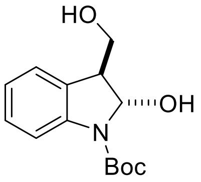

6

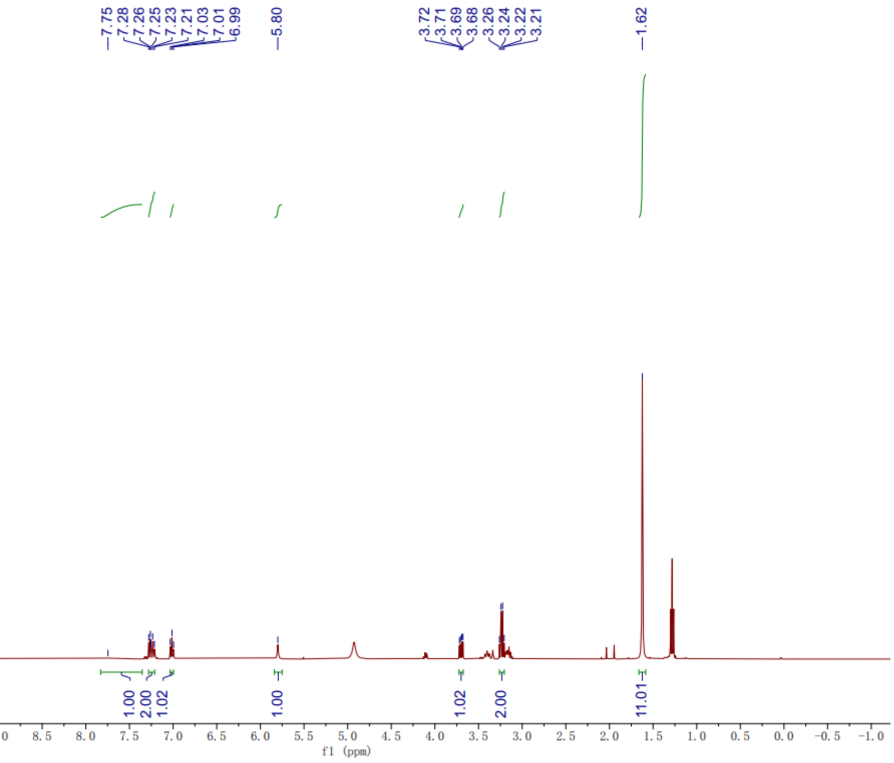
7.75
7.28
7.26
7.25
7.23
7.21
7.03
7.01
6.99
5.80
3.72
3.71
3.69
3.68
3.26
3.24
3.22
3.21
1.62
1.00
2.00
1.02
1.00
1.02
2.00
11.01
f1 (ppm)

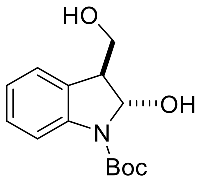

6

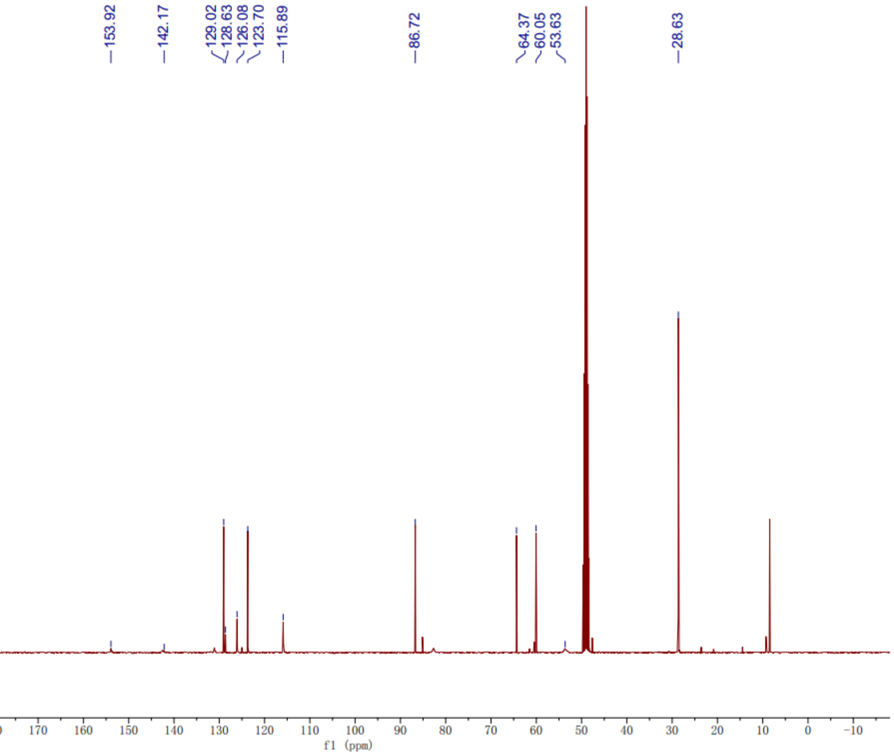
153.92
142.17
129.02
128.63
126.08
123.70
115.89
86.72
64.37
60.05
53.63
28.63
f1 (ppm)

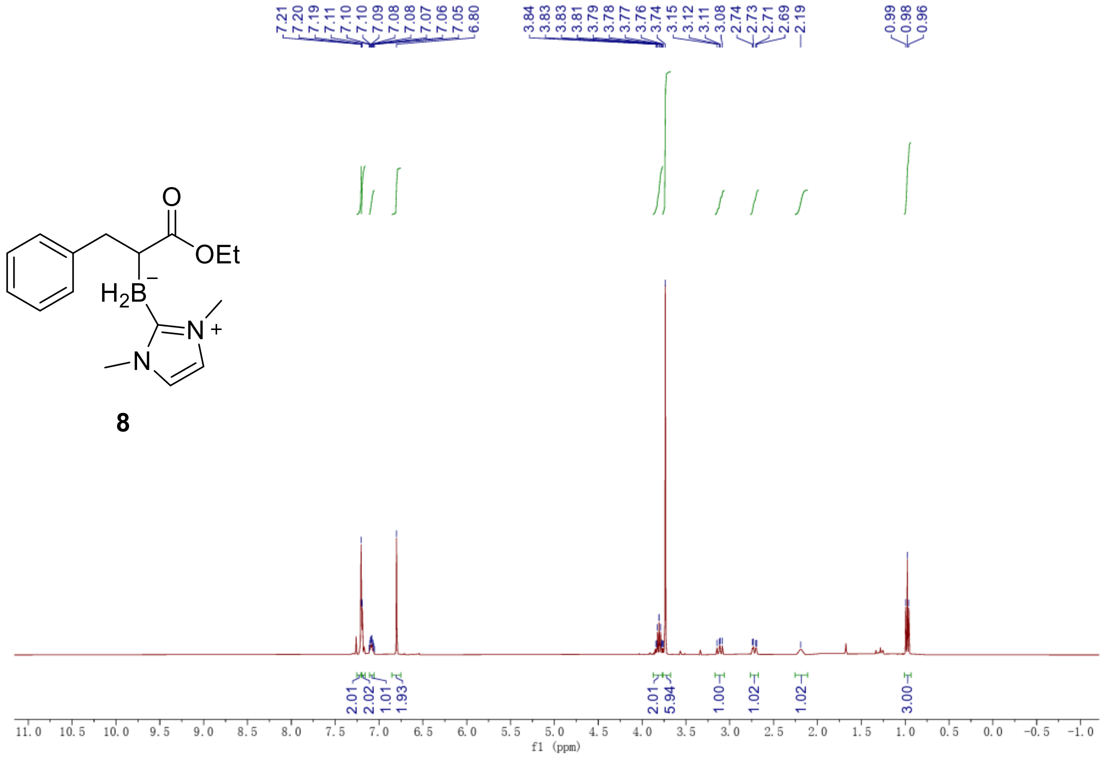

8

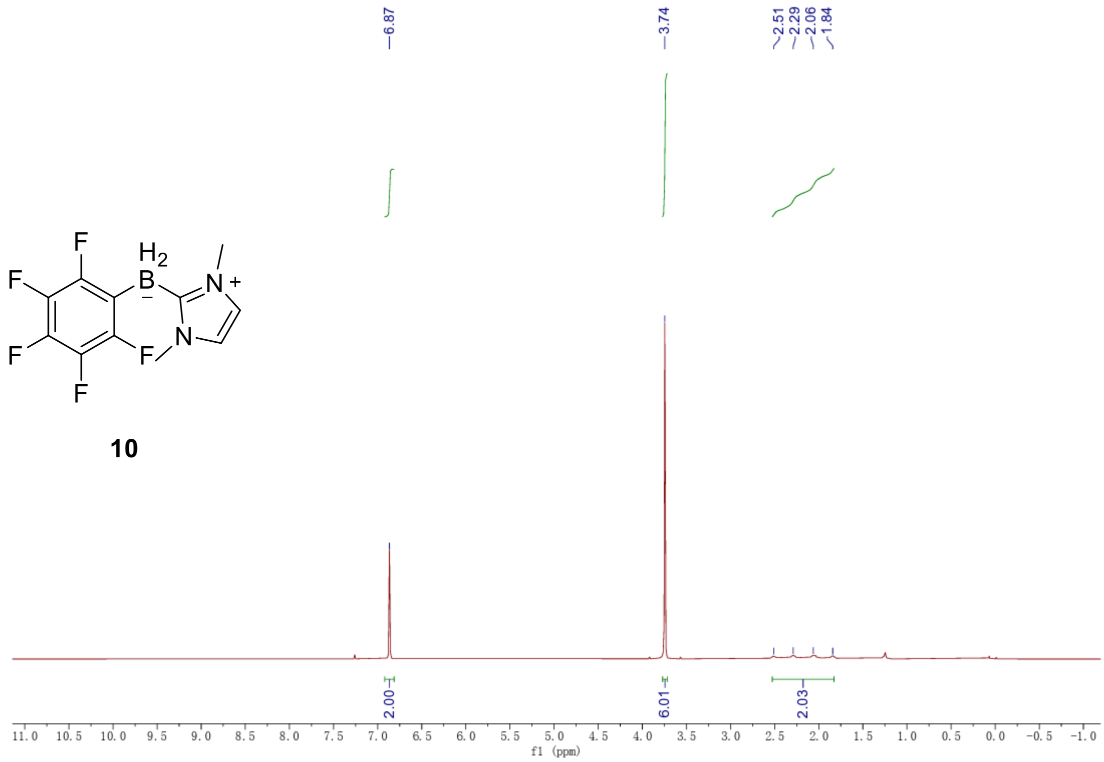

10

References

1. Chen, L., Shen, J., Gao, Q., Xu, S. Synthesis of cyclic chiral alpha-amino boronates by copper-catalyzed asymmetric dearomative borylation of indoles. *Chem. Sci.* **9**, 5855-5859 (2018).

2. Rao, C., Lentz, D., Reissig, H. Synthesis of polycyclic tertiary carbinamines by samarium diiodide mediated cyclizations of indolyl sulfinyl imines. *Angew. Chem.* **54**, 2750-2753 (2015).

3. Zhang, Y., Ji, P., Gao, F., Huang, H., Zeng, F., Wang, W. Photoredox asymmetric nucleophilic dearomatization of indoles with neutral radicals. *ACS Catal.* **11**, 998-1007 (2021).

4. Wang. L., Shao, Y., Liu, Y. Nucleophilic addition of Grignard reagents to 3-acylindoles: stereoselective synthesis of highly substituted indoline scaffolds. *Org. Lett.* **14**, 3978 (2012).

5. Wang, F., Nishimoto, Y., Yasuda, M. Lewis Acid-Catalyzed Diastereoselective C-C Bond Insertion of Diazo Esters into Secondary Benzylic Halides for the Synthesis of alpha,beta-Diaryl-beta-haloesters. *Angew. Chem.* **61**, e202204462 (2022).

6. Bellotti, P. *et al.* Visible-light photocatalyzed peri-(3 + 2) Cycloadditions of Quinolines. *J. Am. Chem. Soc.* **144**, 15662-15671 (2022).

7. Zhao, Y., Su, Y., Li, X., Yang, L., Huang, M., Zhu, S. Dirhodium-Catalyzed Enantioselective B-H Bond Insertion of gem-Diaryl Carbenes: Efficient Access to gem-Diarylmethine Boranes. *Angew. Chem.* **60**, 24214-24219 (2021).

8. Ren, S.-C. *et al.* Regioselective radical alpha-borylation of alpha,beta-unsaturated carbonyl compounds for direct synthesis of alpha-borylcarbonyl molecules. *Nat. Commun.* **10**, 1934 (2019).

9. Xu, W., Jiang H., Leng J., Ong H. W., Wu, J. Visible-light-induced selective defluoroborylation of polyfluoroarenes, gem-difluoroalkenes, and trifluoromethylalkenes. *Angew. Chem. Int. Ed.* **59**, 4009 (2020).

10. Frisch, M. J.; Trucks, G. W.; Schlegel, H. B.; Scuseria, G. E.; Robb, M. A.; Cheeseman, J. R.; Scalmani, G.; Barone, V.; Mennucci, B.; Petersson, G. A.; Nakatsuji, H.; Caricato, M.; Li, X.; Hratchian, H. P.; Izmaylov, A. F.; Bloino, J.; Zheng, G.; Sonnenberg, J. L.; Hada, M.; Ehara, M.; Toyota, K.; Fukuda, R.; Hasegawa, J.; Ishida, M.; Nakajima, T.; Honda, Y.; Kitao, O.; Nakai, H.; Vreven, T.; Montgomery, J. A., Jr.; Peralta, J. E.; Ogliaro, F.; Bearpark, M.; Heyd, J. J.;

Brothers, E.; Kudin, K. N.; Staroverov, V. N.; Keith, T.; Kobayashi, R.; Normand, J.; Raghavachari, K.; Rendell, A.; Burant, J. C.; Iyengar, S. S.; Tomasi, J.; Cossi, M.; Rega, N.; Millam, J. M.; Klene, M.; Knox, J. E.; Cross, J. B.; Bakken, V.; Adamo, C.; Jaramillo, J.; Gomperts, R.; Stratmann, R. E.; Yazyev, O.; Austin, A. J.; Cammi, R.; Pomelli, C.; Ochterski, J. W.; Martin, R. L.; Morokuma, K.; Zakrzewski, V. G.; Voth, G. A.; Salvador, P.; Dannenberg, J. J.; Dapprich, S.; Daniels, A. D.; Farkas, O.; Foresman, J. B.; Ortiz, J. V.; Cioslowski, J.; Fox, D. J. Gaussian 09, Revision D.01, Gaussian, Inc., Wallingford CT, 2013.

11. Grimme, S.; Antony, J.; Ehrlich, S.; Krieg, H. A consistent and accurate ab initio parametrization of density functional dispersion correction (DFT-D) for the 94 elements H-Pu. *J. Chem. Phys.* 132, 154104 (2010).

12. Marenich, A. V., Cramer, C. J., Truhlar, D. G. Universal solvation model based on solute electron density and on a continuum model of the solvent defined by the bulk dielectric constant and atomic surface tensions. *J. Phys. Chem. B,* 113, 6378 (2009).
